# High-depth whole-genome sequencing identifies structure variants, copy number variants and short tandem repeats associated with Parkinson’s disease

**DOI:** 10.1038/s41531-024-00722-1

**Published:** 2024-07-23

**Authors:** Chaodong Wang, Hankui Liu, Xu-Ying Li, Jinghong Ma, Zhuqin Gu, Xiuli Feng, Shu Xie, Bei-Sha Tang, Shengdi Chen, Wei Wang, Jian Wang, Jianguo Zhang, Piu Chan

**Affiliations:** 1 Department of Neurology & Neurobiology, Xuanwu Hospital of Capital Medical University, National Clinical Research Center for Geriatric Diseases, Beijing, 100053 China; 2 BGI-Shenzhen, Beishan Industrial Zone, Shenzhen, 518083 China; 3 National Human Genome Center in Beijing, Beijing Economic-Technological Development Zone, Beijing, 100176 China; 4 Department of Neurology, Xiangya Hospital, Central South University, State Key Laboratory of Medical Genetics, Changsha, China; 5 Department of Neurology, Ruijin Hospital Affiliated to Shanghai Jiao Tong University School of Medicine, Shanghai, China; 6 Hebei Industrial Technology Research Institute of Genomics in Maternal & Child Health, Shijiazhuang, 050000 China; 7 Advanced Innovation Center for Human Brain Protection, Capital Medical University, Beijing, China; 8 Clinical Center for Parkinson’s Disease, Capital Medical University, Key Laboratory for Neurodegenerative Disease of the Ministry of Education, Beijing Key Laboratory for Parkinson’s Disease, Beijing, China; 9 Beijing Institute of Brain Disorders, Collaborative Innovation Center for Brain Disorders, Capital Medical University, Beijing, China

**Keywords:** Parkinson's disease, Next-generation sequencing, Genome-wide association studies

## Abstract

While numerous single nucleotide variants and small indels have been identified in Parkinson’s disease (PD), the contribution of structural variants (SVs), copy number variants (CNVs), and short tandem repeats (STRs) remains poorly understood. Here we investigated the association using the high-depth whole-genome sequencing data from 466 Chinese PD patients and 513 controls. Totally, we identified 29,561 SVs, 32,153 CNVs, and 174,905 STRs, and found that CNV deletions were significantly enriched in the end-proportion of autosomal chromosomes in PD. After genome-wide association analysis and replication in an external cohort of 352 cases and 547 controls, we validated that the 1.6 kb-deletion neighboring *MUC19*, 12.4kb-deletion near *RXFP1* and GGGAAA repeats in *SLC2A13* were significantly associated with PD. Moreover, the *MUC19* deletion and the *SLC2A13* 5-copy repeat reduced the penetrance of the *LRRK2* G2385R variant. Moreover, genes with these variants were dosage-sensitive. These data provided novel insights into the genetic architecture of PD.

## Introduction

Parkinson’s disease (PD) is a common and fast-growing neurodegenerative disease with a strong genetic component^1^. Previous studies have identified 23 causative genes and over 90 susceptible variants for PD^2,3^. Most of the PD-associated variants were single nucleotide variants (SNVs) and small structural variations (SVs), insertions/deletions (Indels), and exonic rearrangements. Copy-number variants (CNVs) in common (*SNCA, PARK2*, and *DJ-1*)^4–8^ and novel genes (*VPS13C, SULT1A3/4*, and *DNAJB2*) have been associated with familial and early-onset PD^9–11^. In the most recent, rapidly increasing number of STRs (e.g., GCC repeats in the 5′UTR of *NOTCH2NLC* and *GIPC1*) have been associated with sporadic PD^12,13^. Interestingly, these repeats were also identified in a variety of neurodegenerative disorders and myopathy, such as neuronal intranuclear inclusion disease, essential tremor, multiple system atrophy, and oculopharyngodistal myopathy. Moreover, genome-wide association studies have identified PD-associated STRs nearby *NDUFAF2, TRIML2, MIRNA-129-1*, and *NCOR1*^14^. In addition, a large whole-genome sequencing (WGS) study showed high sensitivity and specificity for detecting the known repeat expansions associated with neurological repeat-expansion disorders including *AR, ATN1, ATXN1, ATXN2, ATXN3, ATXN7, C9orf72, CACNA1A, DMPK, FMR1, FXN, HTT*, and *TBP*^15^.

Structural variants (SVs) rearrange large segments of DNA from 50 bp, one kilobase (kb) to several megabases (Mb), and influence a high fraction of the human genome. SVs can be classified into mutational classes that include ‘unbalanced’ gains or losses of DNA (e.g., CNVs) and “balanced” rearrangements that occur without dosage alterations^16^, which have profound consequences in protein-coding genes and cis-regulatory architecture^17^, and are attributable to multiple germline and somatic human diseases^18,19^. SVs in some genetic loci (22q11.2 and 16p11.2) without a specific gene were identified to be associated with sporadic PD^20–22^.

The interest in studies for SVs have emerged most recently. WGS studies, including the UK Biobank sequencing and several other sequencing studies for over 10,000 genomes^23–25^, have led to the boosting of large-scale identification of human SVs and some of the data have been integrated into the gnomAD as a reference SV database. The SVs were estimated to be responsible for 25–29% of all rare protein-truncating events per genome, and strong correlations were found between natural selection against damaging SNVs and rare SVs^25^.

Of the large number of SNVs identified, G2385R in *LRRK2* has been considered the most popular variant associated with PD in Eastern Asians^26^. About 7% of old Chinese individuals carry the variant, and the life-time penetrance of the variant in the Chinese population was 25.9%^27^. The predisposing effect may be related to partial loss-of-function effects on the synaptic vesicle trafficking^28^. However, the penetrance of this variant was much lower than the G2019S, R1441C, and I2020T mutations, which have been mostly reported in Westerners^29,30^. The penetrance and age at onset (AAO) of the G2019S mutation has been proposed to be modified by multiple genetic or environmental factors^31,32^, while only one study outlined PD-associated SNPs modifying the penetrance of G2385R^33^. Moreover, it has not been well explored whether the effect of SNVs can be modified by SVs.

With WGS data and newly developed algorithms and databases, all types of SVs, CNVs, and STRs can be detected in an individual genome and associated with diseases^24^. Here, we investigated the populational distribution of SVs, CNVs, and STRs and their contribution to PD, using the high-depth WGS data from a Chinese cohort of PD patients and age-matched controls. We also explored the expression of genes nearby these variants in the human brain cells and investigated the modifiers for the penetrance of the *LRRK2* G2385R variant.

## Results

### The study cohort and analytic pipelines of the WGS data

**Fig. 1:**
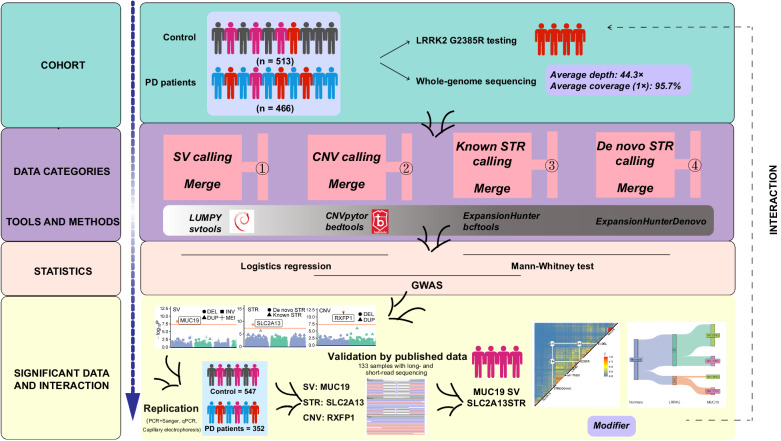
Workflow of the study. The study included 466 PD patients and 513 controls. The whole-genome sequencing data were used for analyzing structure variants (SVs), copy-number variants (CNVs), and known and de novo short-tandem repeats (kSTR and dSTR) with respective variant calling software. The replication study was performed for the SVs, CNVs, and STRs in another cohort of 352 PD patients and 547 controls. Further validation of the existence of the associated SV and STR was conducted using the most recently published long-read and short-read sequencing data. Interactions between the *LRRK2* G2385R variant and the *MUC19* SV and *SLC2A13* STR were analyzed by comparing the variant carriers in different molecular subtypes of PD cases and controls.

The study included the discovery and the external validation cohorts. The discovery cohort consists of 466 PD patients and 513 healthy elderlies from the Northern and Southern Chinese provinces. The average age of PD patients and healthy controls were 61.3 ± 8.5 and 71.8 ± 6.7 years, respectively. The external validation cohort consists of 352 cases and 547 controls, which were aged 62.3 ± 9.3 and 69.6 ± 5.2 years, respectively (Supplementary Table 1). All participants in the discovery cohort had a mean of 44.3× high-depth whole-genome sequencing (WGS) data (Supplementary Table 2 and Supplementary Fig. 1), the SVs, CNVs, and STRs were called and analyzed by a reliable strategy and software pipelines, including LUMPY, Svtools, CNVpytor, ExpansionHunter and ExpansionHunterDenovo. Further validation of the *MUC19* SV and *SLC2A13* STR was conducted via Sanger sequencing and in-depth analyses of 133 samples with published long-read and short-read sequencing data. Among all the participants, 56 cases and 142 controls were previously identified to carry the *LRRK2* G2385R variant, which were used for the analysis of the interaction between the variant and PD-associated SV and STR (Fig. 1).

### SVs, CNVs, and STRs identified in PD cases and controls

**Fig. 2:**
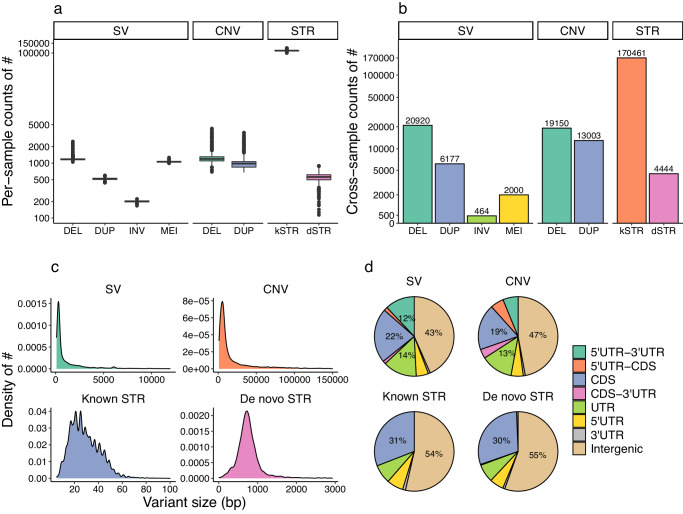
Summary of the identified variants. Number of variants per sample was displayed by variant type (**a**). After quality control and combination, the variant matrix of all the individual vcf files were merged and the number of variants were counted as cross-sample counts (**b**). The densities of variant sizes of the SVs, CNVs, kSTRs, and dSTRs (**c**) and the regions (**d**) of the variants were displayed.

In the WGS data of the discovery study, we identified 3120 reliable SVs per-genome, including 1191 deletions (DEL), 529 duplications (DUP), 203 inversions (INV), and 1197 mobile-element insertions (MEI). We also detected a mean of CNVs (including 1261 DEL and 1029 DUP), and 110,000 known and 554 de novo STRs (Fig. 2a). In all the 979 cases and controls, we obtained 29,561 SVs, 32,153 CNVs, 174,905 STRs, respectively (Fig. 2b). The sizes of CNVs were much larger than those of SVs, and the sizes of de novo STRs were much larger than those of known STRs, which may be determined by the corresponding calling methods (Fig. 2c). With the annotation by AnnotSV, 19–31% of the variants located in the coding sequence (CDS), 43–55% in the intergenic regions, and the remaining variants near the non-coding regions of genes (Fig. 2d).

We compared the SV characteristics of count, size, and minor allele frequency (MAF) with Abel’s study^24^. Per-sample SV counts decreased with the decrease of SV MAFs (Supplementary Fig. 2a) and the sizes of the SVs increased with the decrease of their MAFs (Supplementary Fig. 2b). Moreover, 62% (18,325) SVs in our study had at least 1 bp overlapped with the variants revealed in Abel’s study. Of these variants, 36% (6584) had high (≥80% overlapping of sequence) and 64% (11,741) with low (<80%) identity with the study (Supplementary Fig. 2c). According to the MAFs, the variants with high identity were mostly identified in the common variants (>0.05), while those with low identity and novel variants were identified in low frequency (0.01–0.05) and/or rare variants (<0.01) (Supplementary Fig. 2d). For the SVs with high identity, we revealed a high correlation between the sizes (*R*^2^ = 1, *β* = 1) (Supplementary Fig. 2e) and MAFs (*R*^2^ = 0.8, *β* = 1) (Supplementary Fig. 2f) in our and their study.

To explore the distribution of the SVs, CNVs, and STRs in phenotypes (case/control), birthplaces (North/South), sex (male/female), age (<68/≥68 years old) and sequencing depth (<45× / >45×), we performed the principal component analysis (PCA) for these variants between these groups. The results exhibited no obvious stratification of genetic background between individuals from Northern and Southern China, neither between cases and controls, age groups, or read-depths. However, the CNVs were unequally distributed between males and females, which came from the CNVs in the sex chromosomes (Supplementary Fig. 3a). When we excluded the CNVs from the sex chromosomes, no difference was shown between males and females (Supplementary Fig. 3b).

### CNV deletions were enriched in the end proportions of autosomal chromosomes in PD patients

**Fig. 3:**
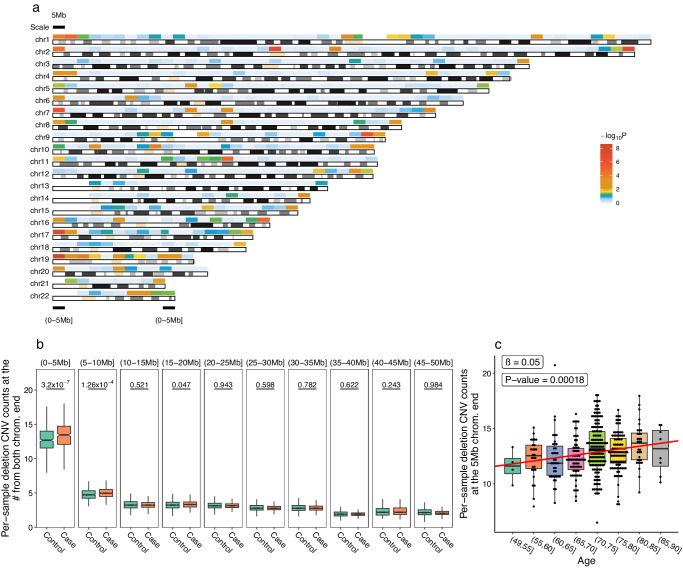
CNV burden in the terminal regions of autosomes in PD. The CNVs deletions in all regions (in 5 Mb windows) of autosomal chromosomes were compared in cases and controls and illustrated. *P* values in different colors indicated the significance of the differences (**a**). The differences were further compared for CNV deletions in the terminal 50 Mb regions (**b**). In the aging controls, the burden of deletions in the terminal 5 Mb region were evaluated in individuals with increased ages. The *P* for trend was indicated (**c**).

We investigated the distribution of the CNVs in the autosomal chromosome regions, which were split into 5 Mb windows. The 5 Mb-wise burdens for deletions between cases and controls were analysed and displayed for the 22 autosomes (Fig. 3a). Comparisons of the terminal 50 Mb in the chromosomes showed that the mean CNVs (13.42 ± 1.88) per sample at the terminal 5 Mb of chromosomes in PD cases were significantly higher than in controls (12.81 ± 1.96) (*P* = 3.78 × 10^−7^), and significant difference at the 5–10 Mb terminal region (*P* = 1.7 × 10^−4^), while the differences were not significant in other chromosomal regions (Fig. 3b). Interestingly, the burden of the CNVs in the terminal 5 Mb region increased with age increment in normal controls (*P* for trend: 0.00018) (Fig. 3c). However, PD patients did not harbor high burden of duplication CNVs compared with controls in the terminal region, and SVs distributed equally in cases and controls (Supplementary Fig. 4, *P* > 0.05).

### Genome-wide association analysis identifies SVs, CNVs, and STRs associated with PD

**Fig. 4:**
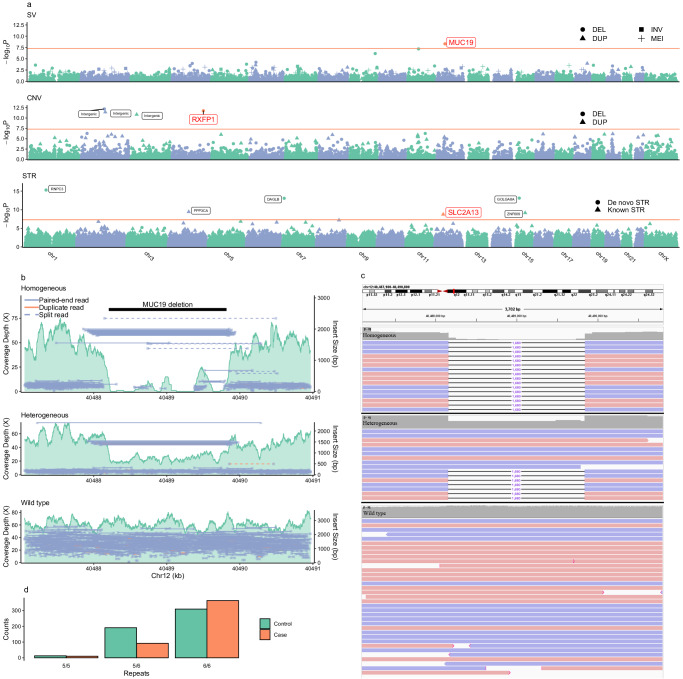
Association of SVs, CNVs, and STRs with Parkinson’s disease by genome-wide association (GWA) and replication studies. Manhattan plots of the GWASs for SVs, CNVs and STRs indicated by different icons. Red lines refer to genome-wide significant threshold (5 × 10^−8^). Names of genes/loci neighboring the variants significantly associated with PD only in the discovery study were indicated in small icons and denoted with small black letters, while those significantly associated in both the discovery and validation studies were indicated in yellow icons with big red letters (**a**). WGS short-read visualization for a single representative sample exhibit an SV event in the 12q12 (*MUC19)* locus. Read depth and insert size evidence supporting the heterozygous and homozygous deletion events (**b**). Alignment of the long-read and short-read sequences confirmed the genotypes of *MUC19* SV. The long-read sequences with a 1.6-kb gap and read-depth support the heterozygous and homozygous deletion events (**c**). The distribution of STR genotypes (6/6: wild-type; 5/6: heterozygous; 5/5 homozygous) with distinct numbers of the GGGAAA repeat neighboring *SLC2A13* STR between cases and controls was displayed (**d**).

Genome-wide association analysis was performed for the variants with MAF >1%, 11 novel signals (1 SV, 4 CNVs, and 6 STRs) passed the genome-wide significance threshold 5 × 10^−8^ (Fig. 4a). The SV (Fig. 4a upper and Table 1) is a 1.6 kb-deletion (chr12:40488206-40489818; 12q12) situated nearby the *MUC19* gene (MIM: 612170), which was part of a locus downstream of *LRRK2* gene (MIM: 609007) (Supplementary Fig. 5). The frequency of the deletion was 28.8% in cases and 45.0% in controls (OR = 0.49, 95% CI: 0.38–0.64; *P* = 4.48 × 10^−9^). The coverage depth and insert sizes of the wild-type, heterozygous, and homozygous 1.6 kb-deletion in *MUC19* was indicated in Fig. 4b.

**Table 1:** The SVs, CNVs, kSTRs, and dSTRs associated with Parkinson’s disease by discovery and replication studies

Location				Variant type		Size (bp)	*P* value	
Chromosome: start-end	Region	Gene	Transcript	Type	Duplication/deletion/repeat		Discovery	Validation
12:40488206-40489818	12q12	*MUC19*	Upstream	SV	Deletion	1613	4.84 × 10^−9^	6.10 × 10^−4^
2:117648701-117656300	2q14.1	-	Intergenic	CNV	Deletion	7600	6.50 × 10^−13^	4.27 × 10^−2^
2:122045201-122053900	2q14.3	-	Intergenic	CNV	Duplication	8700	3.79 × 10^−12^	5.96 × 10^−2^
3:28056701-28068100	3p24.1	-	Intergenic	CNV	Duplication	11,400	1.42 × 10^−11^	1.32 × 10^−1^
4:158425101-158437500	4q32.1	*RXFP1*	Intron	CNV	Deletion	12,400	1.74 × 10^−12^	1.74 × 10^−5^
12:40038759-40038789	12q12	*SLC2A13*	Intron	kSTR	Repeat (GGGAAA)	6	1.81 × 10^−9^	3.33 × 10^−18^
4:101336048-101336060	4q23	*PPP3CA*	Intron	kSTR	Repeat (AC)	2	3.65 × 10^−10^	2.46 × 10^−1^
15:64565232-64565256	15q22.31	*ZNF609*	Intron	kSTR	Repeat (TAT)	3	7.00 × 10^−10^	3.20 × 10^−1^
1:103525117-103525971	1p21.1	*RNPC3*	5′-UTRs	dSTR	Repeat (ACGGCGGGGCGGGGCGC)	17	7.48 × 10^−16^	8.37 × 10^−1^
15:34437067-34438029	15q14	*GOLGA8A*	5′-UTRs	dSTR	Repeat (ACGGCGGCGCGGCGAGG)	17	7.48 × 10^−14^	8.76 × 10^−1^
7:6420852-6422045	7p21.3	*DAGLB*	Exon	dSTR	Repeat (AGCGCGGGAGGCGCAGGC)	18	8.39 × 10^−14^	1.17 × 10^−2^

The CNV-GWAS analysis revealed four CNVs (two deletions and two duplications) were significantly associated with PD (Table 1). However, only the 12.4 kb-deletion (chr4:158425101-158437500; 4q32.1) encompasses the *RXFP1* gene (MIM: 606654), one member of the G-protein-coupled relaxin (GPCR) receptors widely expressed in the cortex and are involved in stress responses and memory and emotional processing. The other CNVs encompass miRNA and lncRNA sequences but no signals related to protein-coding genes. In addition, we did not identify the previously reported CNVs associated with PD, including those in *SNCA, PRKN,* and *VPS13C* genes (Supplementary Table 3).

The STR-GWAS analysis identified six genome-wide significant STR loci (Table 1), including known STR (kSTR) and de novo STR (dSTR). In the three kSTRs, the strongest signal located in *SLC2A13* (chr12: 40038759, 12q12: [GGGAAA]5/6, OR for the five-copy repeats: 0.47 (95% CI: 0.39–0.65); *P* = 1.81 × 10^−9^) (Fig. 4d and Table 1). This STR is situated in the intron 2 of the *SLC2A13* gene (MIM: 611036), which is located upstream of *LRRK2*. The other two known STRs are located in areas in the introns of *PPP3CA* (MIM: 114105; 4q23) and the *ZNF609* gene (MIM: 617474; 15q22.31). dSTRs with high-candidate coverage revealed three dSTRs with GWAS significant association with PD mapping to the 5′-UTR and CDS of *RNPC3* (chr1:103525117-103525971;1p21.1), *GOLGA8A* (15:34437067-34438029; 15q14), and *DAGLB* (chr7:6420852-6422045; 7p21.3) gene, respectively (Table 1). The analysis did not identify the most recently reported PD-associated STRs in *ATXN2, ATXN3, NOTCH2NLC*, and *GIPC1* gene (Supplementary Fig. 6).

### Validation of the GWAS-identified SV, CNVs, and STRs

To validate the association of all 11 SV, CNVs, and STRs with PD, we performed a comprehensive replication study in an external cohort of 352 cases and 547 controls. By PCR and Sanger sequencing, the 1.6 kb-deletion neighboring *MUC19* was validated in samples with the deletion detected by WGS in the discovery study (Supplementary Fig. 7a, b). The qPCR assay confirmed the existence of all four CNVs, and the Sanger sequencing and capillary electrophoresis validated the existence of three kSTRs and three dSTRs in each region or gene. The association analysis for the replications showed that the 1.6 kb-deletion (SV) neighboring *MUC19* (*P* = 6.10 × 10^−4^), the 12.4 kb-deletion (CNV) neighboring *RXFP1* (*P* = 1.74 × 10^−5^), and the five-copy GGGAAA repeats in *SLC2A13* (*P* = 3.33 × 10^−18^) were significantly associated with PD, while the remaining eight variants were not associated with the disease (*P* > 0.05/11 = 0.0045) (Fig. 4a and Table 1).

### Further validation of the *SLC2A13* STR and the *MUC19* SV

For further verification of the *SLC2A13* STR, we first performed the HipSTR analyses for our WGS data and found that 96.1% of the repeat sizes were consistent with those analyzed using the Expansionhunter tool (Table 2). In addition to the PCR and Sanger sequencing as described above, we validated the genotypes of the STR by repeat-primed PCR that showed genotypes consisting of 5- and/or 6-repeat alleles (Supplementary Fig. 8). Finally, the 133 samples with both long-read (PacBio and/or Ont) and short-read (WGSs) sequencing data from most recently published studies (Supplementary Table 4) were analyzed. The data showed a 96.5% coincidence rate between the genotypes determined by both sequencing platforms (Table 2).

**Table 2:** The accuracies for the validations of *MUC19* SV and *SLC2A13* STR

Validation platform	Genotype	Numbers of individuals with the Short-read WGS-determined genotypes			Accuracy/coincidence
		Wild type	Heterozygous	Homozygous	
***MUC19*** **deletion SV**					
Sanger	0|0	120	4	1	97.9%
	0|1	0	116	1	
	1|1	0	0	39	
Pacbio/ont	0|0	68	0	0	98.5%
	0|1	2	52	0	
	1|1	0	0	11	
***SLC2A13*** **GGGAAA repeat**					
HipSTR	6|6	372	9	0	96.1%
	5|6	1	178	0	
	5|5	0	13	16	
Pacbio/ont	6|6	78	1	0	96.5%
	5|6	1	28	2	
	5|5	0	0	3	

Genotypes for the *MUC19* deletion SV: 0|0: wild-type; 0|1: Heterozygous; 1|1: homozygous; for the SLC2A13 GGGAAA repeat: 5|5: homozygous for the 5-repeat, 5|6: heterozygotes for the 5- and 6-repeat; 6|6: homozygotes for the 6-repeat allele. Pacbio/ont: long-read sequencing using the PacBio and Oxford Nanopore Technologies platform. In the *SLC2A13* GGGAAA repeat validation using the Pacbio/ont data, 20 samples with ultra-low depth at this locus were not included.

We also validated the genotype of *MUC19* deletion via Sanger sequencing and re-analysis of the published long-read and short-read sequencing data. In the Sanger sequencing study, we employed a two-step strategy. The first step was similar to that described above, in which the PCR primer (primer 1) was designed to span the 1.6-kb-deletion region. The amplification can span the breakpoint in samples with the deletion, while cannot span this region in wild-type samples (Supplemental Fig. 7a, b). However, this primer cannot differentiate the heterozygous deletion from the homozygous deletion. To address this problem, we designed an additional primer (primer 2) to amplify a fragment within the deleted fragment. This primer cannot amplify the sequence in samples with the homozygous deletion, while it can amplify the normal allele in samples with the heterozygous deletion (Supplemental Fig. 7c, d). Testing with this strategy in 281 samples with high-quality DNA (120 wild-type, 120 heterozygotes, and 41 homozygotes detected by the WGS study, and using the Sanger sequencing-determined genotypes as the “truth” set, the accuracy of the WGS-detected genotypes was 97.9% (Table 2). To further validate the existence of the deletion, we aligned the long-read and short-read sequences from the 133 samples to the sequence of the *MUC19* SV. The alignments of long-read sequences generated a very clear depiction of the breakpoints of heterozygous or homozygous 1.6-kb-deletion (Fig. 4c). Using the genotype of SV detected by long-read data as an additional “truth” set, the accuracy for the WGS-predicted genotypes was 98.5% (DataSet; Table 2).

### Modification of the risk of *LRRK2* G2385R by the SV near *MUC19* and STR in *SLC2A13*

**Fig. 5:**
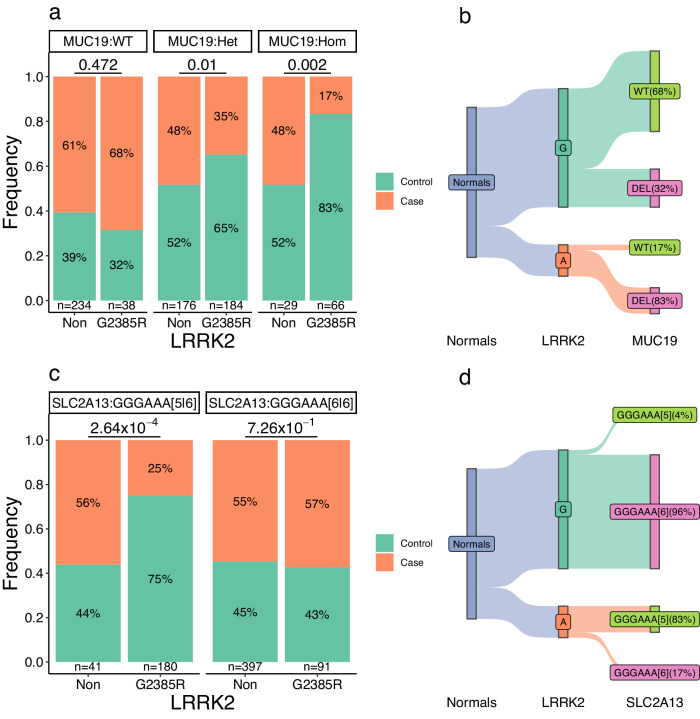
Modification of *LRRK2* G2385R variant by the *MUC19* SVs and *SLC2A13* STR. Among individuals with the wild-type (WT) and heterozygous (Het) and homozygous (Hom) deletion near *MUC19* (**a**) and 5/5 and 6/6 genotypes of the GGGAAA repeat in *SLC2A13* (**c**), the frequency of PD patients was compared in carriers and non-carriers for the *LRRK2* G2385R (c.7153G>A) variant. In contrast, normal carriers for the major G and minor A allele of the G2385R variant were analyzed with the stratification of the allele with wild-type or deletion near *MUC*19 (**b**) and 5- or 6-copy of repeat in the *SLC2A13* gene (**d**).

Since the PD-associated *MUC19* deletion and *SLC2A13* STR map closely to *LRRK2*, we asked whether these variants were associated with PD dependently or independently on the effect of *LRRK2* G2385R (c.7153G>A) variant. Compared with non-carriers, the G2385R carriers simultaneously carrying the heterozygous (Het) and homozygous (Hom) *MUC19* deletion had a significantly lower chance of developing PD than those carrying the wild-type (WT) *MUC*19 deletion (*P* < 0.05) (Fig. 5a). In normal controls, 32% of the carriers for the wild-type G allele simultaneously carried the *MUC19* deletion, while 83% of those with the mutated A allele simultaneously carried the deletion (Fig. 5b), suggesting that the *MUC19* deletion negatively modifies the penetrance of the mutated allele of G2385R.

Similarly, the G2385R carriers simultaneously carried the five-copy GGGAAA repeats, but not the six-copy repeats in *SLC2A13*, had significantly lower chance (*P* = 2.64 × 10^−4^) for developing PD than the non-carriers (Fig. 5c). Among the normal controls with the wild-type G allele of G2385R, only 4% simultaneously carried the five-copy allele of the STR, contrasting to 83% carried the allele in those with the mutant A allele (Fig. 5d), suggesting that the 5-copy allele negatively modifies the penetrance of the G2385R variant.

### The enriched expression of the PD-associated genes in brain cells

**Fig. 6:**
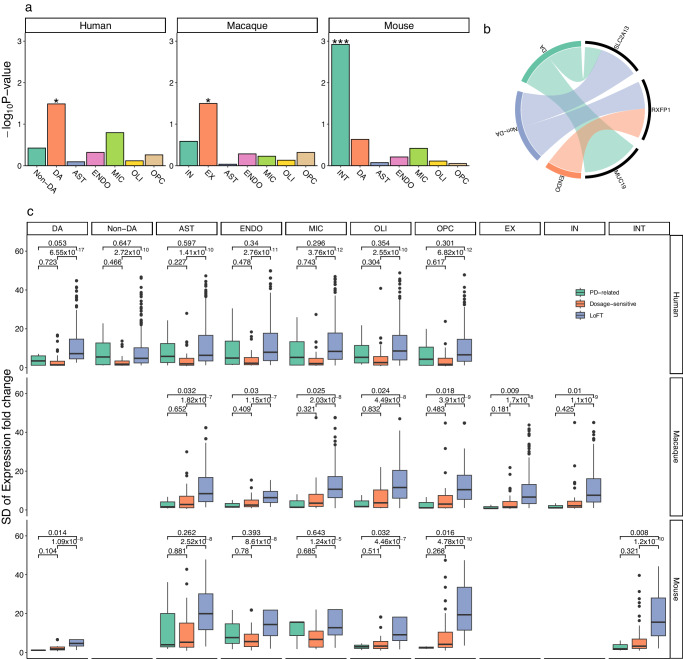
The enriched expression of the PD-associated genes in brain cells. Cell-type enrichment analysis using the human, macaque, and mouse brain single-cell transcriptomic data. The expression levels of genes with PD-associated variants in neurons (dopaminergic and non-dopaminergic) and other brain cells (astrocyte, microglia, oligodendrocytes, etc), and cell types genes were mainly expressed in were indicated in (**a**) and (**b**). Changes (standard deviations) of expressions in these genes were compared with the dosage-sensitive or loss-of-function-tolerant (LoFT) genes in human, macaque, and mouse brain (**c**). EX excitatory neuron, IN inhibitory neuron, OLI, DA dopaminergic neurons, non-DA non-dopaminergic neurons, OPC oligodendrocyte precursor cell, OLI oligodendrocyte, ENDO endothelial cells/pericytes, MIC microglia/macrophage; AST astrocyte, INT interneurons. ^*^P < 0.05, ^***^P < 0.001.

We next examined the enrichment of the three genes with the PD-associated SVs, CNVs, and STRs in brain cell classes, as well as whether these genes are sensitive to dosage changes. We performed a cell-type enrichment analysis using the human, macaque, and mouse brain single-cell transcriptomic data, which were published and released most recently^34–36^. In human neural cells, we found that these genes were significantly enriched in dopaminergic (DA) neurons (*P* < 0.05), but not in non-DA neurons, glial cells (astrocyte, microglia, oligodendrocytes, or oligodendrocyte precursor cells) or endothelial cells. These genes were significantly enriched in interneurons (*P* < 0.001) in mouse brain cells, and excitatory neurons (EX) (*P* < 0.05) in the macaque brain (Fig. 6a). Among these, *MUC19* was specifically expressed in DA neurons, *SLC2A13* was expressed in both DA and non-DA neurons, and *RFXP1* was expressed in endothelial cells and non-DA neurons (Fig. 6b). We also compared the changes (standard deviations, SDs) of expressions of these genes in the human single-cell transcriptomes with those of the dosage-sensitive or loss-of-function-tolerant (LoFT) genes (Supplementary Table 8). We revealed that the SDs of expression levels of all the genes in human DA neurons were lower than those of the LoFT genes with suggestive significance (*P* = 0.053), but not significantly different from the dosage-sensitive genes (*P* > 0.05). These changes were most significantly changed in excitatory neurons (EX) and interneurons (INT) of the macaque brain and INT of the mouse brain (*P* < 0.01, Fig. 6c). The results demonstrated that these PD-related genes were dosage-sensitive in the associated cell types.

## Discussions

The study of structure variations has provided enormous insight into the genetic architecture of many developmental traits and human diseases. We conducted a large discovery and replication study for identifying SVs, CNVs and STRs associated with PD using the high-depth WGS data. The identified variants localized in the introns and untranslated regions (UTRs) of three genes. By studying the burden of CNVs, we revealed that CNV deletions are specifically enriched in PD at the ends of chromosomes that may affect the function of subtelomeres. Moreover, we found and validated that a deletion near *MUC19* and an STR in the *SLC2A13* negatively modified the penetrance of the *LRRK2* G2385R variant. Further, the expressions of such genes were most enriched in the DA neurons and are sensitive to dosage changes. These findings provide unprecedented insights of genomic profiles of PD, which may explain the missing heritability of PD in many GWAS studies.

The contribution of genomic instability to ageing has been widely documented. López-Otin et al proposed the “hallmarks of ageing”, in which the primary hallmarks are genomic instability, telomere attrition, epigenetic alterations, and loss of proteostasis^37,38^. The higher burden of CNV deletions at the chromosomal end in PD, and its correlation with the normal ageing process revealed by our study may provide a genetic explanation for ageing as a risk factor for the disease. Nevertheless, as the age of the PD cases (61.3 ± 8.49) were significantly younger than the normal controls (71.9 ± 6.66 years), the CNV burden must include more factors than ageing that warrant further investigations. It has been suggested that telomere shortening is involved in the pathogenesis of PD^39,40^, but there were also studies that showed that people with longer telomeres might be vulnerable to neurodegenerative disorders^41,42^. Thus, telomeric lengths cannot be the only explanation for CNV burden in PD. These CNVs cover regions much larger than telomeres and may interfere with the regional structural instability related to aging and neurodegeneration^43^. However, assembling and detecting the structural variants are a huge challenge, since the current reference genome maps have missed over 70% of SVs^44,45^. This gap might be addressed by the most recent release of a complete human genome reference, telomere-to-telomere CHM13 (T2T-CHM13), accomplished by the Human Pangenome Reference Consortium (HPRC). The novel reference adds nearly 200 million base pairs of sequence and reveals much more CNVs than the GRCh38 references^46,47^. WGS short-read alignment in complex region with repeat motifs is another challenge. In the regions including telomeres, reads often align to more than one region and result in a zero-map quality. It is critical to generate a high candidate spectrum of CNVs according to the sequence complexity and CNV characteristics, including the fraction of zero-map quality reads and gaps. Long-read sequencing technology (e.g., Pacbio and nanopore platform) and long-read alignment algorithm (e.g., minimap2) generate a better alignment in complex regions for large-fragment variant calling^48^.

Short-tandem repeats are stretches of 2 to12­ bp ­long repeating tracts of DNA found in both coding and non­coding regions of the genome. In our study, known and de novo STRs have been identified in six novel genes, *SLC2A13, PPP3CA, ZNF609, RNPC3, GOLGA8A*, and *DAGLB*, but only the GGGAAA repeat in *SLC2A13* was associated with PD. Rare single nucleotide variants (SNVs) in *SLC2A13* have been associated with early-onset PD^49^. This observation, together with the increasingly reported novel STRs and SNVs, suggests that the PD genetic architecture is far beyond our knowledge. In particular, more than 1.5 million STRs have been identified in the human genome to date, and our knowledge of their native functions remains at the tip of a much larger iceberg^50^.

The GWAS for WGS data have significantly improved the identifications of novel disease-associated genetic signals, including SNVs, STRs, CNVs, and SVs. Although the *P* value threshold for SNVs in GWAS has usually been set at 5 × 10^−8^, this has not been set in the analysis for non-SNV variants. Although it’s reasonable to use a lower genome-wide significant threshold in these analyses, we still used this threshold to minimize the type 1 error, and it was not based on a mathematical calculation such as Bonferroni correction. Moreover, the decrease in sequencing costs and rapid emergence of long-read sequencing data and novel algorithms accelerate the identification of disease-associated non-SNV variants. Our analyses using the 133 samples with both short- and long-read sequencing data perfectly improved the validations for the large *MUC19* SV and the STR in *SLC2A13*, suggesting a promising potential for large-scale and accurate detection of these variants.

*MUC19* and *SLC2A13* are two genes located closely to *LRRK2*. Numerous studies have shown that both gene loci are associated with PD^26–33^. SNPs around the *MUC19* SV (rs145183052) and *SLC2A13* STR (rs1491924)showed a strong linkage with the SV and STR (rs145183052 and *MUC19* SV: D’ = 1, *r*^2^ = 0.01; rs1491924 and *SLC2A13* STR: D’ = 0.99, *r*^2^ = 0.02). Moreover, the rs11175593 variant in the *LRRK2*/*MUC19* locus has been associated with Crohn’s disease (CD)^51^, and an increase in *LRRK2* suppresses autophagy and enhances Dectin-1-induced immunity in a mouse model of colitis^52^. These data collectively suggest that *LRRK2* is associated with both PD and CD. Here we showed that the deletion near *MUC19* and GGGAAA repeats in *SLC2A13* significantly modify the penetrance of the G2385R variant. This result may partially explain the low penetrance of the variant and highlight the importance of genotyping the *MUC19* and *SLC2A13* variants in assessing the PD risk. Nevertheless, mechanisms on variant penetrance might be much more complicated than genetic variants, in which the effect of epigenetic modification, gene-environment, and protein–protein interactions may also have important roles.

The functional consequence of the disease-associated SVs remains elusive. Using the most recently published single-cell transcriptional data, we found that the genes nearby the PD-associated variants were expressed specifically in different neural cells, and are sensitive to dosage changes. Moreover, these genes were linked to molecular events leading to neurodegeneration. *RXFP1*, the relaxin-like family peptide receptor 1, has been established to mediate anti-apoptotic, angiogenic, and chemo-resistant functions in cancer cells^53^. Enriched expression was also demonstrated in brain cells covering the olfactory, cortical, midbrain, and pontine circuits^54^ and it was reduced in parietal cortex of brain of non-depressed Alzheimer’s disease (AD) patients^55^. *SLC2A13* was a novel γ-secretase associated protein (GSAP) that regulates Aβ production without affecting Notch cleavage^56^. These findings collectively suggest that the variants may contribute to neurodegeneration via regulation of the key molecular events of neurodegeneration.

In summary, our study adopted a reliable analytic pipeline to conduct a large-scale analysis of SVs, CNVs, and STRs using high-depth WGS data. We identified novel variants that encompass three genes associated with PD. These findings provide new insights into the genetic architecture of PD, which may be useful for further understanding the etiology, mechanisms, and pathogenesis of the disease.

## Methods

### The participants and design of the study cohort

The study consisted of the discovery and replication cohorts. The discovery cohort included 466 PD cases and 513 controls. The replication study included 352 cases and 547 controls. PD patients and healthy elderly controls were enrolled between January 2016 and May 2021 from the cohort of the Chinese National Consortium on Neurodegenerative Diseases (CNCPD), established by the Chinese Parkinson Study Group (CPSG). The cases were diagnosed by movement disorders specialists using MDS clinical diagnostic criteria for Parkinson's disease^57^, and those with a family history in a first- or second-degree relative were excluded. Control subjects were selected from the community cohorts of the Beijing Longitudinal Study on Aging II (BLSA II) and were sex- and age-matched with PD cases. The BLSA II cohort was launched in 2009 in Beijing, which included 10,039 healthy elderlies aged 55 and older (70.51 ± 7.82) years at baseline recruitment and followed up annually for the development and progression of age-related disorders^58,59^. The clinical features of the two cohorts were outlined in Supplementary Table 1. Whole-genome sequencing was performed and a genome-wide association study was conducted for discovering the PD-associated variants. The replication study was performed to validate the variants identified in the GWAS. For further validation of the *MUC19* SV and *SLC2A13* STR, alignment analyses were performed using the most recently published long-read and short-read sequencing data. Among these individuals, cases, and controls carrying the *LRRK2* G2385R variant were used for studies of the interaction between the variant and the identified PD-associated SVs and STRs (Fig. 1).

### High-depth whole-genome sequencing and bioinformatic analysis

Genomic DNA was extracted from peripheral blood by standard protocols. High-depth WGS was performed on the BGI-seq 500 platform (BGI, Shenzhen, China). An average of 44.3× coverage depth reads (100 bases paired-end) was generated. Adapters of reads, reads with uncertain bases (N) >10% or low-quality bases (Phred <5) >50% were truncated by Fastp^60^. High-quality reads were aligned to the human genome reference sequence (GRCh38/hg38) by BWA (0.7.15)^61^. Duplicated reads were marked by samblaster^62^. The results of aligned reads were stored in cram format by samtools^63^. Structural variations (SVs), copy-number variations (CNVs), and short-tandem repeats (STRs) were detected by public software, including LUMPY^64^, SVTyper^65^, cnvpytor^66^, ExpansionHunter^67^, and Expansion HunterDenovo^67^. These variants were annotated by annotSV software and exomiser database^68,69^. Deletion SV in *MUC19* was visualized by samplot^70^.

### Structure variation (SV) calling

SVs calling was performed by LUMPY^64^ (v.0.2.13) with a bed file (https://github.com/hall-lab/speedseq/blob/master/annotations/exclude.Cnvnator_100bp.GRCh38.20170403.bed) for excluding calling. SV genotyping was performed by SVTyper (v.0.1.4). SVs of all samples were sorted into a single call set according to physical coordinates and SV-type. Neighboring SV coordinates in the single call set were merged according to the variant probability curve by Svtools merge (v.0.3.2). The SVs in the single call set were regenotype by SVTyper in each sample. The copy numbers of SVs were calculated by CNVpytor at SV coordinates. Subsequently, the per-sample VCFs were combined using svtools-vcfpaste. The cross-sample SVs were annotated with allele frequency by svtools-afreq. Identical SVs were pruned by svtools prune. Finally, SVs were reclassified by svtools-classify at large sample model. A part of duplication and deletion SVs were reclassified into breaking and/or MEI according to the copy number and repeat mask in the hg38 genome.

### SV filtering

We employed the strategy for cross-sample SV filtering according to Abel’s study^24^. For duplications, the variants were re-genotyped with more sensitive parameters to better reflect the expected allele balance for simple tandem duplications (https://github.com/ernfrid/regenotype/blob/master/resvtyper.py). For MEI and DEL, their genotypes were set to missing on a per-sample basis if: the length of the DEL and MEI was smaller than the minimum insert size which was estimated by SVTyper (https://github.com/hall-lab/svtools/blob/develop/scripts/del_pe_resolution.py), neither the site was poorly captured by split reads (https://github.com/hall-lab/svtools/ blob/develop/scripts/filter_del.py). DEL and MEI genotypes were refined based on the clustering of allele balance and copy-number values (https://github.com/hall-lab/svtools/blob/develop/scripts/geno_refine_12.py). For INV, variant passed if: mean sample quality ≥150, or split-read and paired-end evidence made up more than 10% of total evidence. Variants with a size <100 bp were excluded. Cross-sample SVs with sample genotyped rate <90% or MAF <1% were excluded for subsequent association study.

### SV identifying

To identify the identical SV in Abel’s study, we accessed the coordinates of our SV in Abel’s study and extracted all the SVs with 1 bp overlap via tabix tools^71^. For each overlapped SV in Abel’s study, the overlap proportion was calculated by the formula (1):

1 $${P}_{i}=\frac{{\left(\min \left\{{{End}}_{0},{{End}}_{i}\right\}-\max \left\{{{Start}}_{0},{{Start}}_{i}\right\}\right)}^{2}}{\left({{End}}_{0}-{{Start}}_{0}\right)\times \left({{End}}_{i}-{{Start}}_{i}\right)}$$

$$i\in \left\{1,2,\ldots ,n\right\}$$

in which Start and End refer to the physical start and end position of SV. The best-fitted SV was identified by the formula (2):

2 $$\mathop{\max }\limits_{i\in \{1,2,\ldots ,n\}}\{{P}_{i}\}$$

The best-fitted SV with overlap proportion at a threshold of >0.8 was examined as a highly identical SV.

### CNV calling

CNV calling was performed by CNVpytor. The bin size was set to 100 bp according to the coverage depth suggested by the author. CNVs with *P* value <0.0001, a fraction of MQ0 reads <0.5, a fraction of *N* base <0.1, and distance from large gap segment >1000 bp were identified as high candidates. CNVs with size <1000 bp or >100,000 bp were excluded from subsequent studies. CNVs coordinates of all samples with 1 bp overlap were merged together by bedtools^72^. A matrix marked the new cross-sample CNV coordinates and CNV size for each sample was generated for subsequent burden analysis.

### STR calling

Known STR (kSTR) and de novo STR (dSTR) calling were performed by ExpansionHunter and ExpansionHunterDenovo, respectively. A known STR catalog (https://github.com/Illumina/RepeatCatalogs) that contains 170,461 STR loci was used for kSTR genotyping in each sample. kSTRs with quality >30 and coverage depth >10× were considered high-candidate and retained. dSTRs were predicted at the genome-wide level with MAPQ of anchor reads >50 and MAPQ of in-repeat reads <40. dSTRs with coverage depth of observed reads and normalized reads both >4× were considered high-candidate and retained. Per-sample kSTR genotypes of all samples were merged together by bcftools^63^. Per-sample dSTR coordinates were merged together by ExpansionHunterDenovo merge.

### Replication of the association between PD and the GWAS-identified SV, CNVs and STRs

We designed strategies for replication of the SV, CNVs, and STRs in the GWAS study. For the 1.6 kb-deletion neighboring the *MUC19* gene, we first designed a primer (F: GAGACAACTAGACTTTCA; R: CCGGCTGATACTGTAGTT) to amplify a 2.2 kb DNA segment across the SV coordinate (62–52 touchdown, 2 min, 35 cycles). A 500–600 bp DNA segment was amplified in participants with the WGS-identified deletion and sequenced by the ABI 3730XL platform.

For verification of the kSTRs and dSTRs, primers were designed for amplifying the short-repeat kSTRs in the *PPP3CA, SLC2A13*, and *ZNF609* genes (Supplementary Table 5). The PCR thermocycling program was: 94 °C for 5 min；28 cycles of 94 °C for 30 s, 58 °C for 30 s, 72 °C for 1 min, followed by the final extension at 72 °C for 10 min. The PCR products were purified and sequenced on the 3730X1 Sequencer (Applied Biosystems, USA) using the sequencing primers. The DNA sequencing analysis was performed, and STRs were analyzed with the Sequencing Analysis 5.2.0 and Sequencher 5.1 package (Applied Biosystems™, USA). Three primers for each gene were designed and fluorescently labeled to amplify the long-repeat dSTR markers in the *RNPC3, GOLGA8A*, and *DAGLB* genes (Supplementary Table 6). The PCR products were amplified in the thermocycling program: 94 °C for 5 min, 35 cycles of 94 °C for 30 s, 55 °C for 30 s, 72 °C for 20 s, and 72 °C extension for 10 min. The PCR products were run and the STRs were detected by capillary electrophoresis in the 3730XL sequencer (Applied Biosystems™, USA).

CNVs were validated using the quantitative real-time PCR (qPCR) using the qPCR sequencer Gentier 96R (Tianlong, China) and FQD-96A (Bori, China). Three primers (the forward, intermediate, and reverse) were designed specifically to sequences inside and flanking the CNVs surrounding the 4 regions (2q14.1, 2q14.3, 3p24.1, and 4q32.1) (Supplementary Table 7). The amplification was normalized to the ALB reference gene, whose primers were also designed. For each qPCR reaction, 10 ul of SYBR qPCR Master Mix, 1 ul of forward and reverse primers (10p), 2 ul of DNA template, and 6 ul of ddH_2_O were used. The amplification was performed in the qPCR system (Gentier 96R, Tianlong Inc, China) using the standard thermocycling program: 95 °C for 3 min, 40 cycles of 95 °C for 15 s, 60 °C for 20 s followed by a melting curve (95 °C for 20 min). Each sample was assayed in triplicate. Relative changes in genomic sequence abundance were calculated using the 2−ddCT method, and error was calculated using standard propagation of errors. Data were visualized using the Medtl_ System_ V1(Tianlong Inc, China) and Gene-9660Setup v1. 0. 13rc (Bori Inc, China). The amplification curve was analyzed using the absolute fluorescence quantification method.

### Further validation of the *MUC19* deletion SV and the *SLC2A13* STR

The *MUC19* deletion was further validated by a two-step PCR and Sanger sequencing strategy using 281 samples tested in the WGS study and with high-quality DNA. In addition, sequences from other published long-read and short-read data were analyzed. In the two-step Sanger sequencing study, the first PCR primer (primer 1) was described above to amplify the 1.6-kb *MUC19* SV, which can amplify the fragment spanning the breakpoint in samples with the deletion, but cannot span the region in wild-type samples. In step 2, for differentiating the heterozygous from homozygous deletion, an additional primer (primer 2; F: 5′-GGCTATCAGCTGGAGTGATG-3′; R: 5′-GCTGATACTGTGGTTGTCCC-3′) was designed for amplifying a small fragment within the 1.6-kb fragment. Sequence was extracted from the ab1 files by Tracy software and aligned by ngmlr software. The sequences amplified by primer 2 were aligned to the sequence within the 1.6-kb fragment. The genotypes of the 281 samples detected by Sanger sequencing were used as a “truth” set. The genotype accuracy of short-read WGS at *MUC19* SV was calculated by the proportion of identical genotypes predicted by the WGS and Sanger sequencing data, using the formula (3):

3 $$\frac{{N}_{1}\left({G}_{s}={0|0},{G}_{{sa}}={0|0}\right)+{N}_{2}\left({G}_{s}={0|1},{G}_{{sa}}={0|1}\right)+{N}_{3}\left({G}_{s}={1|1},{G}_{{sa}}={1|1}\right)}{N}$$

where ${G}_{s}$ refers to the genotype of short-read WGS, ${G}_{{sa}}$ refers to the genotype of sanger sequencing, and $N$ refers to the total number of samples in validation ($N=281$).

To analyze the published long-read and short-read data, we performed alignments of the sequences from 133 samples with both short-read (WGS) and long-read PacBio and/or Ont platforms derived from most recently published studies (Supplementary Table 4) to the *MUC19* deletion coordinate via the IGV tool. Short-read sequences were aligned by BWA and the *MUC19* SV were genotyped by Svtyper, as described above. Long-read sequences were aligned by ngmlr with default parameter pre-set for Pacbio/Ont and *MUC19* SV were genotyped by sniffles with default parameters. We used the genotype of SV detected by long-read data as an additional “truth” set. The accuracies for the WGS-predicted genotypes were calculated by the formula (4):

4 $$\frac{{N}_{1}\left({G}_{s}={0|0},{G}_{l}={0|0}\right)+{N}_{2}\left({G}_{s}={0|1},{G}_{l}={0|1}\right)+{N}_{3}\left({G}_{s}={1|1},{G}_{l}={1|1}\right)}{N}$$

where ${G}_{s}$ refers to the genotype of short-read WGS, ${G}_{l}$ refers to the genotype of long-read WGS, and $N$ refers to the total number of samples ($N=133$).

The *SLC2A13* GGGAAA repeat size was confirmed by HipSTR method for samples of the WGS-identified STR by the ExpansionHunter software. In addition, the sizes were determined by a repeat-primed PCR and re-analysis of the published long-read and short-read sequences. The long-read sequencing data from the 133 above-mentioned samples were analyzed and the repeat sizes were determined using the NanoRepeat software with a default parameter set for Pacbio/Ont. As the NanoRepeat did not tell the genotype, we determined the genotypes by the max likelihoods for the three genotypes (GT = 55, 56, and 66), using the formulas (5–7):

5 $${Likelihood}\left(55|k\right)=P\left(k|{GT}=55\right)=\left({n}\atop{k}\right){(1-\varepsilon )}^{n-k}{\varepsilon }^{k}$$

6 $${Likelihood}\left(56|k\right)=P\left(k|{GT}=56\right)=\left({n}\atop{k}\right){\left(\frac{1}{2}\right)}^{n}$$

7 $${Likelihood}\left(66|k\right)=P\left(k|{GT}=66\right)=\left({n}\atop{k}\right){(1-\varepsilon )}^{k}{\varepsilon }^{n-k}$$

Where $k$ refers to the number of reads supported 6 repeats, $n$ refers to the total number of reads supported 5 and 6 repeats, $\varepsilon$ refers to the sequencing error that is simply set at 0.05.

### SNP calling and linkage distribution analysis

Per-sample SNVs were detected and combined into a cross-sample by GATK. Variants with quality <100, genotyped rate <0.95, *P* value of HWE test <0.0001, or MAF <0.05 were excluded for subsequent linkage distribution analysis. SNPs/SV/STR at *SCL2A13*-*LRRK2-MUC19* loci were used for linkage distribution calculation. Haplotypes were phased by Beagle. The *r*^2^ of pairwise variants was calculated by vcftools^73^.

### Principal component analysis

Principal component analysis (PCA) was performed by prcomp function in R-program. The cross-sample genotype matrix of SV, size matrix of CNV, and repeat number matrix of dSTR and kSTR were used for PCA independently. The mutated allele frequency (MuAF) thresholds of SVs, CNVs, kSTRs, and dSTRs were set to 0.01–0.99, 0.01–1, 0.05–1, and 0.01–1. Specially, MuAF of cross-sample CNV, dSTR, and kSTR were respectively defined by the formulas (8–10):

8 $${CNV\; M}u{AF}=1-\frac{{\sum }_{i=0}^{n}{C}_{i}}{n},{c}_{i}\left\{\begin{array}{ll}=0,{\rm{any}}\; {\rm{size}}\; {\rm{CNV}}\; {\rm{at}}\,i{\rm{t}}{\rm{h}}\,{\rm{sample}}\\ =1,{\rm{no}}\; {\rm{CNV}}\; {\rm{at}}\,i{\rm{t}}{\rm{h}}\,{\rm{sample}}\end{array}\right.$$

9 $${dSTR\; M}u{AF}=1-\frac{{\sum }_{i=0}^{n}{C}_{i}}{n},{c}_{i}\left\{\begin{array}{ll}=0,{\rm{with}}\; {\rm{support}}\; {\rm{reads}}\; {\rm{at}}\,i{\rm{t}}{\rm{h}}\,{\rm{sample}}\\ =1,{\rm{zero}}\; {\rm{support}}\; {\rm{reads}}\; {\rm{at}}\,i{\rm{t}}{\rm{h}}\,{\rm{sample}}\end{array}\right.$$

10 $${kSTR\; M}u{AF}=1-\frac{{\sum }_{i=0}^{n}{C}_{i}}{n},{c}_{i}\left\{\begin{array}{ll}=0,{\rm{altered}}\; {\rm{repeat}}\; {\rm{number}}\; {\rm{at}}\,i{\rm{t}}{\rm{h}}\,{\rm{sample}}\\ =1,{\rm{reference}}\; {\rm{repeat}}\; {\rm{number}}\; {\rm{at}}\,i{\rm{t}}{\rm{h}}\,{\rm{sample}}\end{array}\right.$$

### Cell type enrichment

To indicate what cell types the PD-related genes are specifically expressed in, we perform a cell type enrichment via EWCE R-package. The method first calculated the specificity of a gene in each cell type and then estimated a background distribution of gene specificity by a bootstrap method at 10,000 times resampling. *P* values of genes were calculated by the cumulative density function of the background distribution. We employed three single-cell transcriptome datasets of recent studies from Kamath’s, Lei’s, and Skene’s studies^34–36^. Kamath’ study sampled a total of 387,483 nuclei from midbrain of ten patients with PD and eight controls and identified seven cell types, including dopaminergic neurons (DA), non-dopaminergic neurons (non-DA), oligodendrocyte precursor cell (OPC), oligodendrocyte (OLI), endothelial cells/pericytes (ENDO), microglia/ macrophage (MIC), and astrocyte (AST). Lei’s study generated 358,237 cells from the prefrontal cortex, primary motor cortex, and primary visual cortex of adult female cynomolgus monkey brain and identified seven cell types, including excitatory neuron (EX), inhibitory neuron (IN), OLI, OPC, AST, MIC, and ENDO. Skene’s study collected various single-cell transcriptome datasets of mice brains and identified 24 brain cell types including interneurons (INT), DA, OLI, OPC, AST, MIC, and ENDO. The three datasets were released by the authors and used for cell-type enrichment analysis in our study.

### Dosage sensitivity analysis

To indicate whether the gene with SVs are dosage-sensitive, we calculated the standard deviation (SD) of the fold-change of gene expression in single-cell transcriptomes of mouse brains and then compared the SD of PD-related genes with known dosage-sensitive genes and loss-of-function tolerating (LofT) genes via our previous method^74^. For known dosage-sensitive genes, we employed 299 dosage-sensitive genes predicted by Dang et al.^75^ and retained 49 genes related to neurodegenerative diseases and/or neurodevelopmental diseases, including Alzheimer’s disease, Parkinson’s disease, Huntington's disease, spinocerebellar ataxia, multiple system atrophy, epilepsy, autism spectrum disorder, and schizophrenia. For LofT genes, we employed 330 genes carried high frequency putative homozygous loss of function variant from Lek et al.^76^. All these genes for analysis were listed in Supplementary Table 8. Dosage-sensitive genes are known to be intolerant of expression alteration and are supposed to present low SD values. Oppose to dosage-sensitive genes, LofT genes are known to be tolerant for expression alteration and supposed to present high SD values. SD was calculated by the formula (11):

11 $${\rm{SD}}=\sqrt{\frac{\mathop{\sum }\nolimits_{i}^{n}{({C}_{i}-\bar{C})}^{2}}{n-1}}$$

in which $C$ refers to fold-change, $i$ refers to the *i*th cell, and $n$ refers to the total number of cells^76^. The fold-change was calculated by the expression in one cell divided by the mean expression in all cells, as the formula (12):

12 $${C}_{i}={E}_{i}/\bar{E}$$

in which $E$ refers to the expression in one cell, $\bar{E}$ refers to the mean expression, $i$ refers to the *i*th cell. One-side Mann–Whitney test was used to indicate the significance of low SD values of PD-related genes.

### Statistical analysis

In CNV burden analysis, we classified per-sample CNVs into each 5 Mb region of chromosomes via the physical position of the CNV midpoint. One-side Mann–Whitney test was used to compare the CNV counts between PD patients and controls in each 5 Mb region. For chromosome terminal burden analysis, we calculated the average of CNV counts for the terminal 5 Mb region from 22 autosomes, as well as the 5–10 Mb, 10–15 Mb…..., 45–50 Mb regions. One-side Mann–Whitney test was used to compare the CNV counts between PD patients and controls in each 5 Mb region in the terminal 50 Mb of chromosome region.

In GWAS, variants with MAF >0.01 were used for the association test. Logistics regression was used to compare the allele frequencies of SVs and the sizes of CNV, and the one-side Mann–Whitney test was used to compare the repeat numbers of STRs between PD patients and controls. In logistics regression analysis, gender, age, and the top two principal components of PCA result were used as covariates. An association test was performed on Northern participants and Southern participants, respectively to avoid the genetic bias of geography. Statistical summaries were used for meta-analysis. *P* values of variants were transformed into Z-scores by the formula (13):

13 $${Z}_{i}={\phi }^{-1}\left({P}_{i}/2\right)\times {sign}({\Delta }_{i})$$

in which ${P}_{i}$ refer to the *P* value of the variant in the *i*th dataset, ${\Delta }_{i}$ refers to the direction of effect of the variant in the *i*th dataset. The weight of *P* value was calculated by the formula (14):

14 $${W}_{i}=\sqrt{{N}_{i}}$$

in which ${N}_{i}$ refers to the sample size of the *i*th dataset. Meta *P* value was calculated by the formula (15):

15 $$P=2\phi \left(\left|-\frac{{\sum }_{i}{Z}_{i}{W}_{i}}{\sqrt{{\sum }_{i}{w}_{i}^{2}}}\right|\right)$$

The genome-wide significant threshold, *P* < 0.05/1,000,000, was used to examine significant association in meta-analysis. In the replication analysis of the 11 variants, *P* < 0.05/11 = 0.0045 was used to examine significant association. In interaction analysis, Logistics regression was used to compare the allele frequencies of SV and STR between controls and PD patients with or without the *LRRK2* G2385R variant, respectively. All the statistical analysis was performed by in-house R-script.

### Power analysis

CaTS tool (https://csg.sph.umich.edu/abecasis/CaTS/) is used to calculate the statistical power for SVs at a threshold of type I error at 0.05/100,000 against different allele frequencies. Power analysis showed that our study design has the highest power at the allele frequency of 0.45 and power >0.4 at the allele frequency spectrum of 0.3–0.6. MKpower R-package (https://github.com/stamats/MKpower) is used to calculate the statistical power for CNV and STR at a threshold of type I error at 0.05/100000 against size differences. The result showed 50% power when cases harbor 0.325 standard deviations of mean size higher than that of controls.

### Analytic software packages

Analyses were performed using the following public software packages: (i) Short-read alignment (BWA, https://github.com/lh3/bwa; samblaster, https://github.com/GregoryFaust/samblaster; sambamba, https://github.com/biod/sambamba; Samtools, https://github.com/samtools/samtools); (ii) Variant calling of SV (LUMPY, https://github.com/arq5x/lumpy-sv; SVTyper, https://github.com/hall-lab/svtyper), repeat-expansion (ExpansionHunter, https://github.com/Illumina/ExpansionHunter; Expansion Hunter Denovo, https://github.com/Illumina/ExpansionHunterDenovo), CNV (CNVpytor, https://github.com/abyzovlab/CNVpytor), and SNV (GATK4, https://gatk.Broadinst-itute.org); (iii) Long-read alignment (ngmlr, https://github.com/philres/ngmlr) and genotype of MUC19 SV (sniffles, https://github.com/fritzsedlazeck/Sniffles), SLC2A13 GGGAAA repeat (NanoRepeat, https://github.com/WGLab/NanoRepeat); (iv) Variant filter, merge, and variant annotation (vawk, https://github.com/cc2qe/vawk; bedtools, https://github.com/arq5x/bedtools2; bcftools, https://github.com/samtools/bcftools; tabix, https://github.com/tabixio/tabix; svtools,https://github.com/hall-lab/svtools; AnnotSV, https://github.com/lgmgeo/AnnotSV); (v) Association test and data visualization (R, https://www.r-project.org; Samplot, https://github.com/ryanlayer/sam-plot); (vi) Genotype phasing and linkage disequilibrium calculation (Beagle, http://faculty.washington.edu/browning/beagle/beagle.html; vcftools, https://github.com/vcftools/vcftools); (vii) Cell-type expression enrichment analysis (https://github.com/NathanSkene/EWCE); (viii) Power analysis (CaTS tool, https://csg.sph.umich.edu/abecasis/CaTS/; MKpower R-package, https://github.com/stamats/MKpower).

### Reporting Summary

Further information on research design is available in the Nature Portfolio Reporting Summary linked to this article.

### Supplementary information

Supplementary materials

Reporting summary checklist

Data set 1

## Data Availability

The original DNA sequencing data supporting the conclusions of this article will be available in the China National Center for Bioinformation/Beijing Institute of Genomics, Chinese Academy of Sciences (GSA-Human: HRA005774) that are publicly accessible at https://ngdc.cncb.ac.cn/gsa-human. Public single-cell transcriptome data have been deposited by the authors under accession code/website (Human brain: GSE178265; Macaque brain: https://db.cngb.org/mba/download; Mouse brain: GSE60361). GWAS statistical summary of SVs/CNVs/STRs were released at GitHub (https://github.com/GWASummary/PD-GWAS-SV). All other data are available for researchers who meet the criteria for access to these data from the corresponding author.

## Code availability

The bioinformatic pipeline is written in shell. The code of statistical analysis is written in R-program. Pipeline and necessary statistical code were released at GitHub (https://github.com/liuhankui/PD).
