# Supplementary material for: High-depth whole-genome sequencing identifies structure variants, copy number variants and short tandem repeats associated with Parkinson’s disease
Source: NPJ Parkinsons Dis. 2024 Jul 23;10:134. doi: 10.1038/s41531-024-00722-1 (PMC11266557; doi:10.1038/s41531-024-00722-1)
Supplement: Supplementary file 1 — Supplementary materials [file 41531_2024_722_MOESM1_ESM.pdf]

SUPPLEMENTARY MATERIALS

Supplementary Figure 1. The whole-genome sequencing data. (a) The distribution of coverage and sequencing depth**; (b)** Variant distribution. The 1-4 circles refer to the scaled density of SV, CNV, kSTR and dSTR.

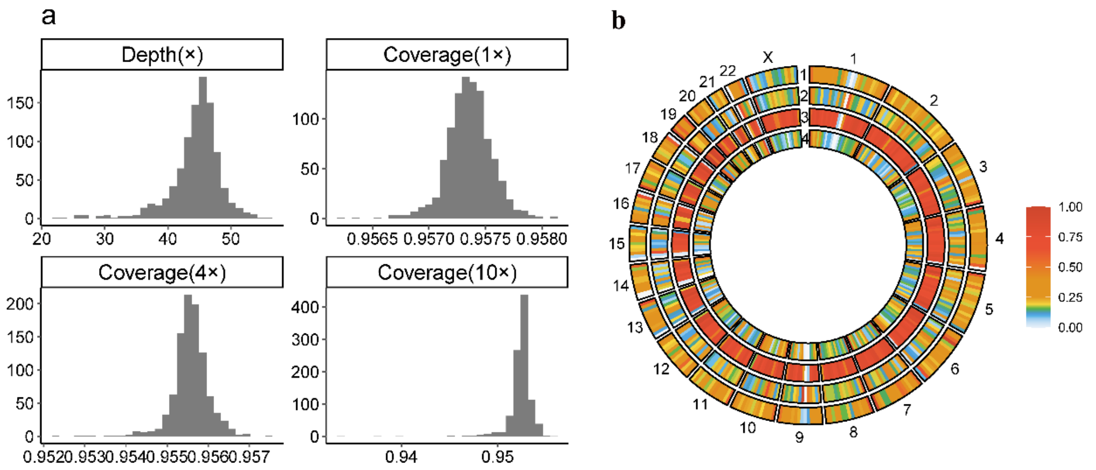

Supplementary Figure 2. Comparison of the SV characteristics of the current study with the Abel's study. (**a**) Per-sample SV counts. Number of DEL, DUP, INV and variants with different minor allele frequency (MAF; common, low frequency, rare and ultra rare) were displayed; (**b**) The sizes of the SVs with different MAFs; (**c**) The counts and identity of the overlapping SVs identified in our study with those in the Abel's study; (**d**) The count of SVs with different identities (high, low and novel) between the two studies; (**e**) Correlation between the SV sizes and MAFs (**f**) of high identity variants between the two studies. DEL: deletion, DUP: duplication; INV: inversion; MEI: mobile-element insertion.

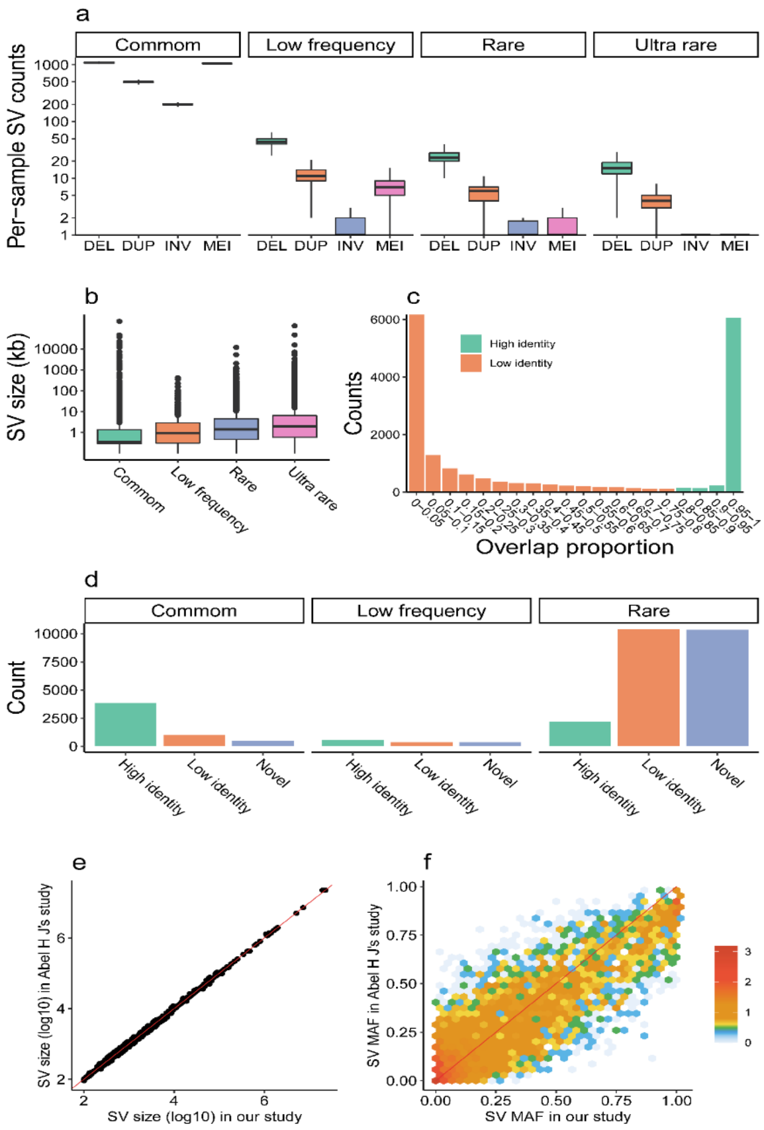

Supplementary Figure 3. Genetic background of individuals with different phenotypic, demographic and sequencing data. Principal component analysis (PCA) was performed for SVs, CNVs and STRs between different groups: PD/Control; North/South; Male/female; <68/≥68 years old; <45×/≥45× read-depths. (**a**) including autosomes and sex chromosomes; (**b**) including only the autosomes.

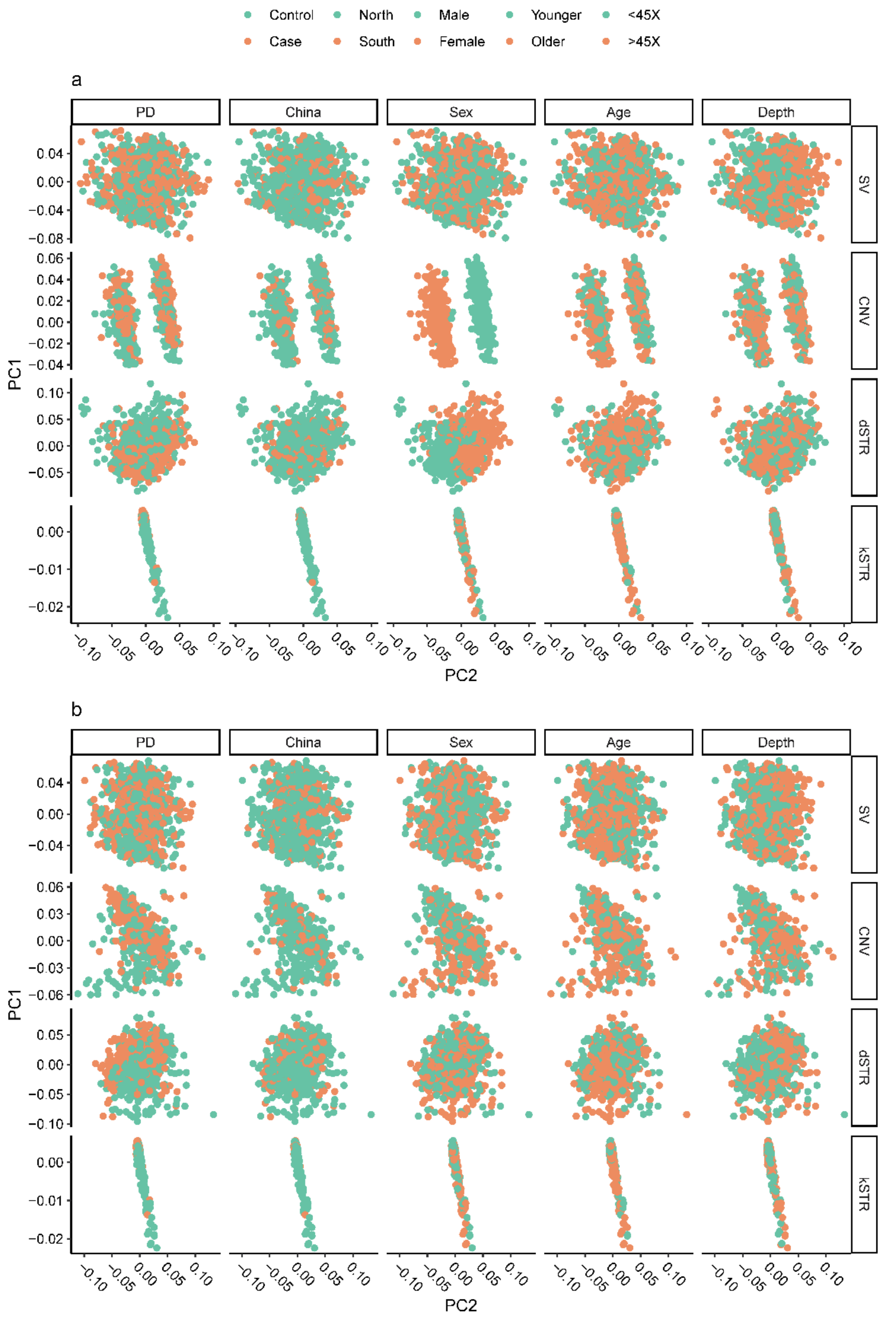

Supplementary Figure 4. The expression of the structural variants (SVs) in PD cases and controls at the terminal 50 Mb chromosome regions (shown in 5 Mb-window). CNV: copy number variant; SV: structural variant; DEL: deletion; DUP: duplication; INV: inversion; MEI: mobile-element insertion.

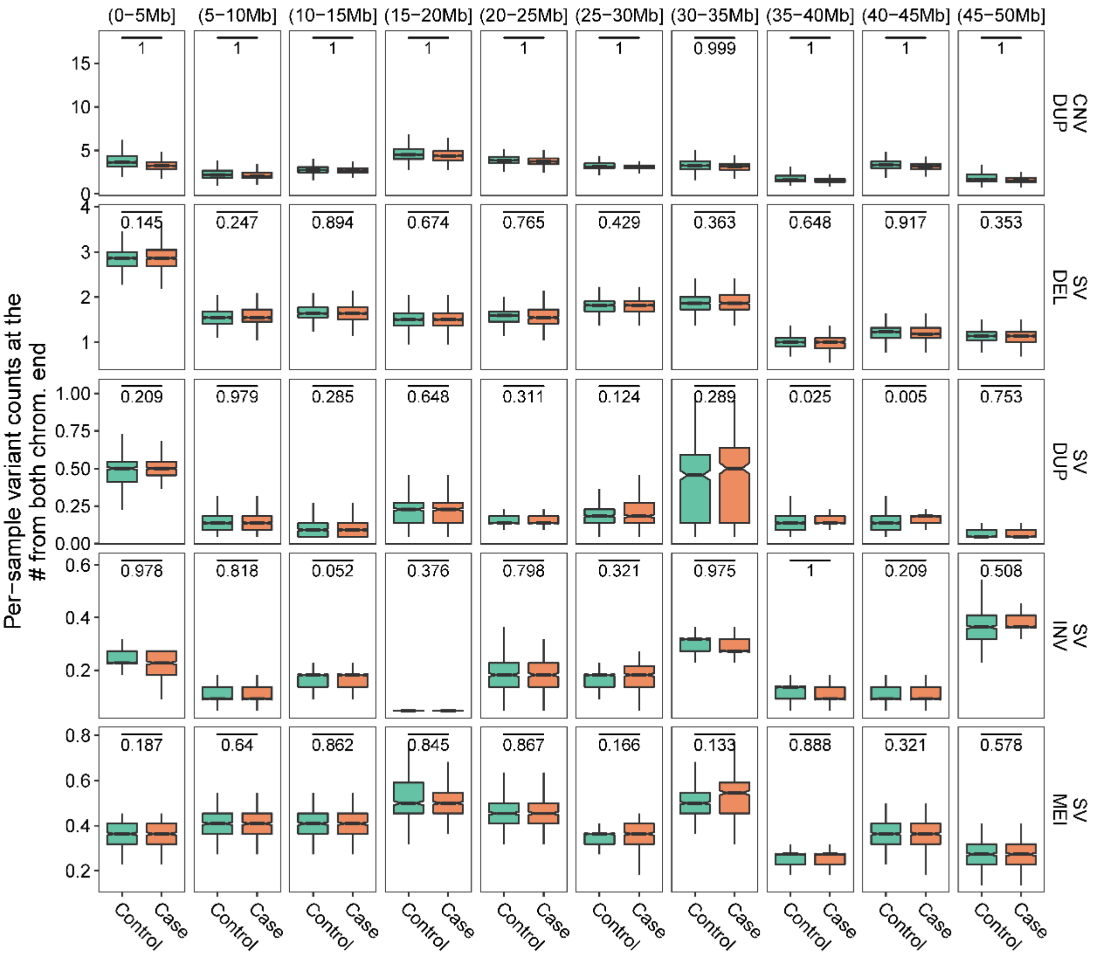

Supplementary Figure 5. Physical mapping of *LRRK2*, *SLC2A13, MUC19* gene and the variants surrounding them. The loci of the genes and variants were displayed. r^2 values indicate the linkage disequilibrium between the variants.

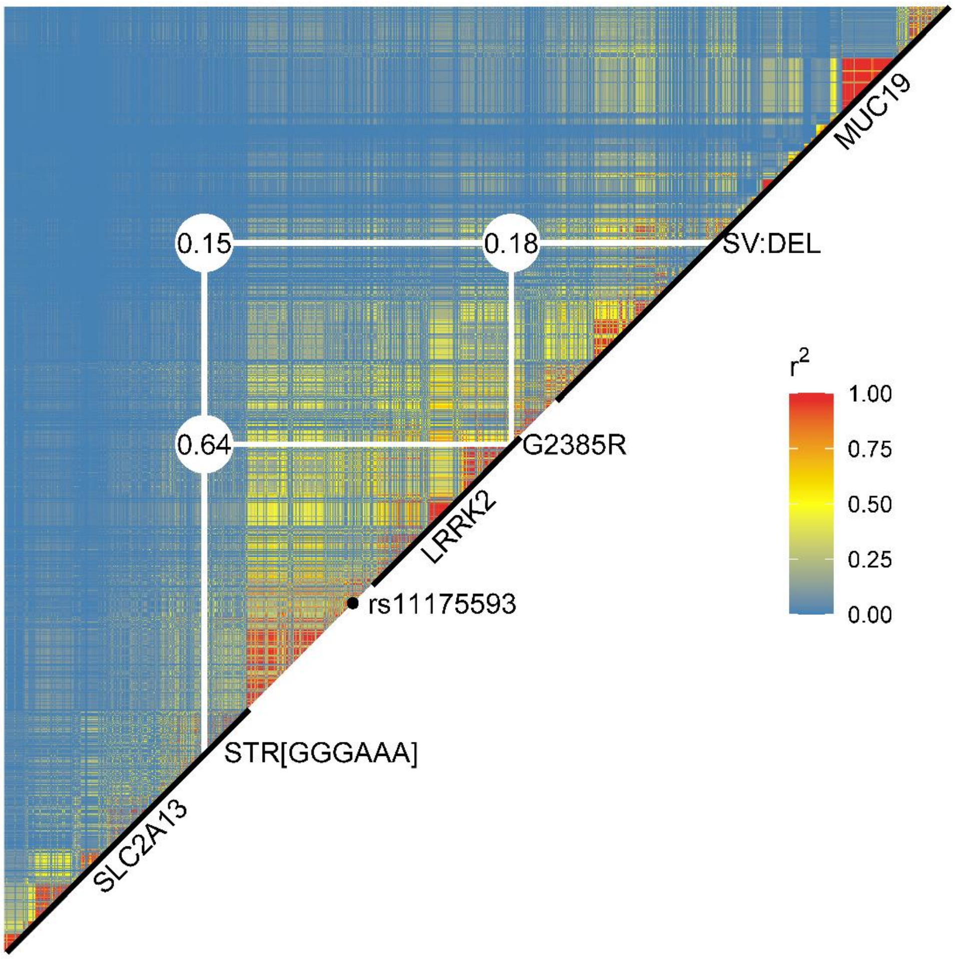

Supplementary Figure 6. The distribution of repeat numbers of the known STRs associated with PD. Repeat numbers of all known genes associated with PD were displayed and compared between cases and controls.

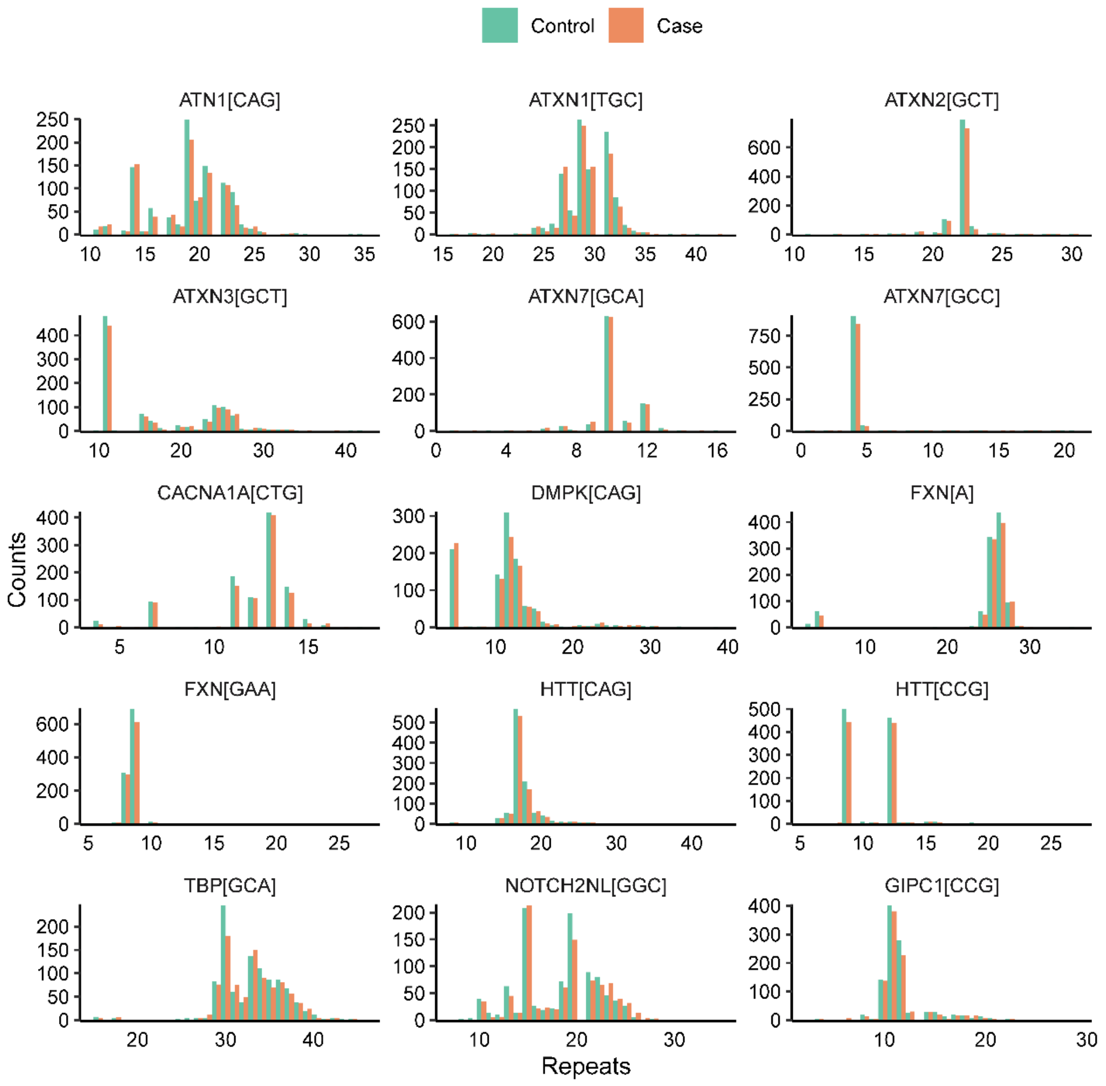

Supplementary Figure 7. Sanger sequencing-validated deletion (SV) neighboring *MUC19*. A PCR primer was designed to amplify a 2.2 kb DNA segment across the 1.6kb *MUC19* SV fragment. (**a**) After PCR with primer 1, the amplified fragment was viewed in the Agarose gel. A 500bp fragment was detected in samples with the WGS-detected heterozygous or homozygous deletion, while it was invisible in the wild-type samples. **(b)** Sequencing of the PCR product for primer 1 showed that the deleted fragment was split at the 246th bp (left:11-246, right:247-505) and aligned to the breakpoint of the SV (left: 40487925-40488161, right: 40489842-40490102) by blast, indicating a 1.6 kb (40488161-40489842) deletion. The location (chr12:40488161-40489842) is consistent with the location (chr12: 40488206-40489818) of the deletion detected by WGS reads. (**c**) The amplified PCR product with primer 2 for differentiation of the heterozygous from the homozygous deletion. (**d**) Alignment of sequences of the amplified fragment with those in the 1.6kb *MUC19* deletion. The sequence of the heterozygous deletion can, while the homozygotes cannot, be aligned to the sequence.

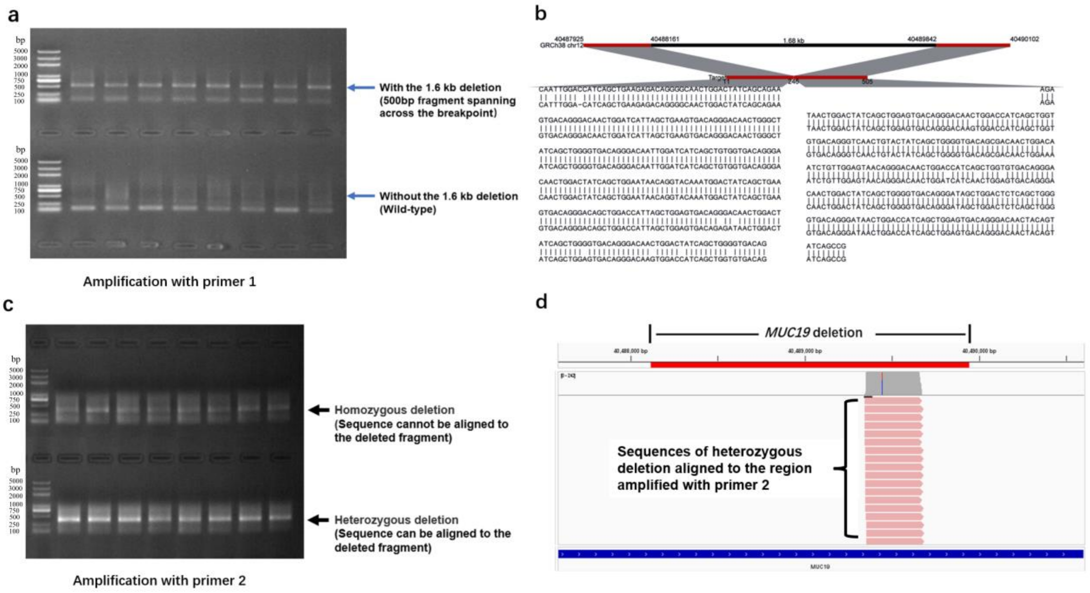

Supplementary Figure 8. Verification of *SLC2A13* STR by repeat-primed PCR. The fragment was amplified by the primers. The waves of the fluorescence indicated the repeat size and the genotypes (GTs) was determine by the positions of the waves.

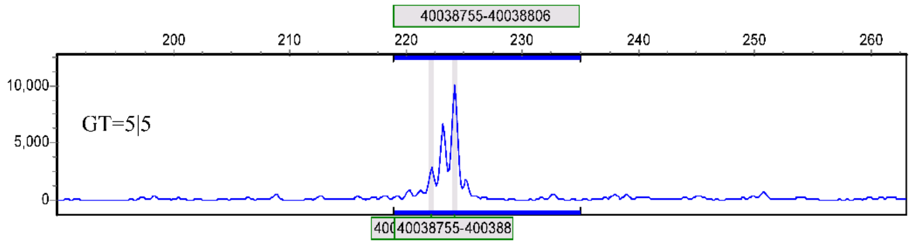

No	Dye	Size	Height	Area	Marker	Allele	Difference	Quality	Score	Allele Comments
1	Blue	222.2	2848	17832	40038755-40038806	40038755-400388		4.8	Pass	43.7
2	Blue	224.2	10068	64461	40038755-40038806	40038755-400388		2.8	Pass	339.6

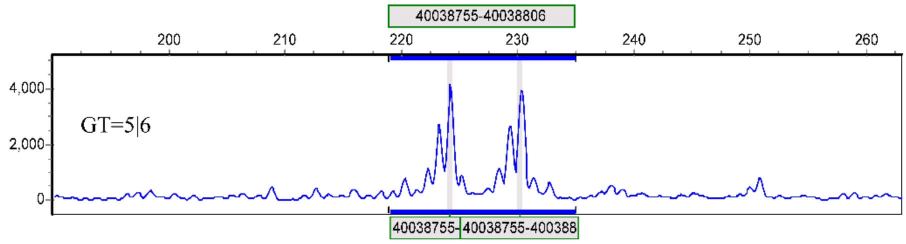

No	Dye	Size	Height	Area	Marker	Allele	Difference	Quality	Score	Allele Comments
1	Blue	224.2	4149	26228	40038755-40038806	40038755-400388		2.8	Pass	267.7
2	Blue	230.3	3962	25155	40038755-40038806	40038755-400388		3.3	Pass	251.0

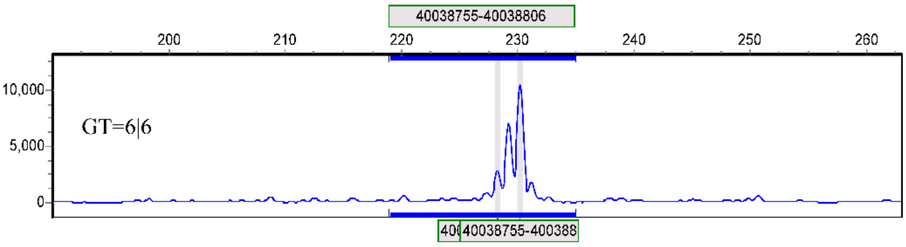

No	Dye	Size	Height	Area	Marker	Allele	Difference	Quality	Score	Allele Comments
1	Blue	228.3	2787	17089	40038755-40038806	40038755-400388		1.3	Pass	40.6
2	Blue	230.2	10566	64042	40038755-40038806	40038755-400388		3.2	Pass	371.2

Supplementary Table 1 Characteristics, sequencing data and variants detected per sample in the discovery cohort

Clinical features	Discovery cohort (fior WGS)		Replication cohort	
	Cases (N=466)	Controls (N=513)	Cases (N=352)	Controls (N=547)
Sex				
Female	193 (41.4%)	309 (60.2%)	131(37.2%)	295 (55.8%)
Male	273 (58.6%)	204 (39.8%)	221(62.8%)	232 (44.2)
Age , year (SD)	61.3 (8.49)	71.9 (6.66)	62.3 (9.3)	69.6 (5.2)
Age at onset, years (SD)	57.9(10.9)	-	58.2 (9.4)	-
Duration, years	7.00 [3.00-8.00]	-	6.00 [4.00-9.00]	-
H-Y stage, Median [Min, Max]	2.00 [2.00-2.50]	-	2.00 [2.00-2.00]	-
MMSE score, Median [Min, Max]	26 [23-27]	28 [26-29]	25 [23-28]	27 [26-29]
RBDQ-HK score, Median [Min, Max]	17 [8-31]	5 [2-13]	17 [8-27]	7 [3-12]
HAMD score, Median [Min, Max]	7 [4-12]	1 [0-3]	7 [2-10]	1 [0-3]
HAMA score, Median [Min, Max]	7 [4-11]	4 [1-7]	8 [4-14]	2 [1-5]
MDS-UPDRS I score, mean（SD)	7.50（3.16)	-	8.00 (3.47)	-
MDS-UPDRS II score, mean (SD)	8.62 (4.83)	-	10.37 (5.79)	-
MDS-UPDRS III score, mean (SD)	23.00 (10.74)	-	25.26 (12.88)	-

Supplementary Table 2 The whole-genome sequencing parameters for the discovery cohort

	Cases (N=466)	Controls (N=513)	Overall (N=979)
**Sequencing base (Gb)**			
Mean (SD)	136 (15.9)	138 (13.4)	137 (14.7)
Median [Min, Max]	139 [41.5, 225]	139 [42.0, 217]	139 [41.5, 225]
**Depth (×)**			
Mean (SD)	44.0 (5.13)	44.6 (4.35)	44.3 (4.75)
Median [Min, Max]	44.9 [13.4, 72.8]	45.1 [13.6, 70.3]	45.0 [13.4, 72.8]
**Coverage**			
Mean (SD)	0.957 (0.000207)	0.957 (0.000213)	0.957 (0.000218)
Median [Min, Max]	0.957 [0.956, 0.958]	0.957 [0.956, 0.958]	0.957 [0.956, 0.958]
**SV**			
Mean (SD)	3110 (110)	3130 (136)	3120 (124)
Median [Min, Max]	3100 [2900, 4440]	3100 [2820, 4520]	3100 [2820, 4520]
**CNV**			
Mean (SD)	2200 (241)	2360 (549)	2280 (438)
Median [Min, Max]	2190 [1550, 3930]	2210 [1680, 4980]	2200 [1550, 4980]
**kSTR**			
Mean (SD)	110000 (1220)	110000 (1130)	110000 (1190)
Median [Min, Max]	110000 [103000, 123000]	110000 [99700, 123000]	110000 [99700, 123000]
**dSTR**			
Mean (SD)	546 (92.4)	561 (98.6)	554 (95.9)
Median [Min, Max]	557 [114, 887]	564 [143, 895]	559 [114, 895]

Supplementary Table 3 Correlation of CNVs in known genes with risk for Parkinson's disease

Gene	Type	MAF		P-value
		Case	Control	
ADK				
10:74450901-74454300	DEL	0.0023	0.0021	0.478811
10:74511401-74514900	DEL	0.0023	0.0021	0.478811
10:74217501-74222600	DUP	0.0023	0.0085	0.897306
10:74568901-74573800	DUP	0.0046	0.0128	0.904395
10:74602701-74607900	DUP	0.0023	0.0255	0.998386
Sum	DEL	0.0046	0.0043	0.468695
	DUP	0.0092	0.0383	0.997875
DNAJB2				
2:219268301-219284600	DUP	0.0023	0.0021	0.480056
GBA				
1:155235101-155241500	DEL	0.0046	0.0021	0.26036
1:155213201-155252500	DUP	0.0183	0.0085	0.099601
MAPT				
17:45963701-45966000	DEL	0.0046	0.0000	0.071111
PRKN				
6:161839901-161959800	DEL	0.0046	0.0043	0.471335
6:161960101-161983200	DEL	0.0023	0.0021	0.480056
6:161983501-162113800	DEL	0.0092	0.0064	0.316205
6:162519301-162645400	DEL	0.0000	0.0064	0.952673
6:162645701-162740200	DEL	0.0000	0.0043	0.913782
6:161875301-161900600	DUP	0.0023	0.0021	0.480056
6:161902601-161983600	DUP	0.0023	0.0043	0.696568
6:161983801-162009800	DUP	0.0000	0.0043	0.913782
6:162011601-162024000	DUP	0.0000	0.0043	0.913782

Supplementary Table 3 Correlation of CNVs in known genes with risk for Parkinson's disease-continued

Gene	Type	MAF		P-value
		Case	Control	
6:162503201-162510300	DUP	0.0000	0.0043	0.913782
Sum	DEL	0.0138	0.0149	0.562
	DUP	0.0023	0.0085	0.896239
SNCA				
4:89776701-89780500	DEL	0.0023	0.0064	0.823645
4:89700301-89772500	DUP	0.0000	0.0043	0.913782
SPG7				
16:89472001-89503500	DUP	0.0023	0.0043	0.69746
VPS13C				
15:61861901-61868100	DEL	0.0115	0.0064	0.208317
15:62010401-62013500	DEL	0.0046	0.0000	0.071111
15:62015901-62037500	DEL	0.0069	0.0106	0.730104
15:61896201-61902200	DUP	0.0023	0.0085	0.896951
15:62016401-62019500	DUP	0.0000	0.0043	0.913782
Sum	DEL	0.0138	0.0149	0.562972
	DUP	0.0023	0.0106	0.939217

Supplementary Table 4 The long- and short-read sequencing data used for validation of MUC19 SV and SLC2A13 STR

ID	Long read sequencing data			Short-read sequencing （WGS） data		
	Platform	Run ID	Source	Platform	Run ID	Source
HG00099	Pacbio hifi	SRR23922670	Liao et al.,2023	Illumina	ERR3240116	Byrska-Bishop et al., 2022
HG00140	Pacbio hifi	SRR23732295	Liao et al.,2023	Illumina	ERR3240146	Byrska-Bishop et al., 2022
HG00280	Pacbio hifi	SRR23922658	Liao et al.,2023	Illumina	ERR3241788	Byrska-Bishop et al., 2022
HG00323	Pacbio hifi	SRR23732294	Liao et al.,2023	Illumina	ERR3240240	Byrska-Bishop et al., 2022
HG00408	Pacbio hifi	SRR23732283	Liao et al.,2023	Illumina	ERR3988762	Byrska-Bishop et al., 2022
HG00423	Pacbio hifi	SRR18189665	Liao et al.,2023	Illumina	ERR3988765	Byrska-Bishop et al., 2022
HG00544	Pacbio hifi	SRR18189653	Liao et al.,2023	Illumina	ERR3988786	Byrska-Bishop et al., 2022
HG00558	Pacbio hifi	SRR23922640	Liao et al.,2023	Illumina	ERR3988789	Byrska-Bishop et al., 2022
HG00597	Pacbio hifi	SRR23732280	Liao et al.,2023	Illumina	ERR3988799	Byrska-Bishop et al., 2022
HG00609	Pacbio hifi	SRR18189646	Liao et al.,2023	Illumina	ERR3988800	Byrska-Bishop et al., 2022
HG00621	Pacbio hifi	SRR21996948	Liao et al.,2023	Illumina	ERR3988803	Byrska-Bishop et al., 2022
HG00639	Pacbio hifi	SRR23922643	Liao et al.,2023	Illumina	ERR3988808	Byrska-Bishop et al., 2022
HG00642	Pacbio hifi	SRR18158610	Liao et al.,2023	Illumina	ERR3988809	Byrska-Bishop et al., 2022
HG00658	ONT	SRR26371197	Liao et al.,2023	Illumina	ERR3988812	Byrska-Bishop et al., 2022
HG00738	Pacbio hifi	SRR18189645	Liao et al.,2023	Illumina	ERR3988825	Byrska-Bishop et al., 2022
HG01074	Pacbio hifi	SRR23922645	Liao et al.,2023	Illumina	ERR3988833	Byrska-Bishop et al., 2022
HG01081	Pacbio hifi	SRR23922647	Liao et al.,2023	Illumina	ERR3988834	Byrska-Bishop et al., 2022
HG01099	Pacbio hifi	SRR18189644	Liao et al.,2023	Illumina	ERR3988838	Byrska-Bishop et al., 2022
HG01175	Pacbio hifi	SRR21996881	Liao et al.,2023	Illumina	ERR3988851	Byrska-Bishop et al., 2022
HG01192	Pacbio hifi	SRR23732279	Liao et al.,2023	Illumina	ERR3988855	Byrska-Bishop et al., 2022
HG01252	ONT	SRR26371195	Liao et al.,2023	Illumina	ERR3988860	Byrska-Bishop et al., 2022
HG01255	Pacbio hifi	SRR18189643	Liao et al.,2023	Illumina	ERR3988861	Byrska-Bishop et al., 2022
HG01258	Pacbio hifi	SRR13684289	Liao et al.,2023	Illumina	ERR3988862	Byrska-Bishop et al., 2022
HG01261	Pacbio hifi	SRR23732278	Liao et al.,2023	Illumina	ERR3988863	Byrska-Bishop et al., 2022

Supplementary Table 4 The long- and short-read sequencing data used for validation of MUC19 SV and SLC2A13 STR-continued

ID	Long read sequencing data			Short-read sequencing （WGS） data		
	Platform	Run ID	Source	Platform	Run ID	Source
HG01346	Pacbio hifi	SRR18158609	Liao et al.,2023	Illumina	ERR3988870	Byrska-Bishop et al., 2022
HG01358	Pacbio hifi	SRR13684283	Liao et al.,2023	Illumina	ERR3988875	Byrska-Bishop et al., 2022
HG01433	Pacbio hifi	SRR18189642	Liao et al.,2023	Illumina	ERR3988882	Byrska-Bishop et al., 2022
HG01496	Pacbio hifi	SRR18189641	Liao et al.,2023	Illumina	ERR3988889	Byrska-Bishop et al., 2022
HG01884	Pacbio hifi	SRR18158605	Liao et al.,2023	Illumina	ERR3988940	Byrska-Bishop et al., 2022
HG01891	Pacbio hifi	SRR13684280	Liao et al.,2023	Illumina	ERR3988943	Byrska-Bishop et al., 2022
HG01934	Pacbio hifi	SRR18189640	Liao et al.,2023	Illumina	ERR3988952	Byrska-Bishop et al., 2022
HG01943	Pacbio hifi	SRR18189663	Liao et al.,2023	Illumina	ERR3988955	Byrska-Bishop et al., 2022
HG01975	Pacbio hifi	SRR23732277	Liao et al.,2023	Illumina	ERR3988964	Byrska-Bishop et al., 2022
HG01981	Pacbio hifi	SRR18189662	Liao et al.,2023	Illumina	ERR3988966	Byrska-Bishop et al., 2022
HG01993	Pacbio hifi	SRR18189661	Liao et al.,2023	Illumina	ERR3988970	Byrska-Bishop et al., 2022
HG02004	Pacbio hifi	SRR18189660	Liao et al.,2023	Illumina	ERR3988972	Byrska-Bishop et al., 2022
HG02015	Pacbio hifi	SRR23732276	Liao et al.,2023	Illumina	ERR3988974	Byrska-Bishop et al., 2022
HG02027	Pacbio hifi	SRR18189659	Liao et al.,2023	Illumina	ERR3988977	Byrska-Bishop et al., 2022
HG02040	Pacbio hifi	SRR23922646	Liao et al.,2023	Illumina	ERR3242342	Byrska-Bishop et al., 2022
HG02056	Pacbio hifi	SRR23732275	Liao et al.,2023	Illumina	ERR3988980	Byrska-Bishop et al., 2022
HG02071	Pacbio hifi	SRR18158592	Liao et al.,2023	Illumina	ERR3988983	Byrska-Bishop et al., 2022

Supplementary Table 4 The long- and short-read sequencing data used for validation of MUC19 SV and SLC2A13 STR-continued

ID	Long read sequencing data			Short-read sequencing （WGS） data		
	Platform	Run ID	Source	Platform	Run ID	Source
HG02074	Pacbio hifi	SRR18158594	Liao et al.,2023	Illumina	ERR3988984	Byrska-Bishop et al., 2022
HG02083	Pacbio hifi	SRR18189658	Liao et al.,2023	Illumina	ERR3988987	Byrska-Bishop et al., 2022
HG02129	Pacbio hifi	SRR23732274	Liao et al.,2023	Illumina	ERR3988992	Byrska-Bishop et al., 2022
HG02132	Pacbio hifi	SRR18158595	Liao et al.,2023	Illumina	ERR3988993	Byrska-Bishop et al., 2022
HG02135	Pacbio hifi	SRR18158585	Liao et al.,2023	Illumina	ERR3988994	Byrska-Bishop et al., 2022
HG02155	Pacbio hifi	SRR23732293	Liao et al.,2023	Illumina	ERR3242169	Byrska-Bishop et al., 2022
HG02165	Pacbio hifi	SRR23922648	Liao et al.,2023	Illumina	ERR3242172	Byrska-Bishop et al., 2022
HG02257	Pacbio hifi	SRR13684279	Liao et al.,2023	Illumina	ERR3989003	Byrska-Bishop et al., 2022
HG02258	Pacbio hifi	SRR23732292	Liao et al.,2023	Illumina	ERR3989004	Byrska-Bishop et al., 2022
HG02735	Pacbio hifi	SRR23922665	Liao et al.,2023	Illumina	ERR3989063	Byrska-Bishop et al., 2022
HG02738	Pacbio hifi	SRR18158588	Liao et al.,2023	Illumina	ERR3989064	Byrska-Bishop et al., 2022
HG02809	Pacbio hifi	SRR18189651	Liao et al.,2023	Illumina	ERR3989077	Byrska-Bishop et al., 2022
HG02922	Pacbio hifi	SRR23922663	Liao et al.,2023	Illumina	ERR3242482	Byrska-Bishop et al., 2022
HG02965	Pacbio hifi	SRR23732290	Liao et al.,2023	Illumina	ERR3989100	Byrska-Bishop et al., 2022
HG02976	Pacbio hifi	SRR23732289	Liao et al.,2023	Illumina	ERR3242607	Byrska-Bishop et al., 2022
HG03017	Pacbio hifi	SRR23922662	Liao et al.,2023	Illumina	ERR3989108	Byrska-Bishop et al., 2022
HG03041	Pacbio hifi	SRR23922661	Liao et al.,2023	Illumina	ERR3989114	Byrska-Bishop et al., 2022

Supplementary Table 4 The long- and short-read sequencing data used for validation of MUC19 SV and SLC2A13 STR-continued

ID	Long read sequencing data			Short-read sequencing （WGS） data		
	Platform	Run ID	Source	Platform	Run ID	Source
HG03130	Pacbio hifi	SRR23922660	Liao et al.,2023	Illumina	ERR3242610	Byrska-Bishop et al., 2022
HG03139	Pacbio hifi	SRR23922659	Liao et al.,2023	Illumina	ERR3242983	Byrska-Bishop et al., 2022
HG03195	Pacbio hifi	SRR23732288	Liao et al.,2023	Illumina	ERR3242620	Byrska-Bishop et al., 2022
HG03209	Pacbio hifi	SRR23922657	Liao et al.,2023	Illumina	ERR3242539	Byrska-Bishop et al., 2022
HG03225	Pacbio hifi	SRR23732287	Liao et al.,2023	Illumina	ERR3242964	Byrska-Bishop et al., 2022
HG03239	Pacbio hifi	SRR23922656	Liao et al.,2023	Illumina	ERR3989139	Byrska-Bishop et al., 2022
HG03453	Pacbio hifi	SRR21996941	Liao et al.,2023	Illumina	ERR3989166	Byrska-Bishop et al., 2022
HG03516	Pacbio hifi	SRR13684285	Liao et al.,2023	Illumina	ERR3989174	Byrska-Bishop et al., 2022
HG03654	Pacbio hifi	SRR18189650	Liao et al.,2023	Illumina	ERR3989196	Byrska-Bishop et al., 2022
HG03669	Pacbio hifi	SRR18189649	Liao et al.,2023	Illumina	ERR3989197	Byrska-Bishop et al., 2022
HG03688	Pacbio hifi	SRR18158607	Liao et al.,2023	Illumina	ERR3989200	Byrska-Bishop et al., 2022
HG03704	Pacbio hifi	SRR23922654	Liao et al.,2023	Illumina	ERR3989204	Byrska-Bishop et al., 2022
HG03710	Pacbio hifi	SRR18189648	Liao et al.,2023	Illumina	ERR3989206	Byrska-Bishop et al., 2022
HG03831	Pacbio hifi	SRR18189647	Liao et al.,2023	Illumina	ERR3989224	Byrska-Bishop et al., 2022
NA19043	Pacbio hifi	SRR23922639	Liao et al.,2023	Illumina	ERR3239700	Byrska-Bishop et al., 2022
NA20752	Pacbio hifi	SRR23922641	Liao et al.,2023	Illumina	ERR3239832	Byrska-Bishop et al., 2022
NA20805	Pacbio hifi	SRR23732281	Liao et al.,2023	Illumina	ERR3239873	Byrska-Bishop et al., 2022

Supplementary Table 4 The long- and short-read sequencing data used for validation of MUC19 SV and SLC2A13 STR-continued

ID	Long read sequencing data			Short-read sequencing （WGS） data		
	Platform	Run ID	Source	Platform	Run ID	Source
NA20905	Pacbio hifi	SRR23922642	Liao et al.,2023	Illumina	ERR3240106	Byrska-Bishop et al., 2022
AK1	Pacbio hifi	SRR3575394-SRR3575493	Seo et al., 2016.	Illumina	SRR3602738	Seo et al., 2016
CHM1	Pacbio hifi	SRR1304331-SRR1304557	Chaisson et al., 2015	Illumina	SRR1514950-SRR1514952	Chaisson et al., 2015
HG00514	Pacbio hifi	https://www.internationalgenome.org	Chaisson et al., 2019	Illumina	ERR3988781	Byrska-Bishop et al., 2022
HG00732	Pacbio hifi	https://www.internationalgenome.org	Chaisson et al., 2019	Illumina	ERR3241755	Byrska-Bishop et al., 2022
HG00733	Pacbio hifi	https://www.internationalgenome.org	Chaisson et al., 2019	Illumina	ERR3988823	Byrska-Bishop et al., 2022
HG00268	Pacbio hifi	SRR7511979-SRR7512008	Audano et al., 2019	Illumina	ERR3240225	Byrska-Bishop et al., 2022
HG01352	Pacbio hifi	SRR4115411-SRR4115460	Audano et al., 2019	Illumina	ERR3988873	Byrska-Bishop et al., 2022
HG01596	Pacbio hifi	ERR5101153	Chaisson et al., 2019	Illumina	ERR3242063	Byrska-Bishop et al., 2022
HG02011	Pacbio hifi	ERR3861411, ERR3861412	Chaisson et al., 2019	Illumina	ERR3988973	Byrska-Bishop et al., 2022
HG02059	Pacbio hifi	SRR5228709-SRR5228758	Audano et al., 2019	Illumina	ERR3988981	Byrska-Bishop et al., 2022
HG02106	Pacbio hifi	SRR7515657-SRR7515701	Audano et al., 2019	Illumina	ERR3988989	Byrska-Bishop et al., 2022
HG02492	Pacbio hifi	ERR3861400	Chaisson et al., 2019	Illumina	ERR3989019	Byrska-Bishop et al., 2022
HG02587	Pacbio hifi	ERR4968417	Chaisson et al., 2019	Illumina	ERR3989031	Byrska-Bishop et al., 2022
HG02818	Pacbio hifi	SRR6056437-SRR6056467	Audano et al., 2019	Illumina	ERR3989080	Byrska-Bishop et al., 2022
HG03683	Pacbio hifi	ERR3861403	Chaisson et al., 2019	Illumina	ERR3989199	Byrska-Bishop et al., 2022
HG03732	Pacbio hifi	ERR5101158	Chaisson et al., 2019	Illumina	ERR3989210	Byrska-Bishop et al., 2022

Supplementary Table 4 The long- and short-read sequencing data used for validation of MUC19 SV and SLC2A13 STR-continued

ID	Long read sequencing data			Short-read sequencing （WGS） data		
	Platform	Run ID	Source	Platform	Run ID	Source
NA19043	Pacbio hifi	SRR23922639	Liao et al.,2023	Illumina	ERR3239700	Byrska-Bishop et al., 2022
NA20752	Pacbio hifi	SRR23922641	Liao et al.,2023	Illumina	ERR3239832	Byrska-Bishop et al., 2022
NA20805	Pacbio hifi	SRR23732281	Liao et al.,2023	Illumina	ERR3239873	Byrska-Bishop et al., 2022
NA20905	Pacbio hifi	SRR23922642	Liao et al.,2023	Illumina	ERR3240106	Byrska-Bishop et al., 2022
AK1	Pacbio hifi	SRR3575394-SRR3575493	Seo et al., 2016.	Illumina	SRR3602738	Seo et al., 2016
HG04217	Pacbio hifi	SRR7538470SRR7538517	Audano et al., 2019	Illumina	ERR3989261	Byrska-Bishop et al., 2022
NA12878	Pacbio hifi	SRR3658270-SRR3658319	Audano et al., 2019	Illumina	ERR3239334	Byrska-Bishop et al., 2022
NA19238	Pacbio hifi	https://www.internationalgenome.org	Chaisson et al., 2019	Illumina	ERR3239453	Byrska-Bishop et al., 2022
NA19239	Pacbio hifi	ERR4914942, ERR4914959, ERR4914962, ERR4914966, ERR4914967	-	Illumina	ERR3239454	Byrska-Bishop et al., 2022
NA19240	Pacbio hifi	https://www.internationalgenome.org	Chaisson et al., 2019	Illumina	ERR3989410	Byrska-Bishop et al., 2022
NA19434	Pacbio hifi	SRR7209475-SRR7257576	Audano et al., 2019	Illumina	ERR3239756	Byrska-Bishop et al., 2022
NA19983	Pacbio hifi	ERR3861402	Chaisson et al., 2019	Illumina	ERR3989454	Byrska-Bishop et al., 2022
HX1	ONT	SRR9001343-SRR9001361	Shi et al., 2016	Illumina	SRR8732413	Shi et al., 2016.

References

1. Liao WW, Asri M, Ebler J, et al. A draft human pangenome reference. Nature, 2023, 617(7960), 312–324. 2. Byrska-Bishop M, Evani US, Zhao X, et al. High-coverage whole-genome sequencing of the expanded
1000 Genomes Project cohort including 602 trios. Cell, 2022, 185(18), 3426–3440.e19.
3. Seo JS, Rhie A, Kim J, et al. De novo assembly and phasing of a Korean human genome. Nature, 2016, 538(7624), 243–247.

4. Chaisson MJ, Huddleston J, Dennis MY, et al. Resolving the complexity of the human genome using single-molecule sequencing. Nature, 2015; 517(7536), 608–611.
5.Miga KH, Koren S, Rhie A, et al. Telomere-to-telomere assembly of a complete human X chromosome. Nature, 2020; 585(7823), 79–84.
6. Zook JM, Hansen NF, Olson ND, et al. A robust benchmark for detection of germline large deletions and insertions. Nature biotechnology, 2020;38(11), 1347–1355.
7. Chaisson MJP, Sanders AD, Zhao X, et al. Multi-platform discovery of haplotype-resolved structural variation in human genomes. Nature communications, 2019;10(1), 1784.
8. Audano PA, Sulovari A, Graves-Lindsay TA, et .al. Characterizing the Major Structural Variant Alleles of the Human Genome. Cell, 2019;176(3), 663–675.e19.
9. Shi L, Guo Y, Dong C, et al. Long-read sequencing and de novo assembly of a Chinese genome. Nature communications, 2016; 7, 12065.

Supplementary Table 5 Primer sequences for kSTRs in the *PPP3CA, SLC2A13* and *ZNF609* genes

Gene	Primers	Sequence	Product length (bp)	Annealing temperature
PPP3CA	p36048-F	5’-CTATTCCTCACCCAGCATGTTT-3’	411bp	58℃
	p36048-R	5’-TTTCAAGGGACTCCAATAGACTG-3’		
	Repeat	AC		
	Sequencing	5’-GGAGGAGGACCTAGAAACTGG-3’		
SLC2A13	p38759-F	5’-GCAGTACAATACTATTAAATACCTTGTC-3’	305bp	58℃
	p38759-R	5’-GG’GCATGGTGGCATGTACTTC-3’		
	Repeat	GGGAAA		
	Sequencing	5’-AAATACCTTGTCAAAACTGACATTT-3’		
ZNF609	p65232-F	5’-GCAGTGGCGTGATCTCTGTTT-3’	277bp	58℃
	p65232-R	5’-GATGTTAGTGGCTGGGTGTGG-3’		
	Repeat	TAT		
	Sequencing	5’-CTTTGGGAGGTCAAGGTGGGT-3’		

Supplementary Table 6 Primer sequences for dSTRs in the *RNPC3, GOLGA8A* and *DAGLB* genes

Gene	Primers	Sequence	Product length	Annealing temp
RNPC3	Forward	5’-TAAGAGCCGCACGGTCAGCT -3’	274bp	60℃
	Reverse	5’-AAGGTTGACGCTCAAGGACAA-3’		
	Repeat unit	ACGGCGGGGCGGGGCGC	17bp	
GOLGA8A	Forward	5’-GCTAGGCGCGAGCTCTGC-3’	486bp	60℃
	Reverse	5’-CTCTTGGCCCGCAGCTTAC-3’		
	Repeat unit	ACGGCGGCGCGGCGAGG	17bp	
DAGLB	Forward	5’-AGATGGGTGCCTCGGGTC-3’	500bp	60℃
	Reverse	5’-CTCAAATGCCCAACCTGCTAC-3’		
	Repeat unit	AGCGCGGGAGGCGCAGGC	18bp	

Supplementary Table 7 Primer sequences for the qPCR amplification

Chr. region	Primers	Forward sequence	Reverse sequence
2q14.1	2q141-qF1	5'- CCAGACTTGGGGAAGCATTC -3'	5'- AATGAATGACCATCTTGGCAAT -3'
	2q141-qF2	5'- TTAGGGACCATCACAACATCCA -3'	5'- TTTAGGAAATATGTCCAGAATCCAAT -3'
	2q141-qF3	5'- TGCCTTTCCAATACTACTTTTCAC -3'	5'--3'ATACCACTCCGTGTGCTAAATTC
2q143	2q143-qF1	5'- GGCTGAAACCTTCACCCTTT -3'	5'--3'GAGACCAGTCAGATGGGAGAACT
	2q143-qF2	5'- CATTGCTAAATACCCAGCCAT -3'	5'--3'CTTCAAGGAGCTTTCGATCAA
	2q143-qF3	5'- GCCTTGGGACTGAAGAGCCT -3'	5'--3'GCAGAAAAGACAGGGAGTGAACAA
3p241	3p241-qF1	5'- TGTCTGTTCCATTTTGCCCT -3'	5'--3'CATGAATCCAATTTATAGCACCA
	3p241-qF2	5'- GGATGTCGATTTCATTAACACAAC -3'	5'- TGAGAAGTCCAAAGAATGCACA -3'
	3p241-qF3	5'- GAGCCTGTGTTCCTACCTGATTC -3'	5'- CGGGTCCTAGACGCAGAACTA -3'
4q321	4q321-qF1	5'- TAATGGCTAATGATGTTGAATATCCT -3'	5'- GAACCCTCAAGACAAACAATACAAT -3'
	4q321-qF2	5'- TTTCTCATCAAGGCATACATTTTCT -3'	5'- GGAGTTCTACACCCTAAGTGGAAAC -3'
	4q321-qF3	5'- TCTATAAGGTATCTGGGAACACATTC -3'	5'- CATGGAGTATAATGGTAGCTGTAACC -3'

Supplementary Table 8 Symbols for dosage-sensitive and loss-of-function tolerant genes

Gene symbol	Panel	Gene symbol	Panel
ANK2	Dosage-sensitive	*A2M*	Loss-of-function tolerance
APC	Dosage-sensitive	*ABCA10*	Loss-of-function tolerance
BCL11B	Dosage-sensitive	*ABCA8*	Loss-of-function tolerance
CD2AP	Dosage-sensitive	*ABCC11*	Loss-of-function tolerance
COMT	Dosage-sensitive	*ABCC12*	Loss-of-function tolerance
CSF2RA	Dosage-sensitive	*ABHD12B*	Loss-of-function tolerance
DMPK	Dosage-sensitive	*ABHD14B*	Loss-of-function tolerance
DYRK1A	Dosage-sensitive	*ACSBG2*	Loss-of-function tolerance
EGR1	Dosage-sensitive	*ACSM2A*	Loss-of-function tolerance
EHMT1	Dosage-sensitive	*ACSM2B*	Loss-of-function tolerance
FGF10	Dosage-sensitive	*ACSM3*	Loss-of-function tolerance
FOXP1	Dosage-sensitive	*ADAM2*	Loss-of-function tolerance
FOXP2	Dosage-sensitive	*ADPRHL1*	Loss-of-function tolerance
GCH1	Dosage-sensitive	*ADSSL1*	Loss-of-function tolerance
GHRL	Dosage-sensitive	*AHNAK2*	Loss-of-function tolerance
GTF2I	Dosage-sensitive	*AKAP3*	Loss-of-function tolerance
HOXD13	Dosage-sensitive	*ALDH1B1*	Loss-of-function tolerance
IGF1	Dosage-sensitive	*ANKRD30A*	Loss-of-function tolerance
KCNQ2	Dosage-sensitive	*ANKRD35*	Loss-of-function tolerance
LMX1B	Dosage-sensitive	*ANO5*	Loss-of-function tolerance
MAPT	Dosage-sensitive	*AP1G2*	Loss-of-function tolerance
MC4R	Dosage-sensitive	*APIP*	Loss-of-function tolerance
NF1	Dosage-sensitive	*APOBEC3A*	Loss-of-function tolerance
NKX2-5	Dosage-sensitive	*APOBEC3B*	Loss-of-function tolerance
NLGN4X	Dosage-sensitive	*ASB15*	Loss-of-function tolerance
NLRP3	Dosage-sensitive	*ASPSCR1*	Loss-of-function tolerance
NPAS3	Dosage-sensitive	*ATP10B*	Loss-of-function tolerance

NSD1	Dosage-sensitive	*ATP11A*	Loss-of-function tolerance
PARK2	Dosage-sensitive	*ATP12A*	Loss-of-function tolerance
PAX6	Dosage-sensitive	*ATP2C2*	Loss-of-function tolerance
PIK3R1	Dosage-sensitive	*BPHL*	Loss-of-function tolerance
PRODH	Dosage-sensitive	*BPIFA3*	Loss-of-function tolerance
PTEN	Dosage-sensitive	*BTN3A3*	Loss-of-function tolerance
RELN	Dosage-sensitive	*BTNL2*	Loss-of-function tolerance
SATB2	Dosage-sensitive	*BTNL8*	Loss-of-function tolerance
SCN1A	Dosage-sensitive	*BTNL9*	Loss-of-function tolerance
SEMA5A	Dosage-sensitive	*C10orf53*	Loss-of-function tolerance
SHFM1	Dosage-sensitive	*C11orf40*	Loss-of-function tolerance
SHMT1	Dosage-sensitive	*C1orf127*	Loss-of-function tolerance
SNCA	Dosage-sensitive	*C2orf40*	Loss-of-function tolerance
SPR	Dosage-sensitive	*C3orf14*	Loss-of-function tolerance
ST7	Dosage-sensitive	*C4orf46*	Loss-of-function tolerance
TBX1	Dosage-sensitive	*C4orf50*	Loss-of-function tolerance
TCF4	Dosage-sensitive	*C6*	Loss-of-function tolerance
TGFB1	Dosage-sensitive	*CABYR*	Loss-of-function tolerance
TPM1	Dosage-sensitive	*CAPN9*	Loss-of-function tolerance
TSC1	Dosage-sensitive	*CARD6*	Loss-of-function tolerance
TSC2	Dosage-sensitive	*CCDC121*	Loss-of-function tolerance
WWOX	Dosage-sensitive	*CCDC13*	Loss-of-function tolerance
		CCDC60	Loss-of-function tolerance
		CCDC66	Loss-of-function tolerance
		CD180	Loss-of-function tolerance
		CD36	Loss-of-function tolerance
		CD96	Loss-of-function tolerance
		CDH19	Loss-of-function tolerance
		CDK11A	Loss-of-function tolerance
		CDKL2	Loss-of-function tolerance

CELA1	Loss-of-function tolerance
CEP72	Loss-of-function tolerance
CES1	Loss-of-function tolerance
CES5A	Loss-of-function tolerance
CFHR1	Loss-of-function tolerance
CFHR2	Loss-of-function tolerance
CFHR3	Loss-of-function tolerance
CHD1L	Loss-of-function tolerance
CHIT1	Loss-of-function tolerance
CHPF2	Loss-of-function tolerance
CLCN1	Loss-of-function tolerance
CLYBL	Loss-of-function tolerance
CNKSR1	Loss-of-function tolerance
COL16A1	Loss-of-function tolerance
COL6A2	Loss-of-function tolerance
COL6A5	Loss-of-function tolerance
CPXM2	Loss-of-function tolerance
CROT	Loss-of-function tolerance
CRYGN	Loss-of-function tolerance
CRYZ	Loss-of-function tolerance
CSH1	Loss-of-function tolerance
CTSE	Loss-of-function tolerance
CYP2A6	Loss-of-function tolerance
CYP2C8	Loss-of-function tolerance
CYP2D6	Loss-of-function tolerance
CYP2F1	Loss-of-function tolerance
CYP3A5	Loss-of-function tolerance
CYP4B1	Loss-of-function tolerance
DCDC2B	Loss-of-function tolerance
DCHS2	Loss-of-function tolerance

DDX60	Loss-of-function tolerance
DEFB126	Loss-of-function tolerance
DHDH	Loss-of-function tolerance
DMBT1	Loss-of-function tolerance
DNAH7	Loss-of-function tolerance
DQX1	Loss-of-function tolerance
DUOX2	Loss-of-function tolerance
ECT2L	Loss-of-function tolerance
EFCAB13	Loss-of-function tolerance
EFCAB3	Loss-of-function tolerance
EFCAB5	Loss-of-function tolerance
EFCAB6	Loss-of-function tolerance
ENOSF1	Loss-of-function tolerance
ENPEP	Loss-of-function tolerance
EPPK1	Loss-of-function tolerance
EPX	Loss-of-function tolerance
ERAP1	Loss-of-function tolerance
ERV3-1	Loss-of-function tolerance
EXO5	Loss-of-function tolerance
FAM129C	Loss-of-function tolerance
FAM151A	Loss-of-function tolerance
FAM187B	Loss-of-function tolerance
FAM45A	Loss-of-function tolerance
FAM81B	Loss-of-function tolerance
FCGBP	Loss-of-function tolerance
FCGR2A	Loss-of-function tolerance
FCN3	Loss-of-function tolerance
FLG	Loss-of-function tolerance
FLG2	Loss-of-function tolerance
FMO2	Loss-of-function tolerance

FRG2B	Loss-of-function tolerance
FUT2	Loss-of-function tolerance
FUT6	Loss-of-function tolerance
GADL1	Loss-of-function tolerance
GBGT1	Loss-of-function tolerance
GBP3	Loss-of-function tolerance
GBP4	Loss-of-function tolerance
GCFC2	Loss-of-function tolerance
GH2	Loss-of-function tolerance
GJB4	Loss-of-function tolerance
GLB1L2	Loss-of-function tolerance
GMPR	Loss-of-function tolerance
GOLGA8S	Loss-of-function tolerance
GP6	Loss-of-function tolerance
GPATCH2L	Loss-of-function tolerance
GRIN3B	Loss-of-function tolerance
GRK7	Loss-of-function tolerance
GYPB	Loss-of-function tolerance
HELB	Loss-of-function tolerance
HK3	Loss-of-function tolerance
HLA-B	Loss-of-function tolerance
HLA-DPA1	Loss-of-function tolerance
HPSE	Loss-of-function tolerance
HRG	Loss-of-function tolerance
HRNR	Loss-of-function tolerance
IDI2	Loss-of-function tolerance
IFIH1	Loss-of-function tolerance
IFNK	Loss-of-function tolerance
IL17RC	Loss-of-function tolerance
IL3RA	Loss-of-function tolerance

IQCH	Loss-of-function tolerance
ITIH1	Loss-of-function tolerance
KIAA0753	Loss-of-function tolerance
KIAA1257	Loss-of-function tolerance
KIAA1586	Loss-of-function tolerance
KIR3DL1	Loss-of-function tolerance
KLK14	Loss-of-function tolerance
KLK3	Loss-of-function tolerance
KRT4	Loss-of-function tolerance
KRT77	Loss-of-function tolerance
KRT83	Loss-of-function tolerance
LMF2	Loss-of-function tolerance
LMO7	Loss-of-function tolerance
LPA	Loss-of-function tolerance
LRRC39	Loss-of-function tolerance
LRTM1	Loss-of-function tolerance
MANEA	Loss-of-function tolerance
MAP3K4	Loss-of-function tolerance
MAZ	Loss-of-function tolerance
MCF2L	Loss-of-function tolerance
MCOLN3	Loss-of-function tolerance
MFSD9	Loss-of-function tolerance
MGAM	Loss-of-function tolerance
MLANA	Loss-of-function tolerance
MMP10	Loss-of-function tolerance
MOGAT1	Loss-of-function tolerance
MOK	Loss-of-function tolerance
MOXD1	Loss-of-function tolerance
MS4A6A	Loss-of-function tolerance
MST1	Loss-of-function tolerance

MUC17	Loss-of-function tolerance
MUC6	Loss-of-function tolerance
MUTYH	Loss-of-function tolerance
MYBBP1A	Loss-of-function tolerance
MYH1	Loss-of-function tolerance
MYH13	Loss-of-function tolerance
MYH8	Loss-of-function tolerance
MYO1A	Loss-of-function tolerance
MYOC	Loss-of-function tolerance
MYOF	Loss-of-function tolerance
NAALAD2	Loss-of-function tolerance
NBPF14	Loss-of-function tolerance
NBPF15	Loss-of-function tolerance
NEIL1	Loss-of-function tolerance
NLRP13	Loss-of-function tolerance
NLRP9	Loss-of-function tolerance
NOP16	Loss-of-function tolerance
NUDT8	Loss-of-function tolerance
OARD1	Loss-of-function tolerance
OBSCN	Loss-of-function tolerance
OCEL1	Loss-of-function tolerance
OR8S1	Loss-of-function tolerance
PAPLN	Loss-of-function tolerance
PDE11A	Loss-of-function tolerance
PDIA2	Loss-of-function tolerance
PGPEP1L	Loss-of-function tolerance
PHRF1	Loss-of-function tolerance
PKD1L2	Loss-of-function tolerance
PKHD1L1	Loss-of-function tolerance
PLA2G2C	Loss-of-function tolerance

PLA2G4D	Loss-of-function tolerance
PLA2R1	Loss-of-function tolerance
PLEKHG7	Loss-of-function tolerance
PLIN4	Loss-of-function tolerance
PNLIPRP3	Loss-of-function tolerance
POLM	Loss-of-function tolerance
POTEH	Loss-of-function tolerance
PPEF2	Loss-of-function tolerance
PPL	Loss-of-function tolerance
PPP1R3A	Loss-of-function tolerance
PRAMEF2	Loss-of-function tolerance
PRB1	Loss-of-function tolerance
PRB2	Loss-of-function tolerance
PRB4	Loss-of-function tolerance
PSG1	Loss-of-function tolerance
PSG11	Loss-of-function tolerance
PSG4	Loss-of-function tolerance
PSG9	Loss-of-function tolerance
PTCHD3	Loss-of-function tolerance
PTGDR	Loss-of-function tolerance
PXDNL	Loss-of-function tolerance
PZP	Loss-of-function tolerance
RAI1	Loss-of-function tolerance
RERGL	Loss-of-function tolerance
RETSAT	Loss-of-function tolerance
RFPL1	Loss-of-function tolerance
RGPD4	Loss-of-function tolerance
RGS11	Loss-of-function tolerance
RHD	Loss-of-function tolerance
RNF32	Loss-of-function tolerance

ROPN1B	Loss-of-function tolerance
RP1L1	Loss-of-function tolerance
RPTN	Loss-of-function tolerance
RTKN2	Loss-of-function tolerance
RTP1	Loss-of-function tolerance
SAMD11	Loss-of-function tolerance
SEMG2	Loss-of-function tolerance
SERHL2	Loss-of-function tolerance
SERPINA10	Loss-of-function tolerance
SERPINA9	Loss-of-function tolerance
SERPINB3	Loss-of-function tolerance
SFI1	Loss-of-function tolerance
SIGLEC1	Loss-of-function tolerance
SIGLEC5	Loss-of-function tolerance
SLC17A9	Loss-of-function tolerance
SLC22A10	Loss-of-function tolerance
SLC22A14	Loss-of-function tolerance
SLC22A25	Loss-of-function tolerance
SLC26A10	Loss-of-function tolerance
SLC5A4	Loss-of-function tolerance
SLCO1B1	Loss-of-function tolerance
SLFN13	Loss-of-function tolerance
SPATA31A6	Loss-of-function tolerance
SPATA4	Loss-of-function tolerance
SPATC1	Loss-of-function tolerance
SPNS3	Loss-of-function tolerance
SULT1A2	Loss-of-function tolerance
SULT1C4	Loss-of-function tolerance
SYNM	Loss-of-function tolerance
SYTL2	Loss-of-function tolerance

TAF6	Loss-of-function tolerance
TCF3	Loss-of-function tolerance
TCHHL1	Loss-of-function tolerance
TEKT3	Loss-of-function tolerance
TGM4	Loss-of-function tolerance
THBS4	Loss-of-function tolerance
THEM5	Loss-of-function tolerance
TIGD6	Loss-of-function tolerance
TLR10	Loss-of-function tolerance
TLR5	Loss-of-function tolerance
TMC2	Loss-of-function tolerance
TMEM82	Loss-of-function tolerance
TMIE	Loss-of-function tolerance
TMPRSS7	Loss-of-function tolerance
TNN	Loss-of-function tolerance
TRIM22	Loss-of-function tolerance
TRIM45	Loss-of-function tolerance
TRIM48	Loss-of-function tolerance
TRIM59	Loss-of-function tolerance
TRMT10B	Loss-of-function tolerance
TRMT2A	Loss-of-function tolerance
TTC38	Loss-of-function tolerance
TTN	Loss-of-function tolerance
UGT2B10	Loss-of-function tolerance
UGT2B17	Loss-of-function tolerance
UGT2B28	Loss-of-function tolerance
UMODL1	Loss-of-function tolerance
UNC93A	Loss-of-function tolerance
UPB1	Loss-of-function tolerance
UPK3A	Loss-of-function tolerance

UPP2	Loss-of-function tolerance
USP45	Loss-of-function tolerance
USP6	Loss-of-function tolerance
VILL	Loss-of-function tolerance
VWA3B	Loss-of-function tolerance
VWA7	Loss-of-function tolerance
WDR27	Loss-of-function tolerance
WDR90	Loss-of-function tolerance
XIRP1	Loss-of-function tolerance
XRRA1	Loss-of-function tolerance
ZAN	Loss-of-function tolerance
ZNF223	Loss-of-function tolerance
ZNF229	Loss-of-function tolerance
ZNF257	Loss-of-function tolerance
ZNF30	Loss-of-function tolerance
ZNF343	Loss-of-function tolerance
ZNF396	Loss-of-function tolerance
ZNF417	Loss-of-function tolerance
ZNF486	Loss-of-function tolerance
ZNF528	Loss-of-function tolerance
ZNF544	Loss-of-function tolerance
ZNF587	Loss-of-function tolerance
ZNF599	Loss-of-function tolerance
ZNF611	Loss-of-function tolerance
ZNF790	Loss-of-function tolerance
ZNF83	Loss-of-function tolerance
ZNF831	Loss-of-function tolerance
ZNF844	Loss-of-function tolerance
ZNF846	Loss-of-function tolerance
ZNF860	Loss-of-function tolerance

ZNF878	Loss-of-function tolerance
ZNF92	Loss-of-function tolerance
ZRANB3	Loss-of-function tolerance
