# Supplementary material for: High-depth whole-genome sequencing identifies structure variants, copy number variants and short tandem repeats associated with Parkinson’s disease
Source: NPJ Parkinsons Dis. 2024 Jul 23;10:134. doi: 10.1038/s41531-024-00722-1 (PMC11266557; doi:10.1038/s41531-024-00722-1)
Supplement: Supplementary file 2 — Reporting summary checklist [file 41531_2024_722_MOESM2_ESM.pdf]

nature portfolio

Corresponding author(s): Piu Chan

Last updated by author(s): May 1, 2024

Reporting Summary

Nature Portfolio wishes to improve the reproducibility of the work that we publish. This form provides structure for consistency and transparency in reporting. For further information on Nature Portfolio policies, see our Editorial Policies and the Editorial Policy Checklist.

Statistics

For all statistical analyses, confirm that the following items are present in the figure legend, table legend, main text, or Methods section.

n/a	Confirmed	
☐	☒	The exact sample size (*n*) for each experimental group/condition, given as a discrete number and unit of measurement
☐	☒	A statement on whether measurements were taken from distinct samples or whether the same sample was measured repeatedly
☐	☒	The statistical test(s) used AND whether they are one- or two-sided<br>*Only common tests should be described solely by name; describe more complex techniques in the Methods section.*
☐	☒	A description of all covariates tested
☐	☒	A description of any assumptions or corrections, such as tests of normality and adjustment for multiple comparisons
☐	☒	A full description of the statistical parameters including central tendency (e.g. means) or other basic estimates (e.g. regression coefficient) AND variation (e.g. standard deviation) or associated estimates of uncertainty (e.g. confidence intervals)
☐	☒	For null hypothesis testing, the test statistic (e.g. *F*, *t*, *r*) with confidence intervals, effect sizes, degrees of freedom and *P* value noted<br>*Give P values as exact values whenever suitable.*
☐	☒	For Bayesian analysis, information on the choice of priors and Markov chain Monte Carlo settings
☒	☐	For hierarchical and complex designs, identification of the appropriate level for tests and full reporting of outcomes
☒	☐	Estimates of effect sizes (e.g. Cohen's *d*, Pearson's *r*), indicating how they were calculated

Our web collection on statistics for biologists contains articles on many of the points above.

Software and code

Policy information about availability of computer code

Data collection	No specific software was used.
Data analysis	Analyses were performed using the following public software packages: i Short-read alignment (BWA, https://github.com/lh3/bwa; samblaster, https://github.com/GregoryFaust/samblaster; sambamba, https://github.com/biod/sambamba; Samtools, https://github.com/samtools/samtools); (ii) Variant calling of SV (LUMPY, https://gith ub.co m/arq5x/lumpy-sv; SVTyper, https://github.com/hall-lab/svtyper), repeat-expansion (ExpansionHunter, https://github.com/Illumina/ExpansionHunter; ExpansionHunterDenovo, https://github.com/Illumina/ExpansionHunterDenovo), CNV (CNVpytor, https://github.com/abyzo vlab/CNVpytor), and SNV (GATK4, https://gatk.broadinstitute.org); (iii) Long-read alignment (ngmlr, https://github.com/philres/ngmlr) and genotype of MUC19 SV (sniffles, https://github.com/fritzsedlazeck/Sniffles), SLC2A13 GGGAAA repeat (NanoRepeat, https://github.com/WGLab/NanoRepeat); (iv) Variant filter, merge, and variant annotation (vawk, https://github. com/cc2qe/vawk; bedtools, https://github. com/arq5x /bedtools2; bcftools, https:// github.com/samtools/bcftools; tabix, https://github.com/tabixio/tabix; svtools, https://github.com/hall-lab/svtools; AnnotSV, https://github.com/lgmgeo/Annot SV); (v) Association test and data visualization (R, https://www.r-project.org; Samplot, https://github.com/ryanlayer/samplot); (vi) Genotype phasing and linkage disequilibrium calculation (Beagle, http://faculty.washington.edu/browning/beagle/beagle.html; vcftools, https://github.com/vcftools/vcftools); (vii) Cell-type expression enrichment analysis (https://github.com/NathanSkene/EWCE); (viii) Power analysis (CaTS tool, https://csg.sph.umich.edu/abecasis/CaTS/; MKpower R-package, https://github.com/stamats/MKpower).

For manuscripts utilizing custom algorithms or software that are central to the research but not yet described in published literature, software must be made available to editors and reviewers. We strongly encourage code deposition in a community repository (e.g. GitHub). See the Nature Portfolio guidelines for submitting code & software for further information.

Data

Policy information about availability of data

All manuscripts must include a data availability statement. This statement should provide the following information, where applicable:

- Accession codes, unique identifiers, or web links for publicly available datasets
- A description of any restrictions on data availability
- For clinical datasets or third party data, please ensure that the statement adheres to our policy

The original DNA sequencing data supporting the conclusions of this article will be available in the China National Center for Bioinformation/Beijing Institute of Genomics, Chinese Academy of Sciences (GSA-Human: HRA005774) that are publicly accessible at https://ngdc.cncb.ac.cn/gsa-human. Public single-cell transcriptome data have been deposited by the authors under accession code/website (Human brain: GSE178265; Macaque brain: https://db.cngb.org/mba/download; Mouse brain: GSE60361). GWAS statistical summary of SVs/CNVs/STRs were released at GitHub (https://github.com/GWASummary/PD-GWAS-SV). All other data are available for researchers who meet criteria for access to these data from the corresponding author.

Research involving human participants, their data, or biological material

Policy information about studies with human participants or human data. See also policy information about sex, gender (identity/presentation), and sexual orientation and race, ethnicity and racism.

Reporting on sex and gender	Sex was used to reflect the biological attribute at birth as participants reported or ID card record.
Reporting on race, ethnicity, or other socially relevant groupings	Participants involved in the study are Chinese Han population.
Population characteristics	The study included the discovery and the external validation cohorts. The discovery cohort consists of 466 PD patients and 513 healthy elderlies from the Northern and Southern Chinese provinces. The average age of PD patients and healthy controls were 61.3±8.5 and 71.8±6.7 years, respectively. The external validation cohort consists of 352 cases and 547 controls, which were aged 62.3±9.3 and 69.6±5.2 years, respectively.
Recruitment	The cases were diagnosed by movement disorders specialists using MDS clinical diagnostic criteria for Parkinson's disease, and those with a family history in a first or second-degree relative were excluded. Control subjects were selected from the community cohorts of the Beijing Longitudinal Study on Aging II (BLSA II) and were sex- and age-matched with the PD cases.
Ethics oversight	The collection and use of patient samples was approved by the Institutional Review Board of Xuanwu Hospital of Capital Medical University. The study was approved by The China Human Genetic Resource Administration Office (China Ministry of Science and Technology Genetics Audit: [2022] CJ1262. Informed consent was obtained from all patients or their guardians.

Note that full information on the approval of the study protocol must also be provided in the manuscript.

Field-specific reporting

Please select the one below that is the best fit for your research. If you are not sure, read the appropriate sections before making your selection.

☒ Life sciences ☐ Behavioural & social sciences ☐ Ecological, evolutionary & environmental sciences

For a reference copy of the document with all sections, see nature.com/documents/nr-reporting-summary-flat.pdf

Life sciences study design

All studies must disclose on these points even when the disclosure is negative.

Sample size	The discovery cohort included 466 PD cases and 513 controls.
Data exclusions	Low quality sequencing reads and variants were excluded. The filter criteria and threshold were described for read alignment and each variant type at Methods.
Replication	The replication study included 352 cases and 547 controls.
Randomization	The cases were diagnosed by movement disorders specialists using MDS clinical diagnostic criteria for Parkinson's disease, and those with a family history in a first or second-degree relative were excluded. Control subjects were selected from the community cohorts of the Beijing Longitudinal Study on Aging II (BLSA II) and were gender- and age-matched with PD cases.
Blinding	Whole genome sequencing and variant calling were applied at cases and controls with the same parameters.

Reporting for specific materials, systems and methods

We require information from authors about some types of materials, experimental systems and methods used in many studies. Here, indicate whether each material, system or method listed is relevant to your study. If you are not sure if a list item applies to your research, read the appropriate section before selecting a response.

Materials & experimental systems

n/a	Involved in the study
☒	☐ Antibodies
☒	☐ Eukaryotic cell lines
☒	☐ Palaeontology and archaeology
☒	☐ Animals and other organisms
☒	☐ Clinical data
☒	☐ Dual use research of concern
☒	☐ Plants

Methods

n/a	Involved in the study
☒	☐ ChIP-seq
☒	☐ Flow cytometry
☒	☐ MRI-based neuroimaging

Plants

Seed stocks	NA
Novel plant genotypes	NA
Authentication	NA
