# Supplementary material for: High-depth whole-genome sequencing identifies structure variants, copy number variants and short tandem repeats associated with Parkinson’s disease
Source: NPJ Parkinsons Dis. 2024 Jul 23;10:134. doi: 10.1038/s41531-024-00722-1 (PMC11266557; doi:10.1038/s41531-024-00722-1)

CHM1

Short−reads

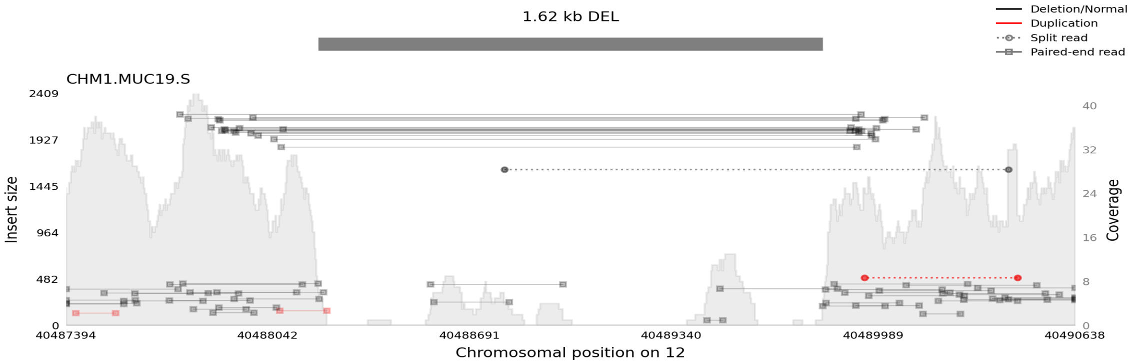

CHM1

Long−reads

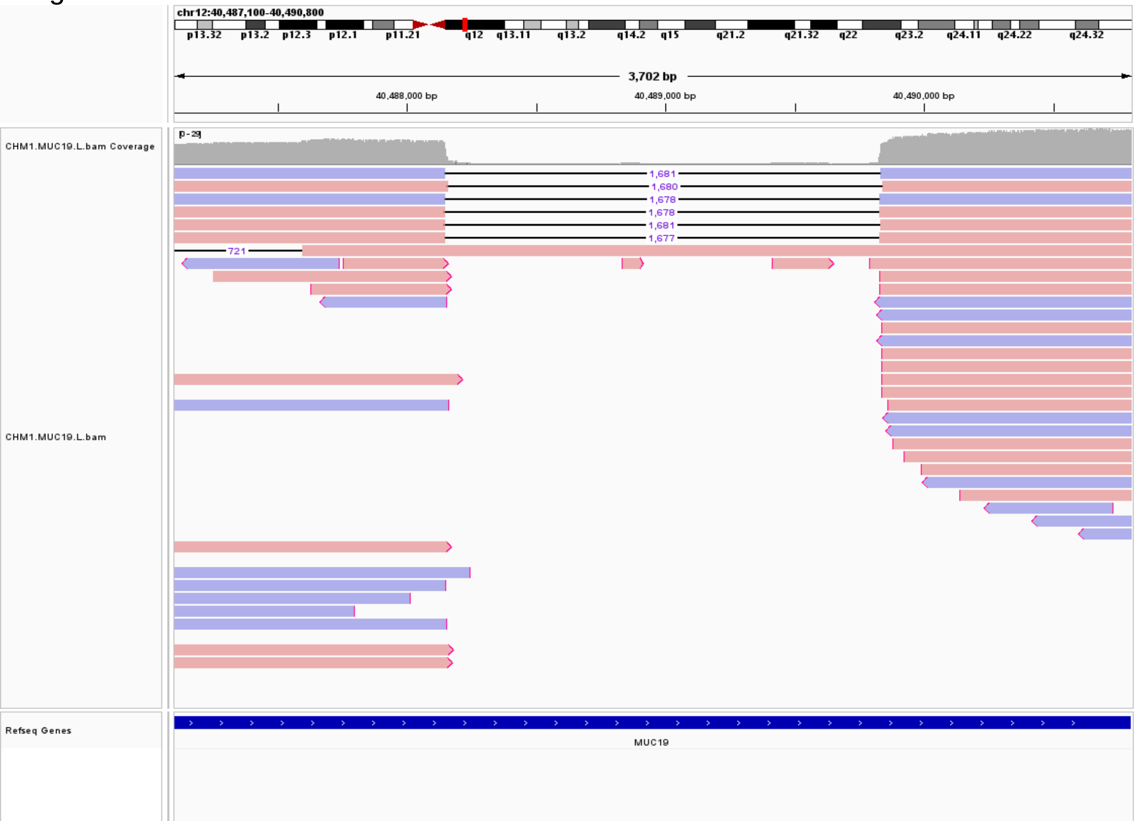

CHM13

Short−reads

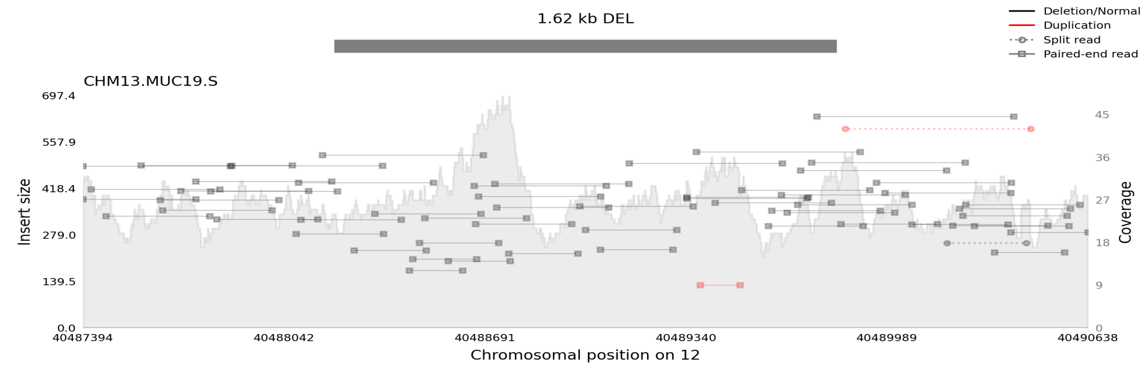

CHM13

Long−reads

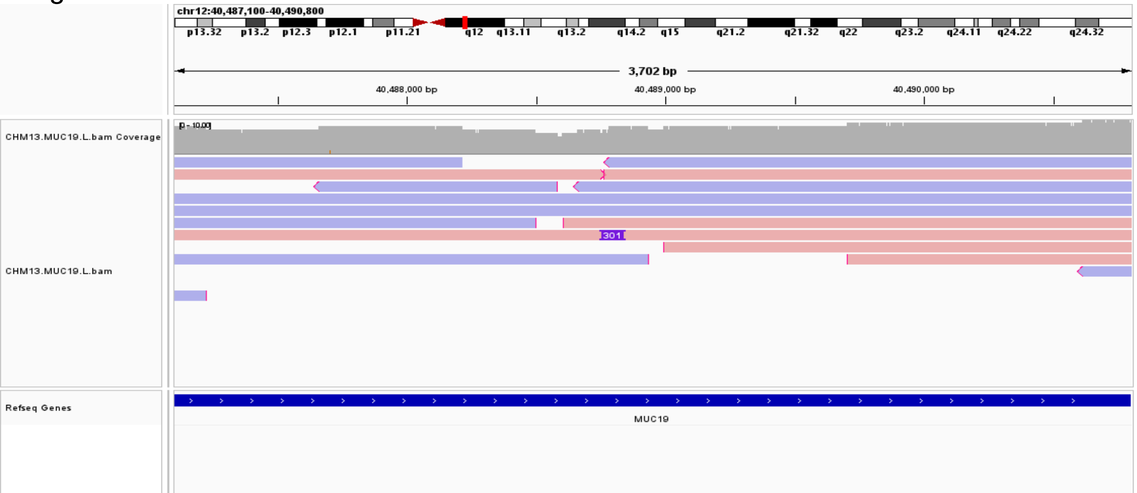

HG00099

Short−reads

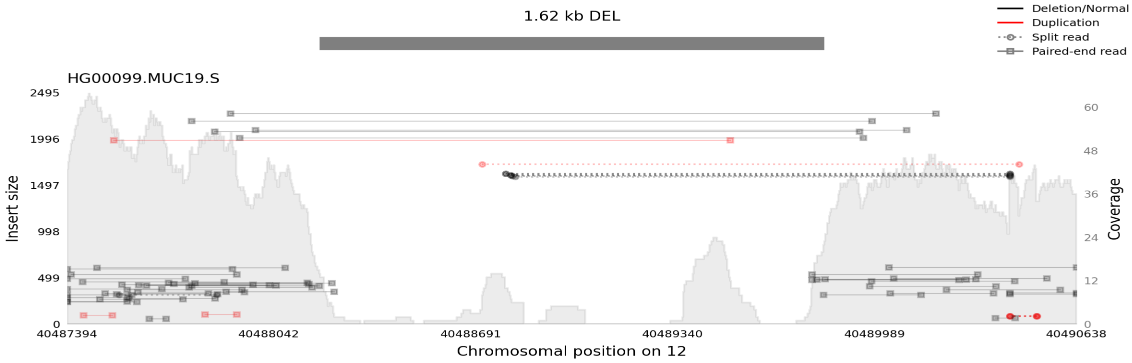

HG00099

Long−reads

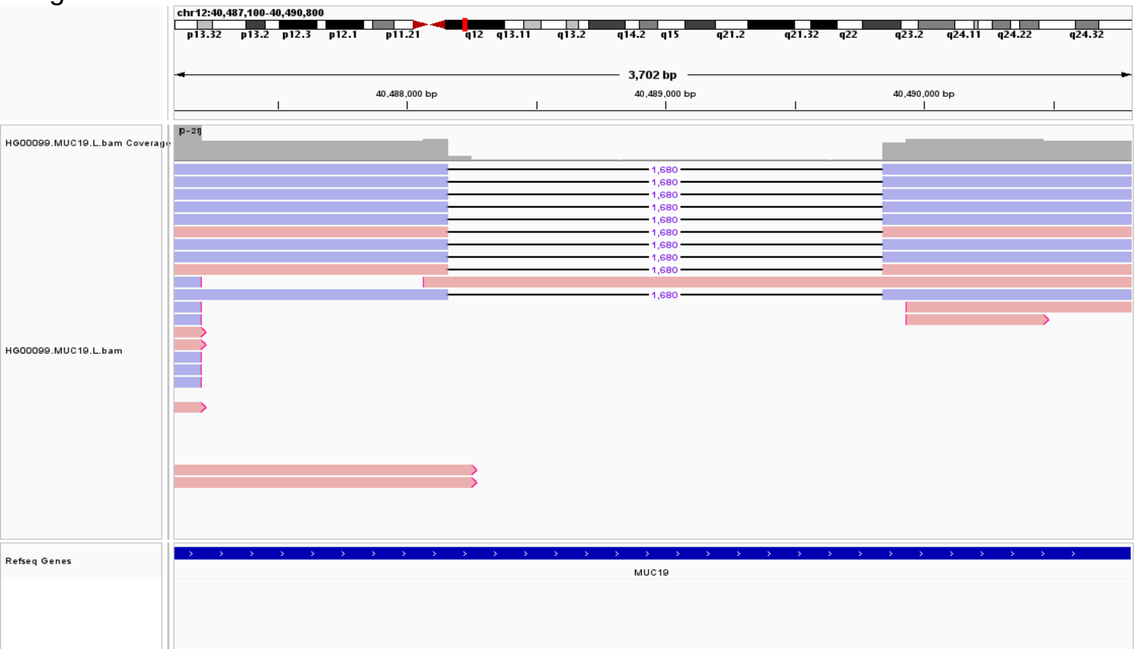

HG00140

Short−reads

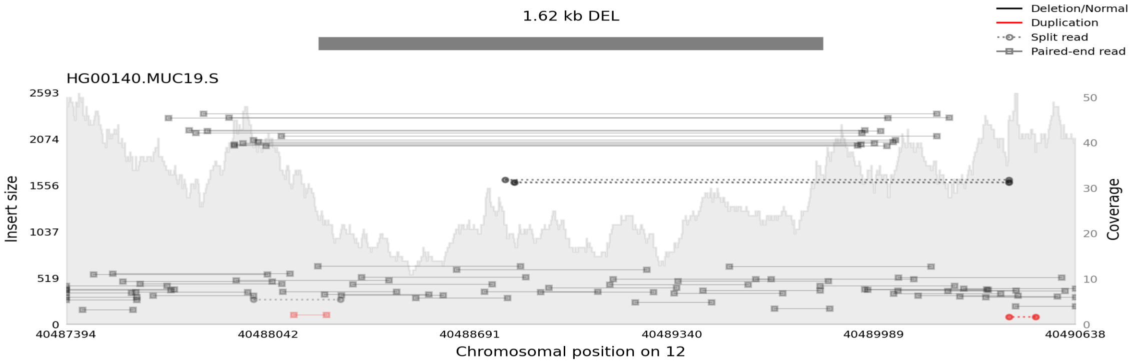

HG00140

Long−reads

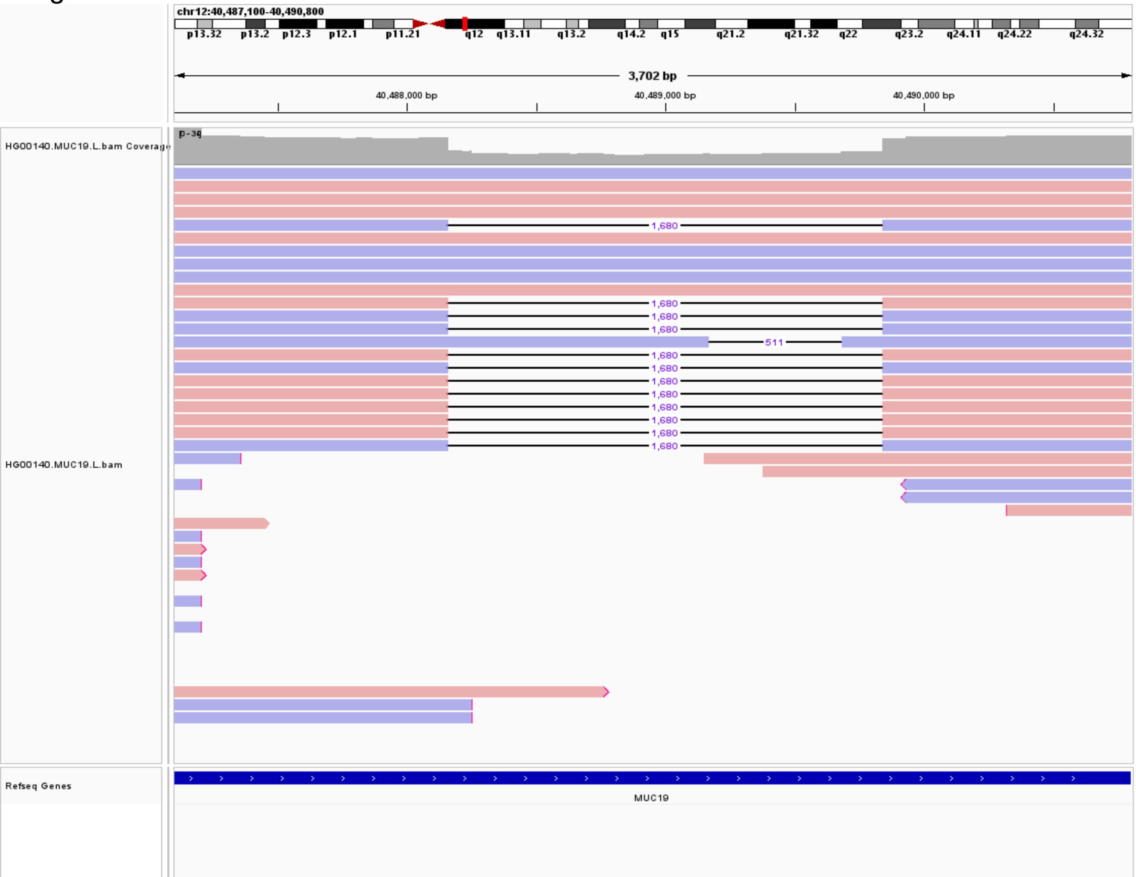

HG002

Short−reads

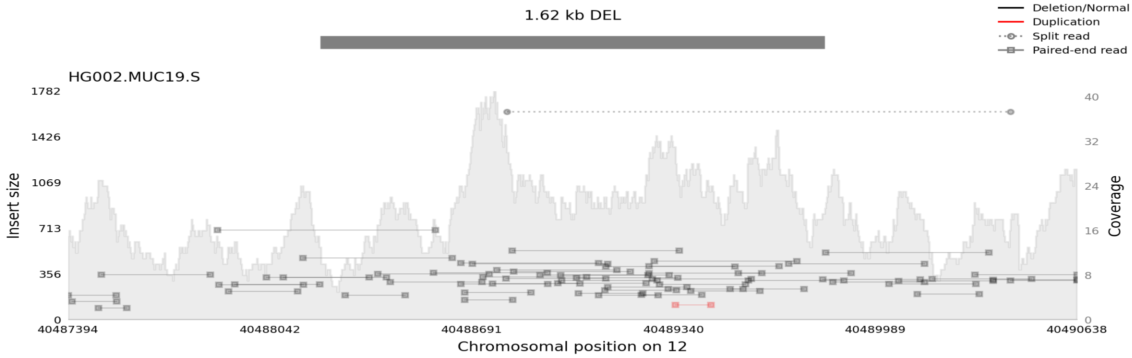

HG002

Long−reads

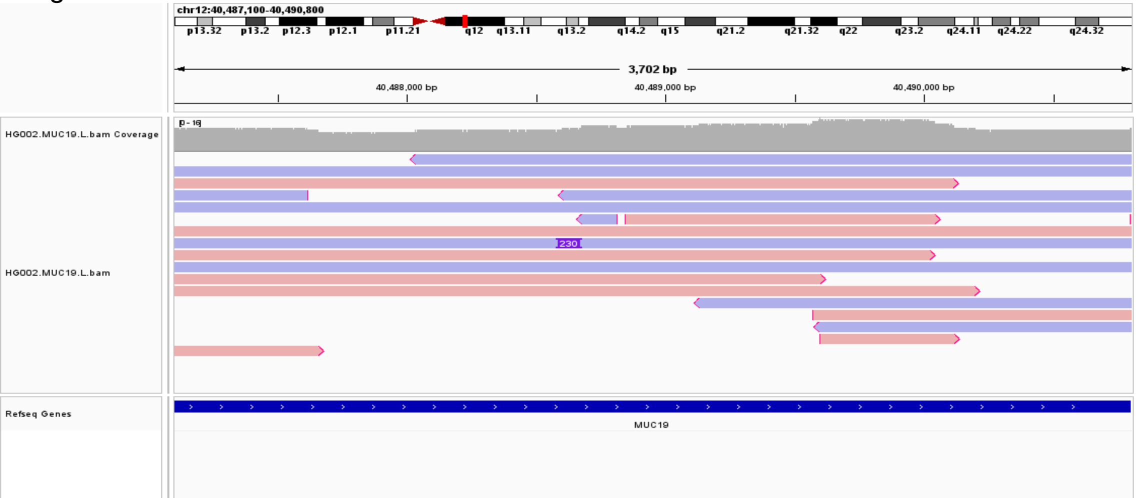

HG00268

Short−reads

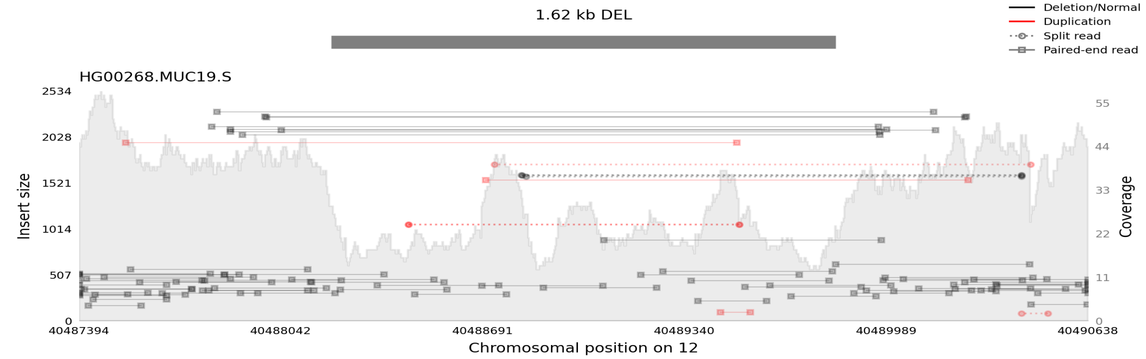

HG00268

Long−reads

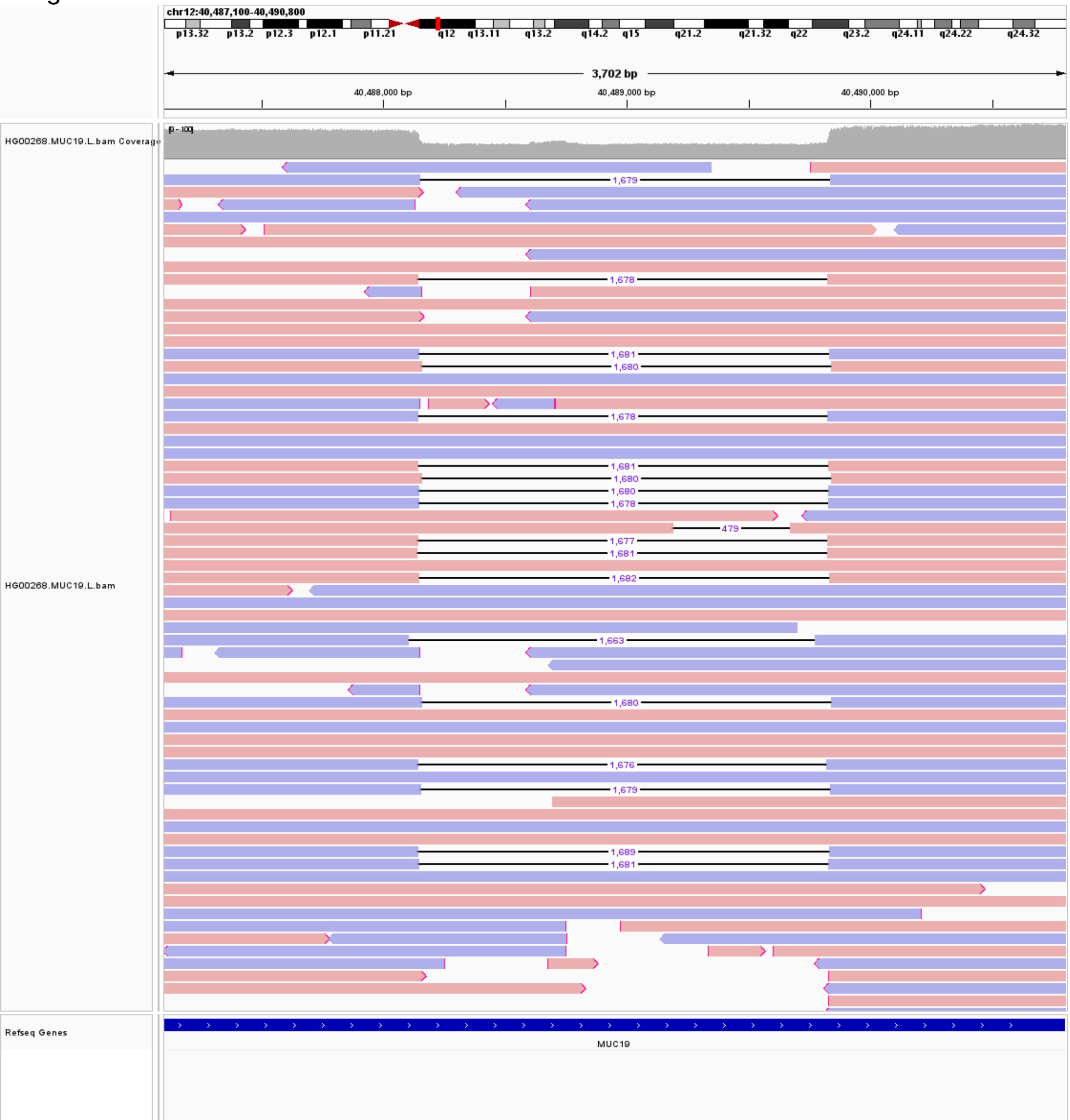

HG00280

Short−reads

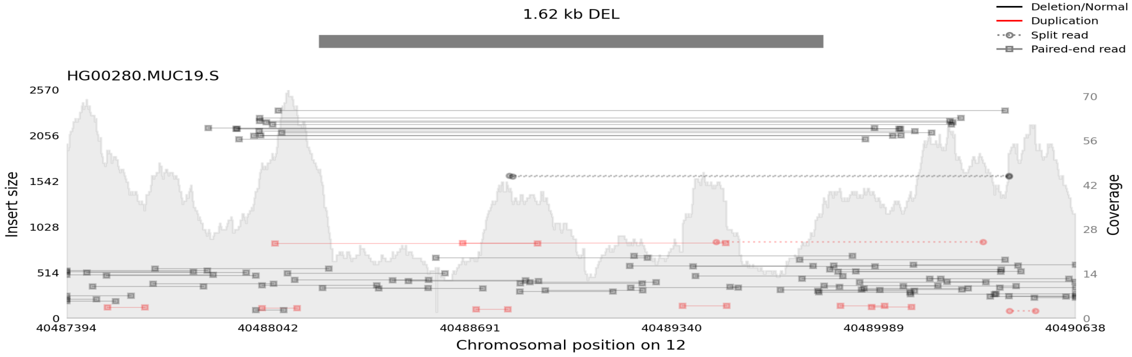

HG00280

Long−reads

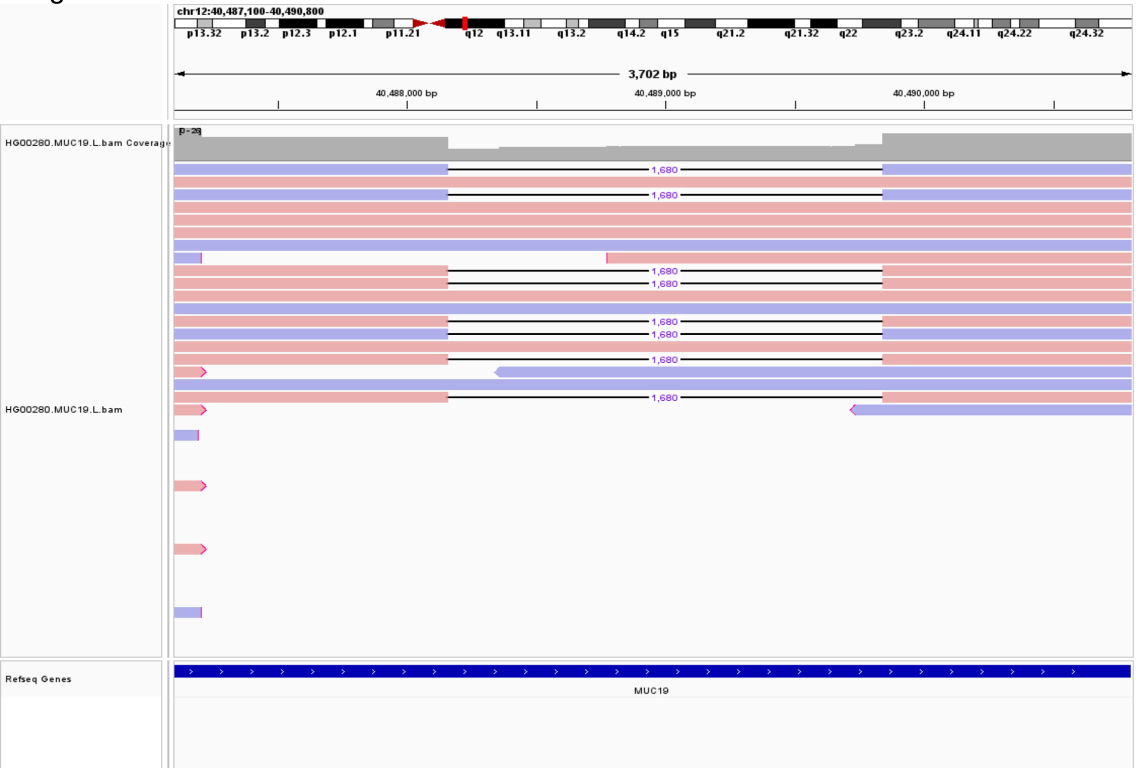

HG003

Short−reads

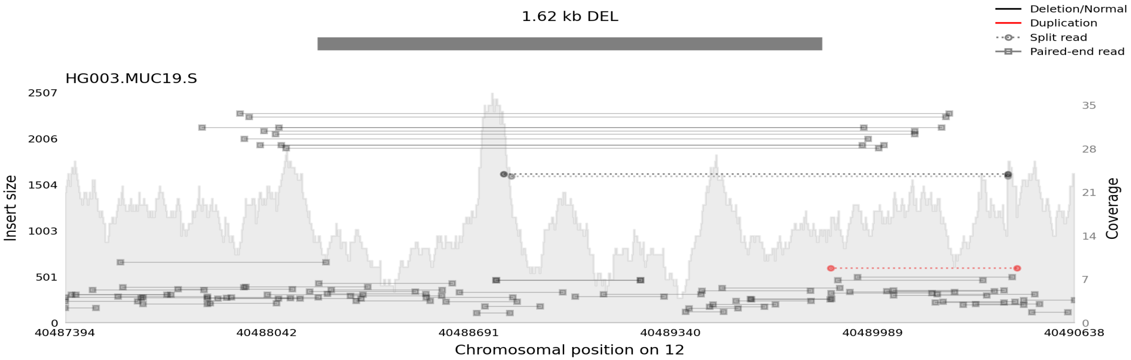

HG003

Long−reads

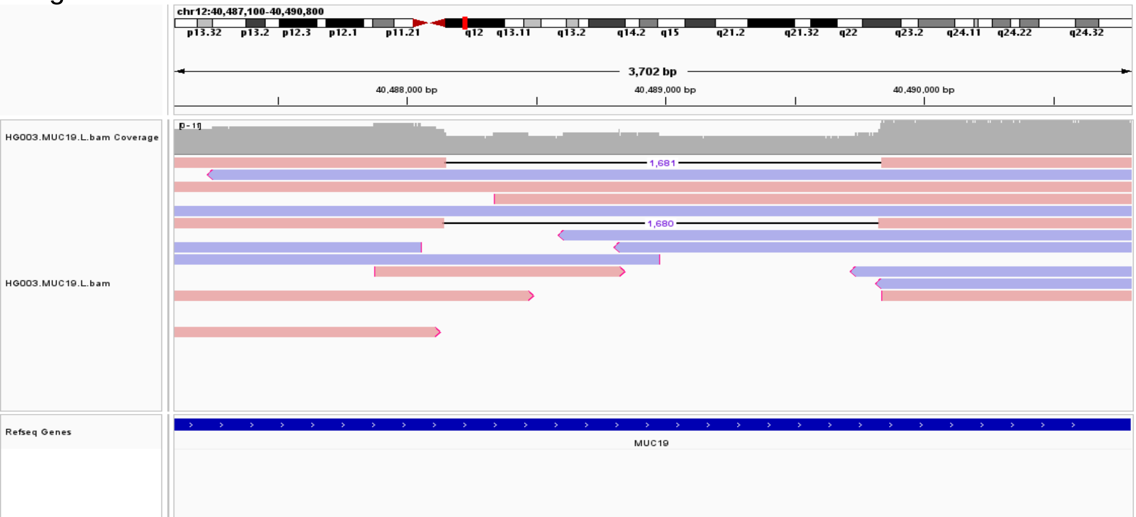

HG00323

Short−reads

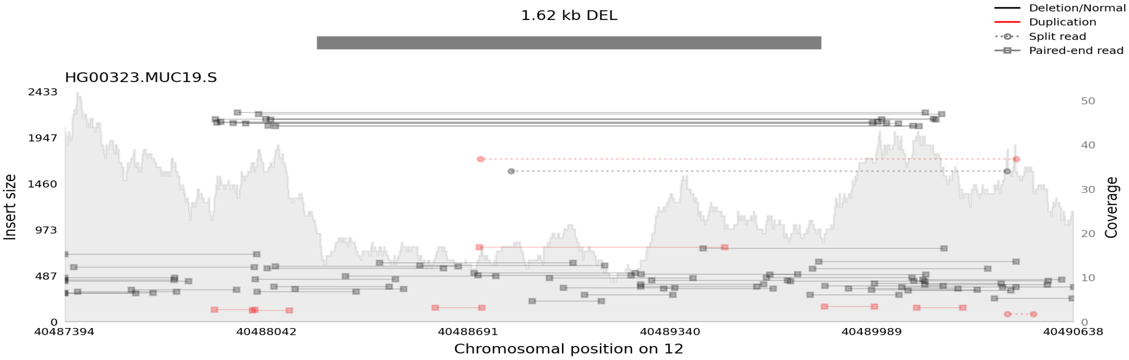

HG00323

Long−reads

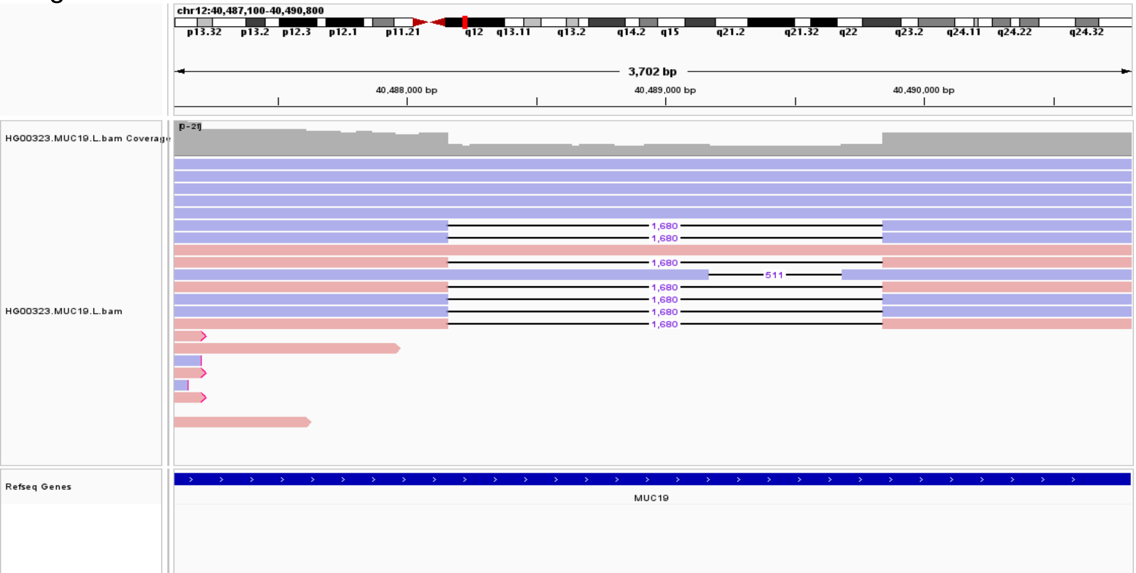

HG004

Short−reads

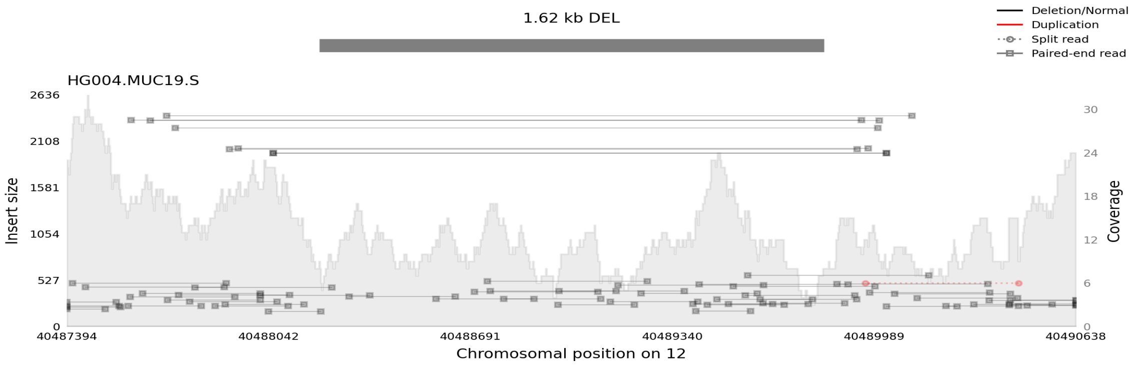

HG004

Long−reads

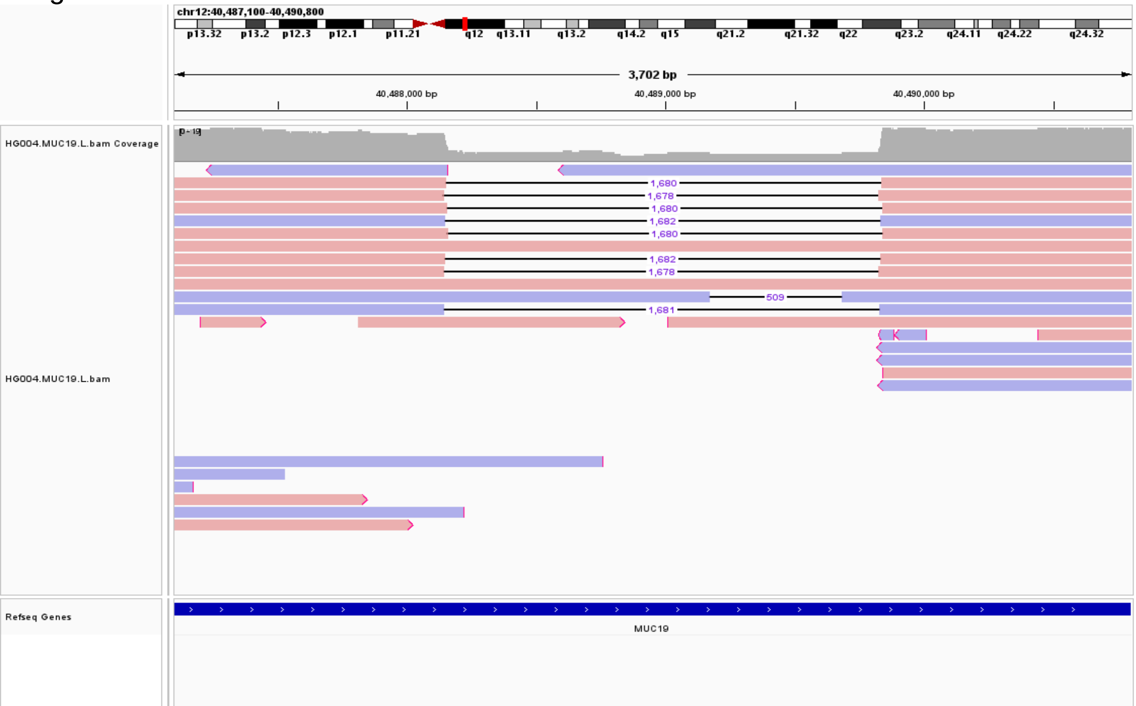

HG00408
Short−reads

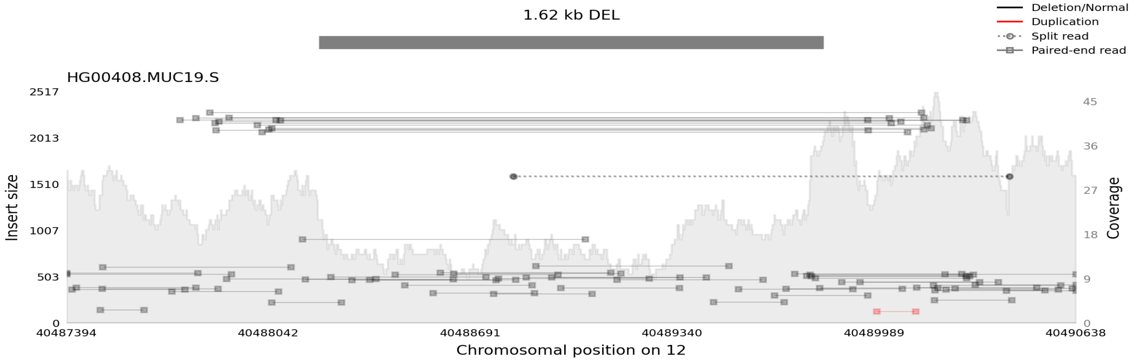

HG00408
Long−reads

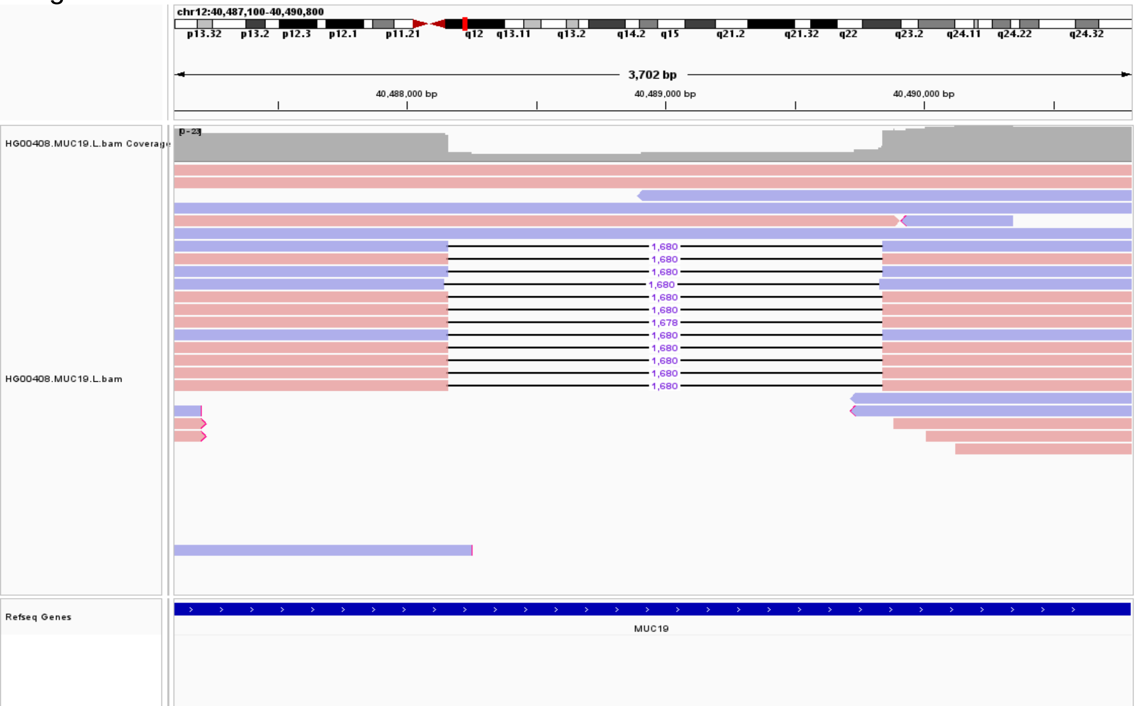

HG00423

Short−reads

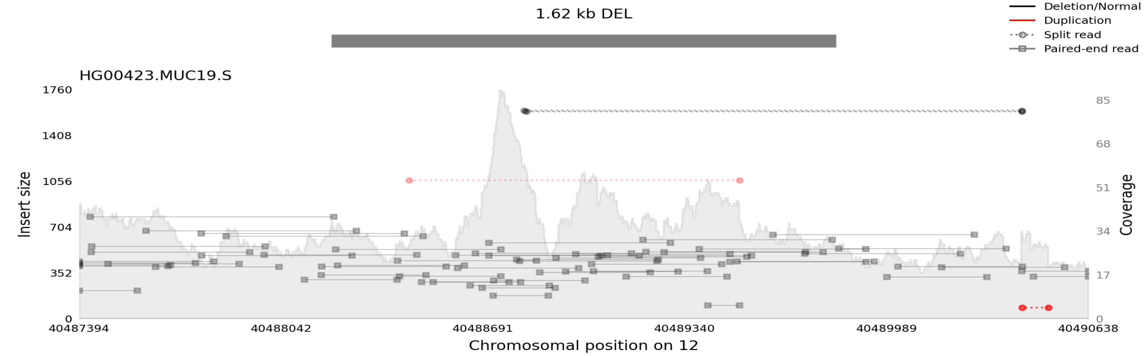

HG00423

Long−reads

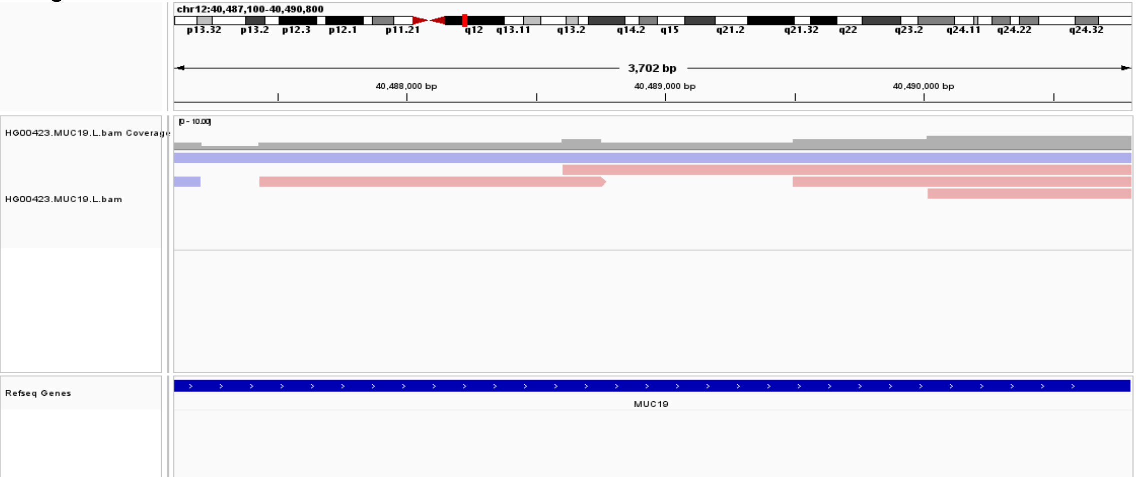

HG005

Short−reads

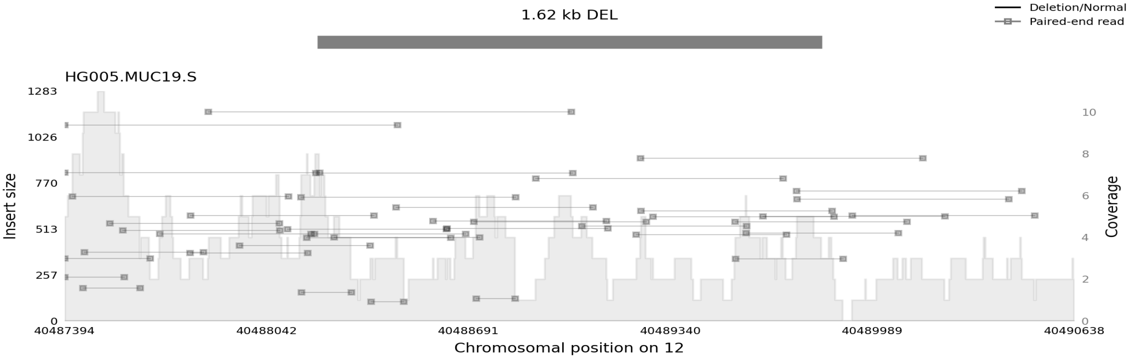

HG005

Long−reads

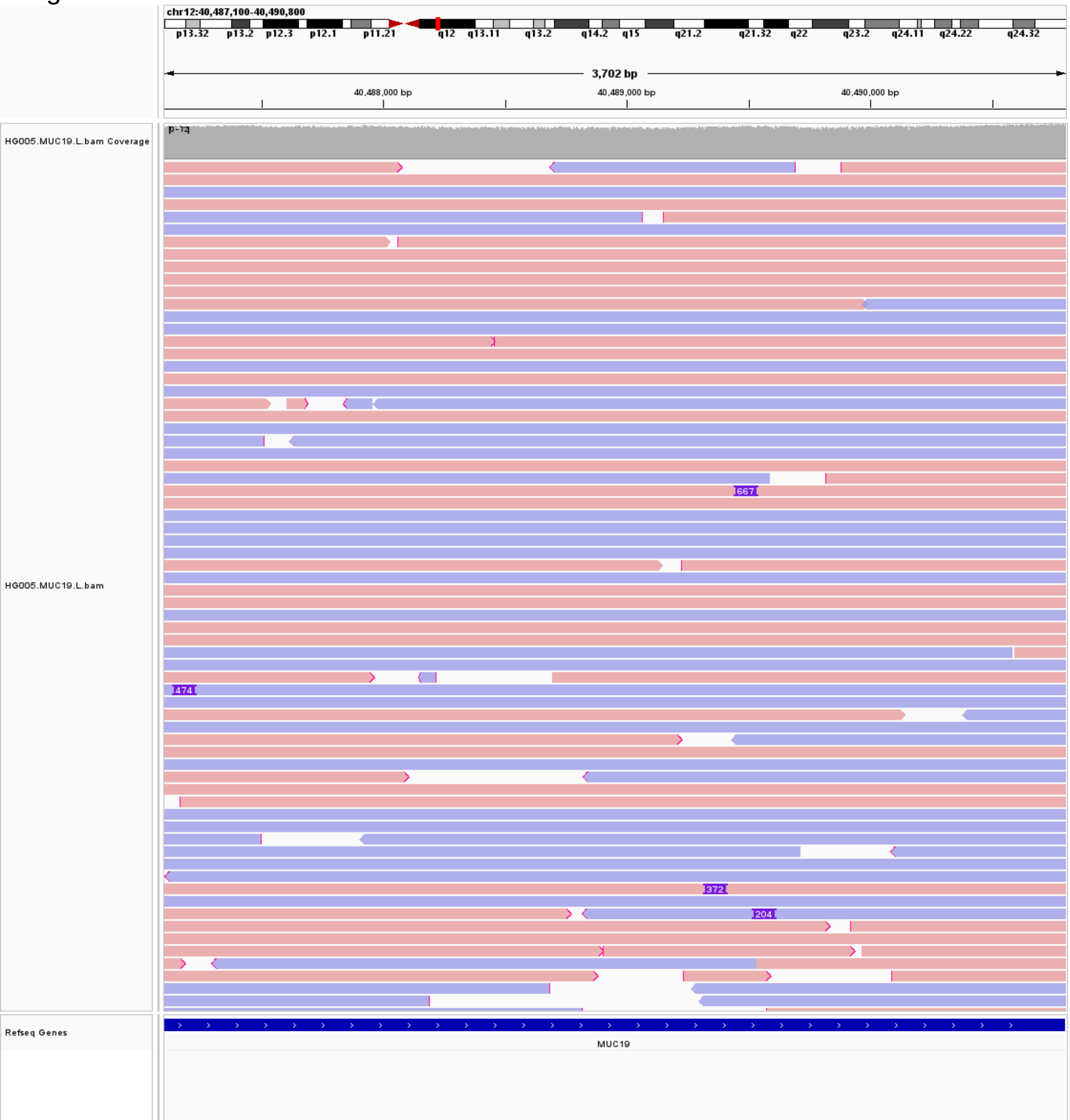

HG00512

Short−reads

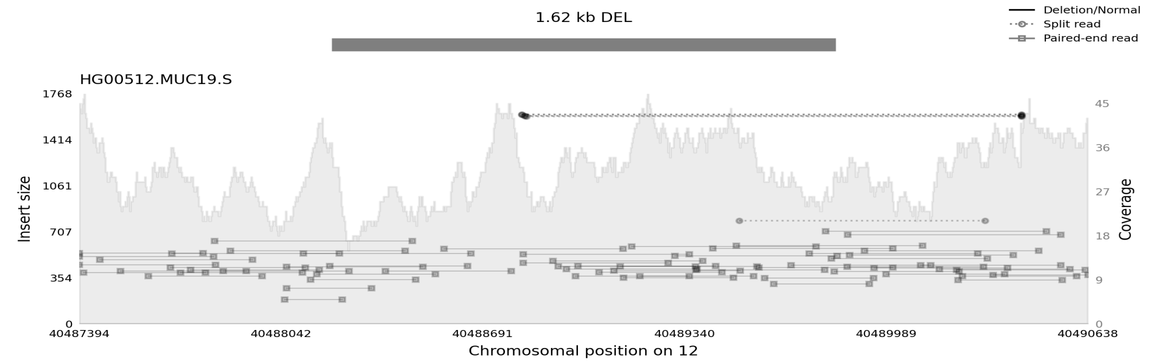

HG00512

Long−reads

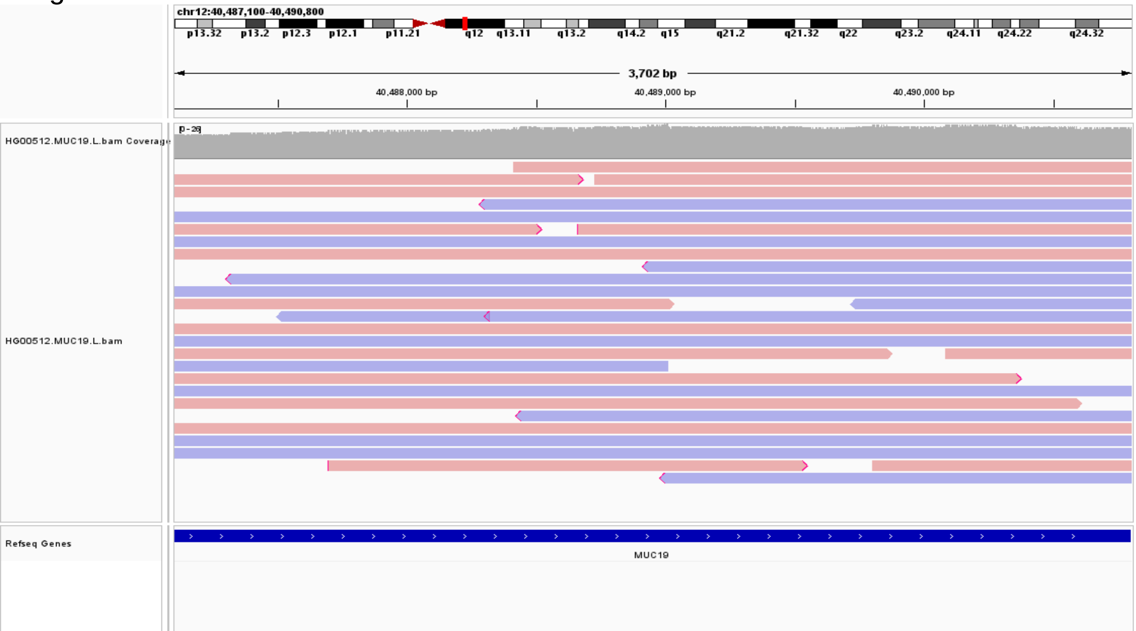

HG00513

Short−reads

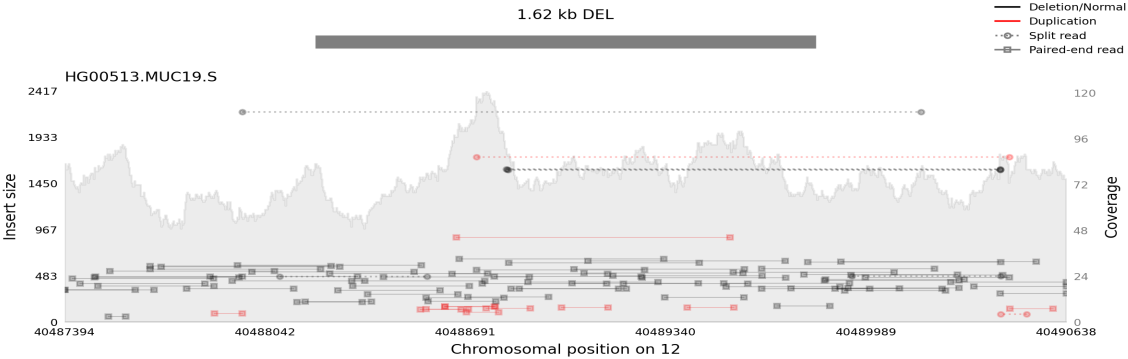

HG00513

Long−reads

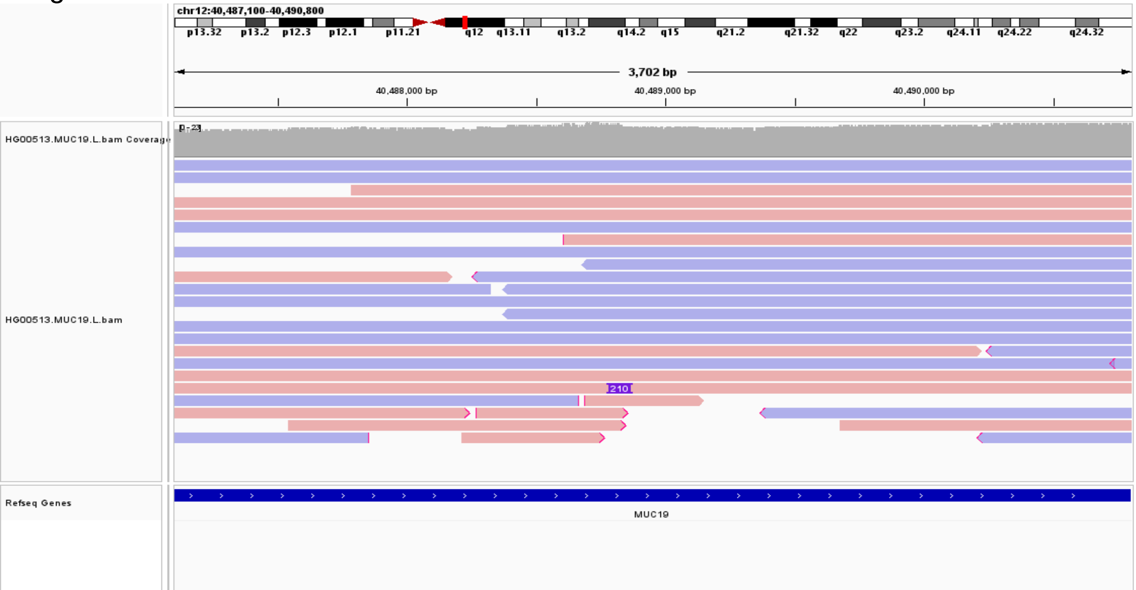

HG00514
Short−reads

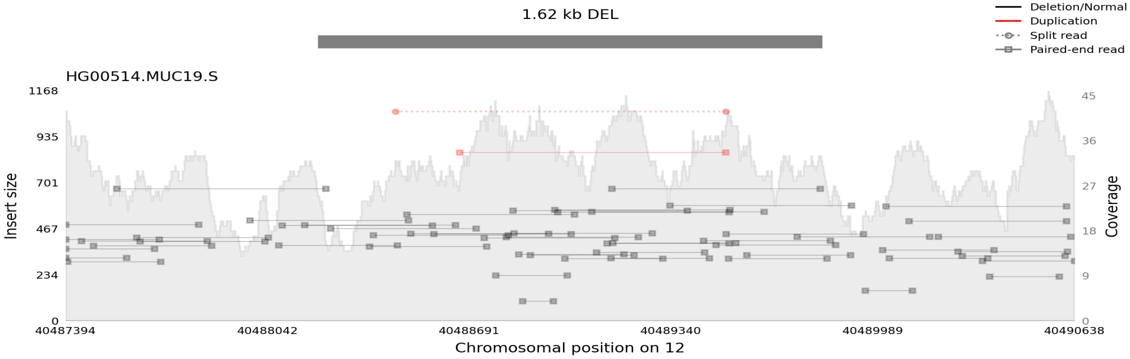

HG00514
Long−reads

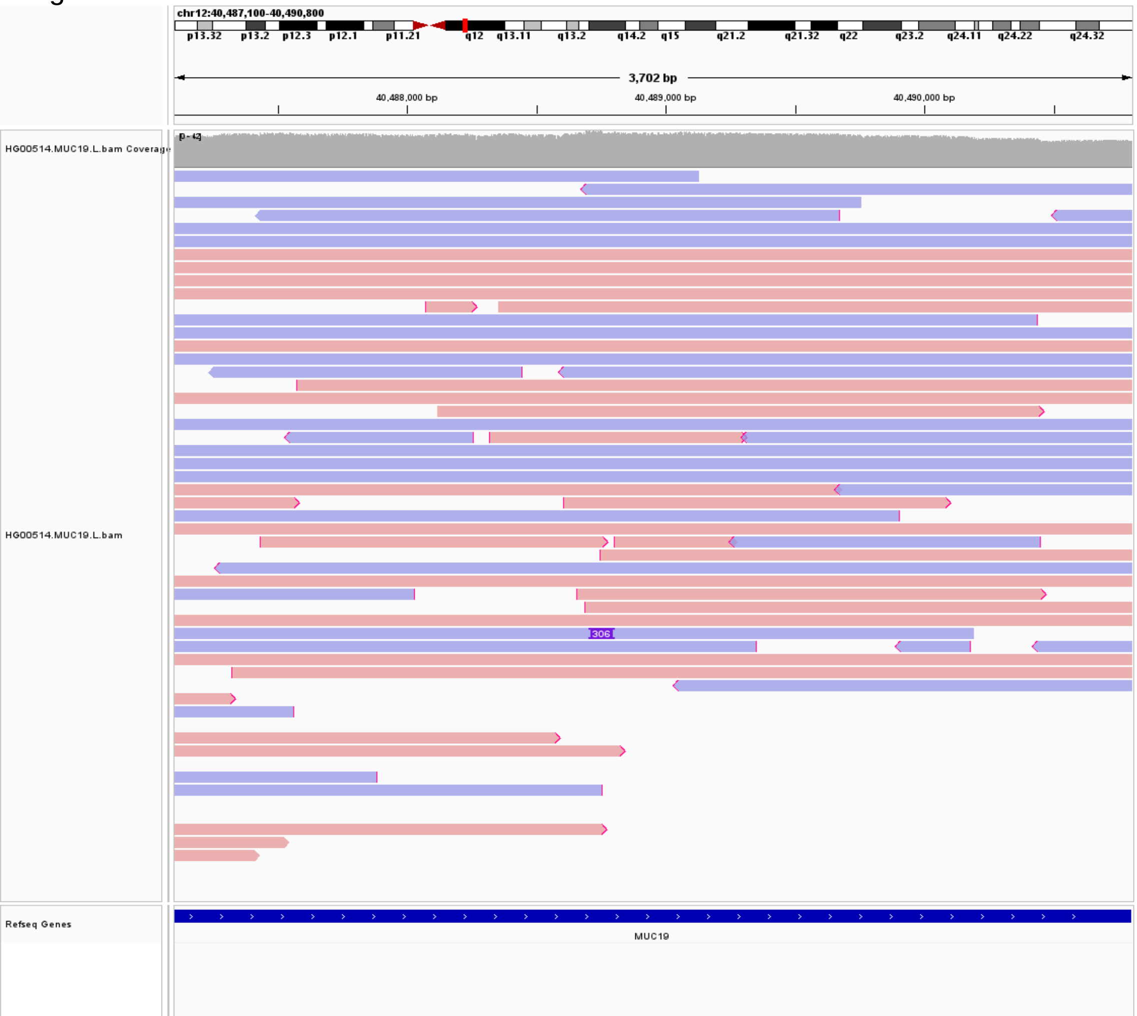

HG00544

Short−reads

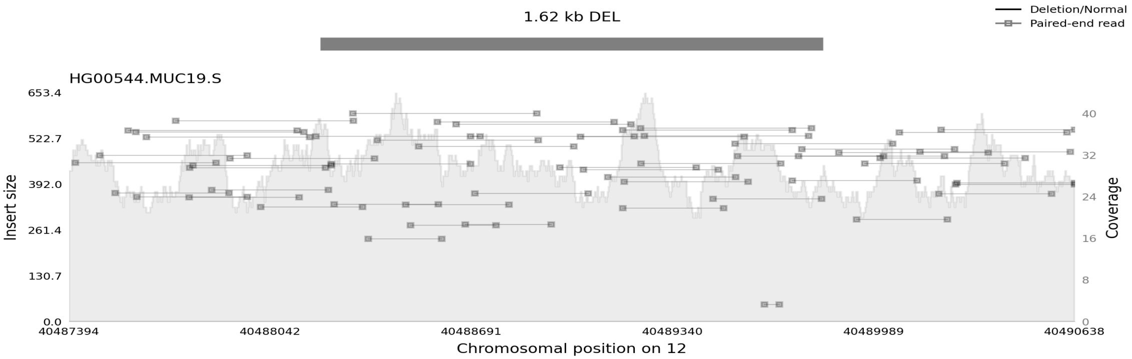

HG00544

Long−reads

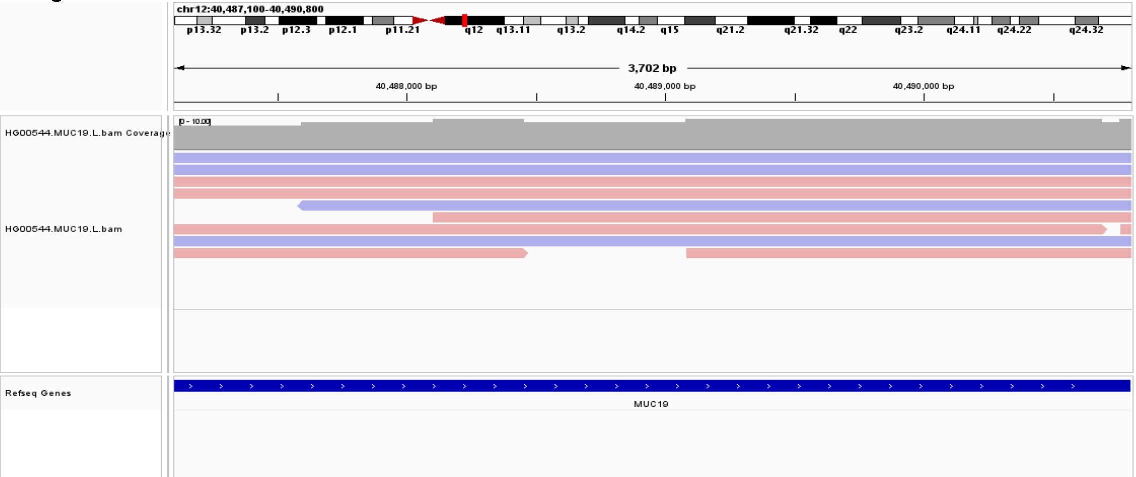

HG00558

Short−reads

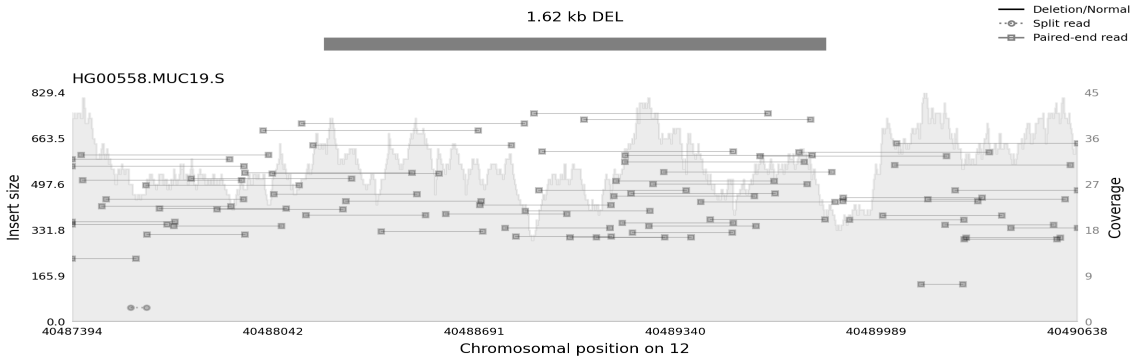

HG00558

Long−reads

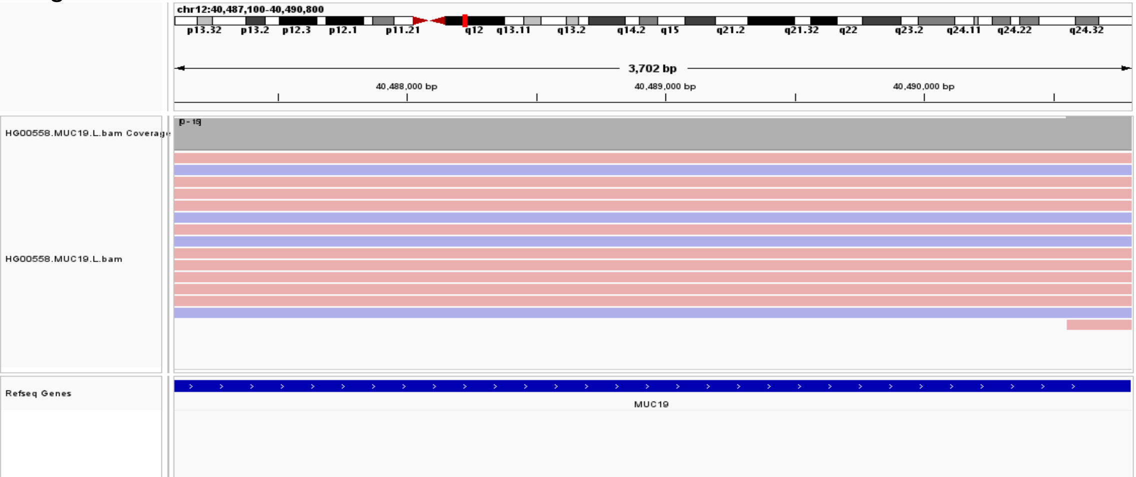

HG00597

Short−reads

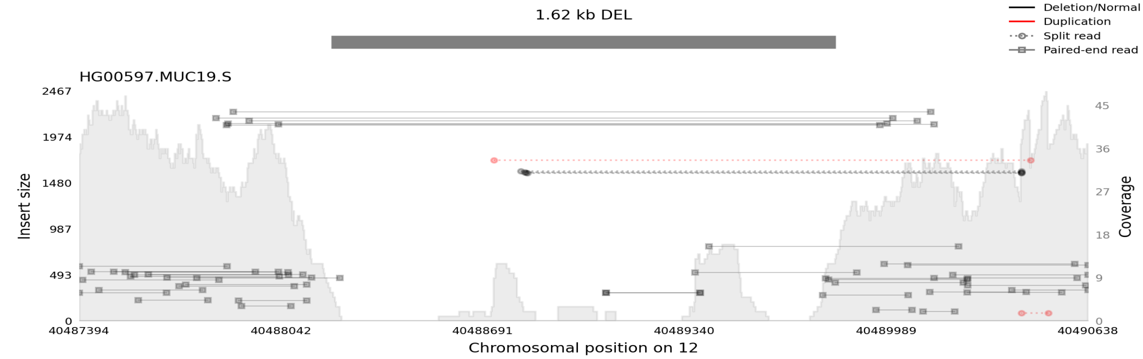

HG00597

Long−reads

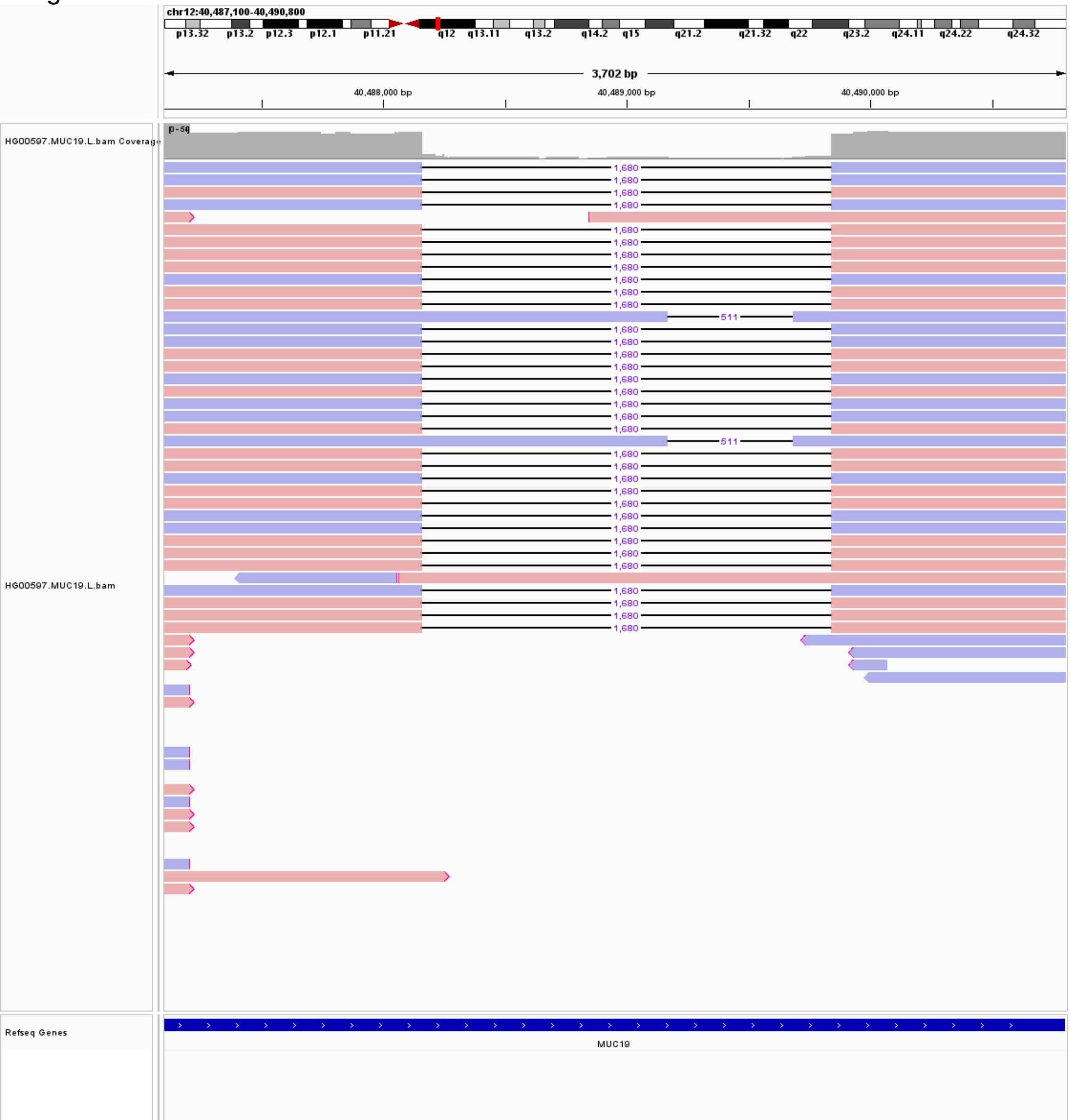

HG006
Short−reads

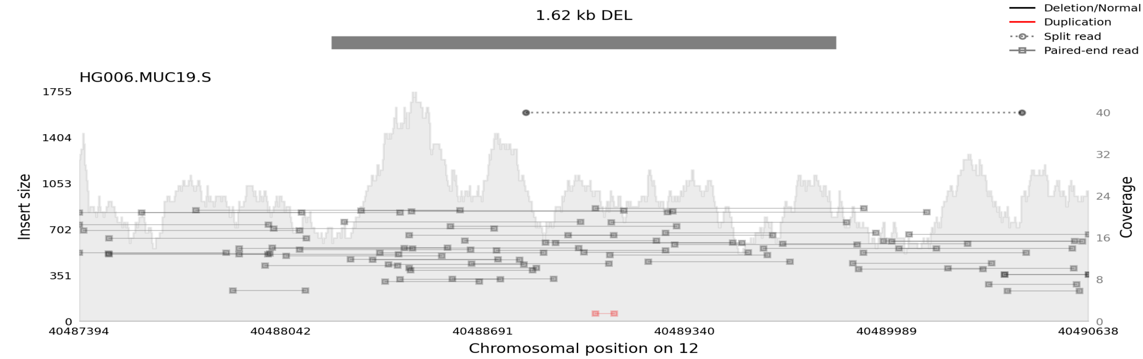

HG006
Long−reads

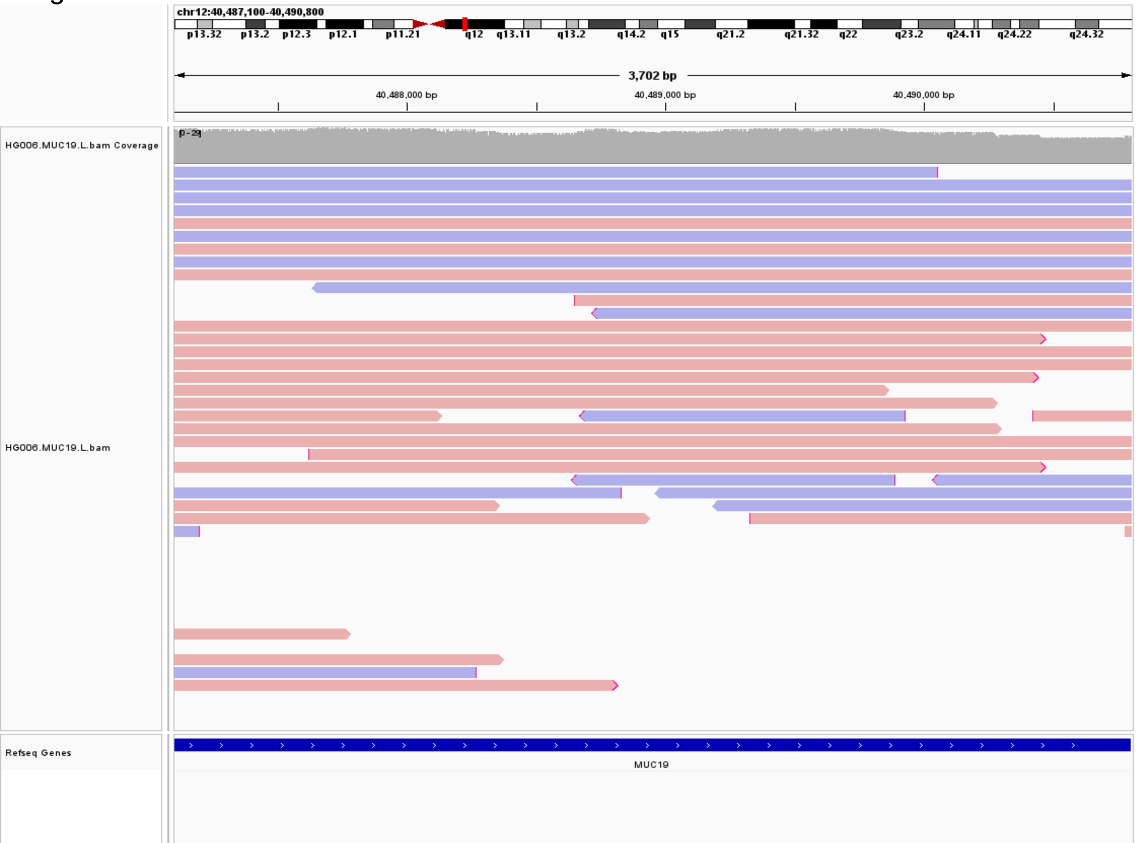

HG00609

Short−reads

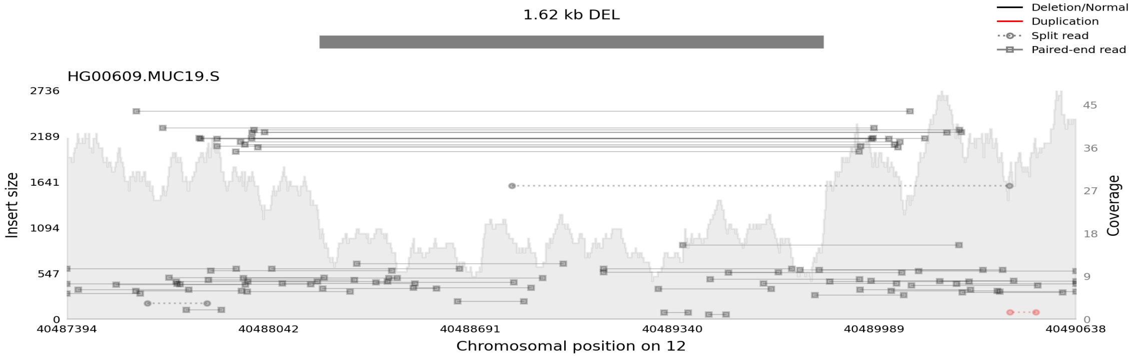

HG00609

Long−reads

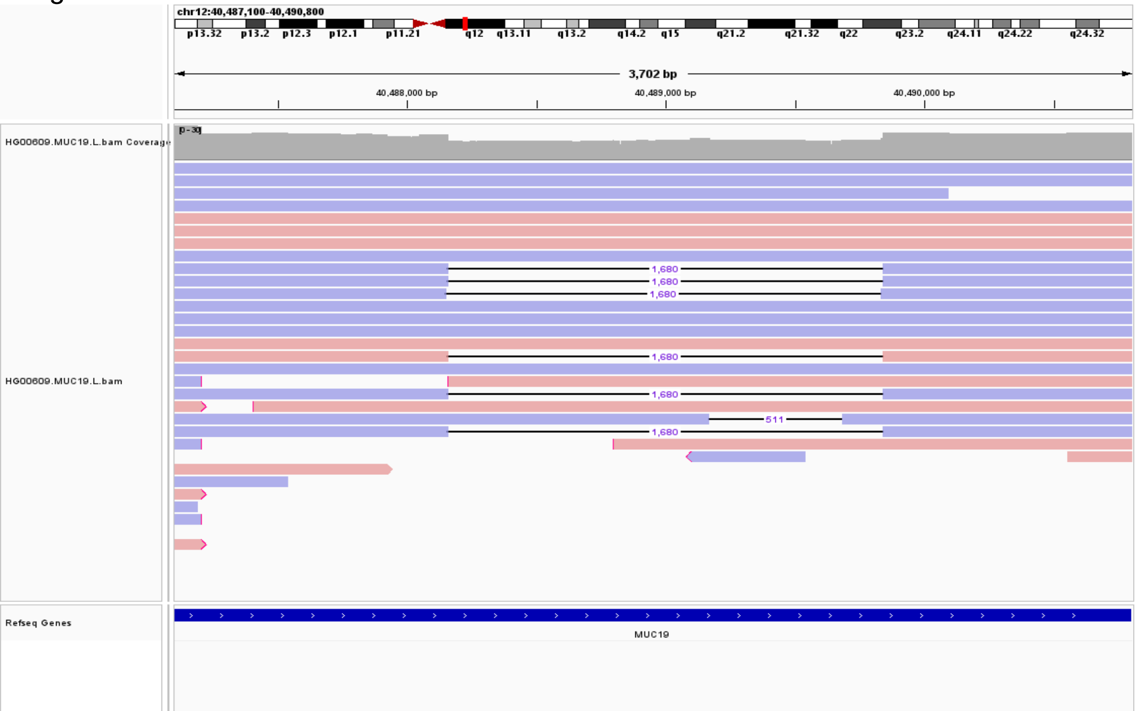

HG00621
Short−reads

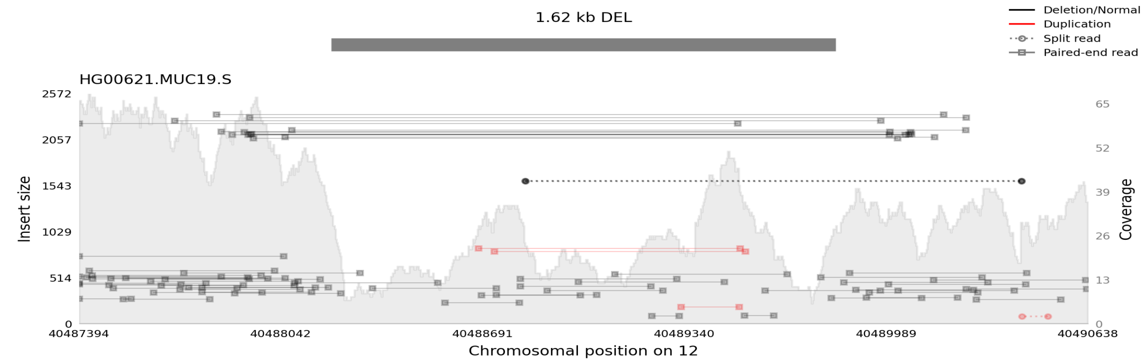

HG00621
Long−reads

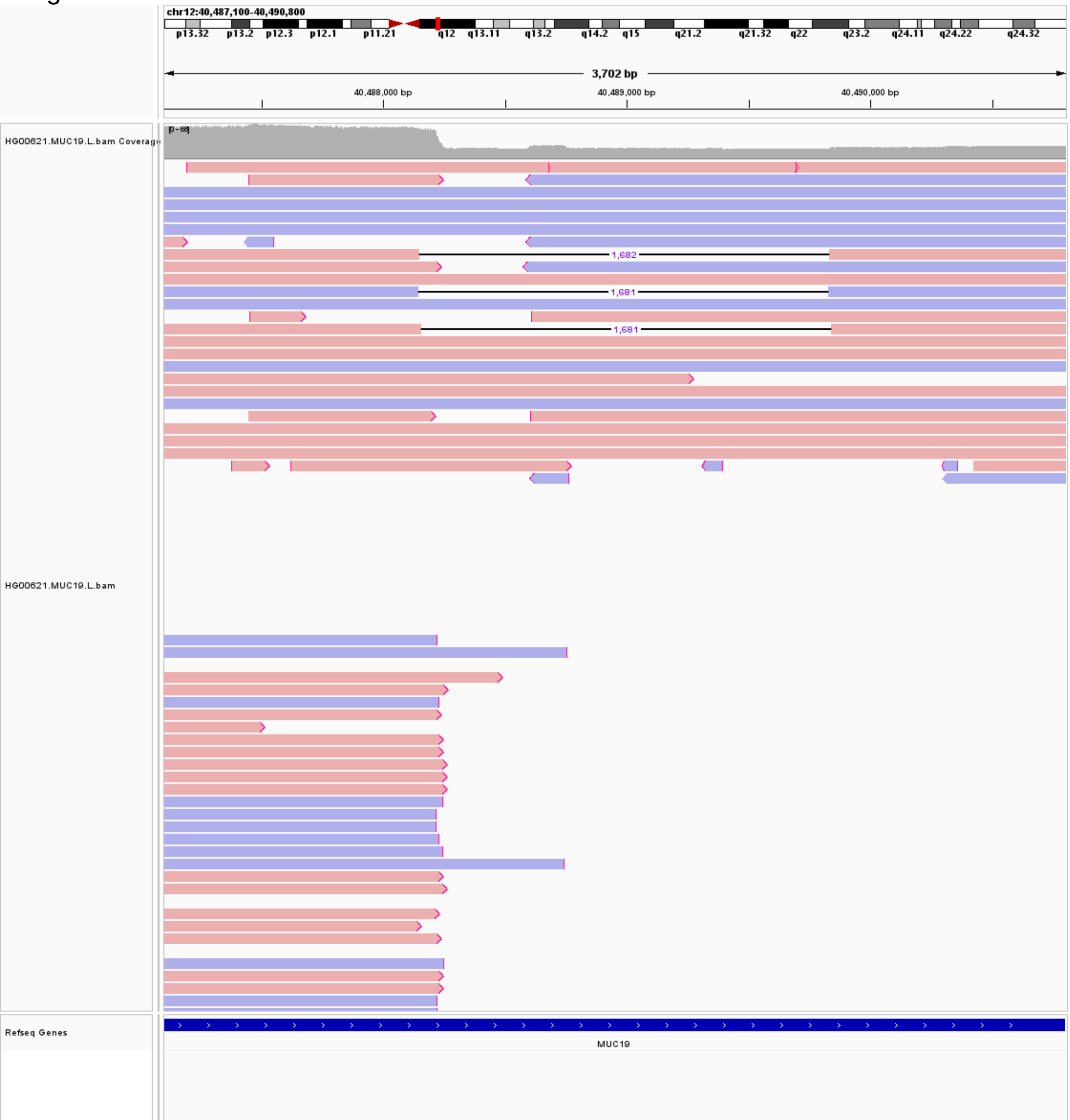

HG00639

Short−reads

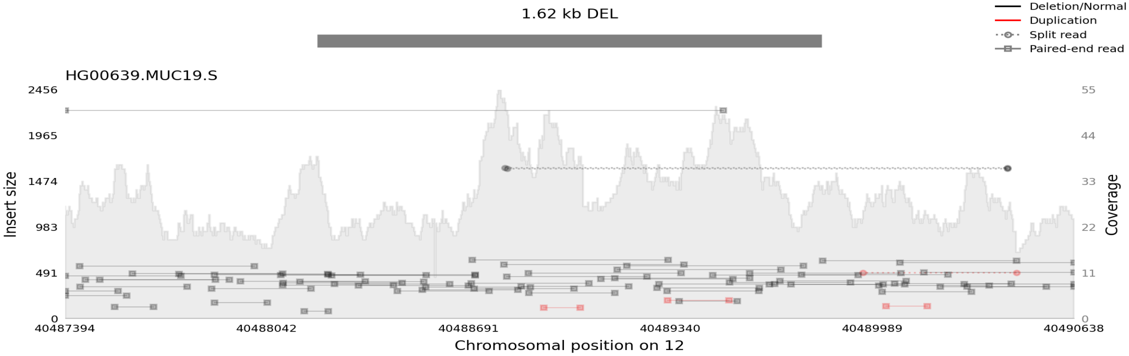

HG00639

Long−reads

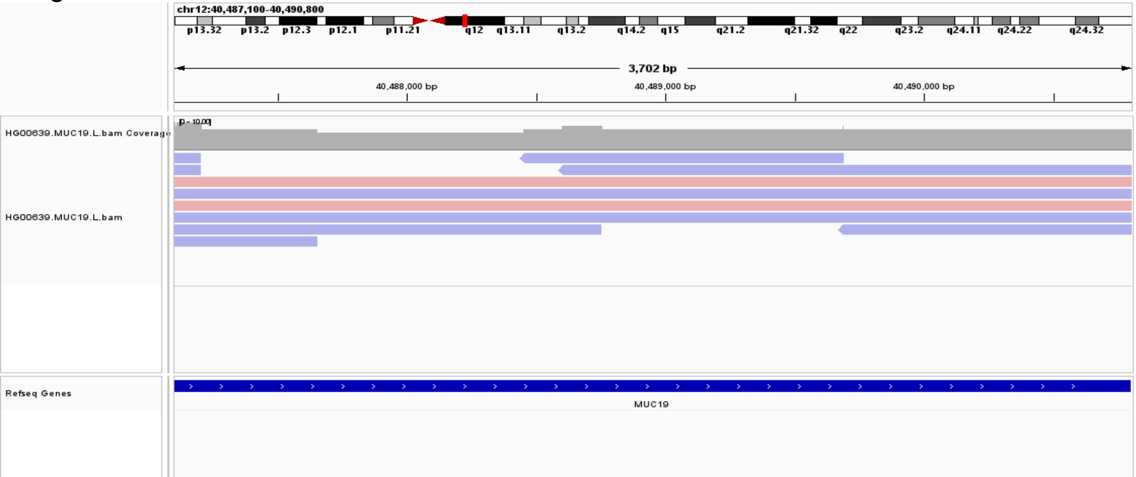

HG00642

Short−reads

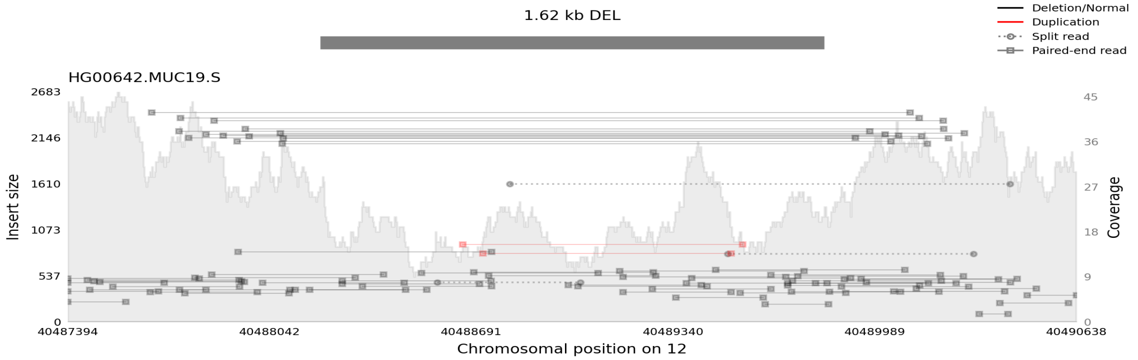

HG00642

Long−reads

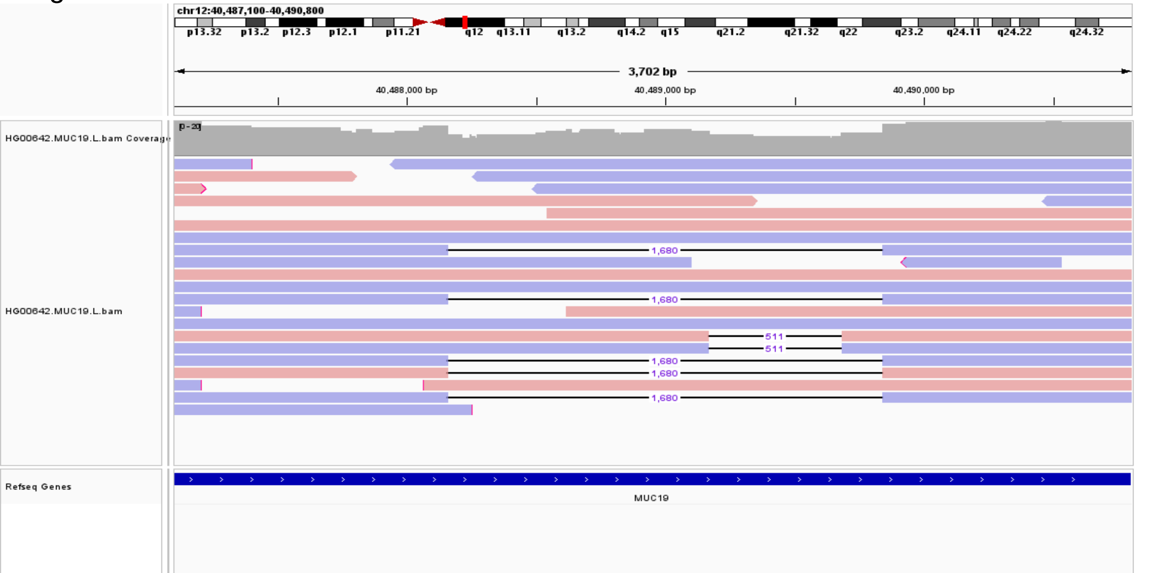

HG00658

Short−reads

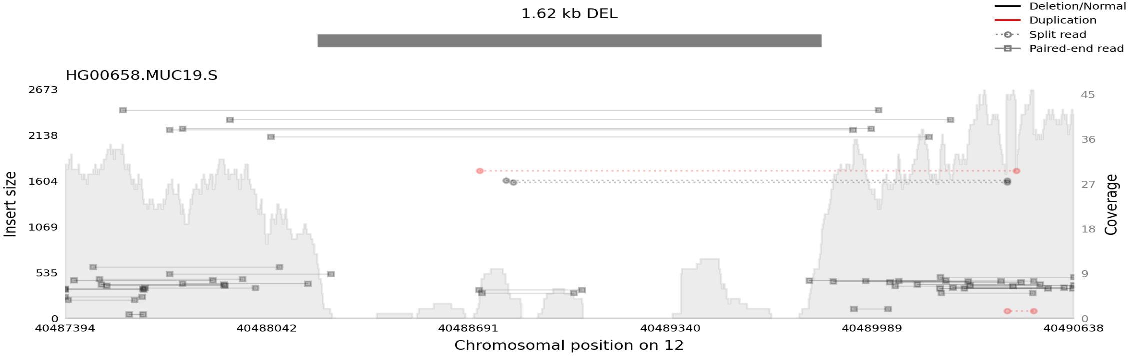

HG00658

Long−reads

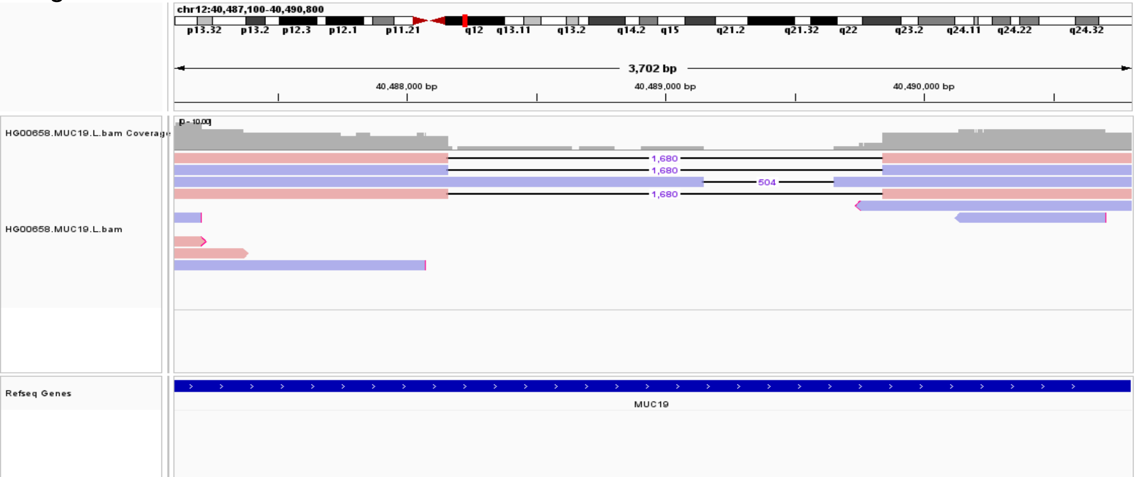

HG007

Short−reads

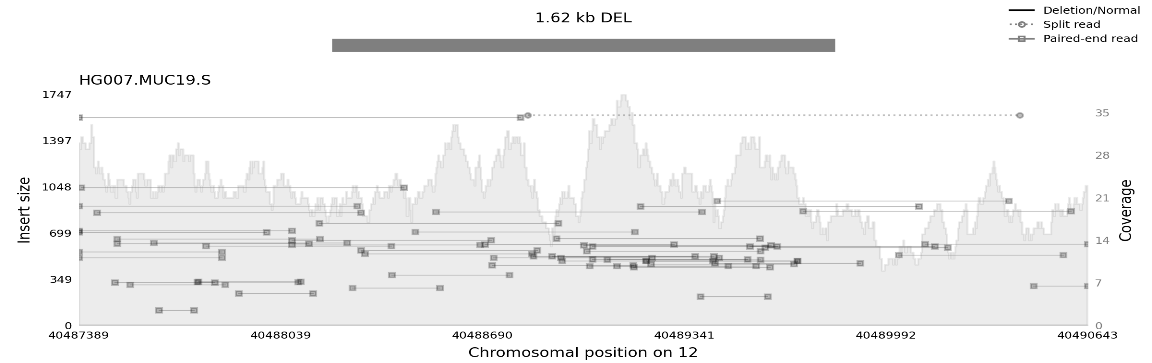

HG007

Long−reads

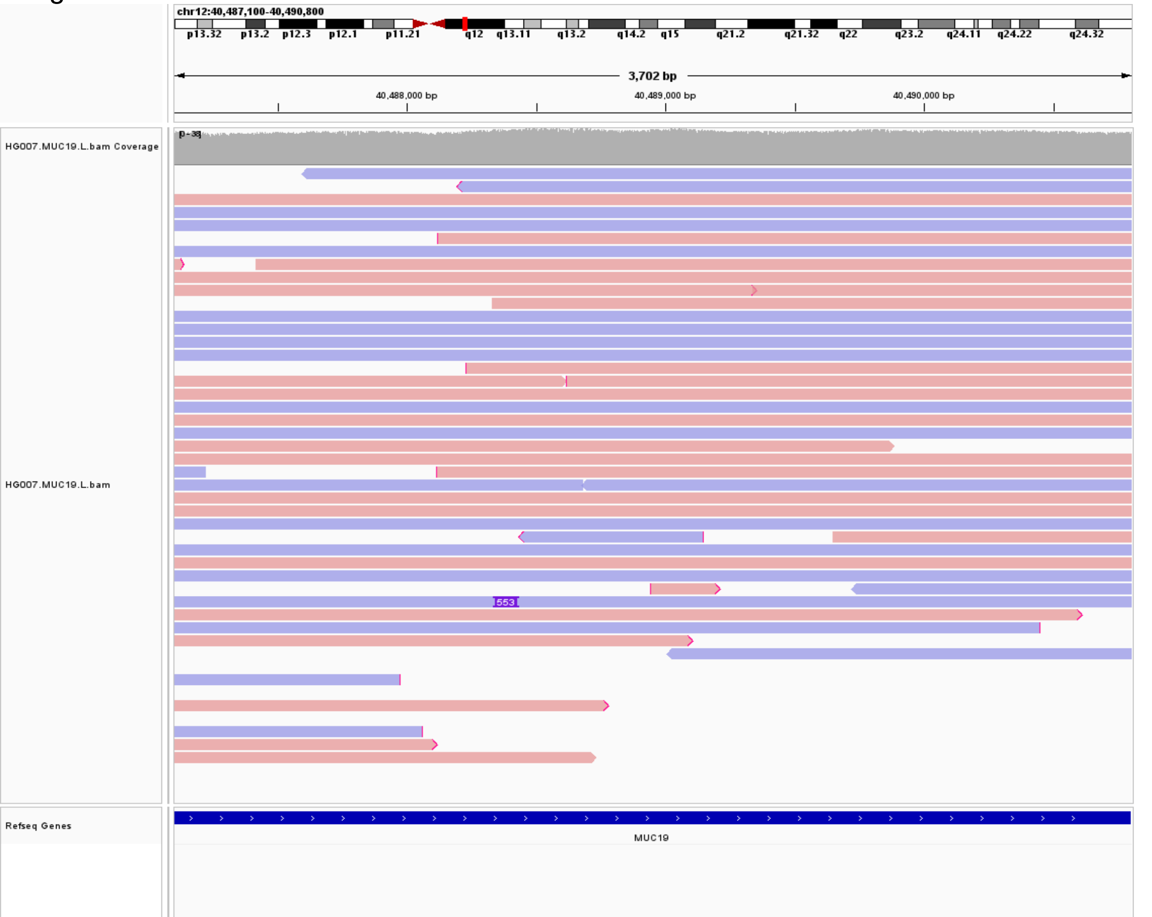

HG00731

Short−reads

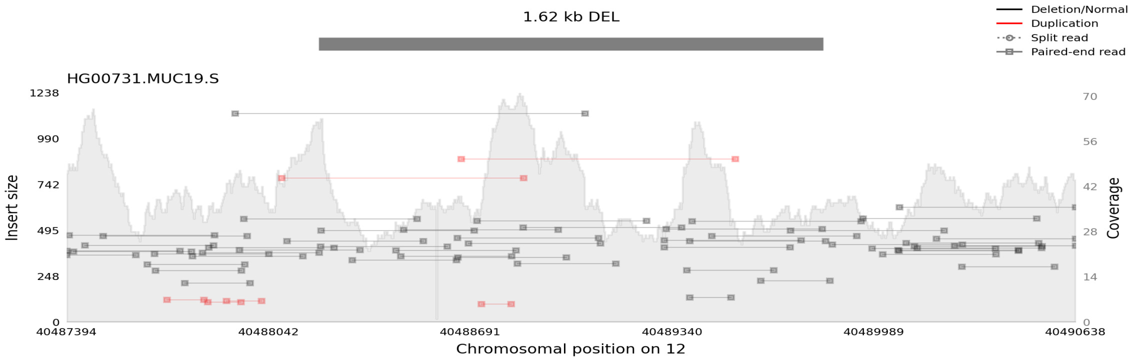

HG00731

Long−reads

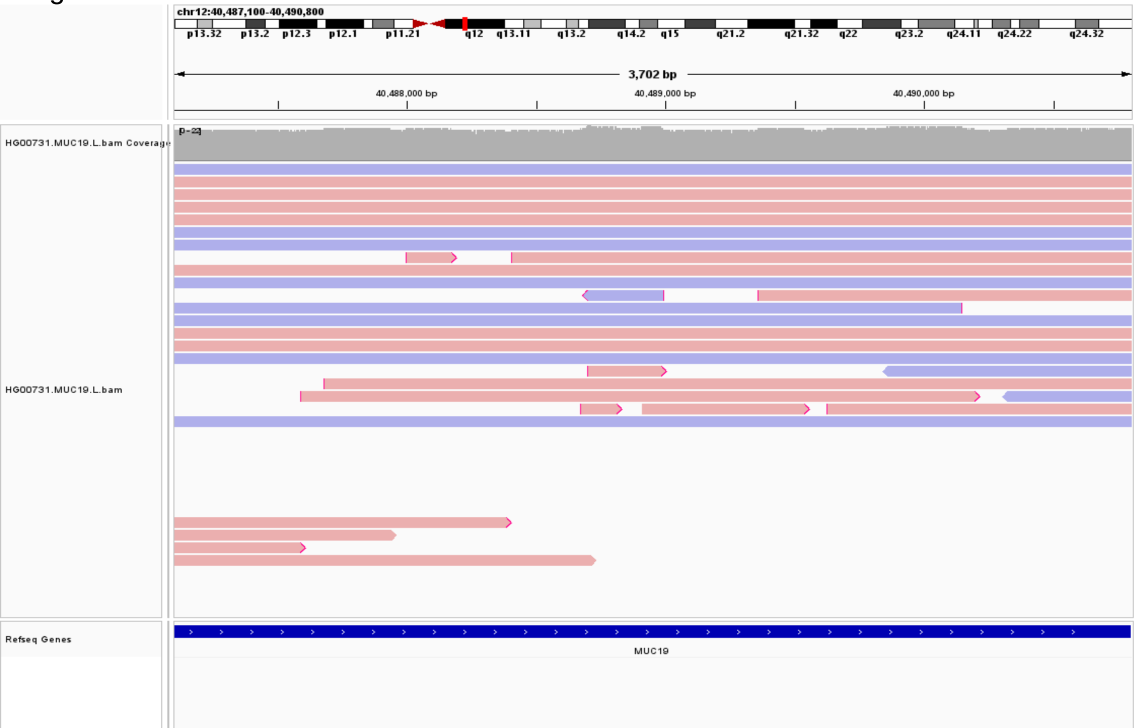

HG00732

Short−reads

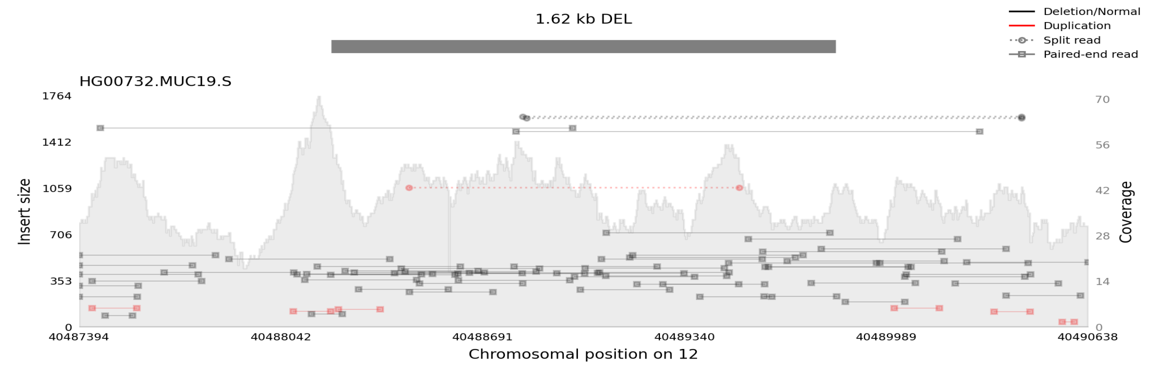

HG00732

Long−reads

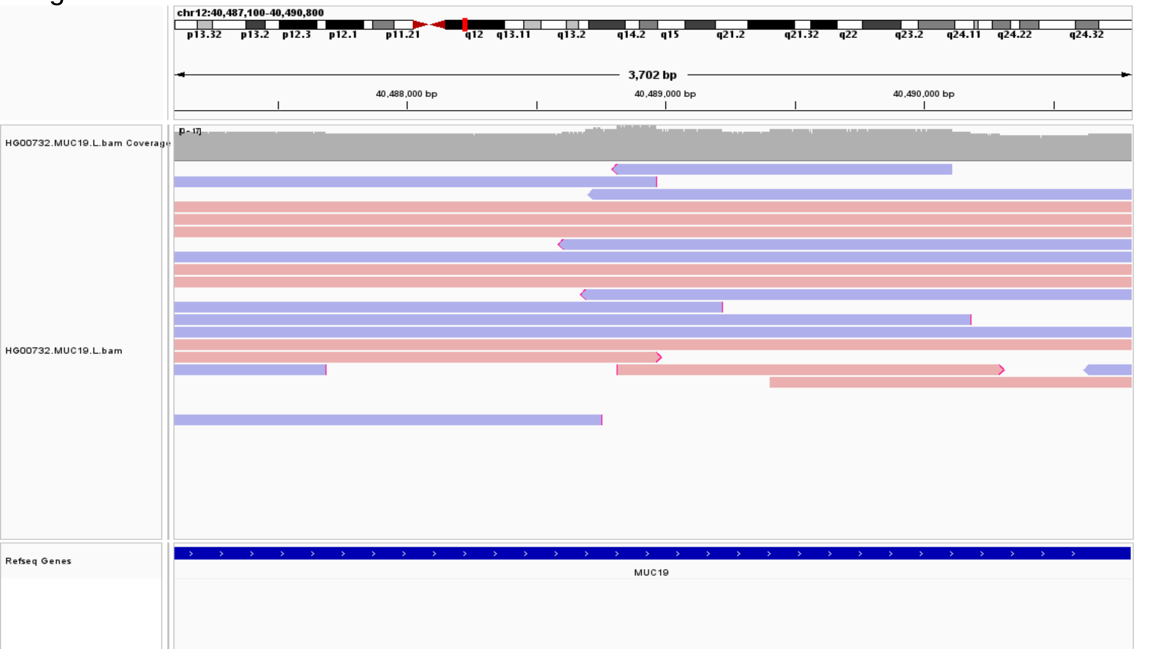

HG00733

Short−reads

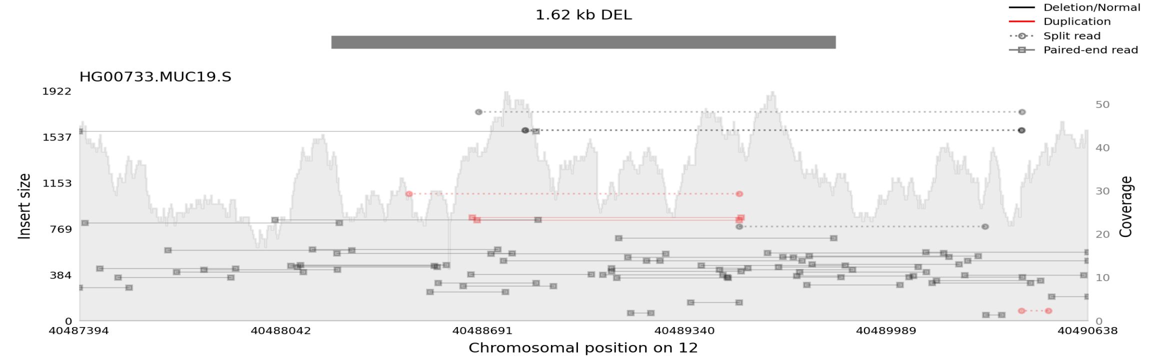

HG00733

Long−reads

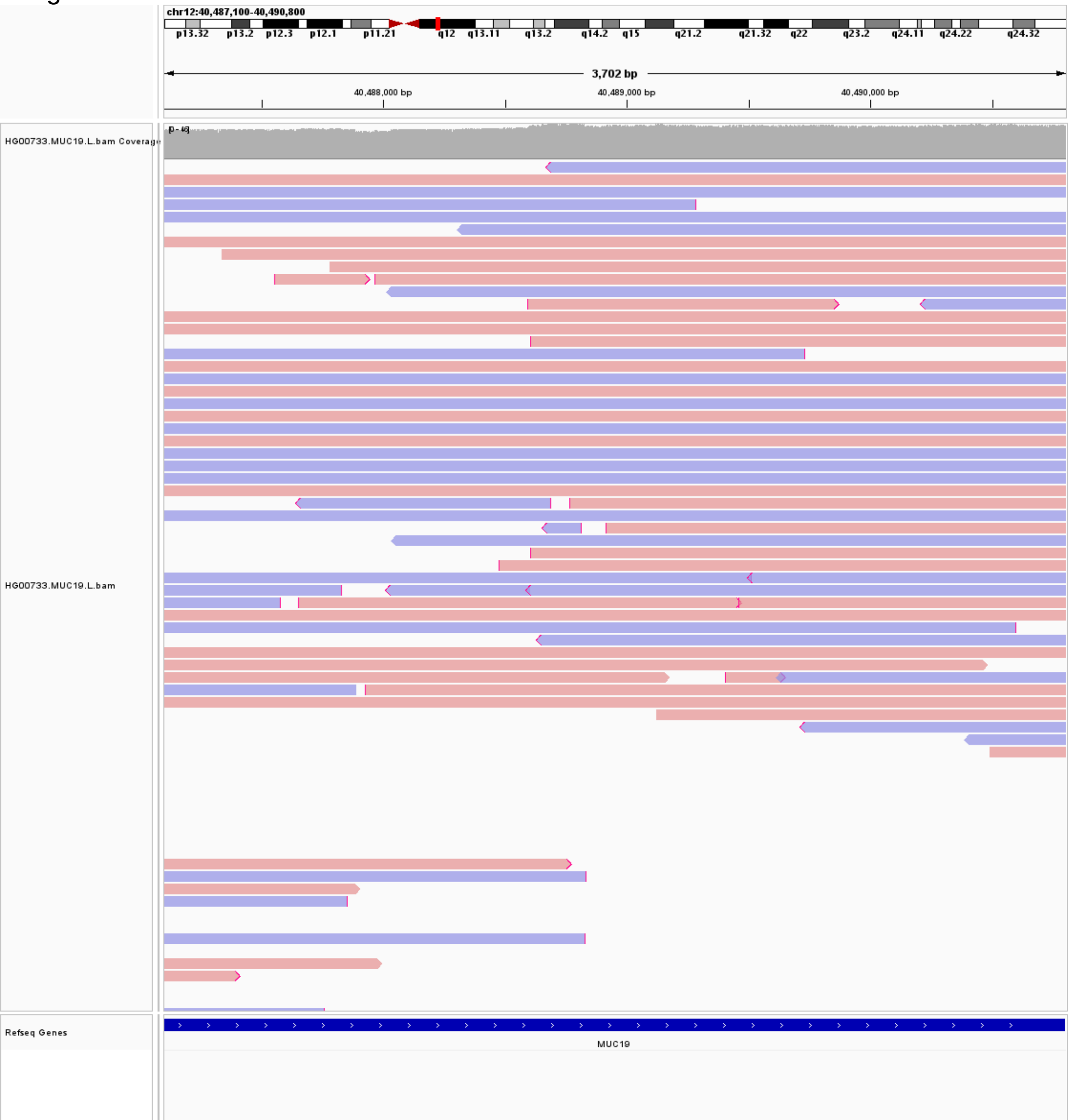

HG00738

Short−reads

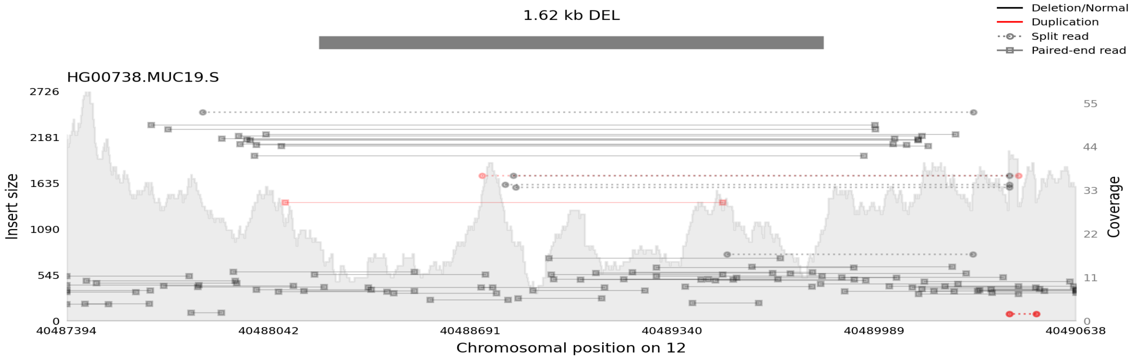

HG00738

Long−reads

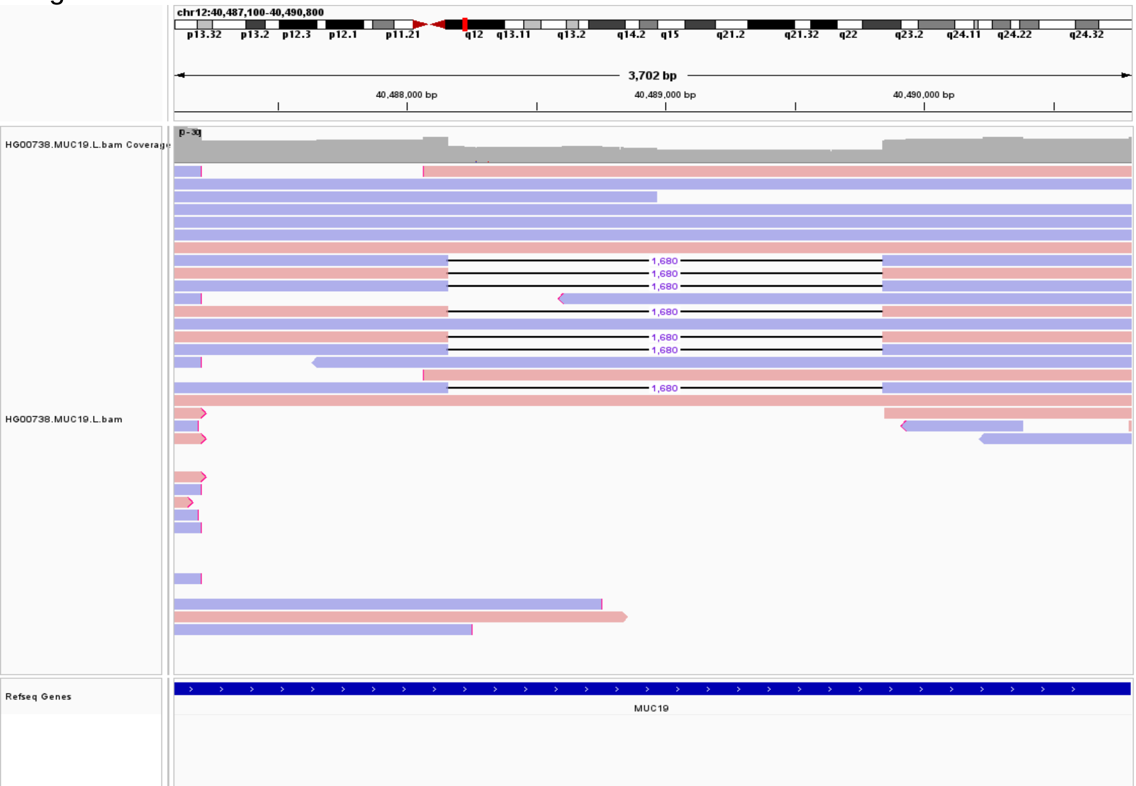

HG01074

Short−reads

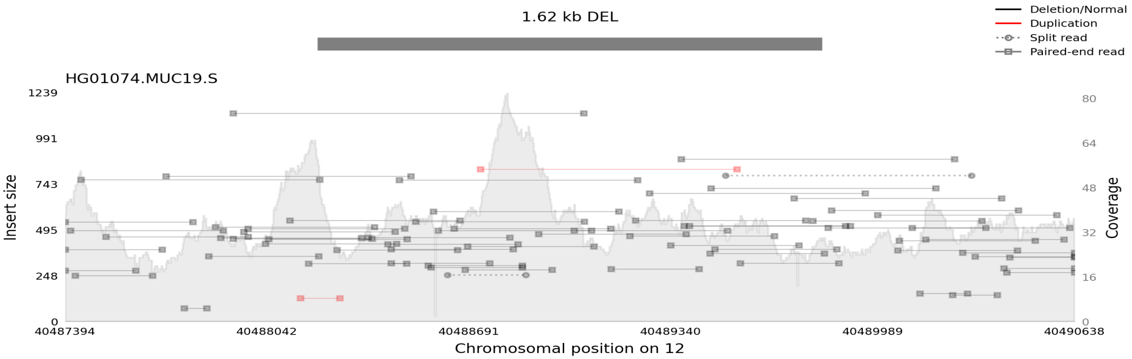

HG01074

Long−reads

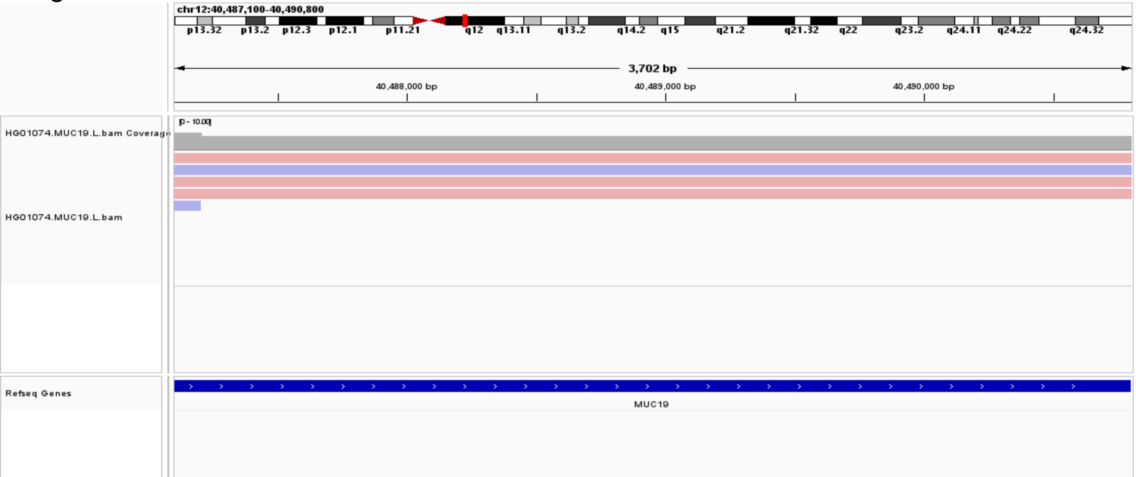

HG01081

Short−reads

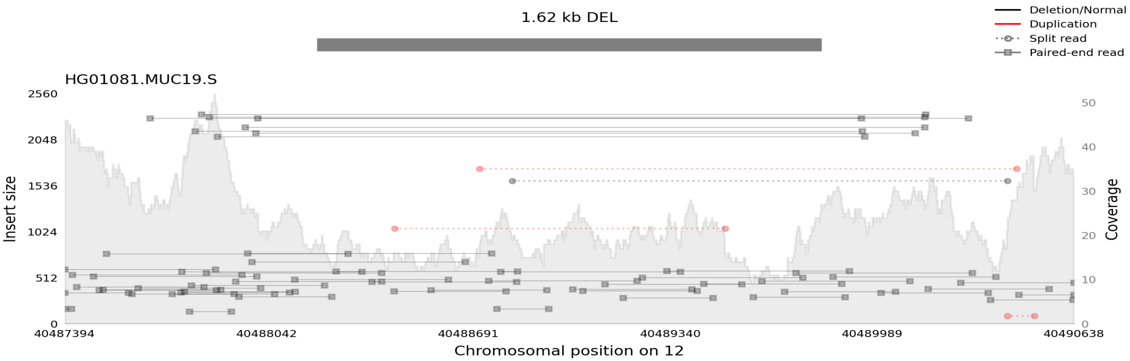

HG01081

Long−reads

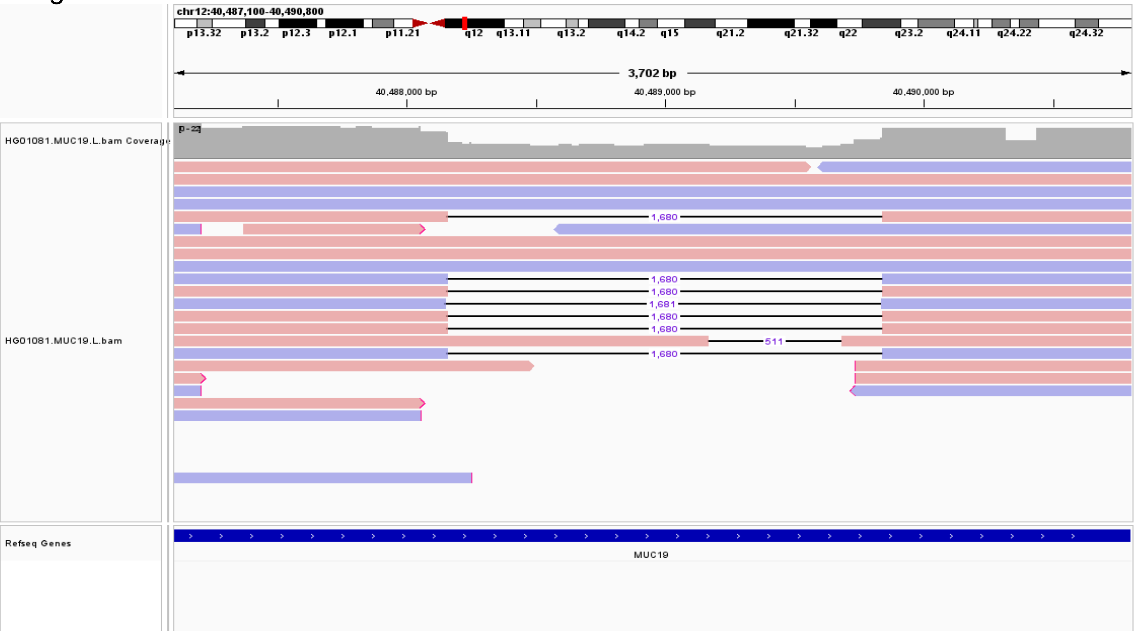

HG01099

Short−reads

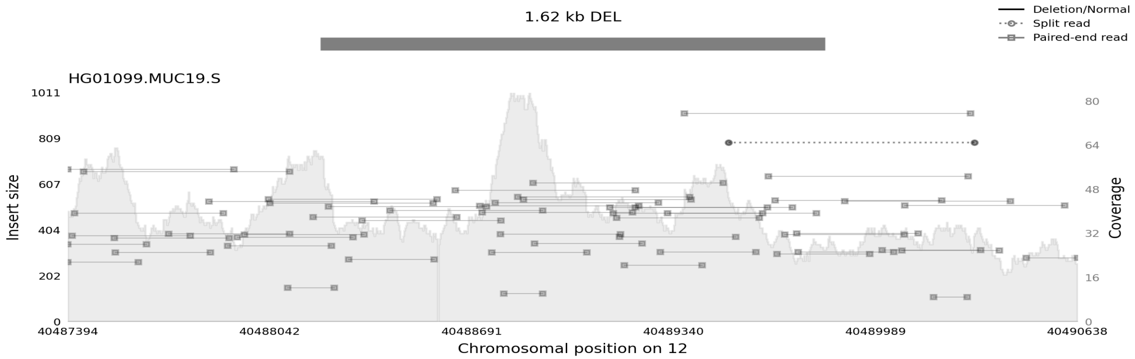

HG01099

Long−reads

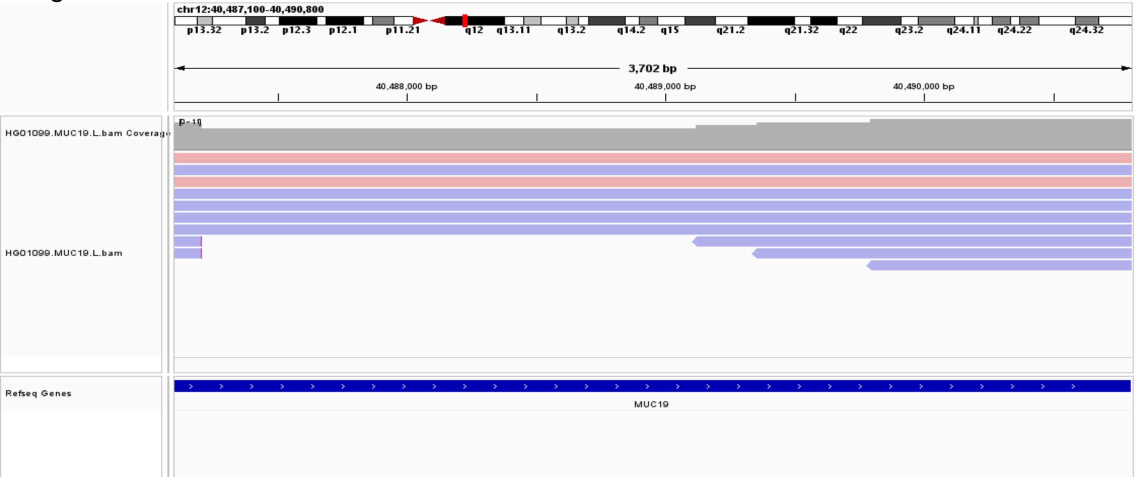

HG01175

Short−reads

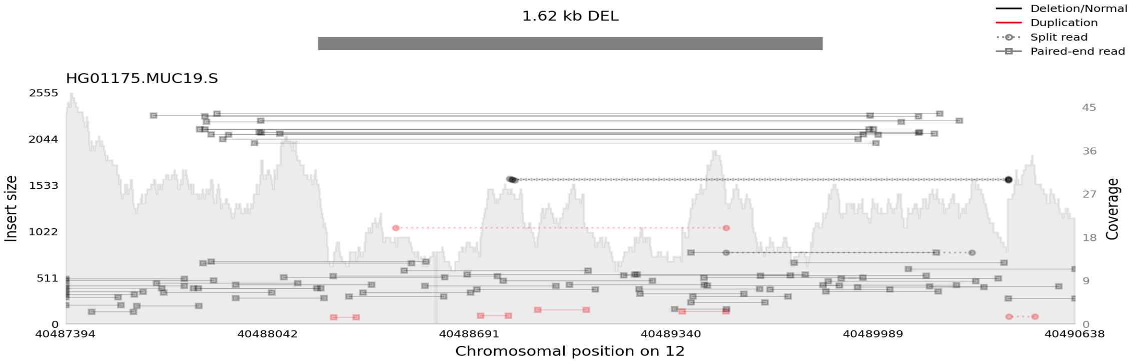

HG01175

Long−reads

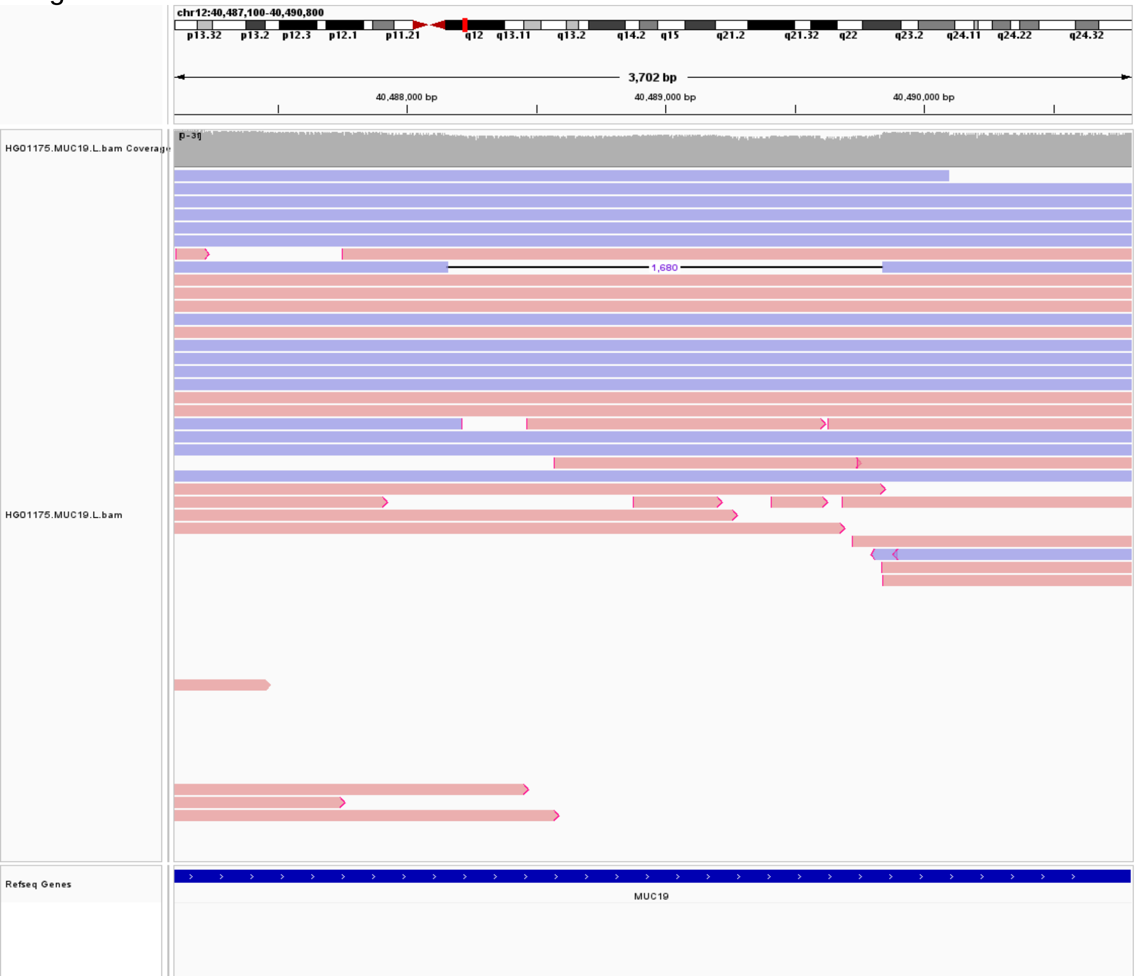

HG01192

Short−reads

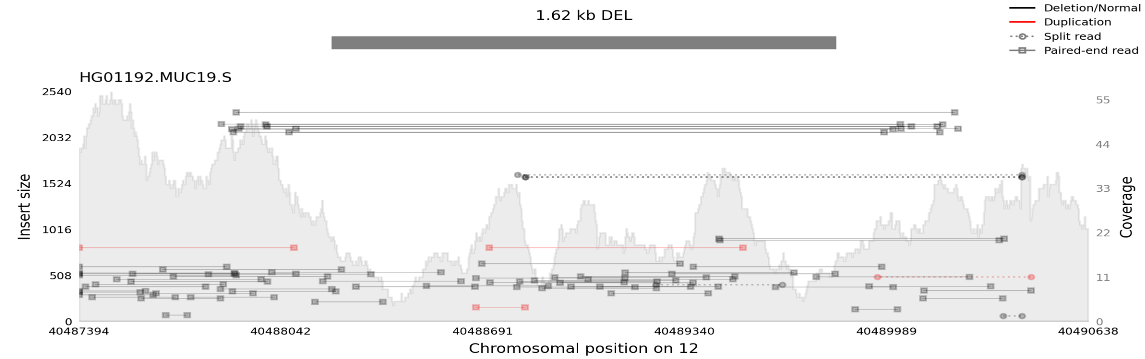

HG01192

Long−reads

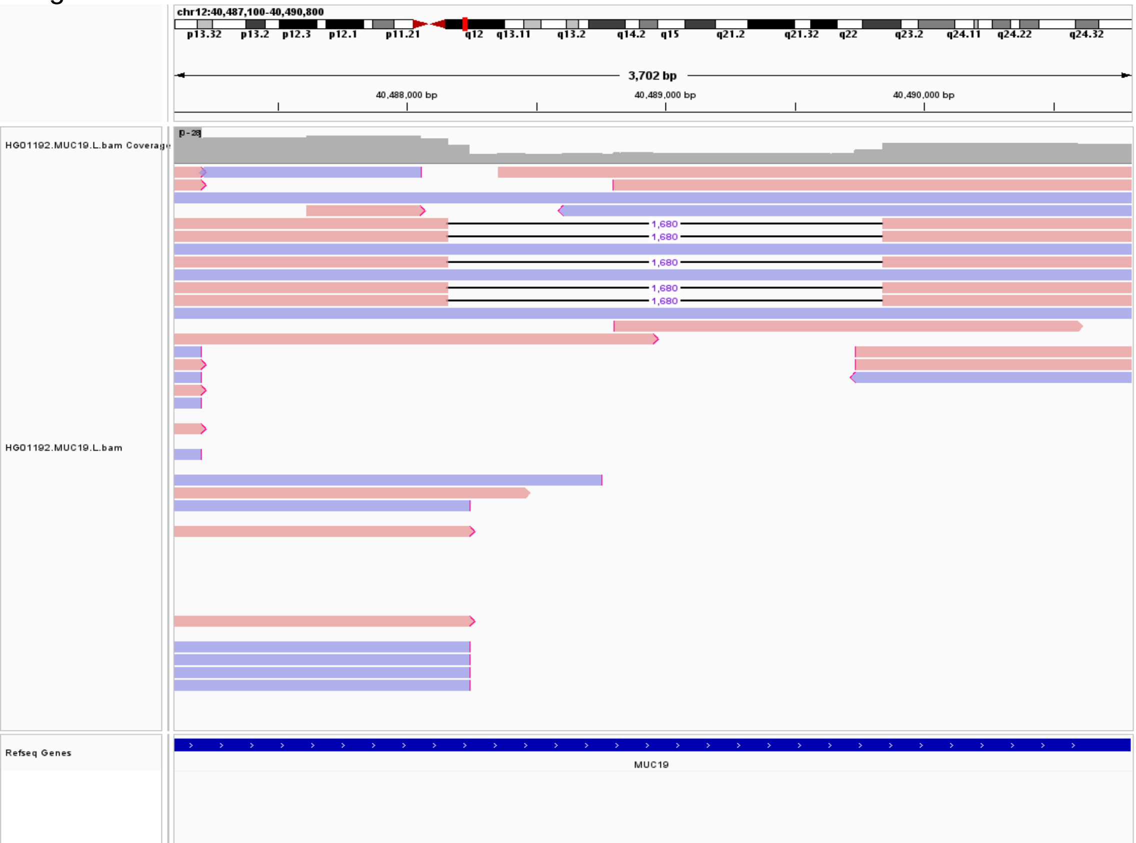

HG01252
Short−reads

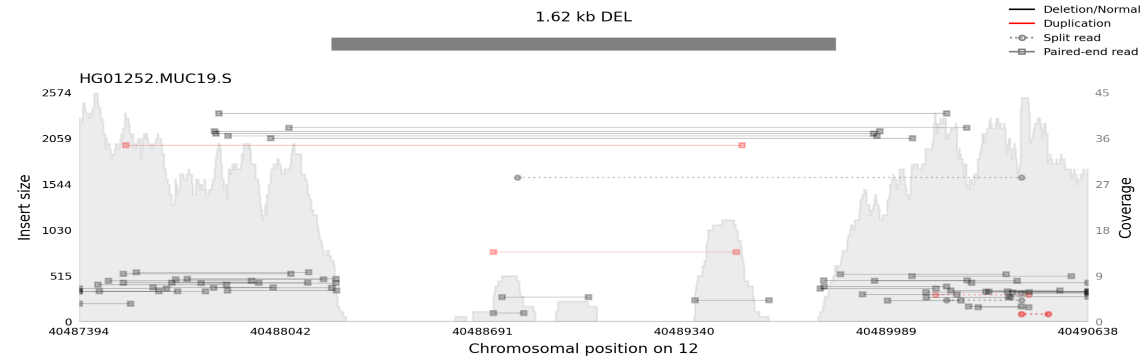

HG01252
Long−reads

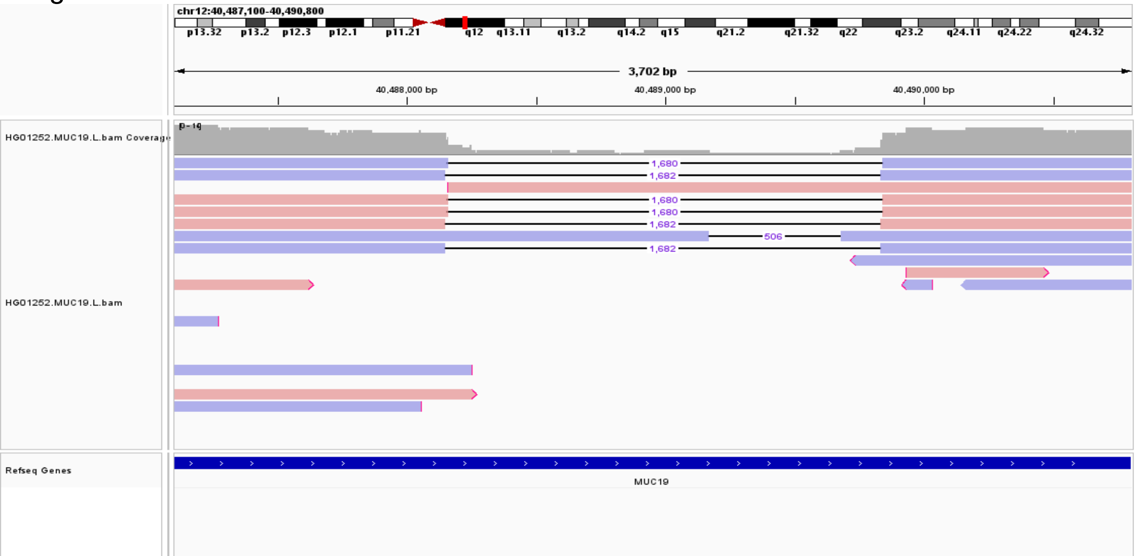

HG01255

Short−reads

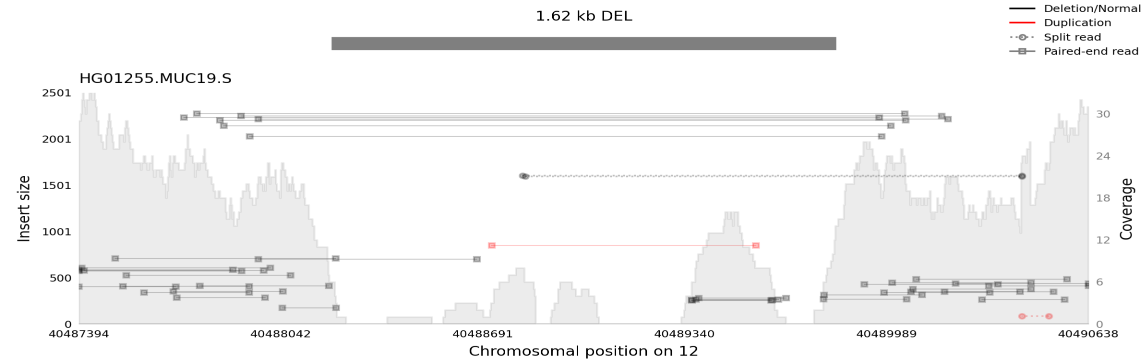

HG01255

Long−reads

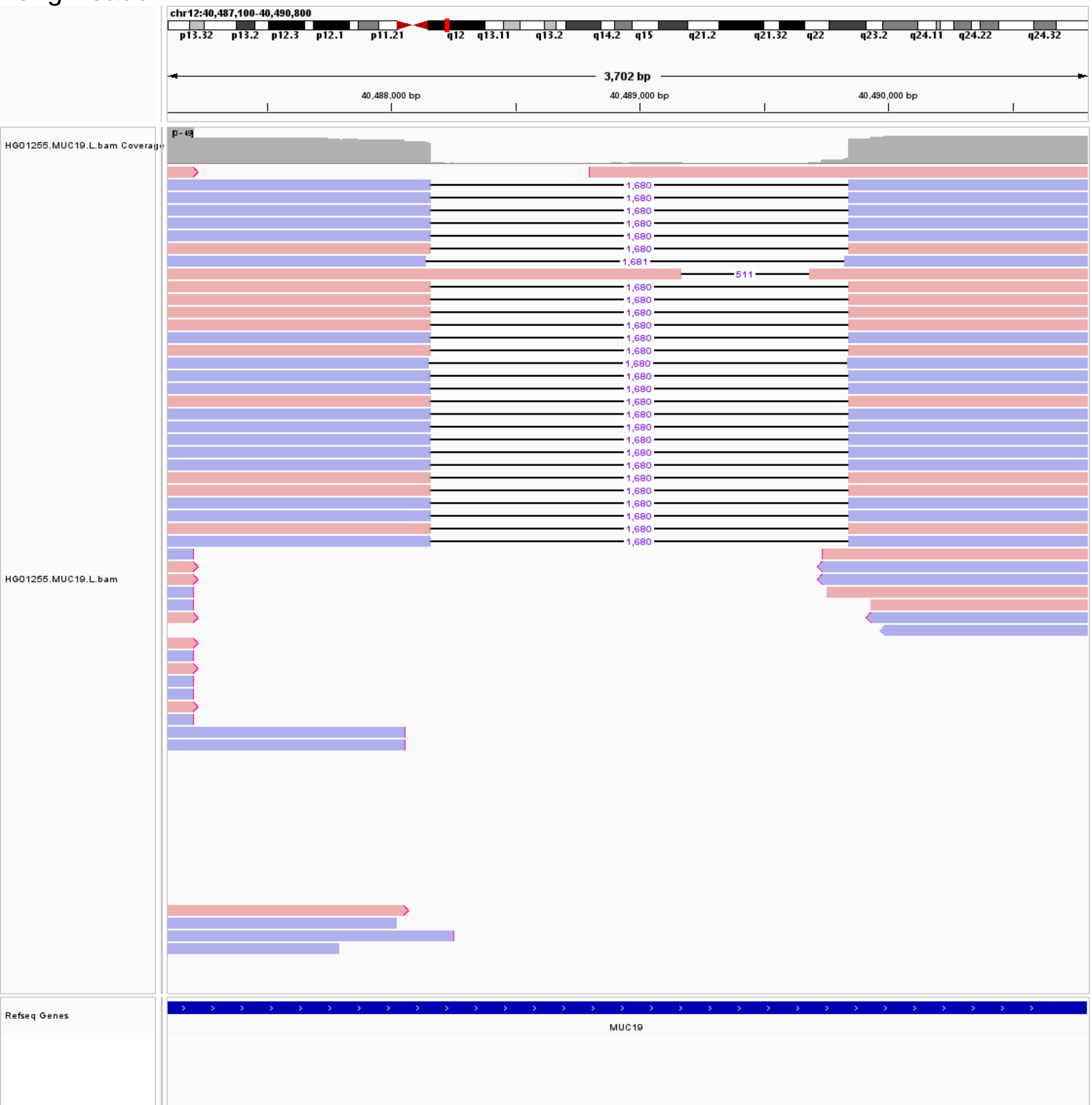

HG01258

Short−reads

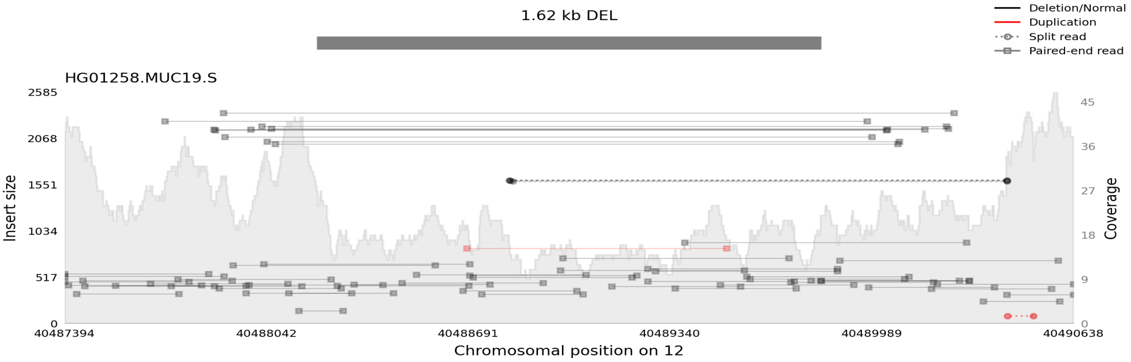

HG01258

Long−reads

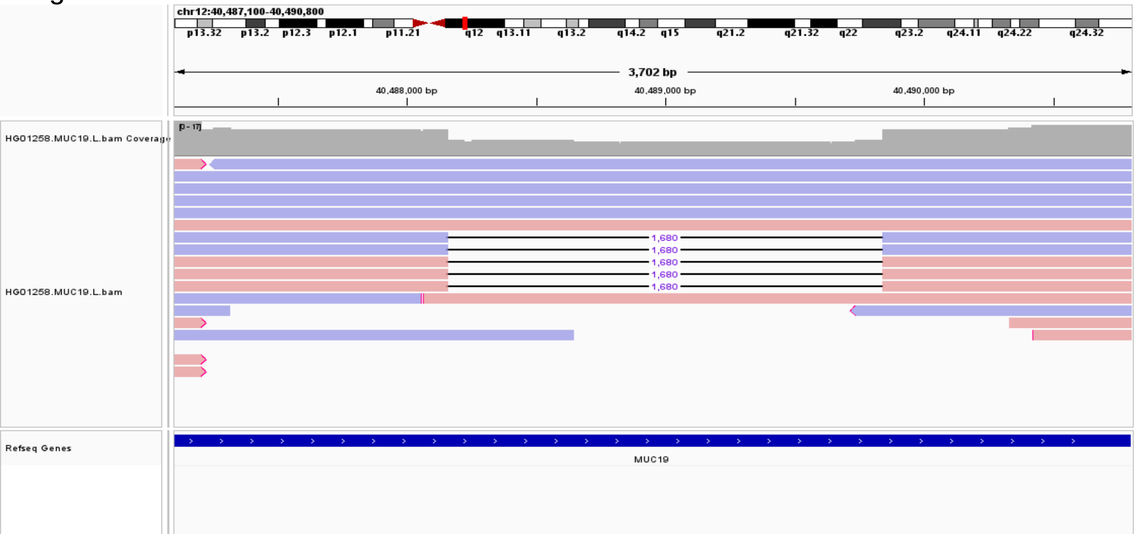

HG01261

Short−reads

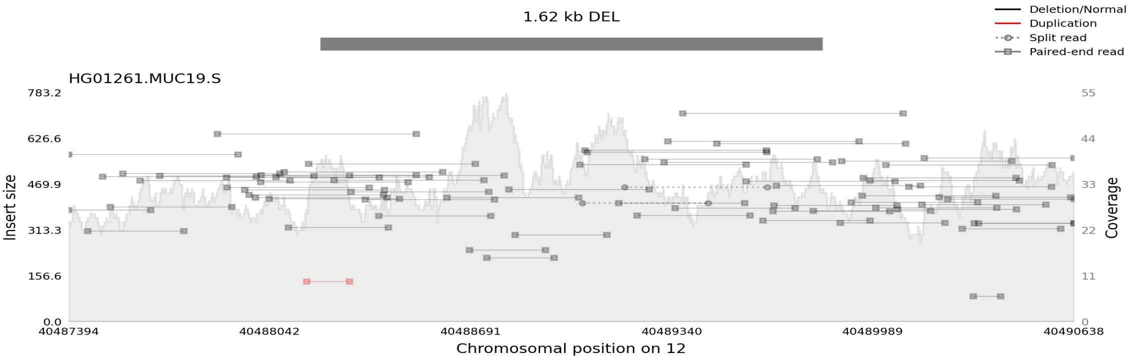

HG01261

Long−reads

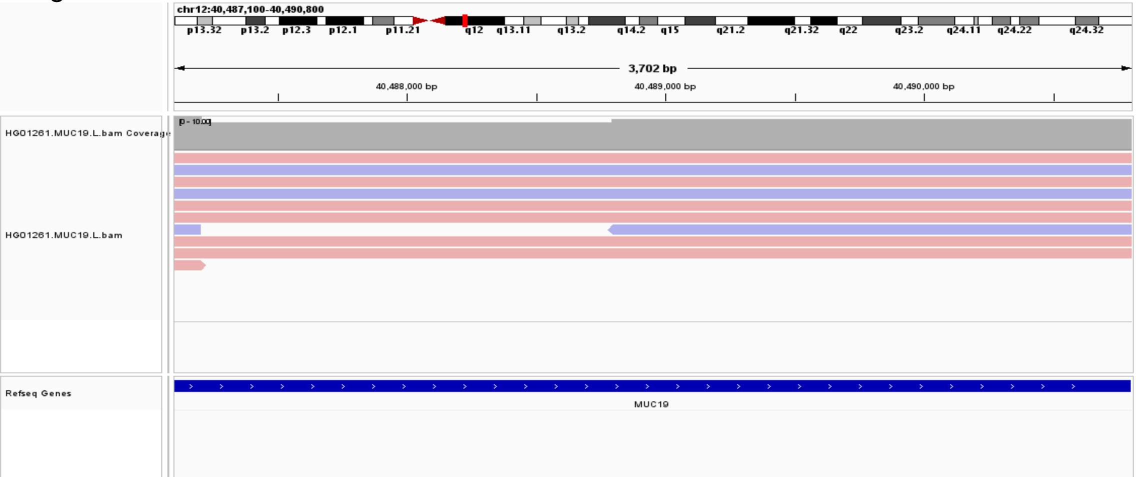

HG01346

Short−reads

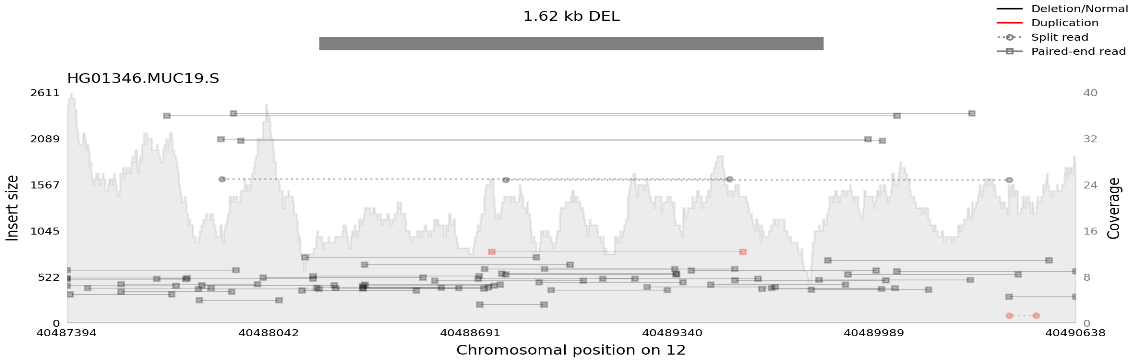

HG01346

Long−reads

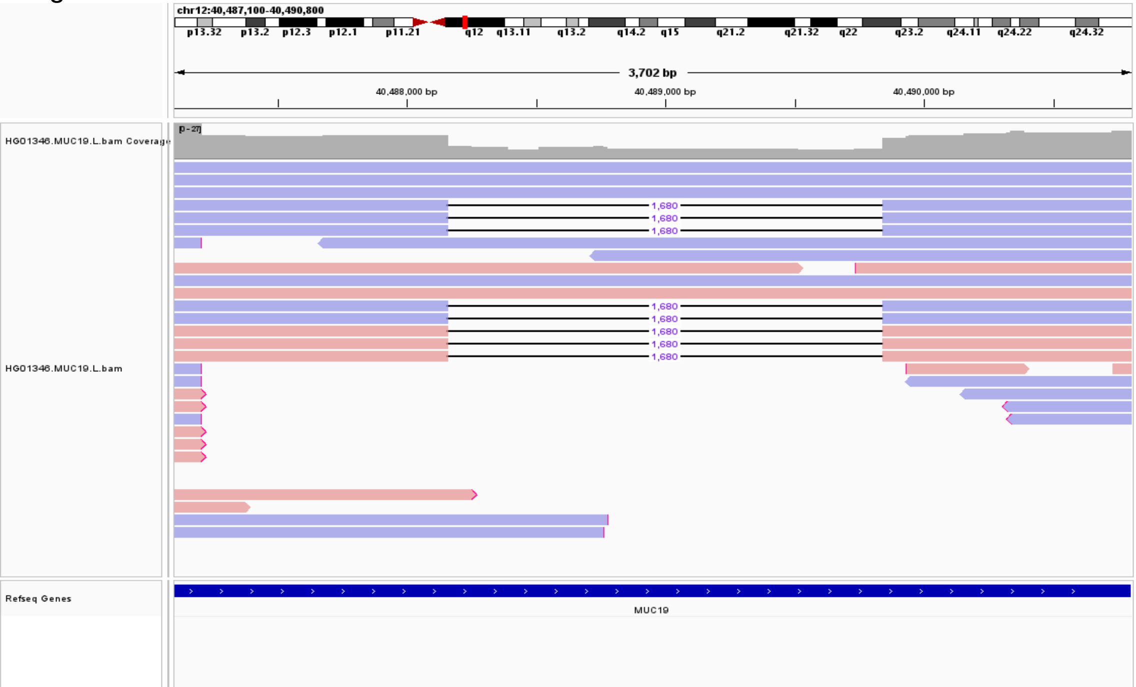

HG01352

Short−reads

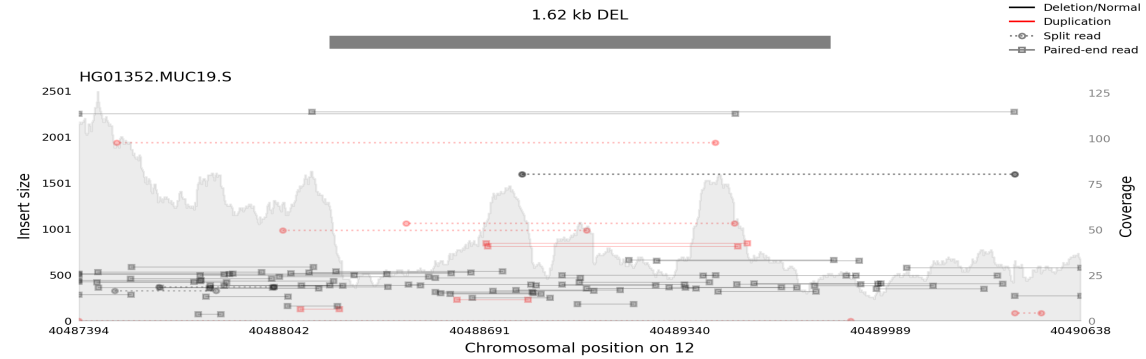

HG01352

Long−reads

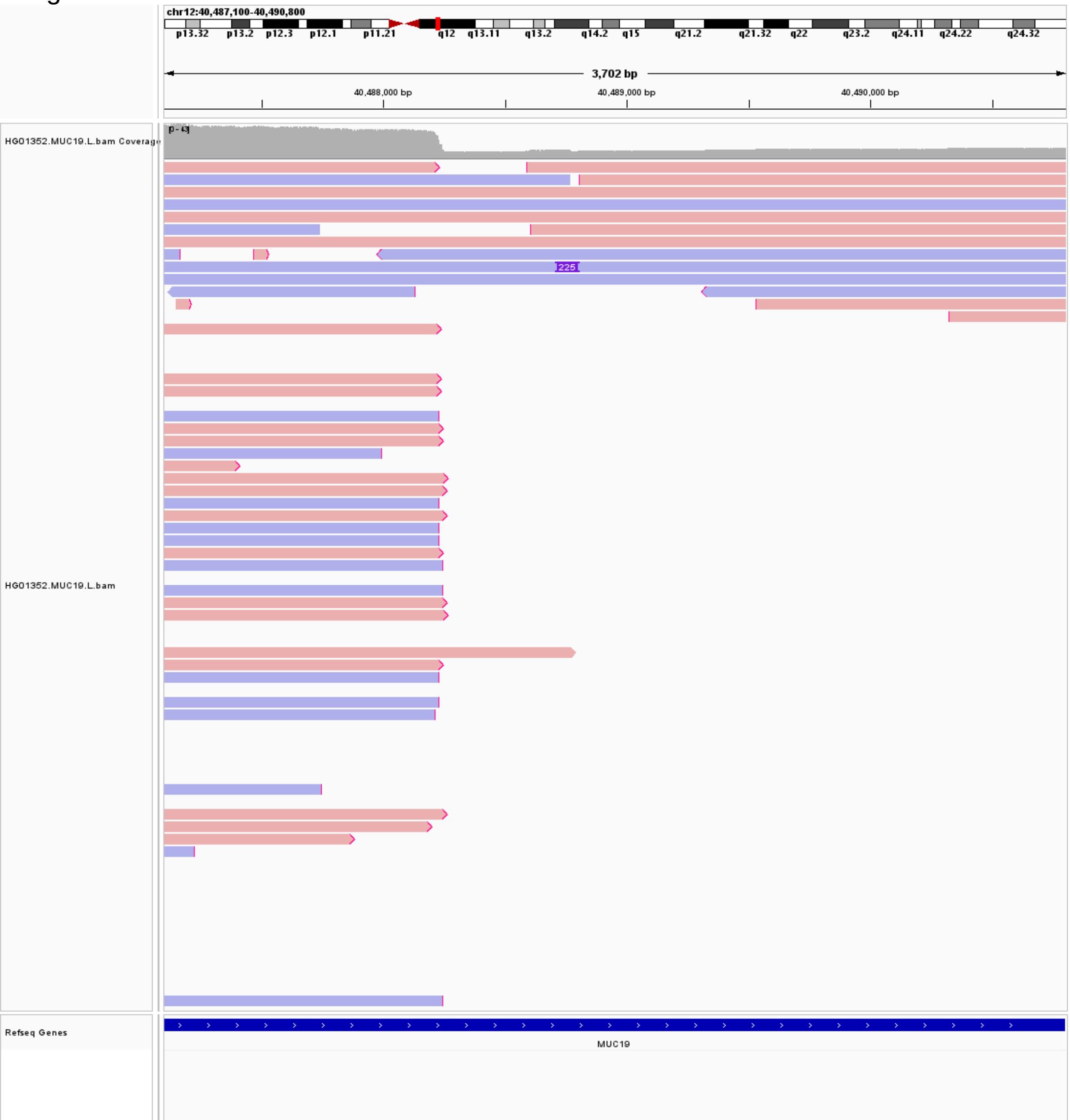

HG01358

Short−reads

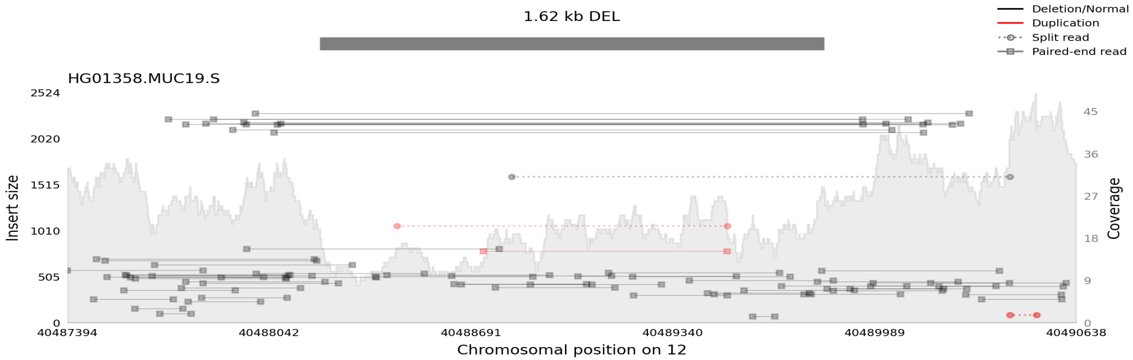

HG01358

Long−reads

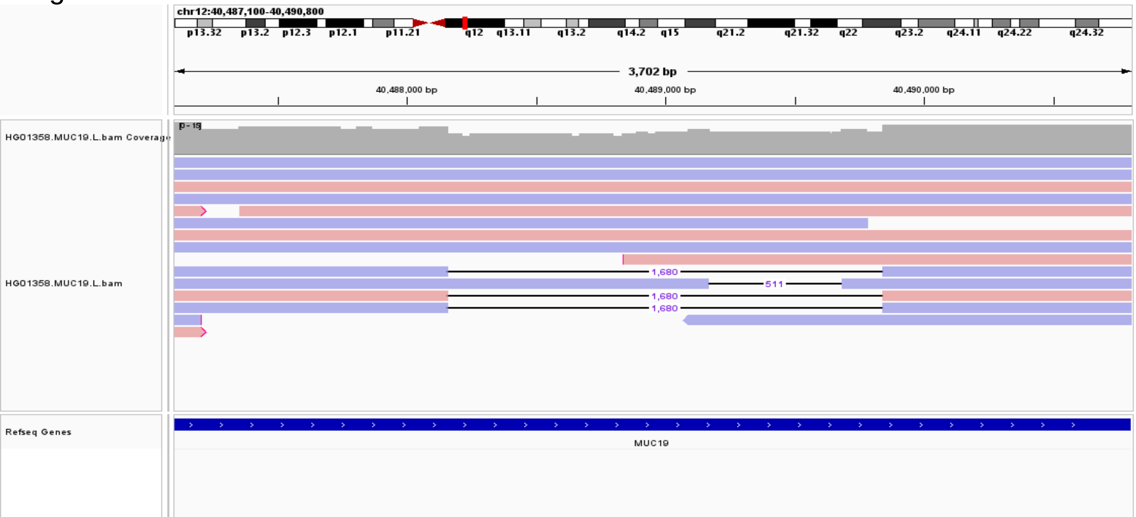

HG01433

Short−reads

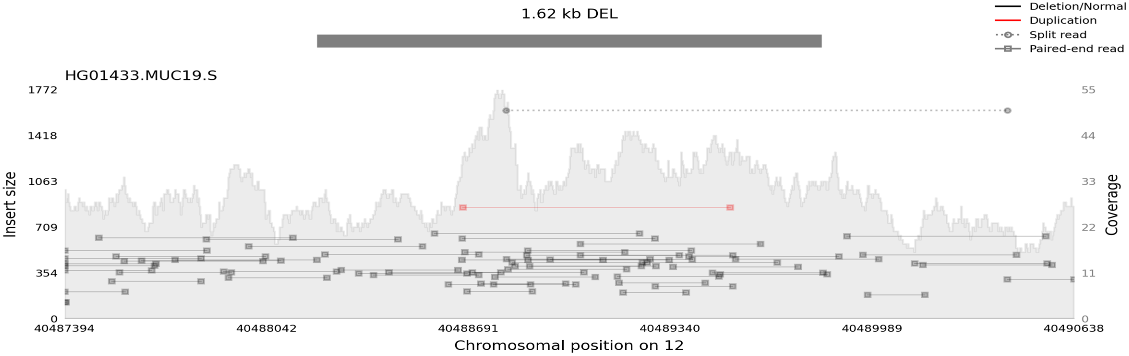

HG01433

Long−reads

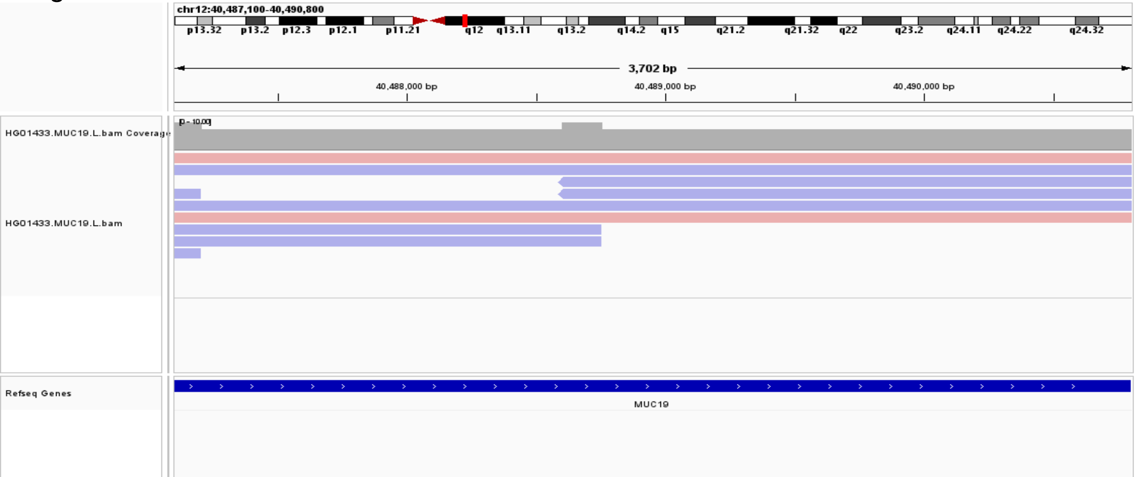

HG01496

Short−reads

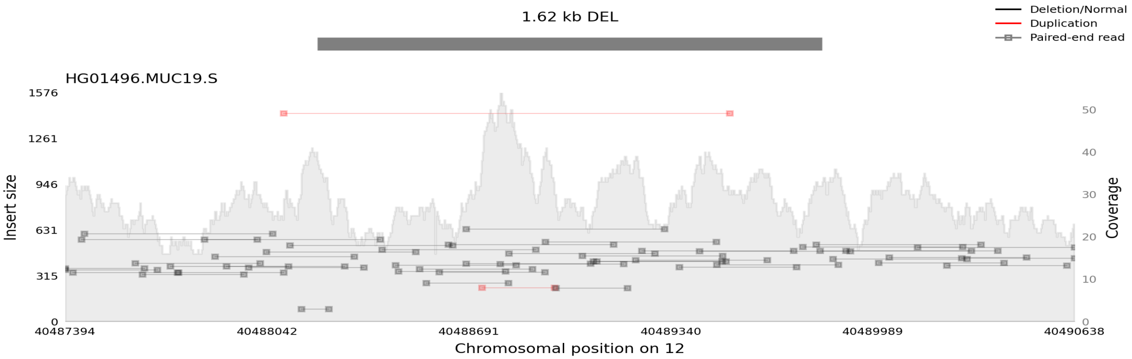

HG01496

Long−reads

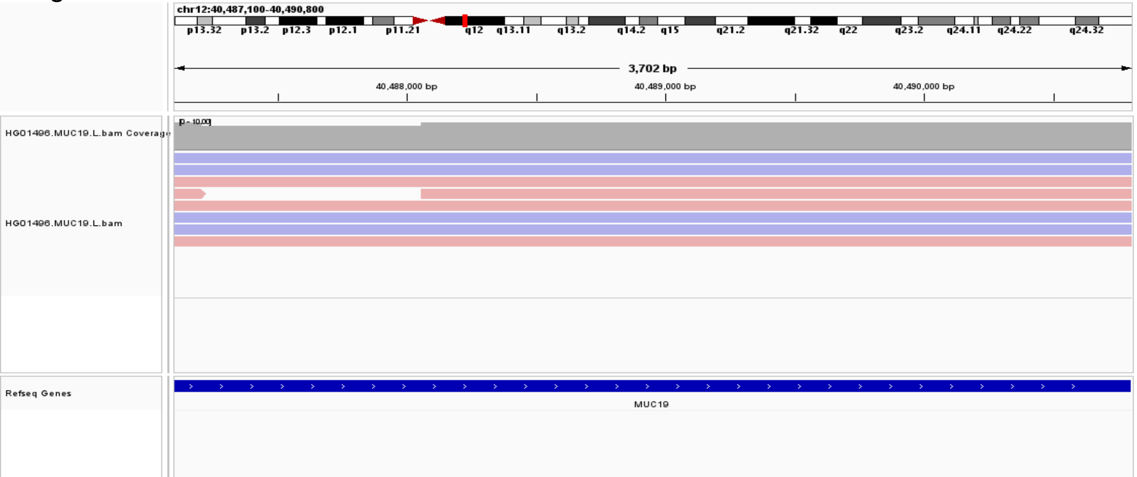

HG01596

Short−reads

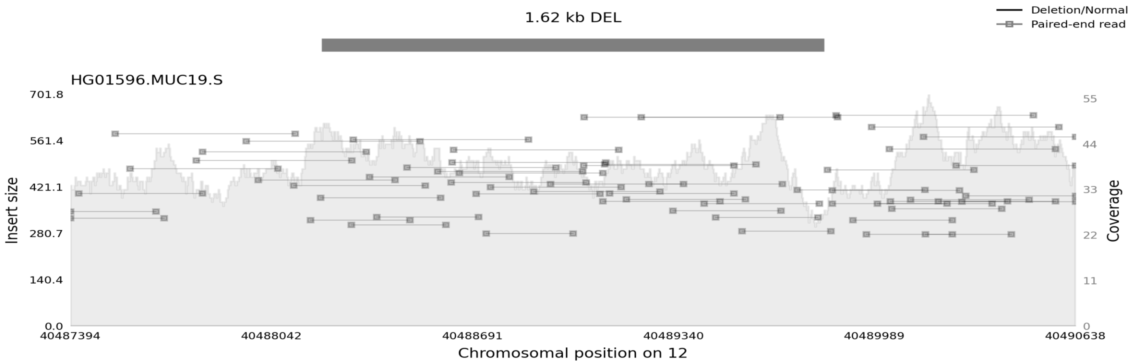

HG01596

Long−reads

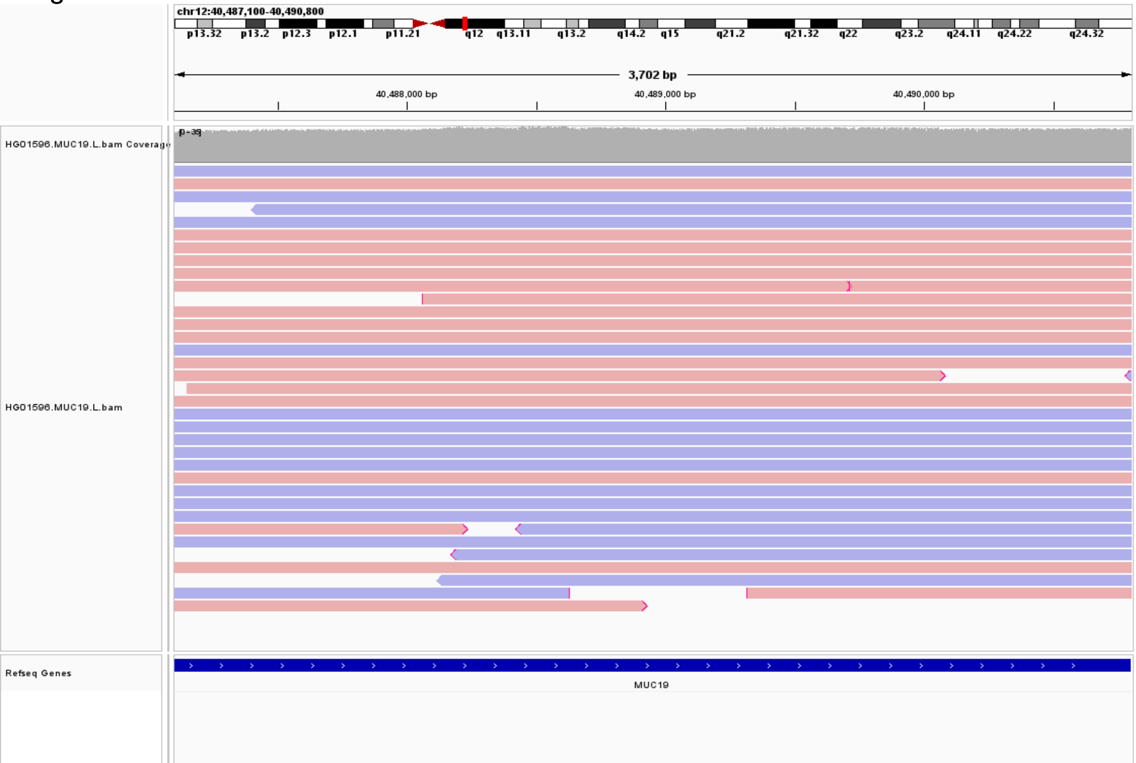

HG01884

Short−reads

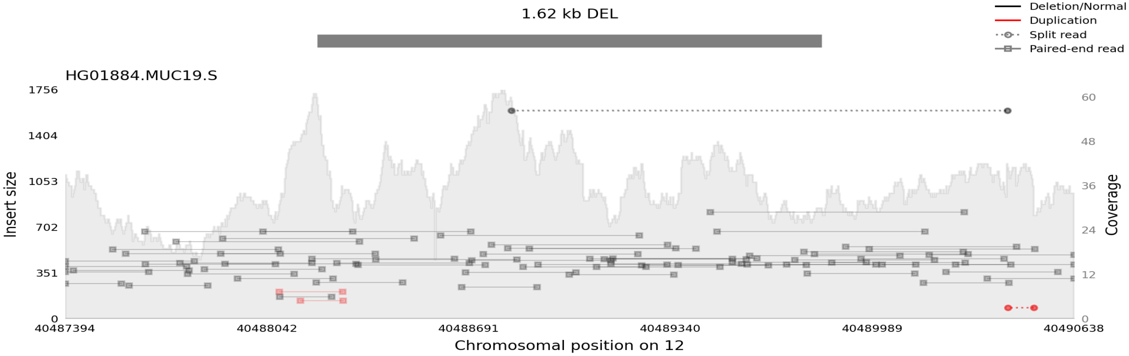

HG01884

Long−reads

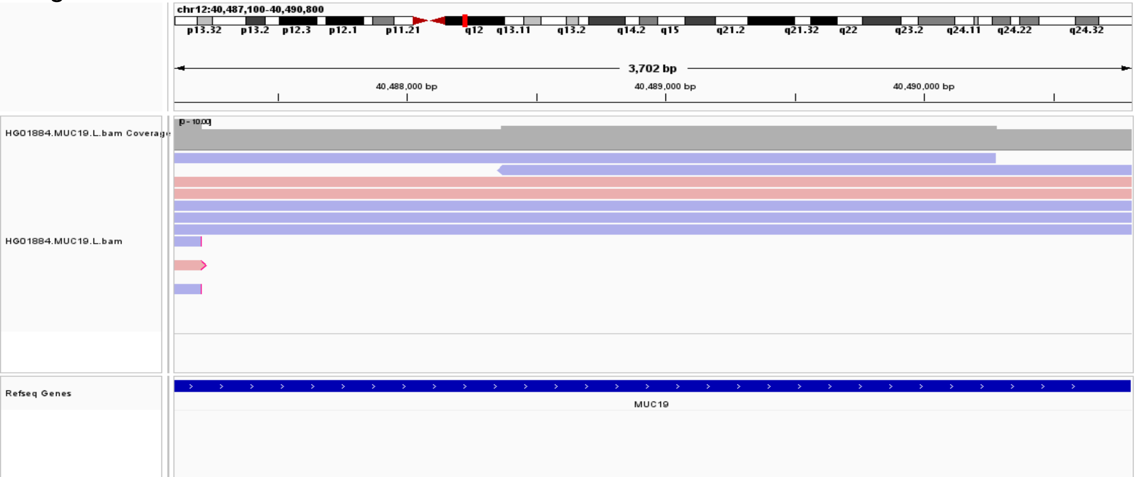

HG01891

Short−reads

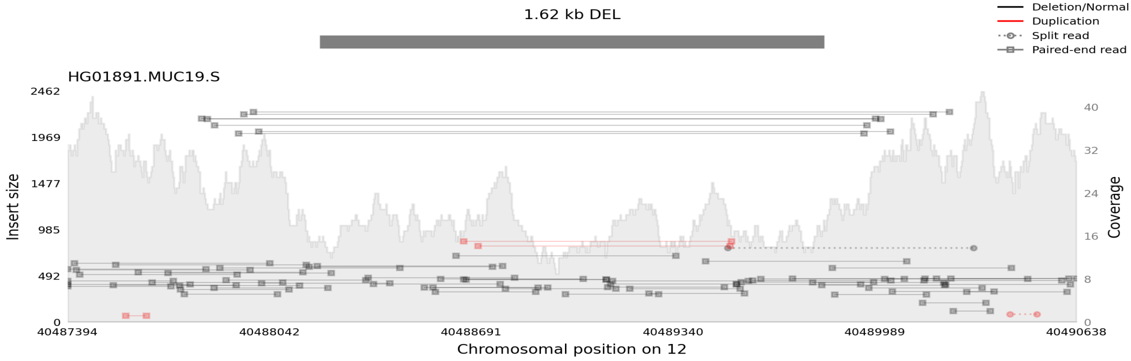

HG01891

Long−reads

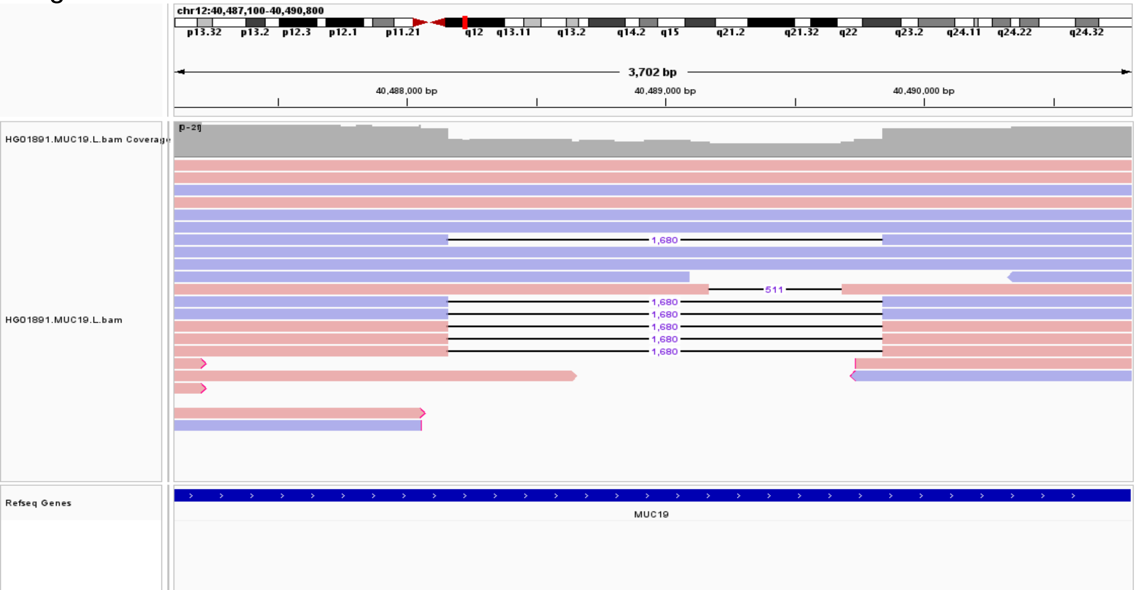

HG01934

Short−reads

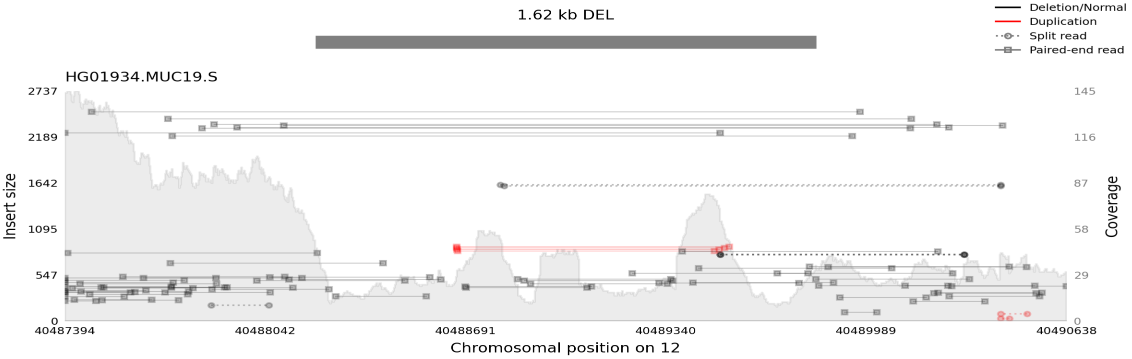

HG01934

Long−reads

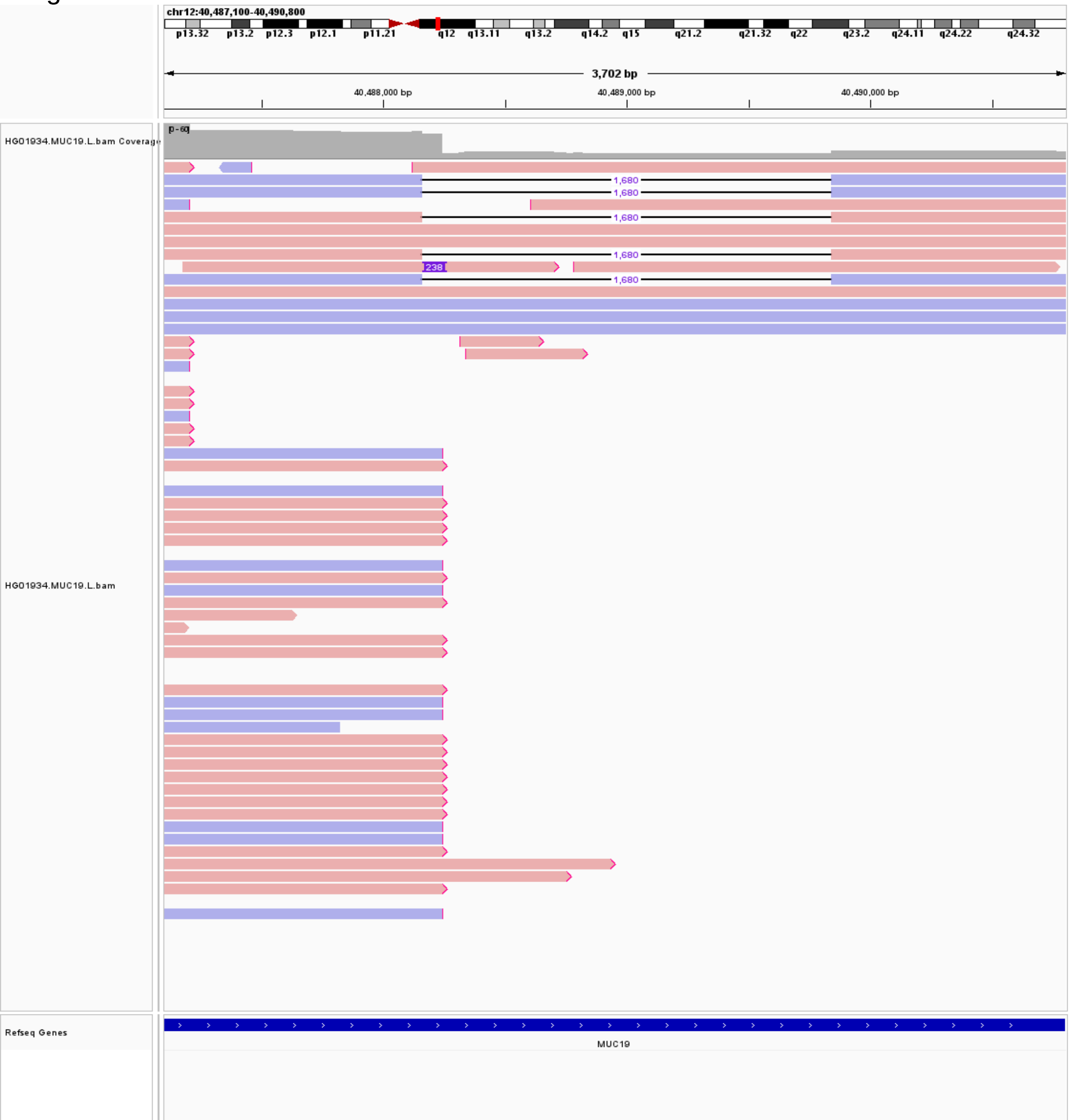

HG01943

Short−reads

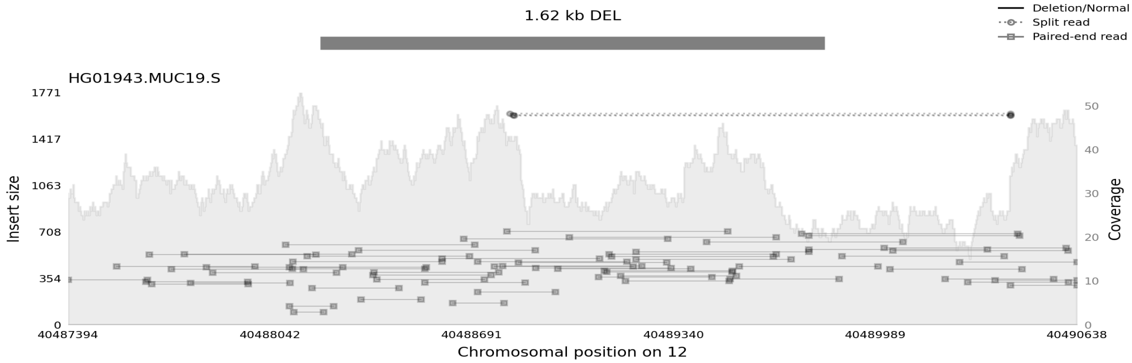

HG01943

Long−reads

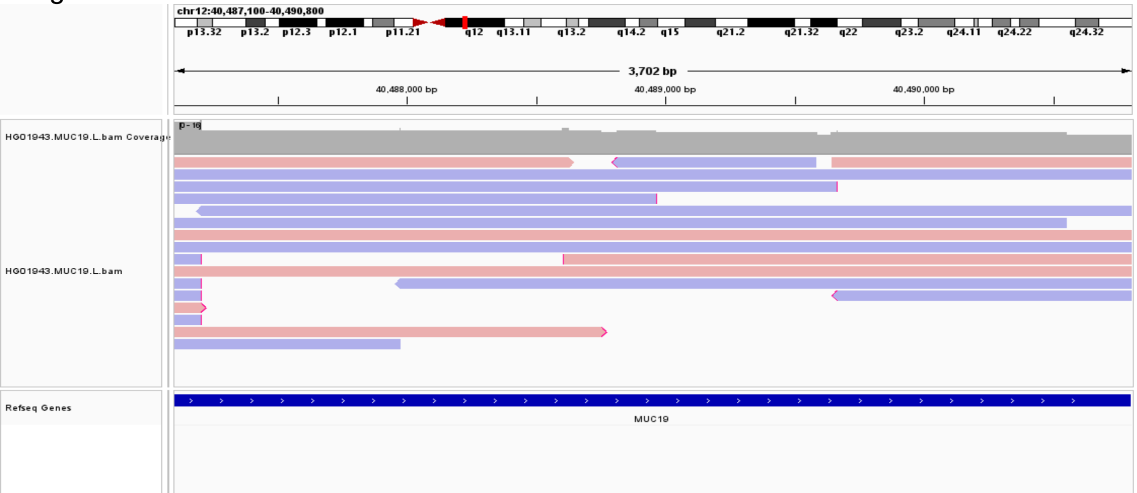

HG01975

Short−reads

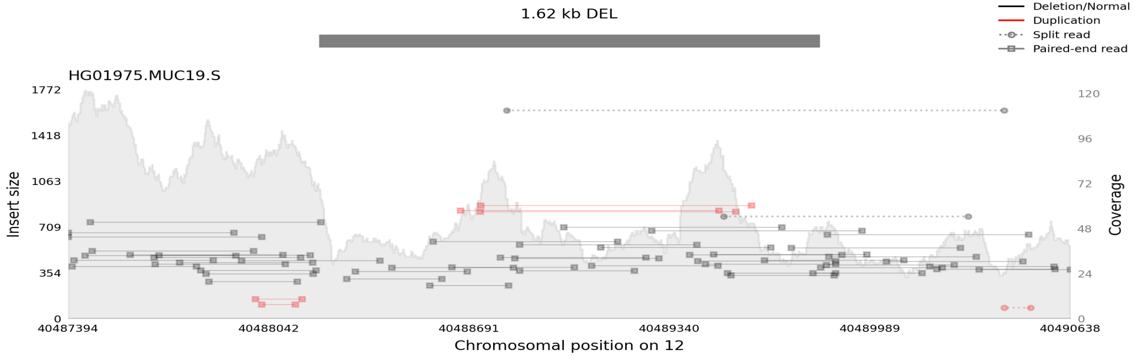

HG01975

Long−reads

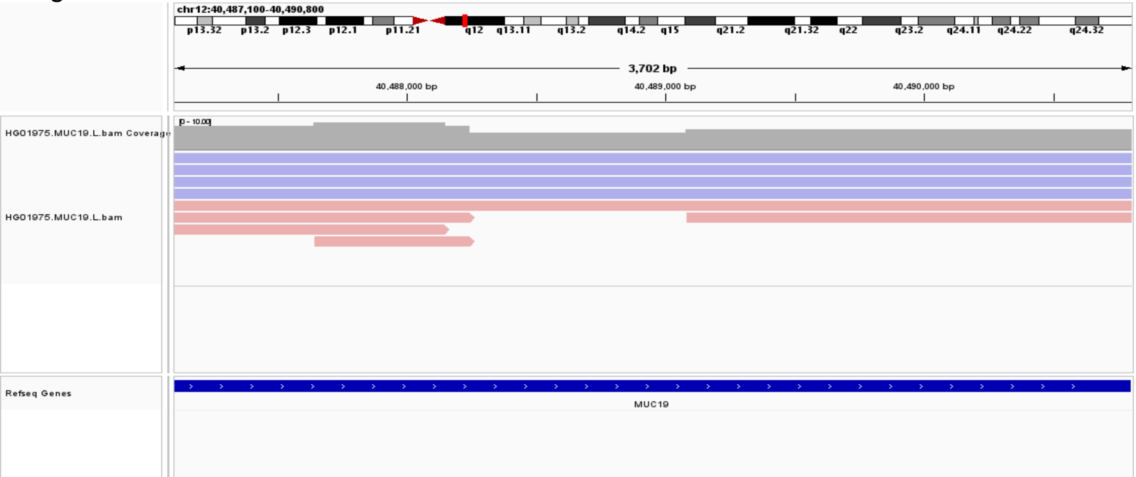

HG01981

Short−reads

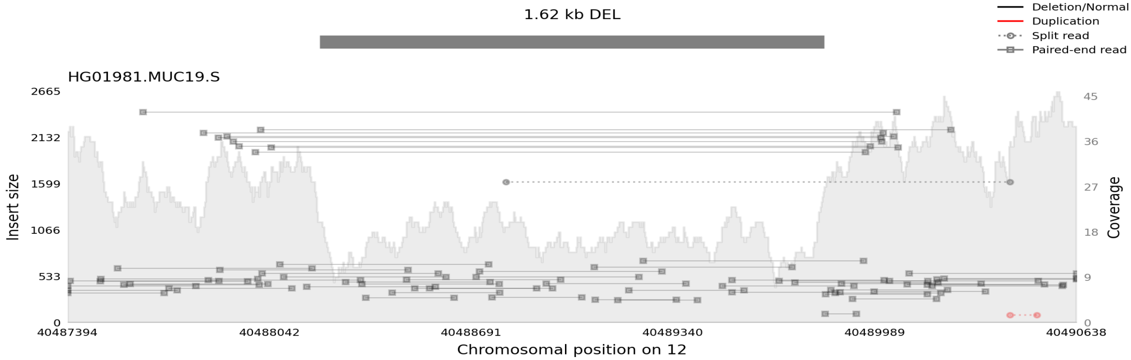

HG01981

Long−reads

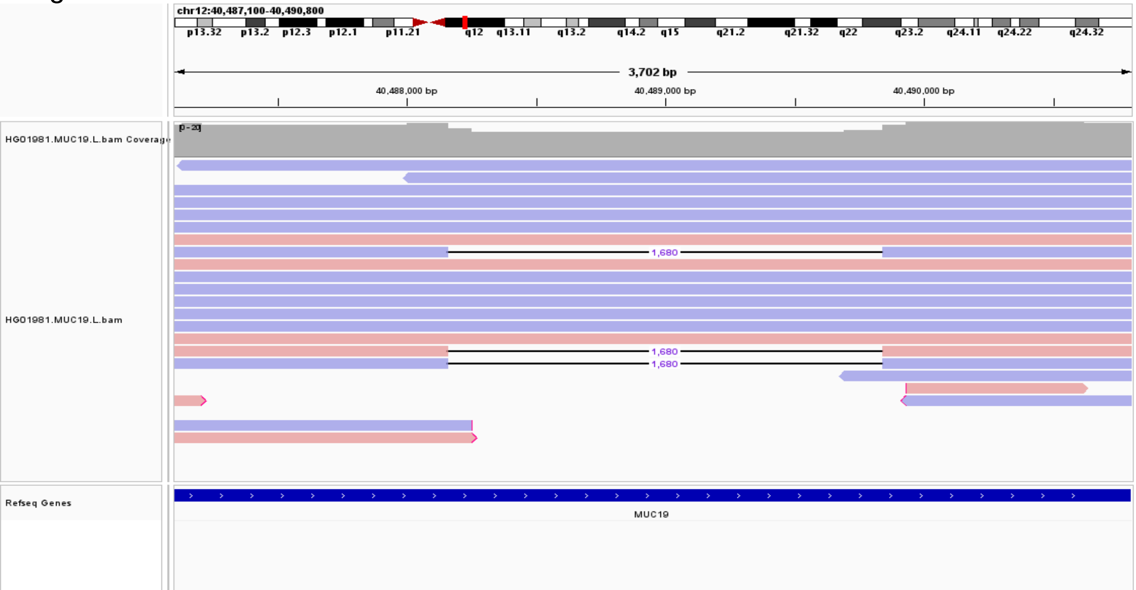

HG01993

Short−reads

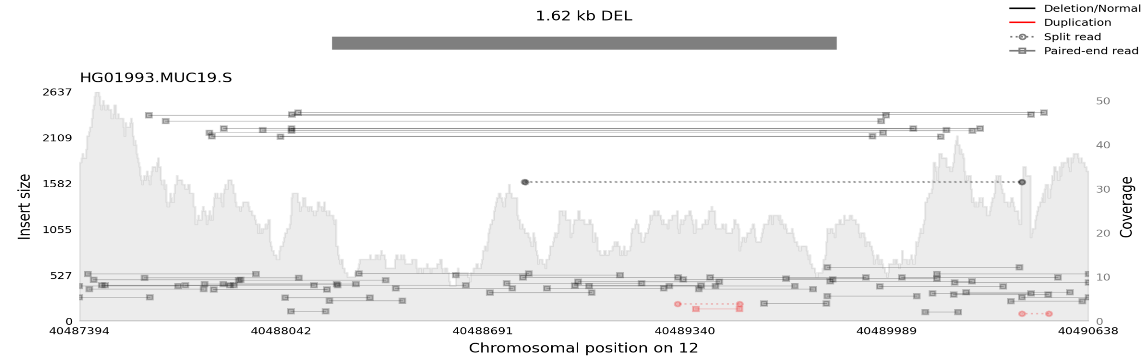

HG01993

Long−reads

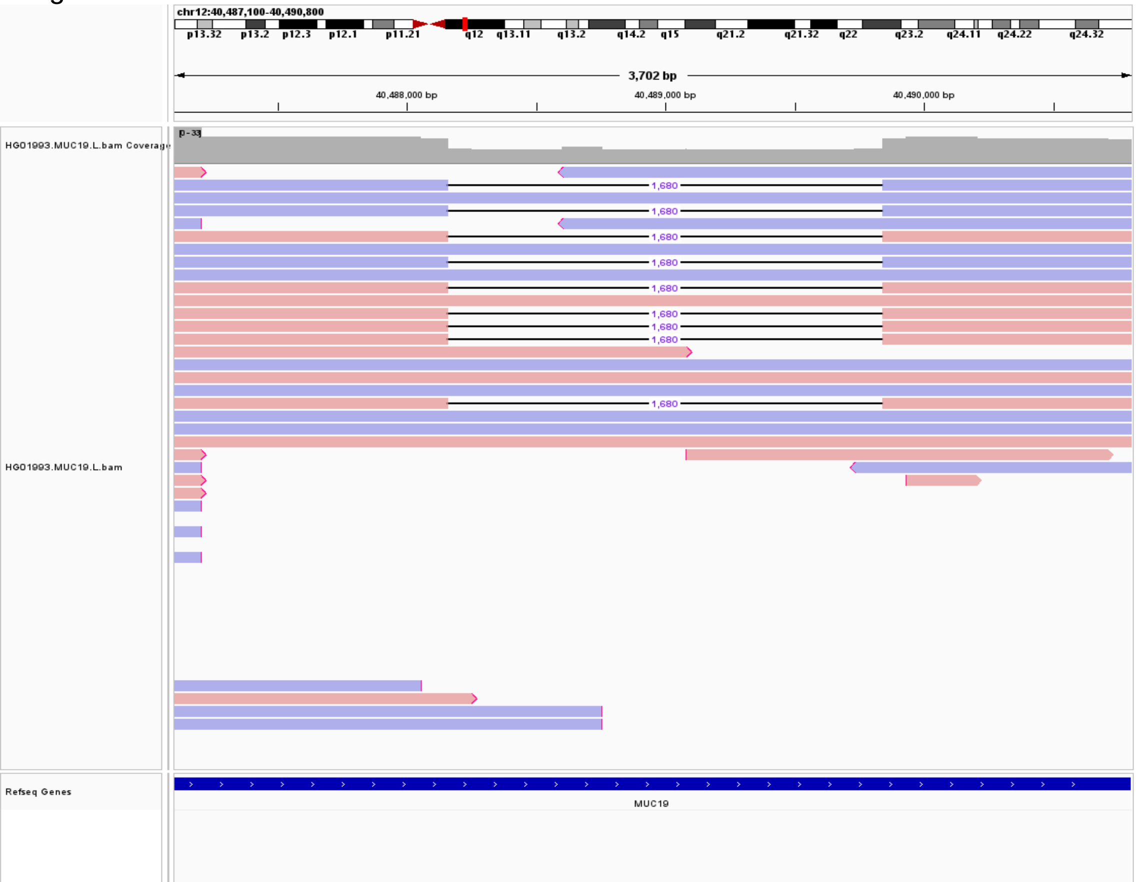

HG02004

Short−reads

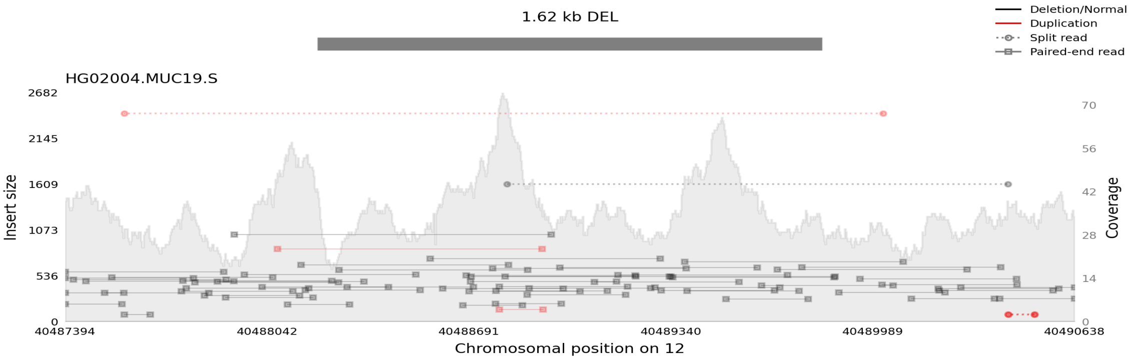

HG02004

Long−reads

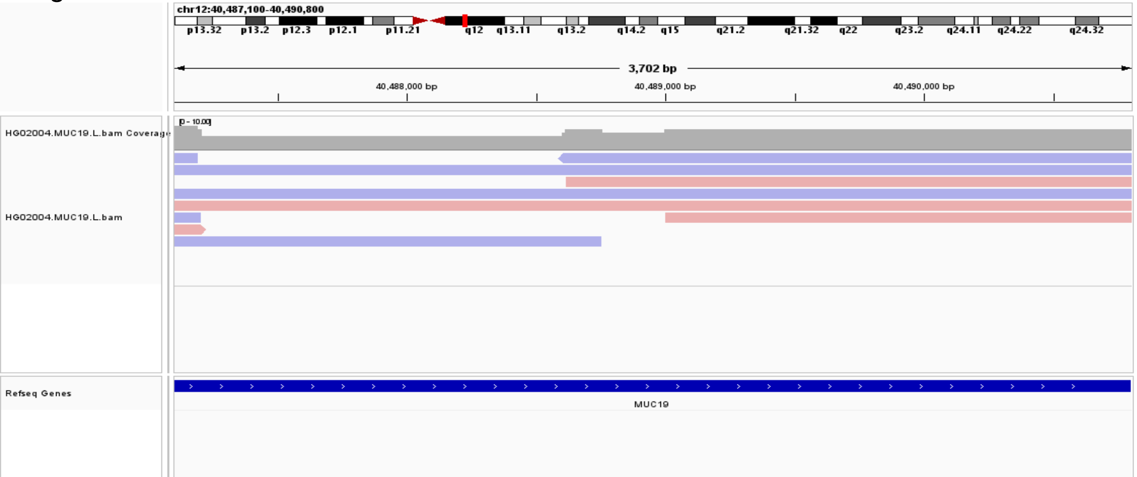

HG02011

Short−reads

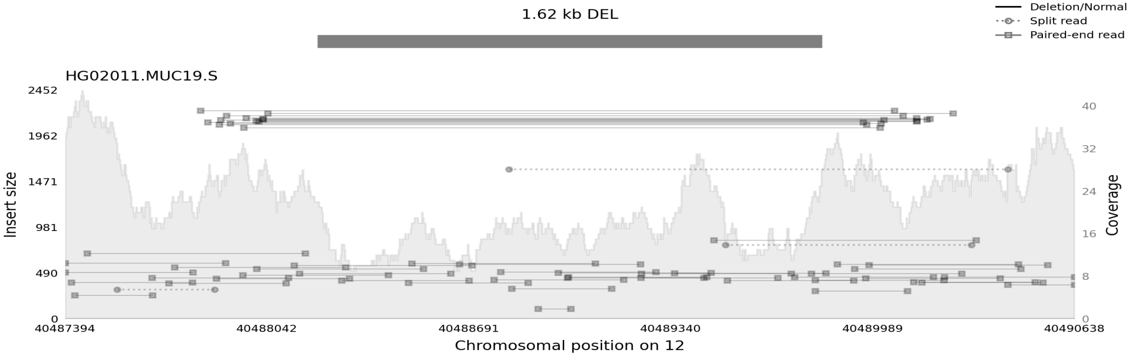

HG02011

Long−reads

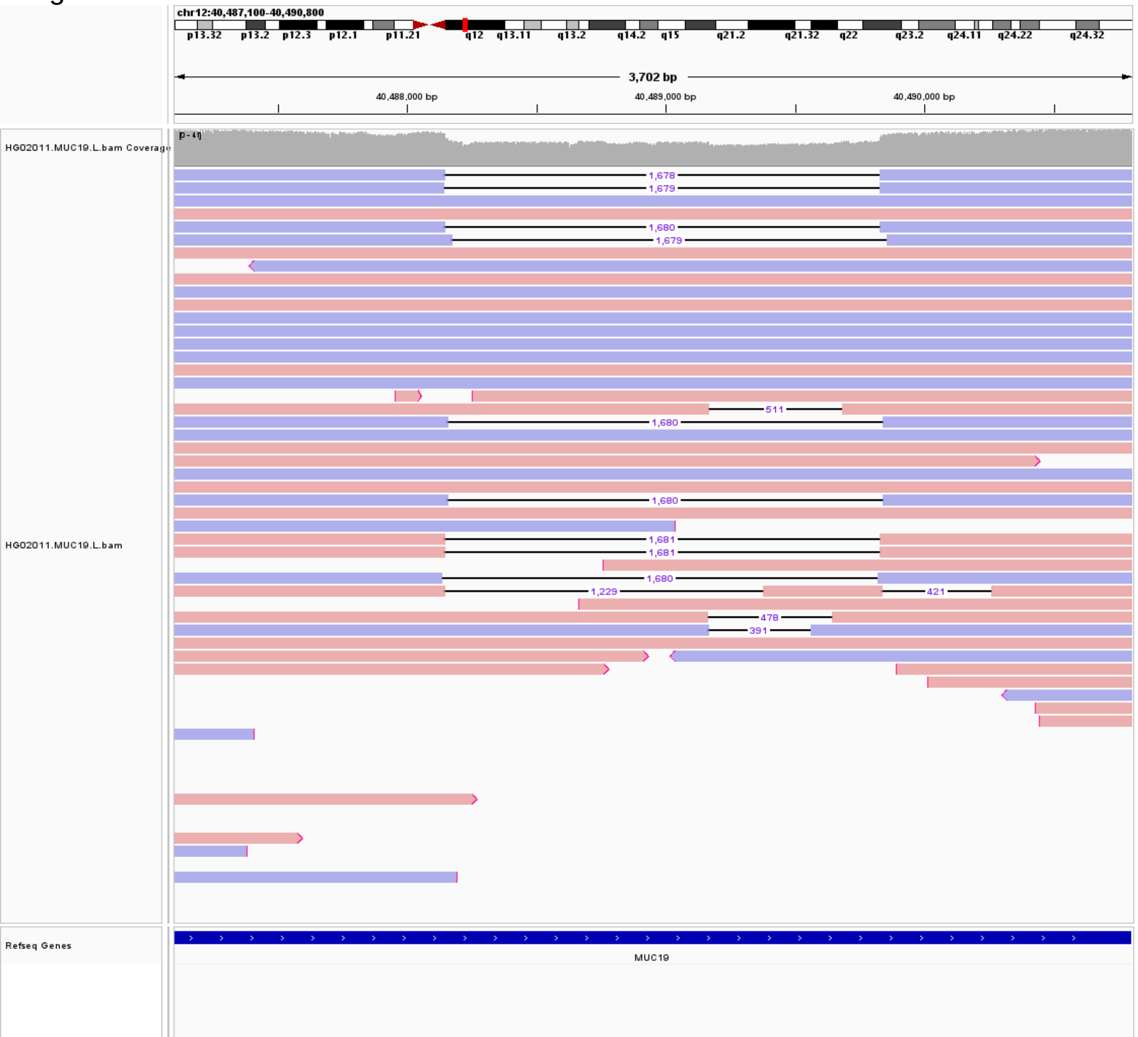

HG02015
Short−reads

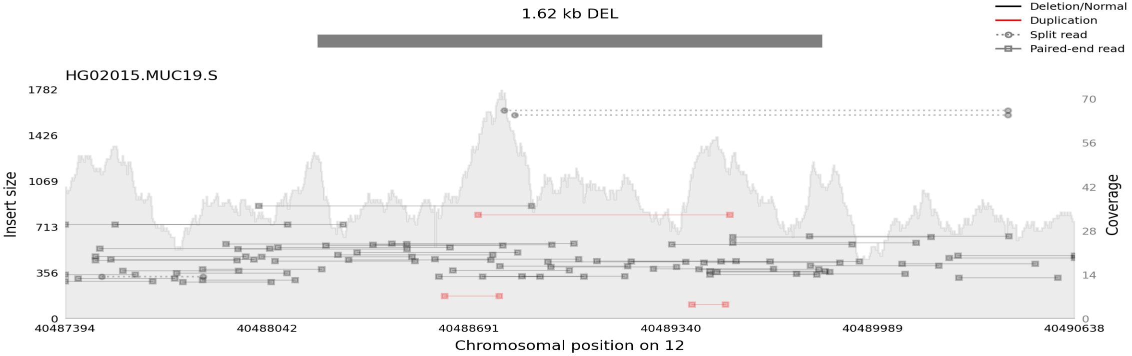

HG02015
Long−reads

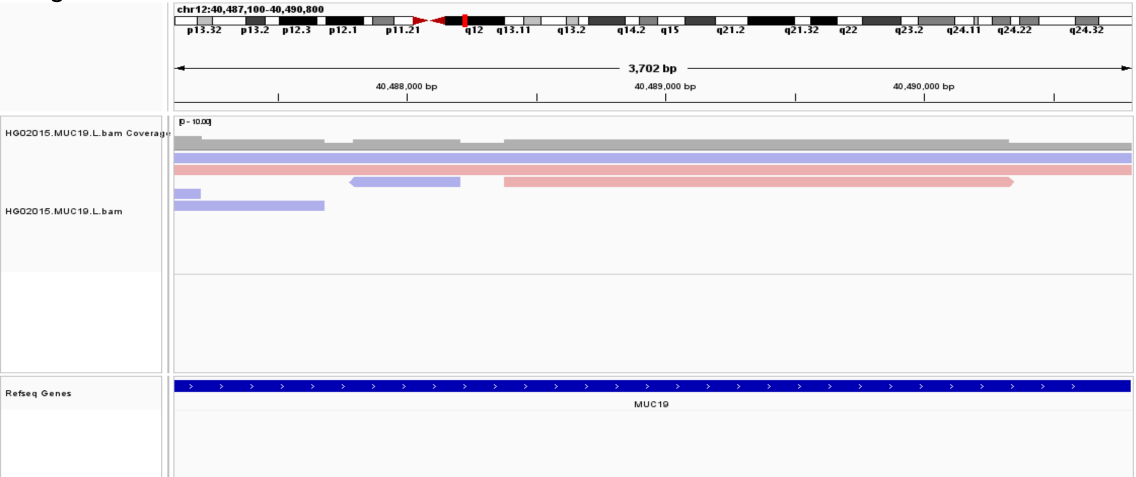

HG02027

Short−reads

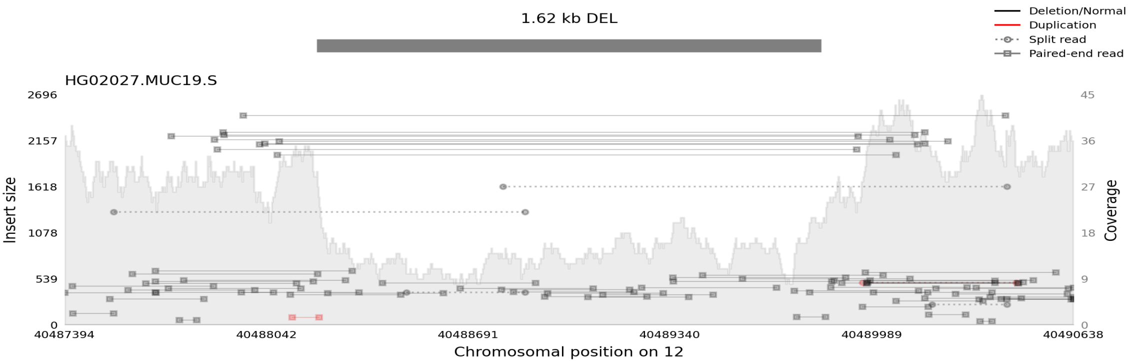

HG02027

Long−reads

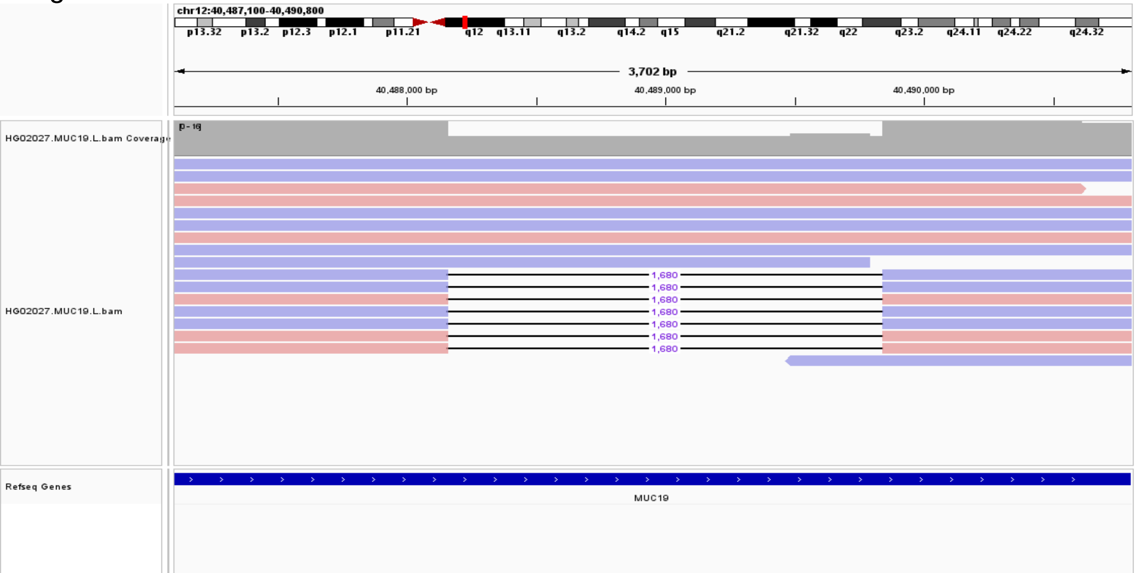

HG02040

Short−reads

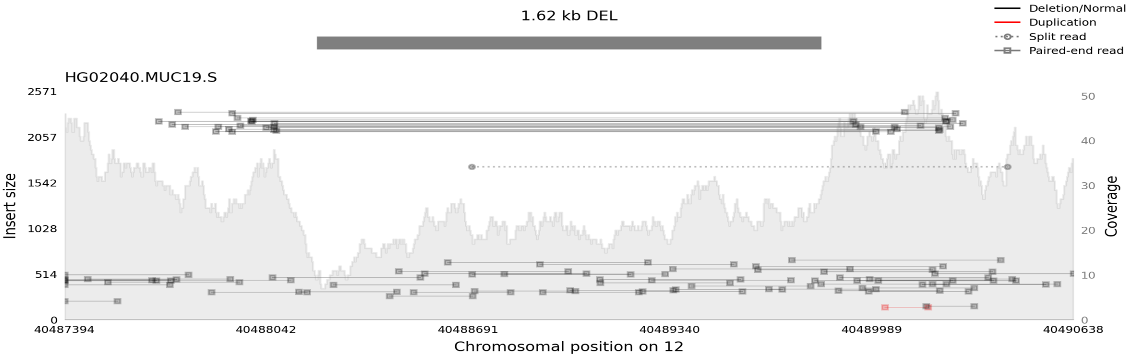

HG02040

Long−reads

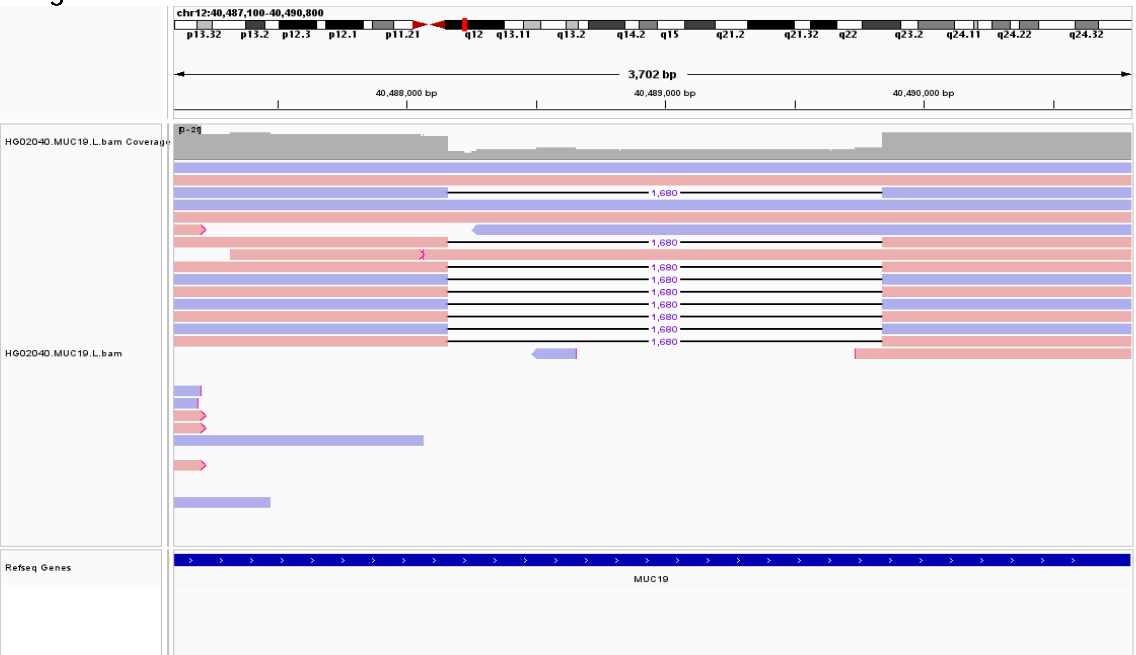

HG02056

Short−reads

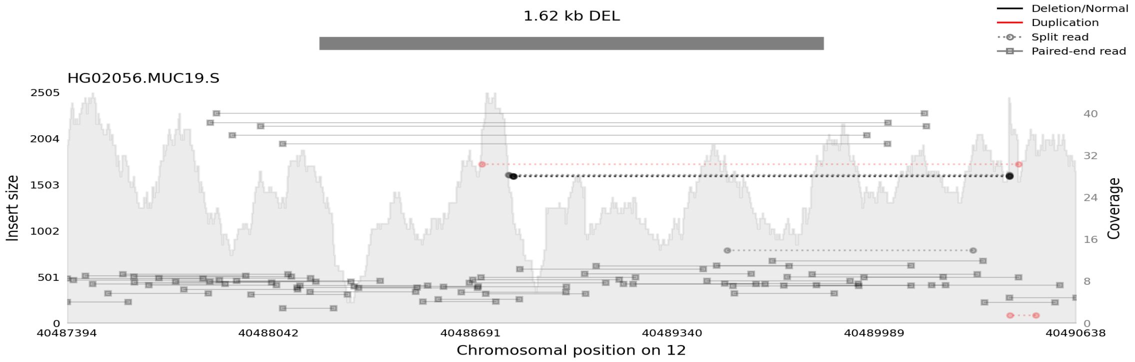

HG02056

Long−reads

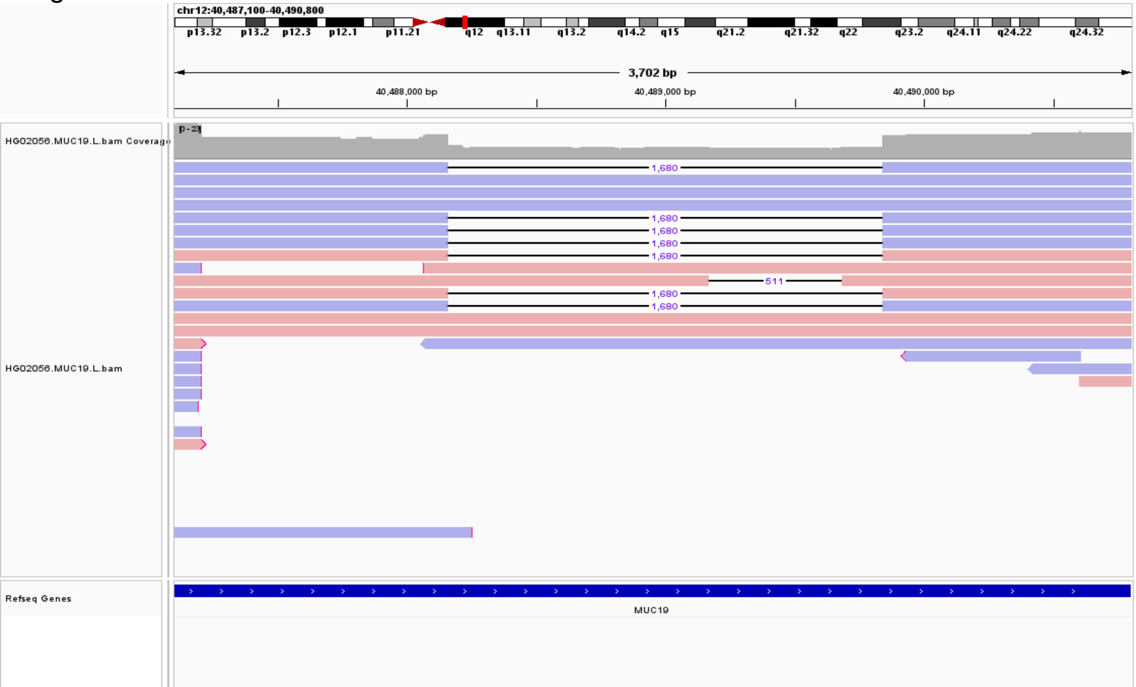

HG02059

Short−reads

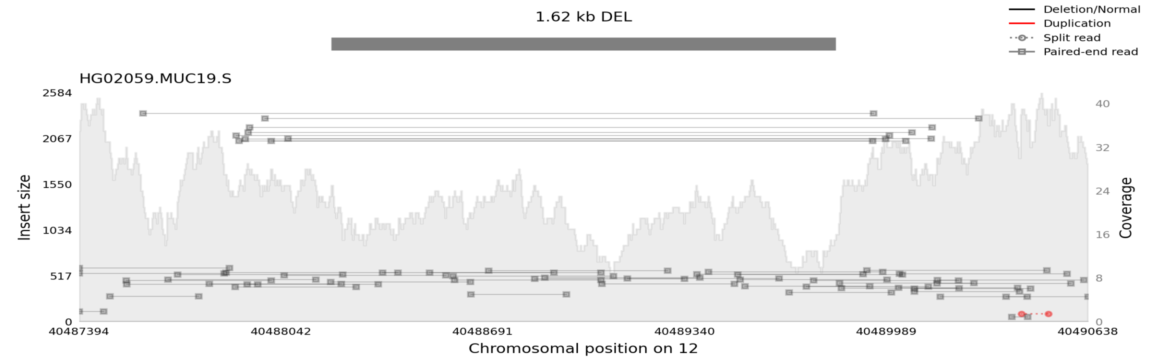

HG02059

Long−reads

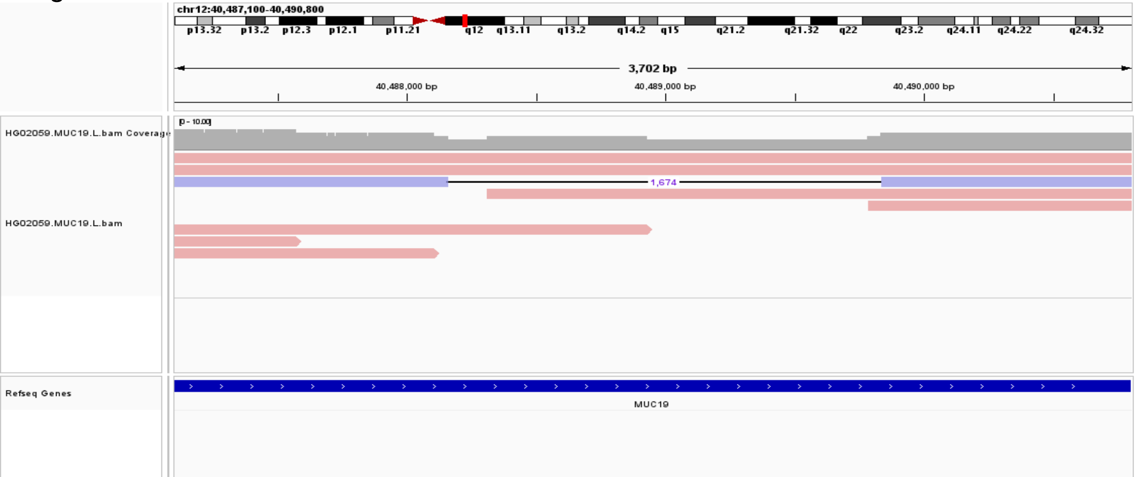

HG02071

Short−reads

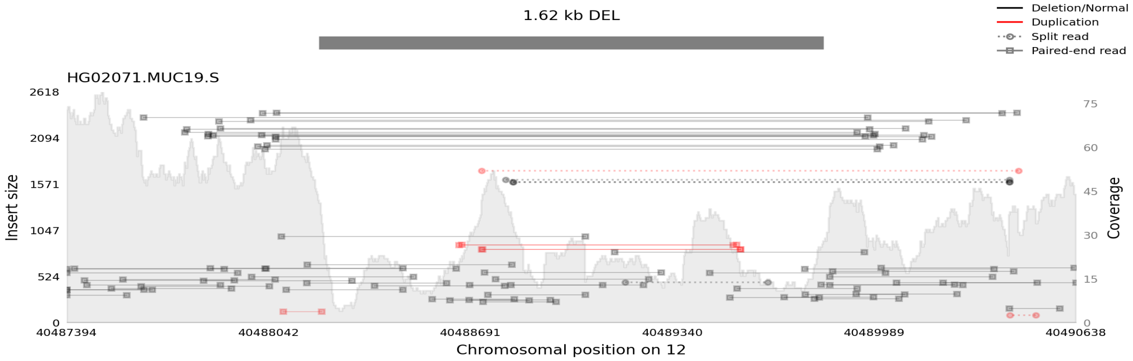

HG02071

Long−reads

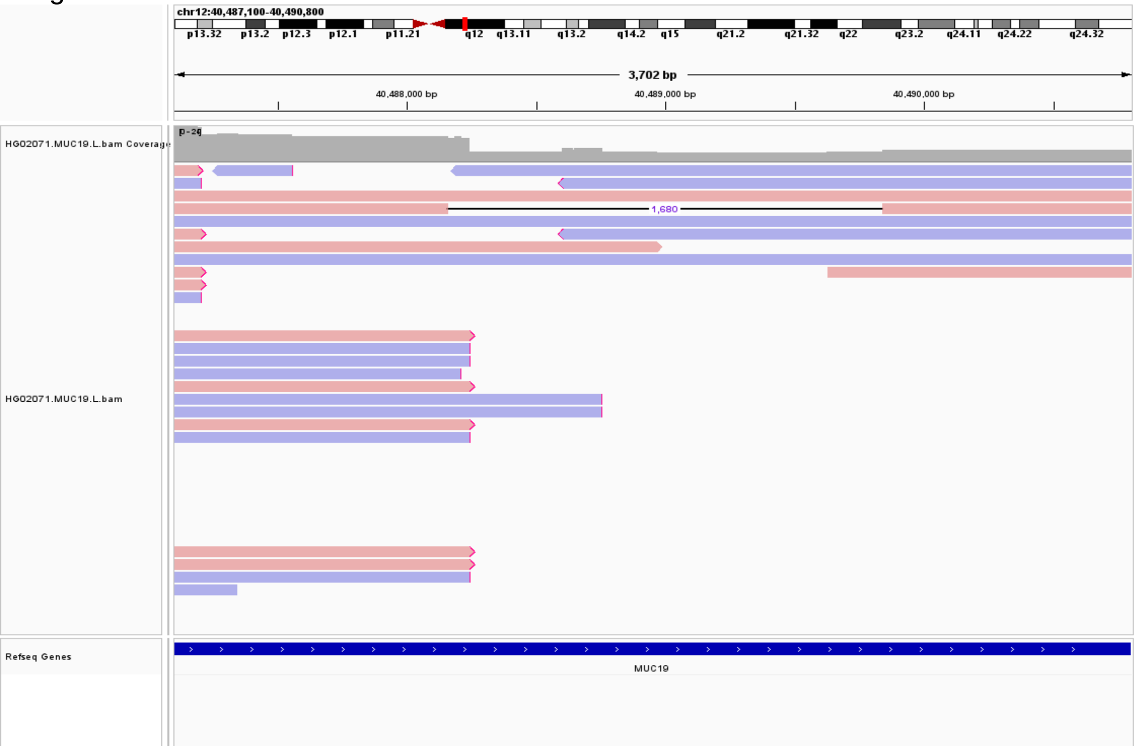

HG02074

Short−reads

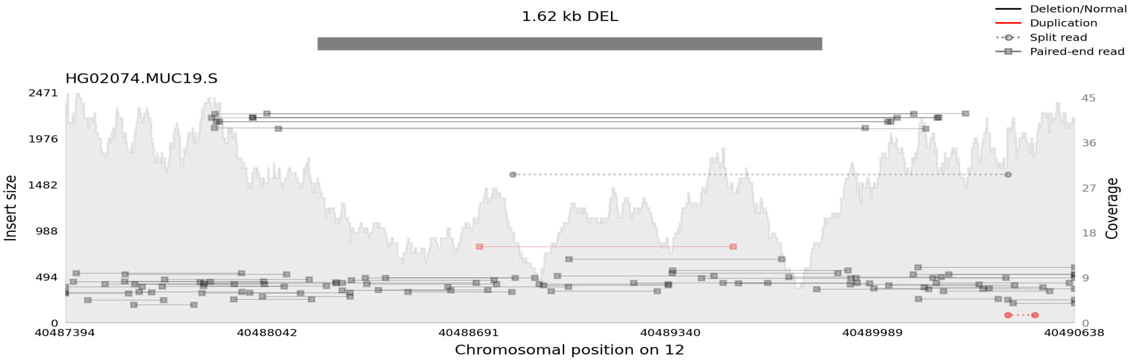

HG02074

Long−reads

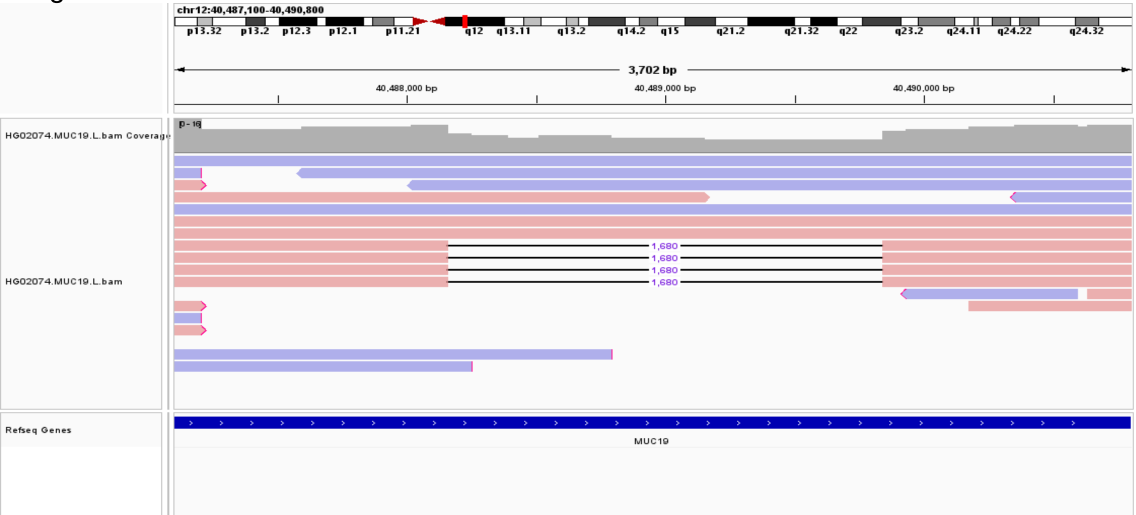

HG02083

Short−reads

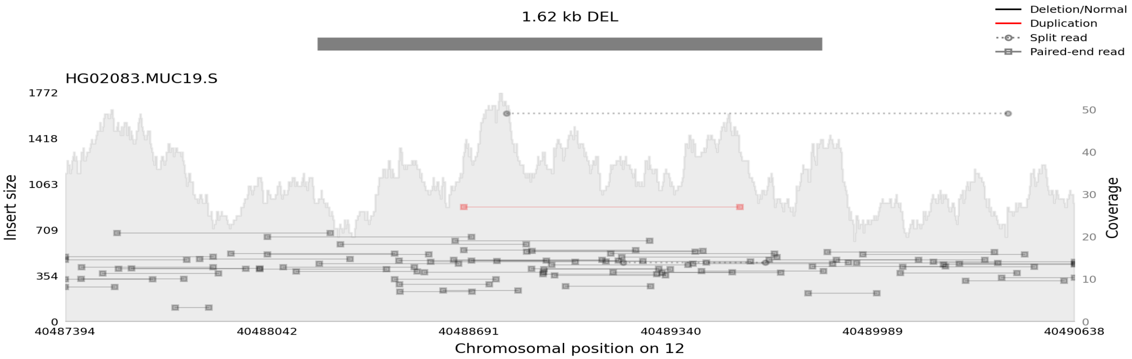

HG02083

Long−reads

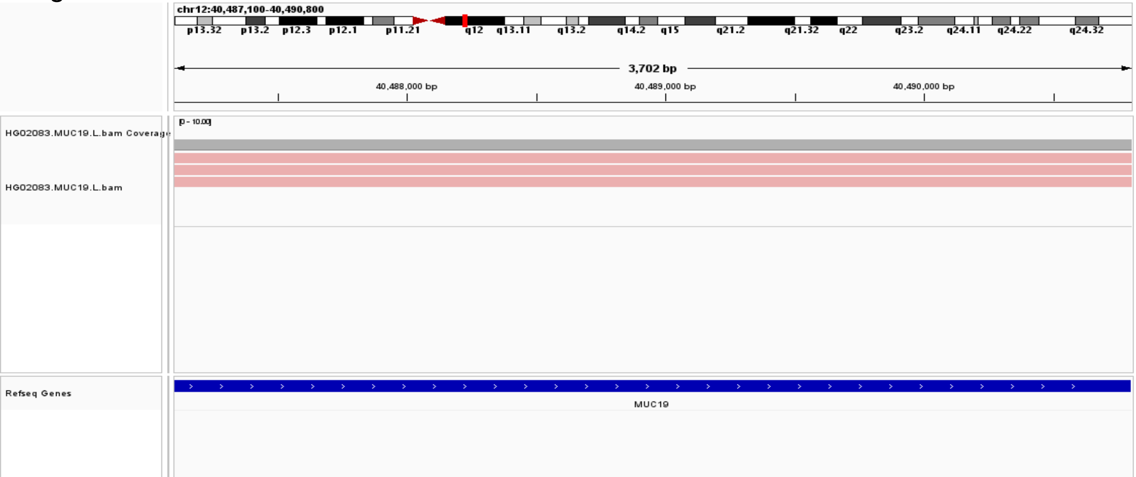

HG02106

Short−reads

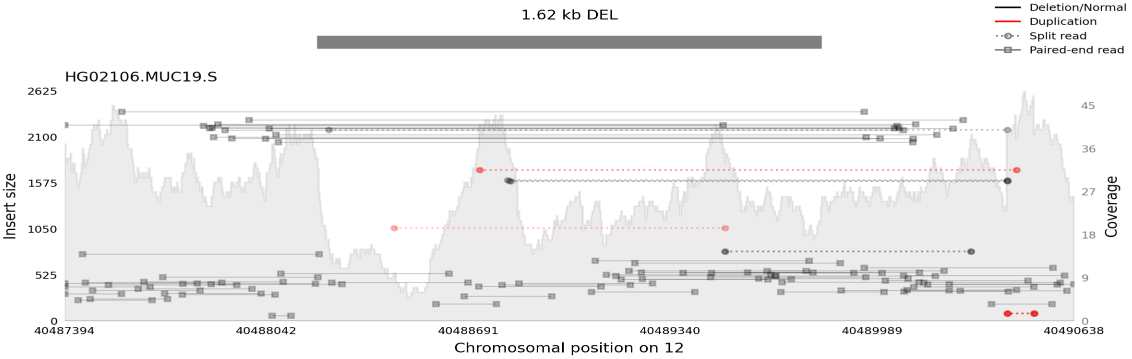

HG02106

Long−reads

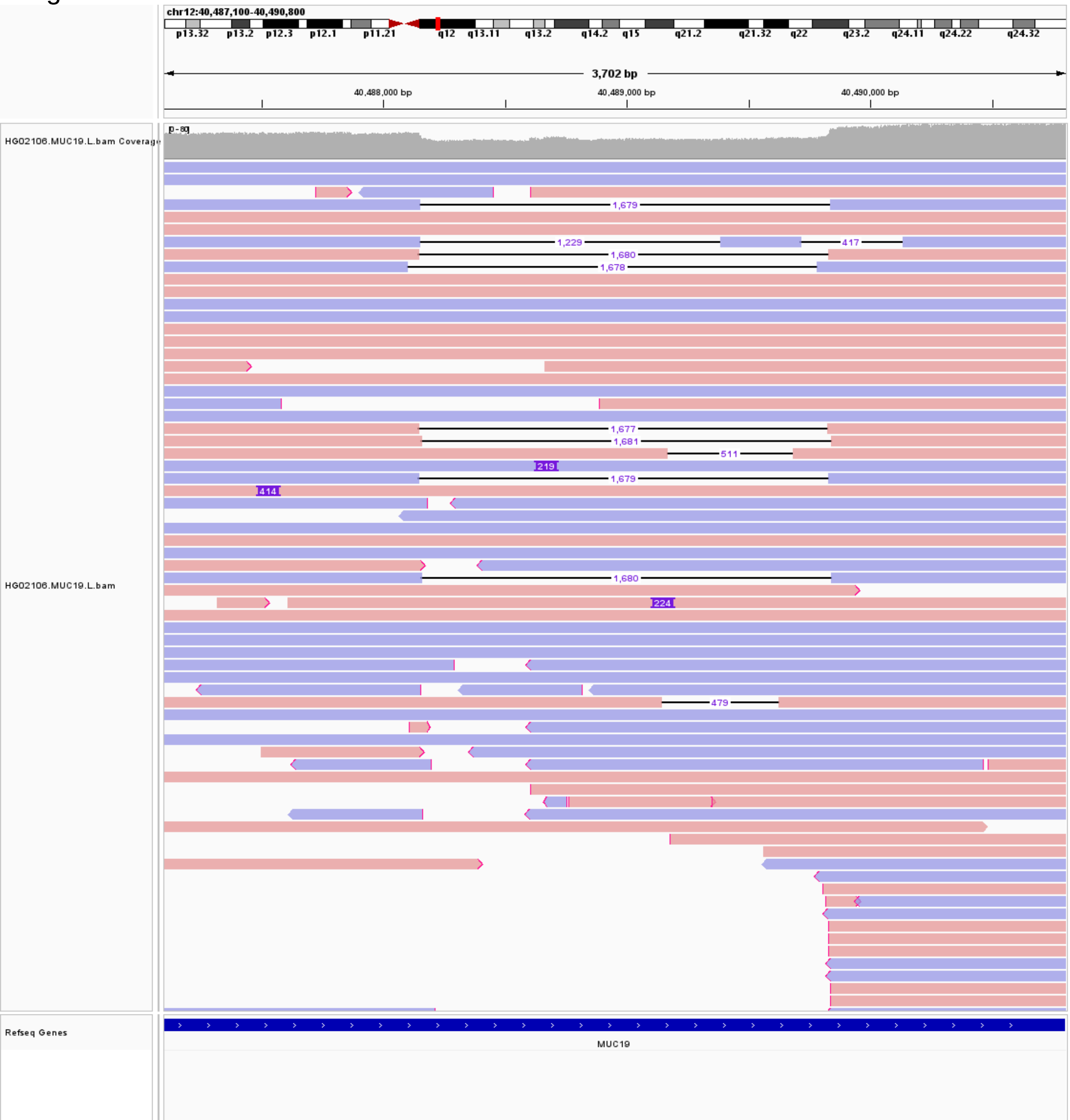

HG02129

Short−reads

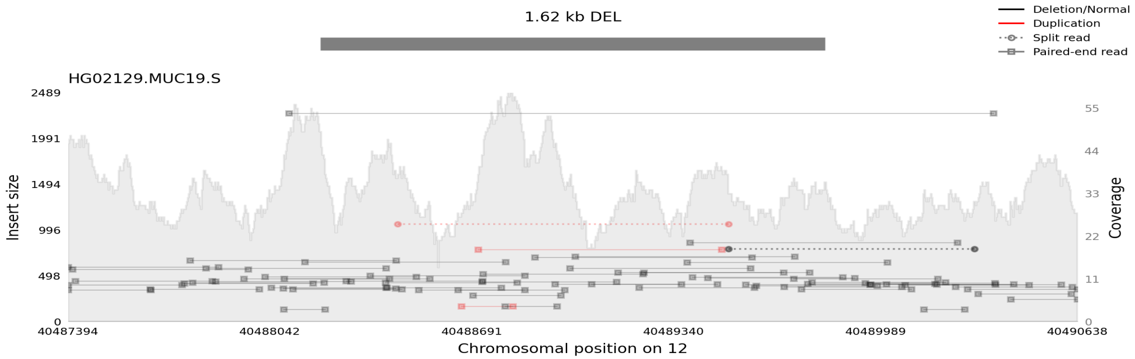

HG02129

Long−reads

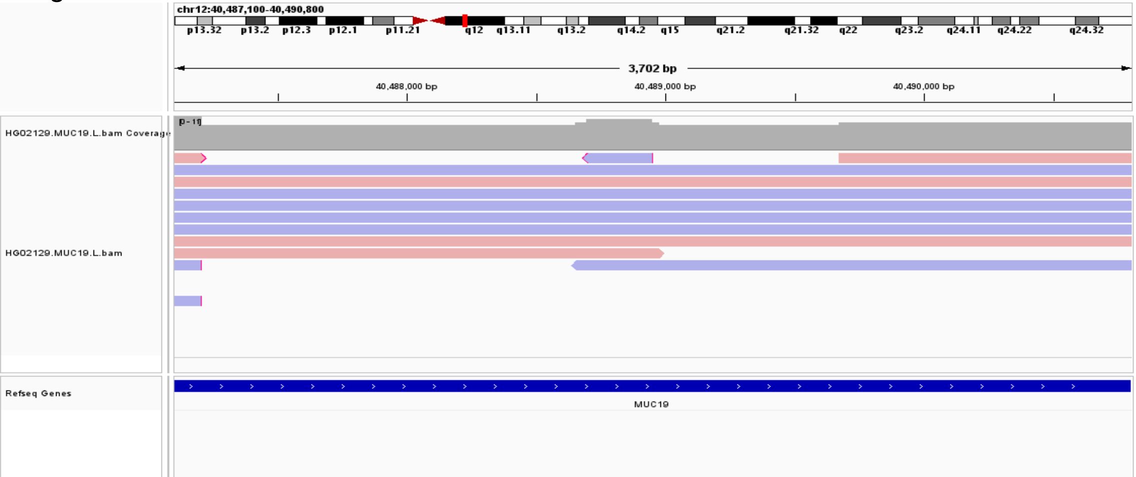

HG02132
Short−reads

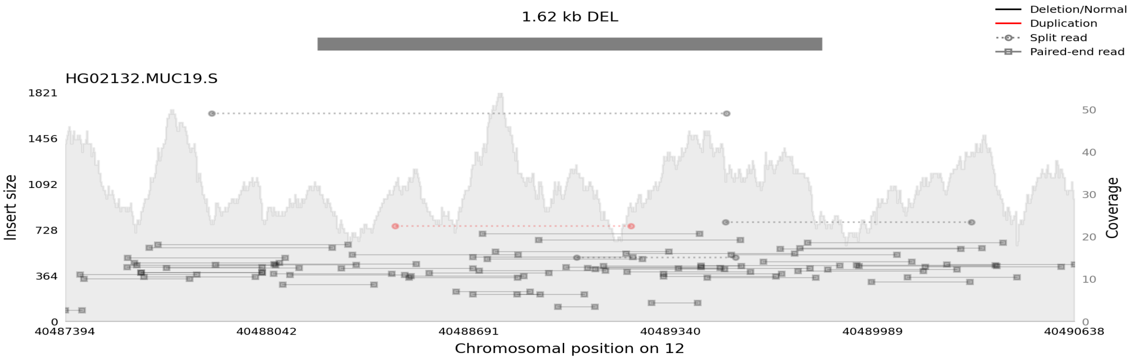

HG02132
Long−reads

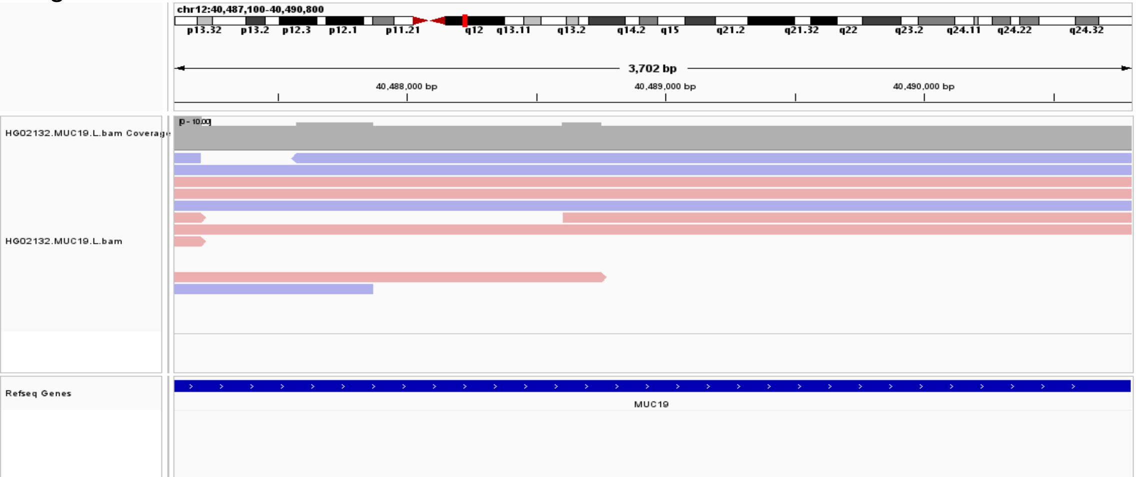

HG02135

Short−reads

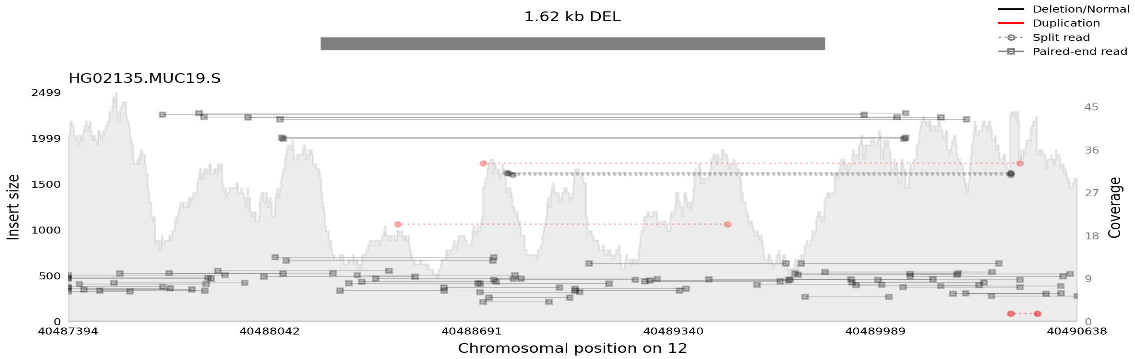

HG02135

Long−reads

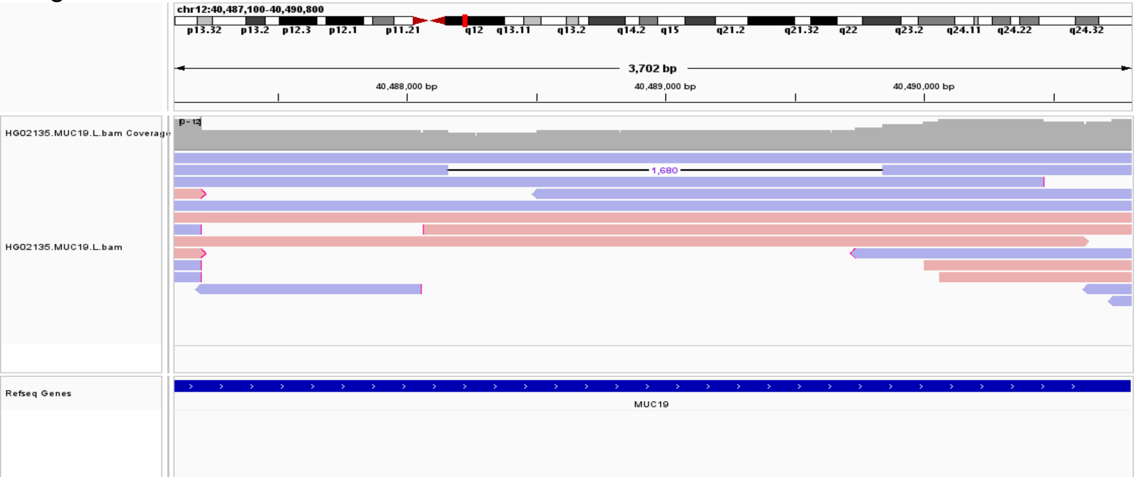

HG02155

Short−reads

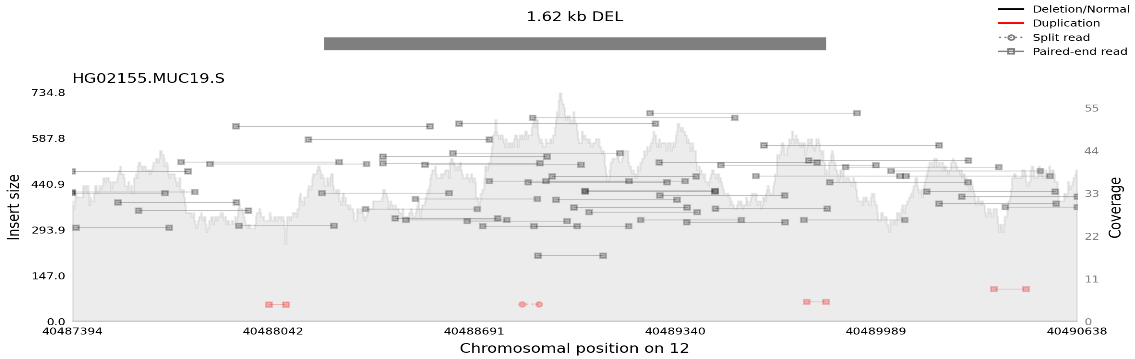

HG02155

Long−reads

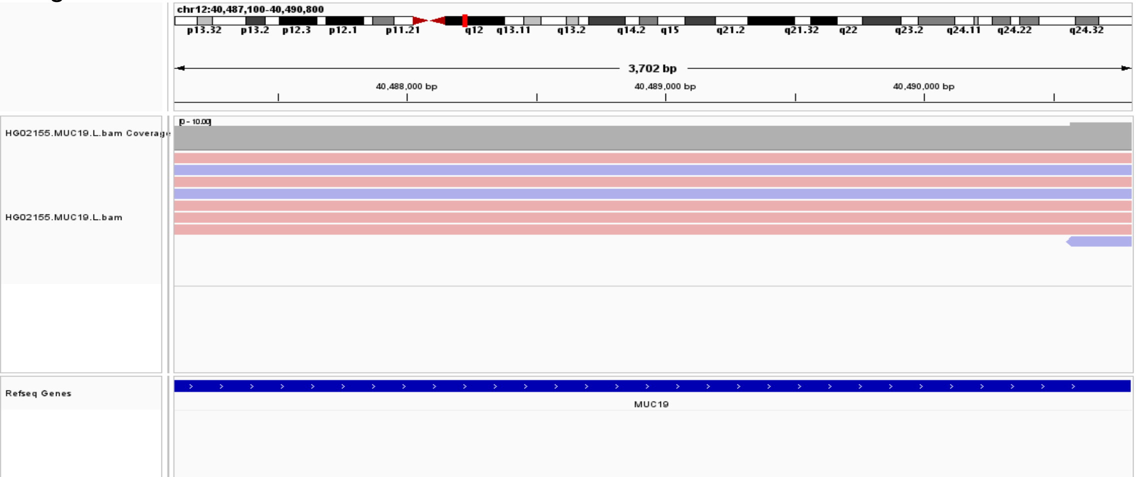

HG02165

Short−reads

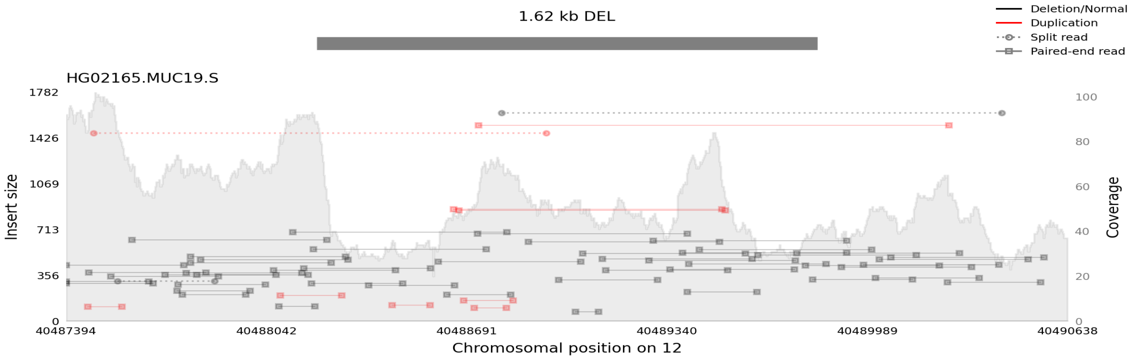

HG02165

Long−reads

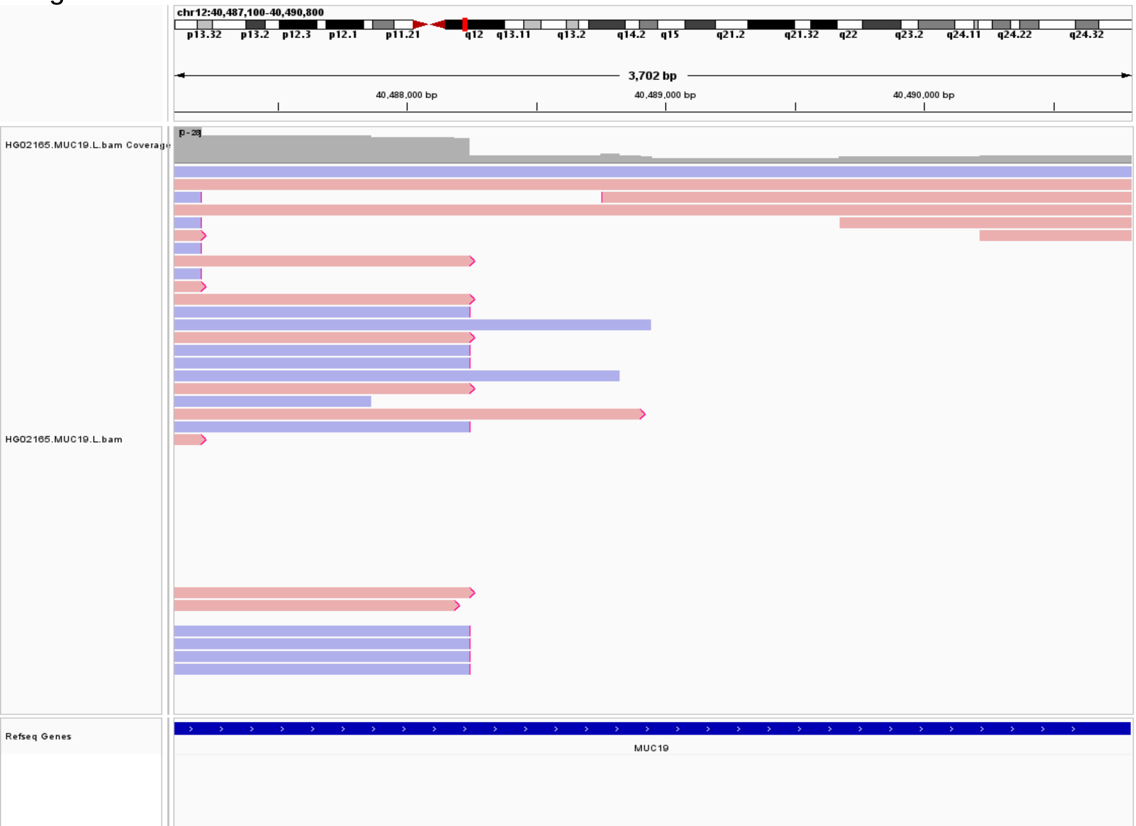

HG02257

Short−reads

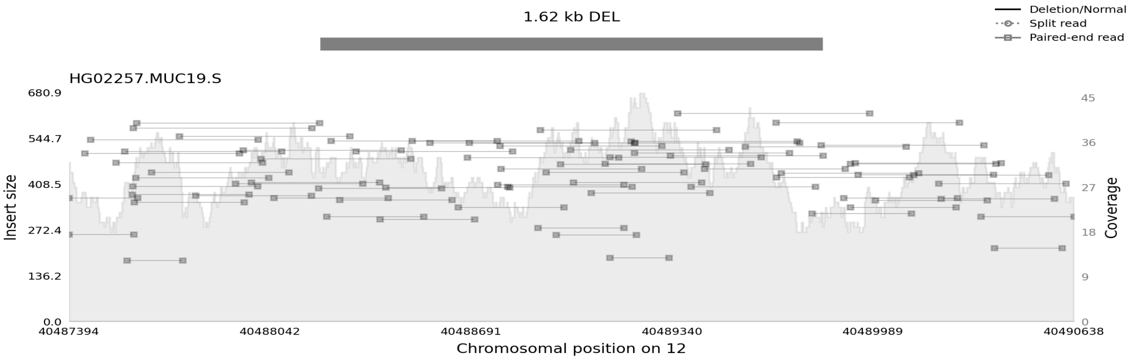

HG02257

Long−reads

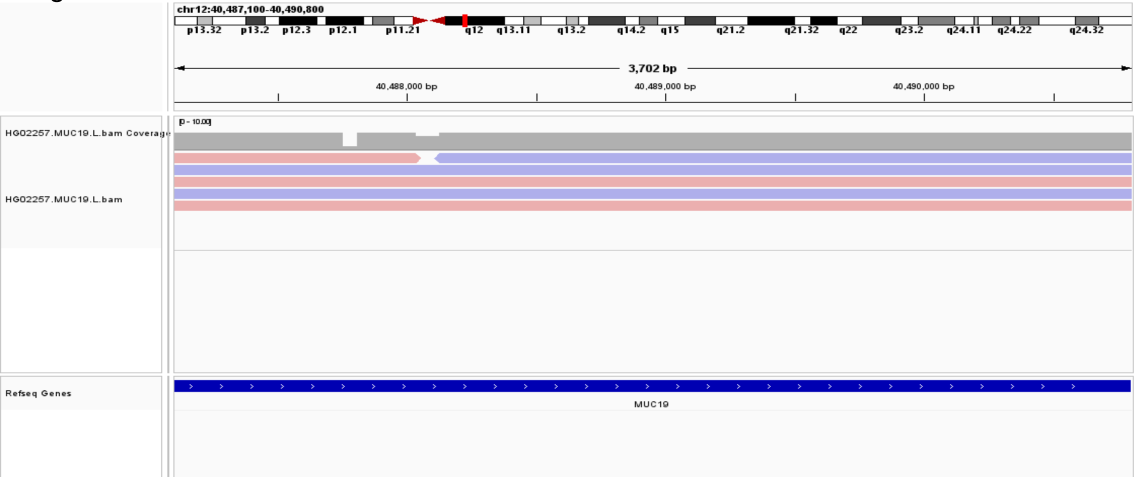

HG02258

Short−reads

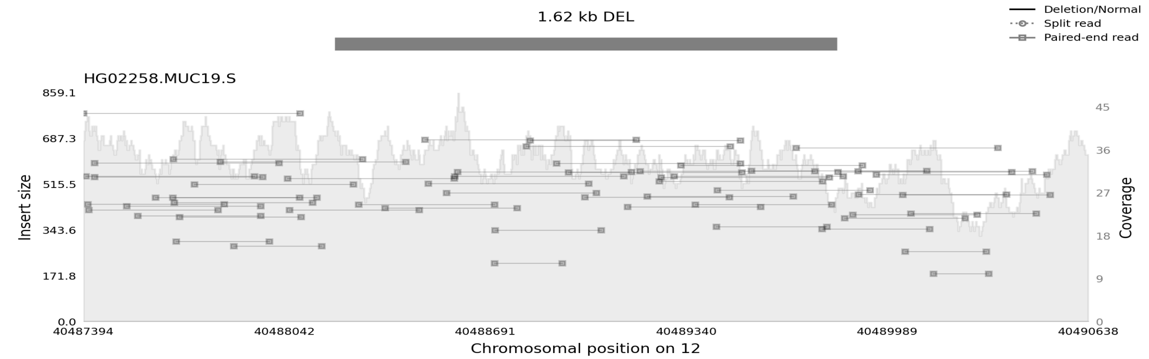

HG02258

Long−reads

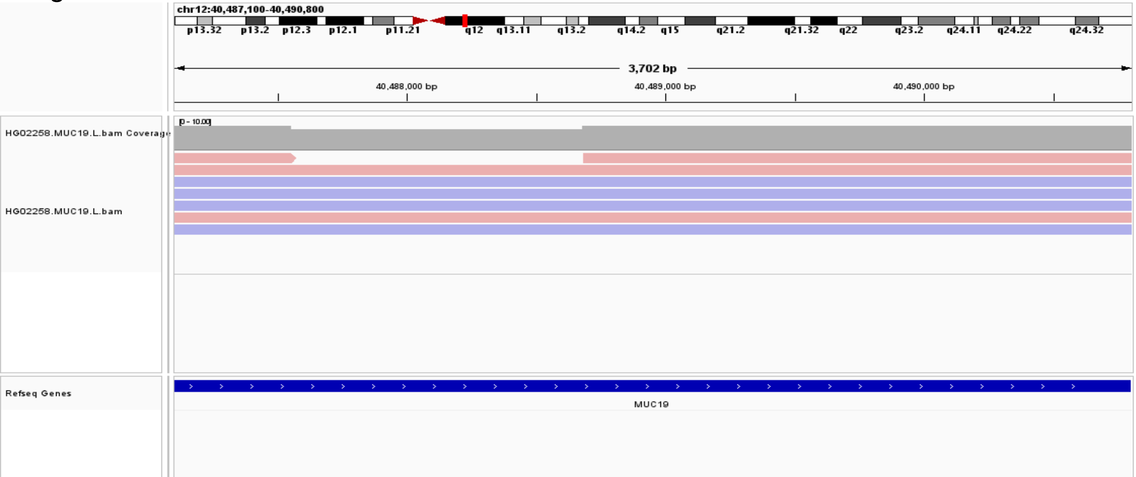

HG02273
Short−reads

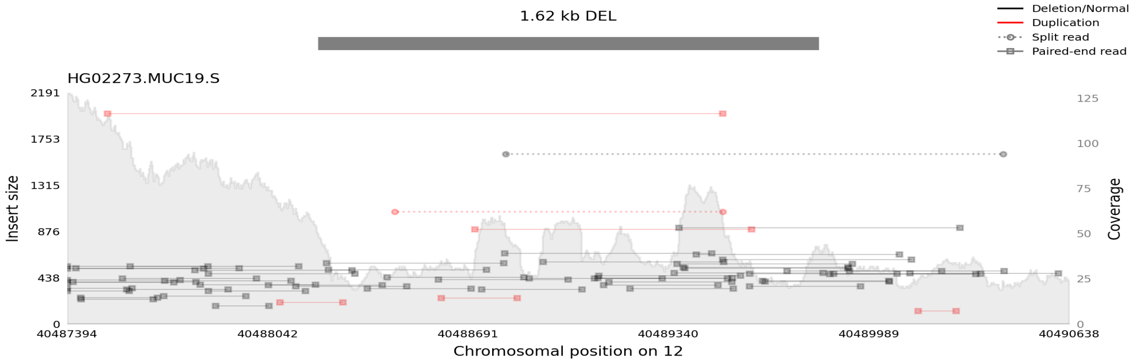

HG02273
Long−reads

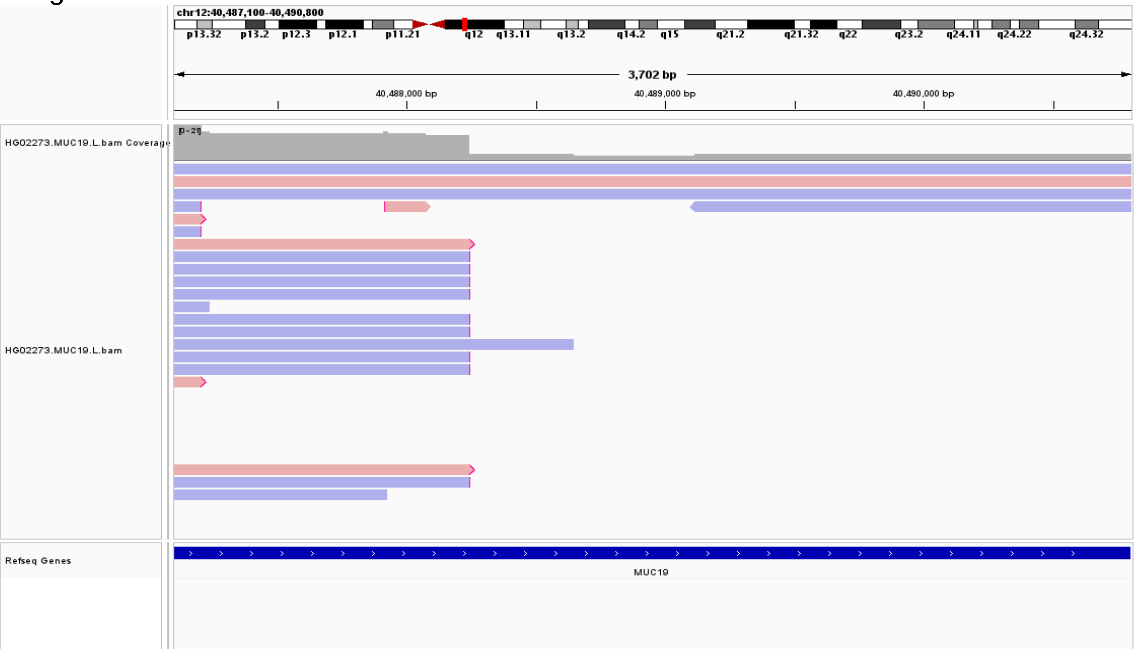

HG02280

Short−reads

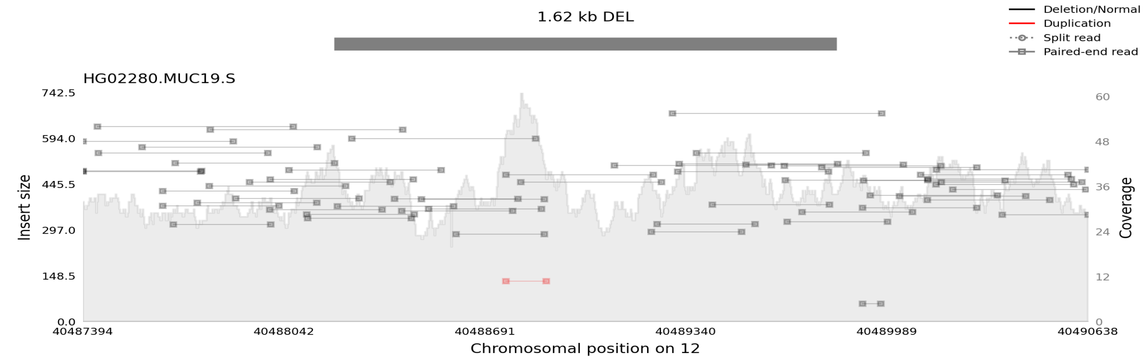

HG02280

Long−reads

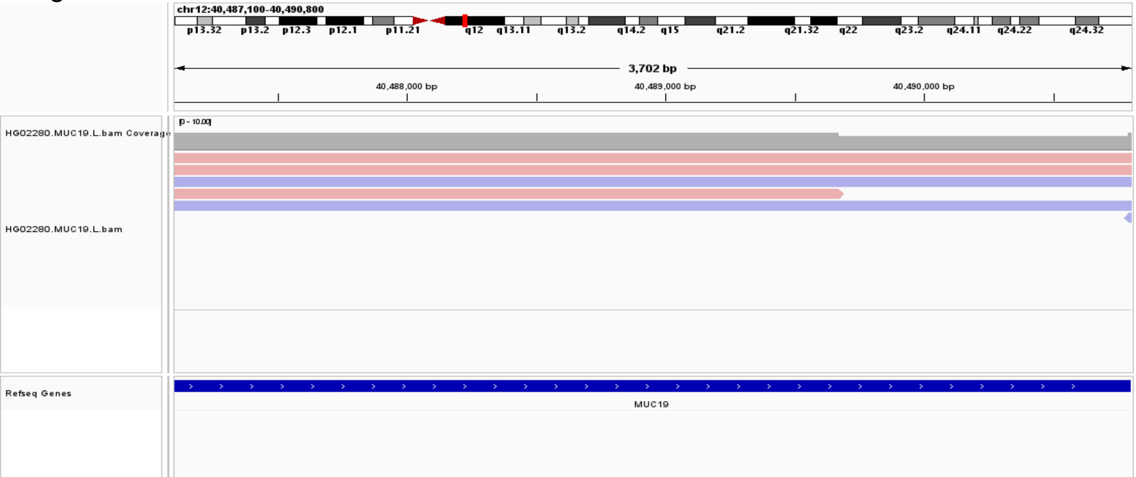

HG02293

Short−reads

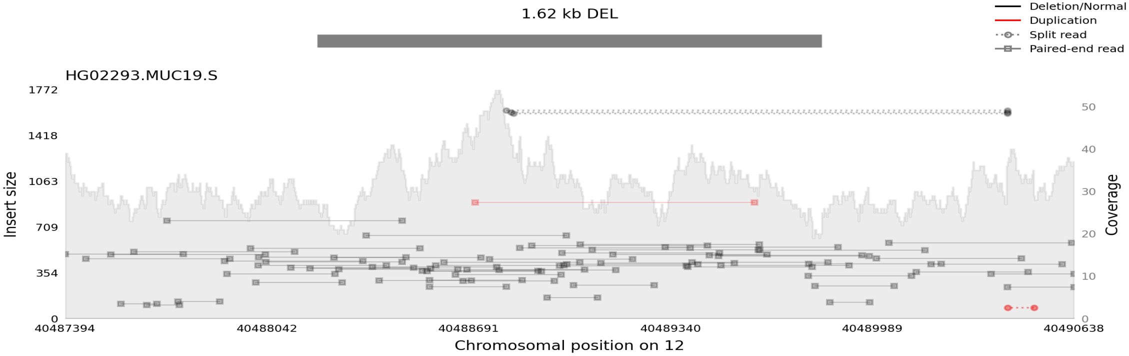

HG02293

Long−reads

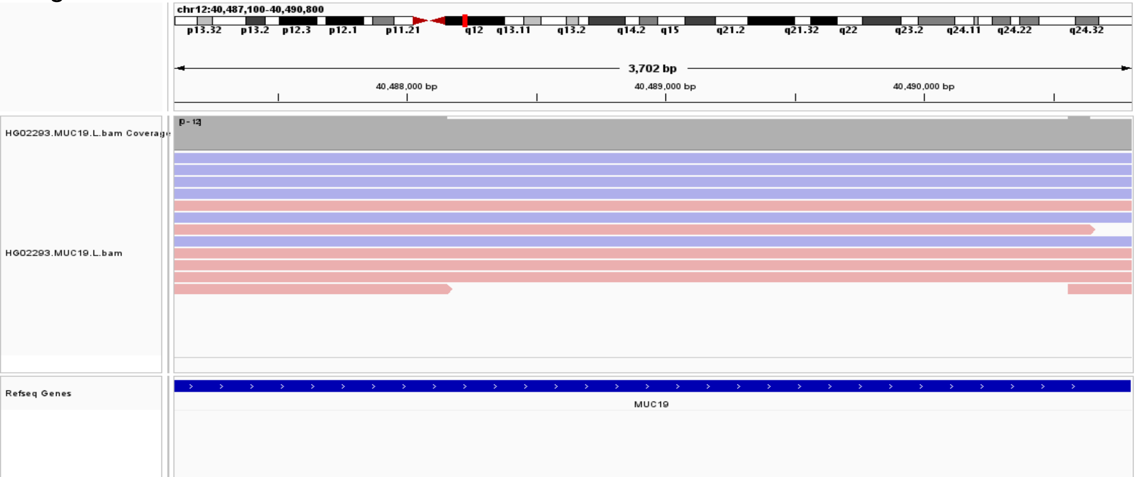

HG02300

Short−reads

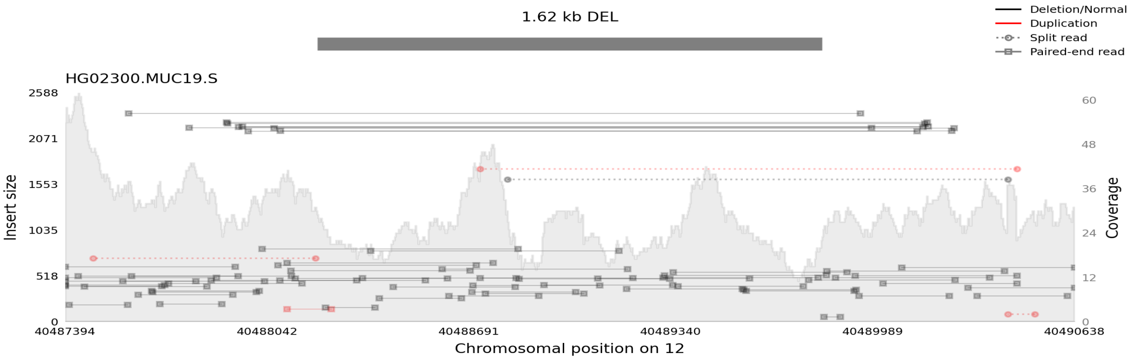

HG02300

Long−reads

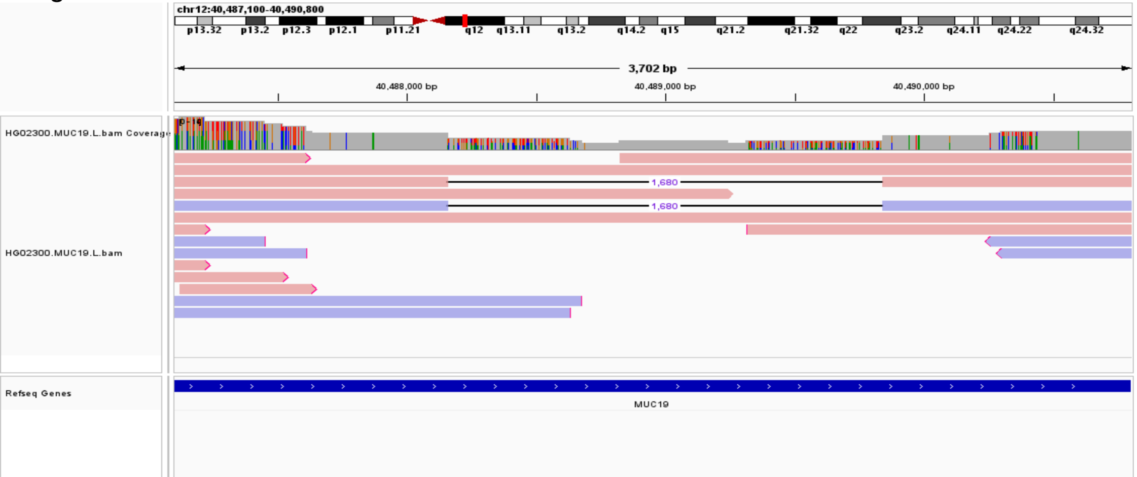

HG02451

Short−reads

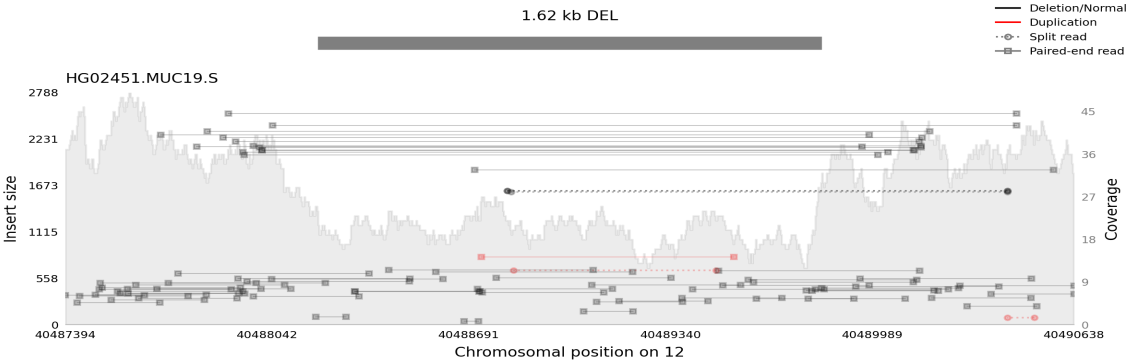

HG02451

Long−reads

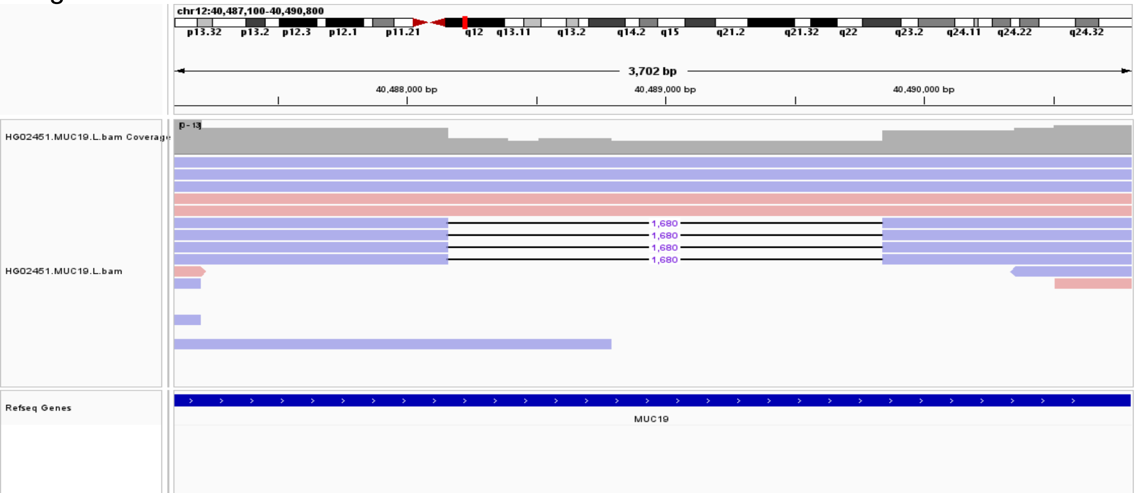

HG02492
Short−reads

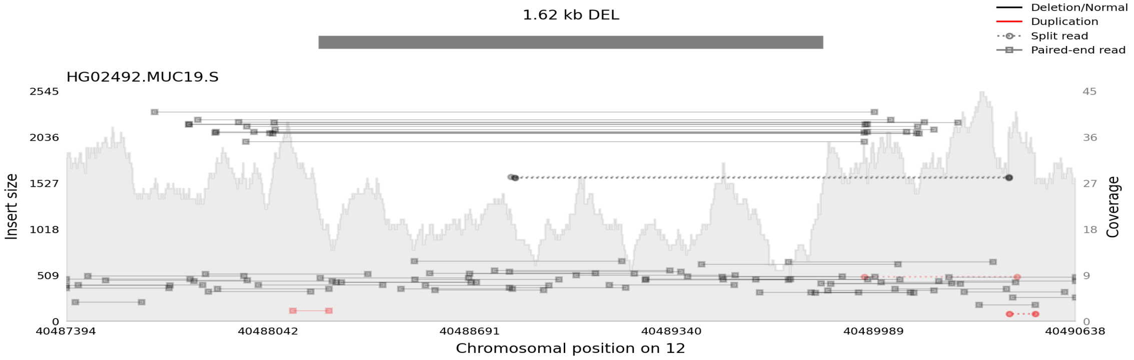

HG02492
Long−reads

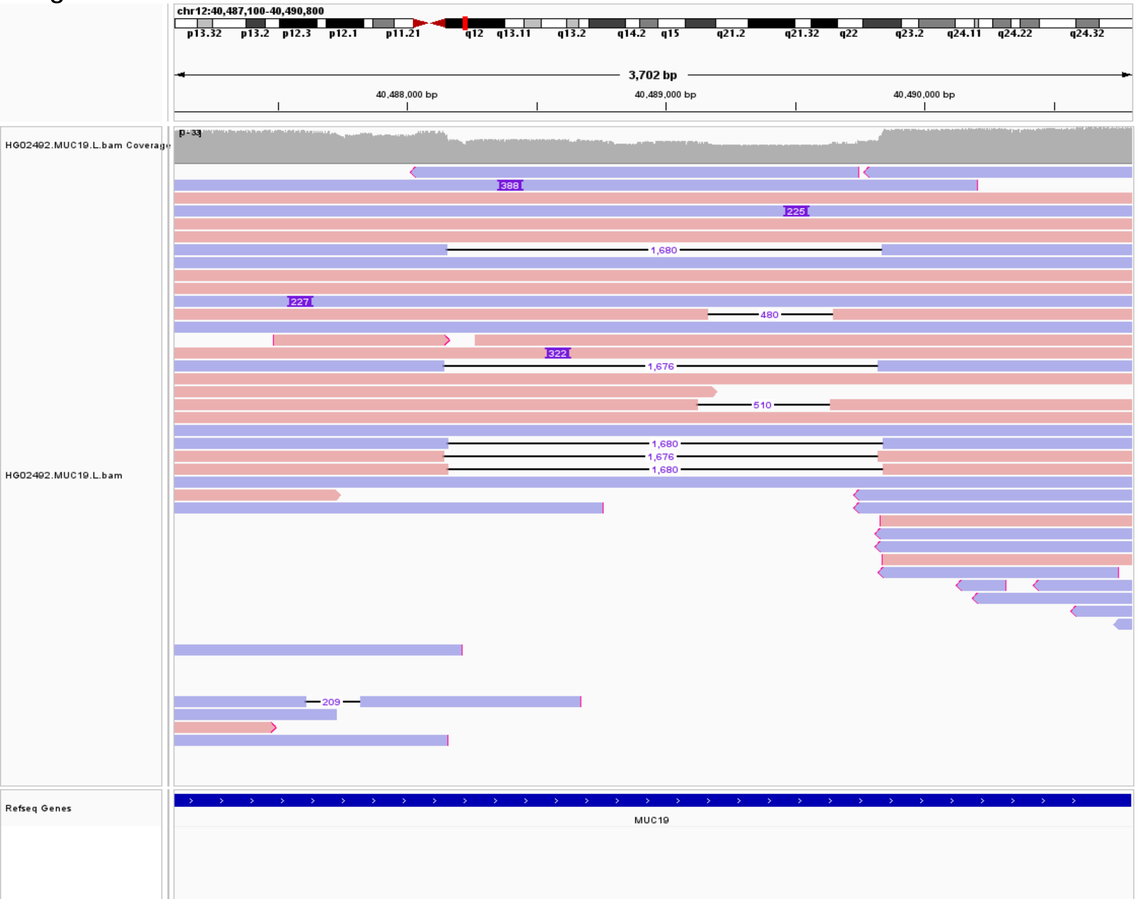

HG02523

Short−reads

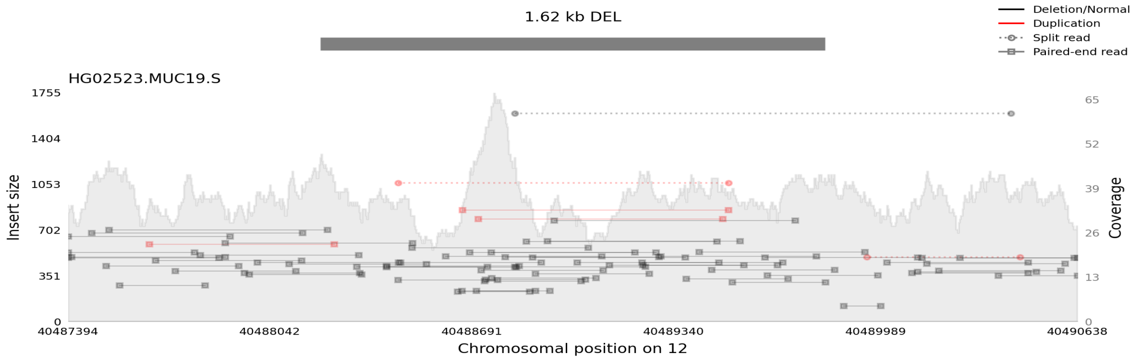

HG02523

Long−reads

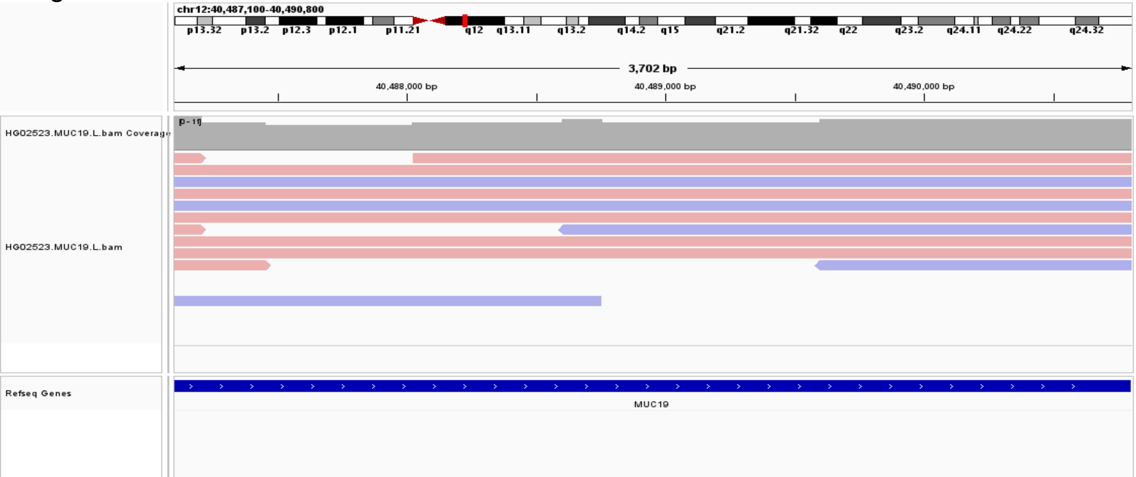

HG02572

Short−reads

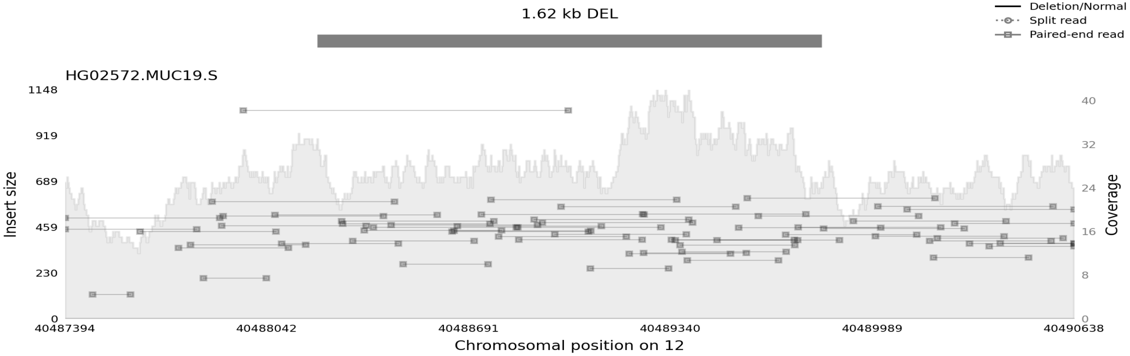

HG02572

Long−reads

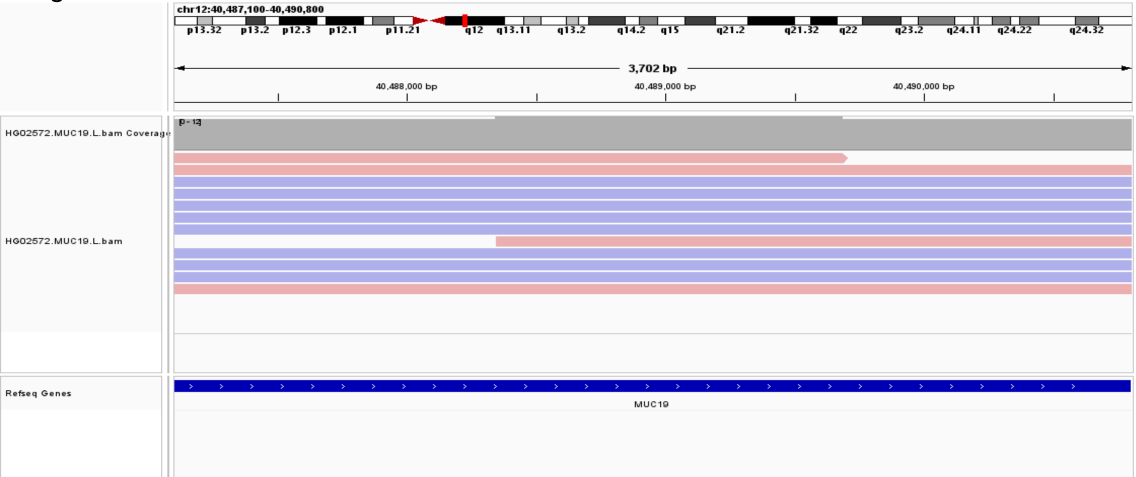

HG02587

Short−reads

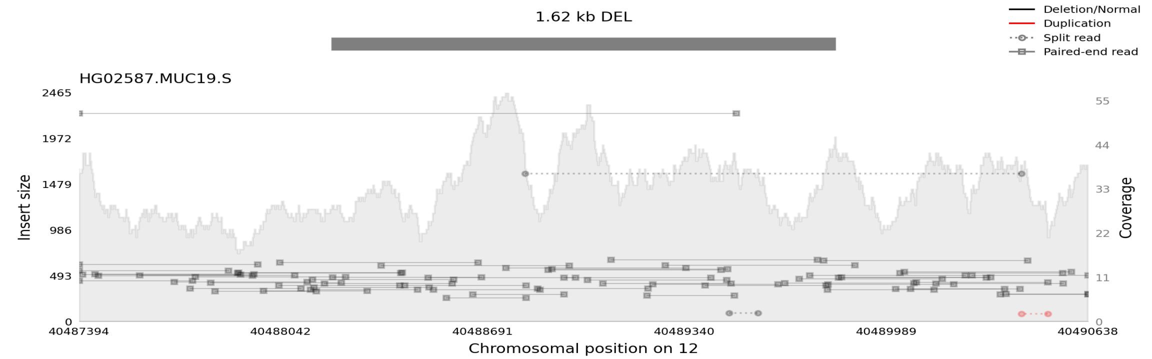

HG02587

Long−reads

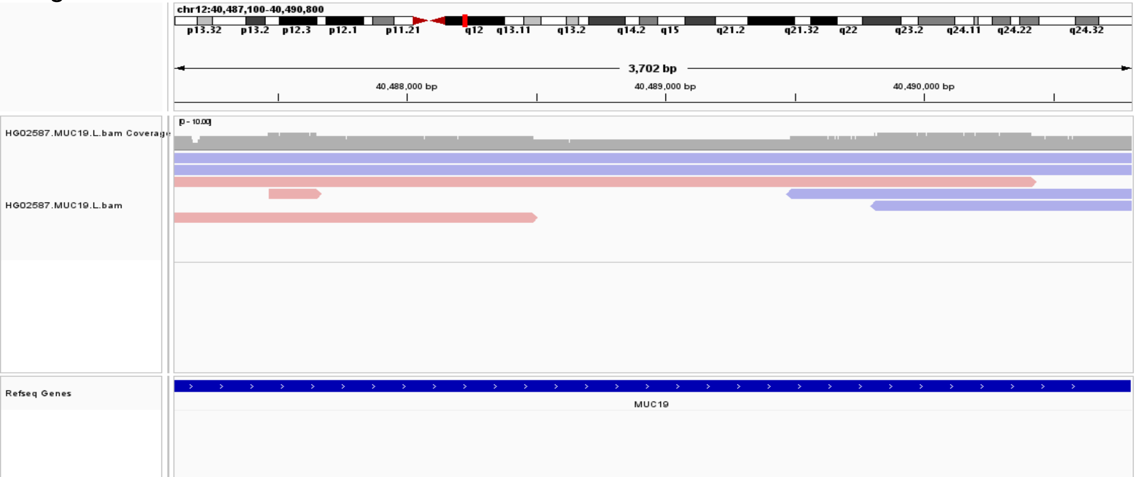

HG02602

Short−reads

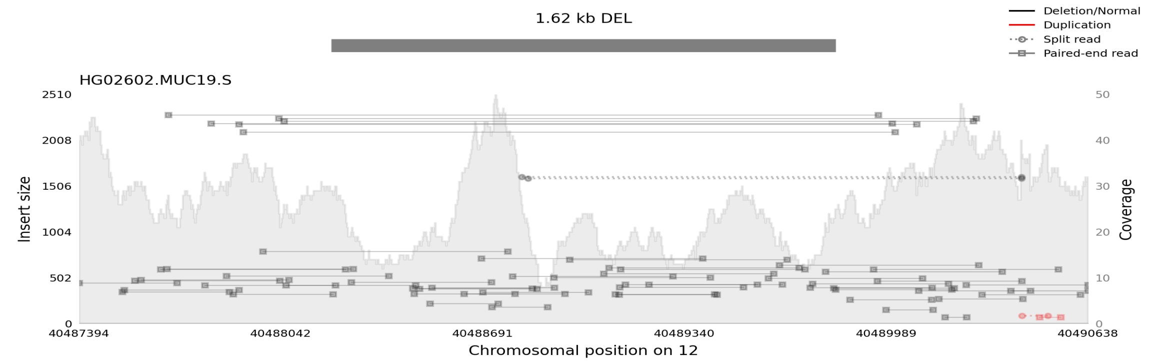

HG02602

Long−reads

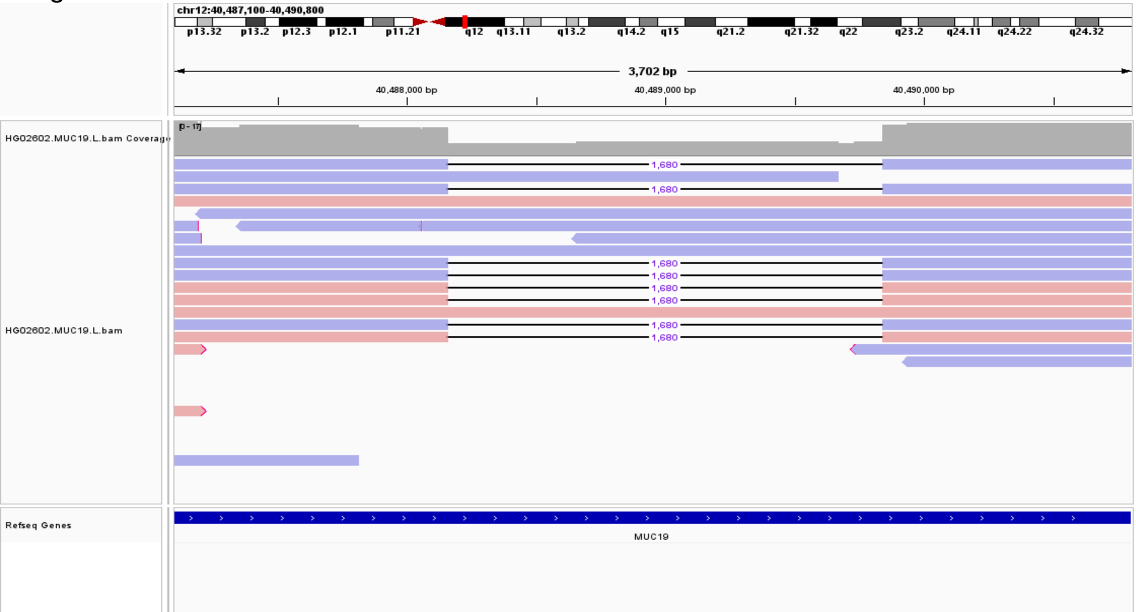

HG02615

Short−reads

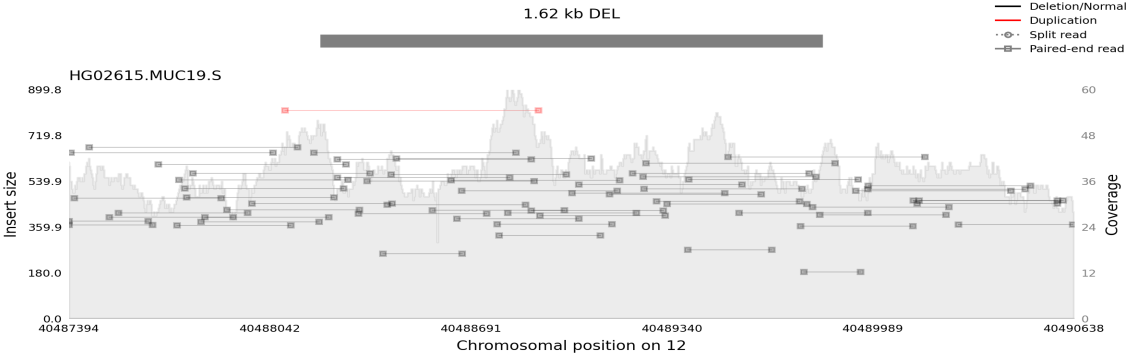

HG02615

Long−reads

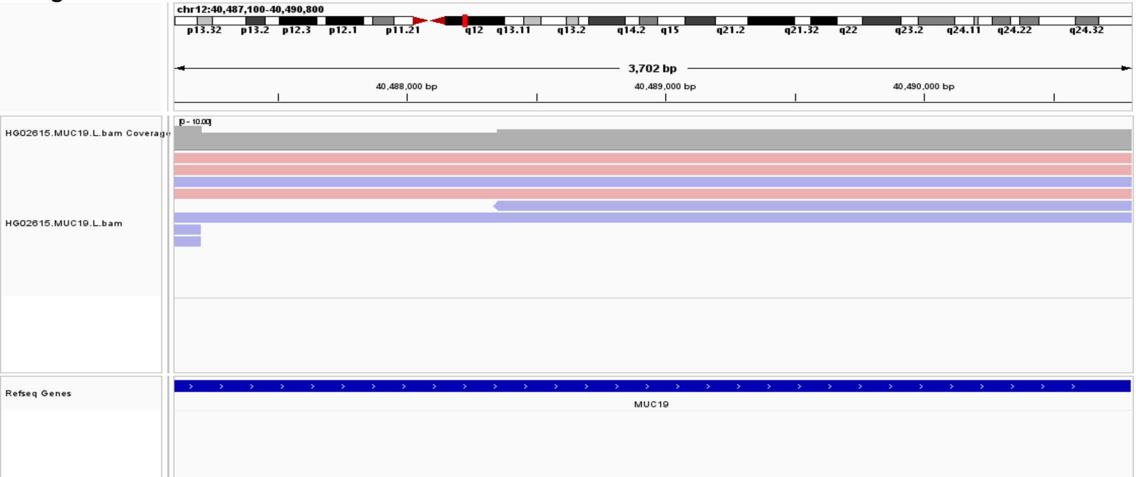

HG02647

Short−reads

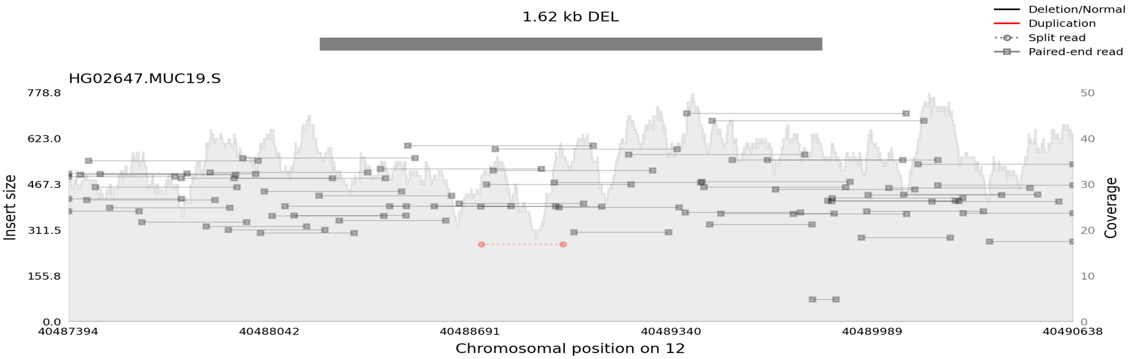

HG02647

Long−reads

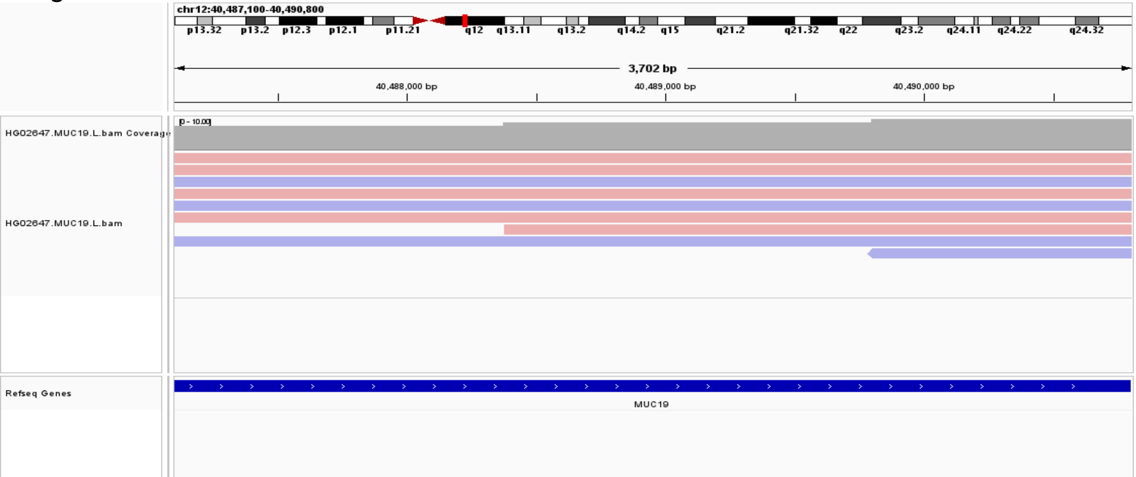

HG02683

Short−reads

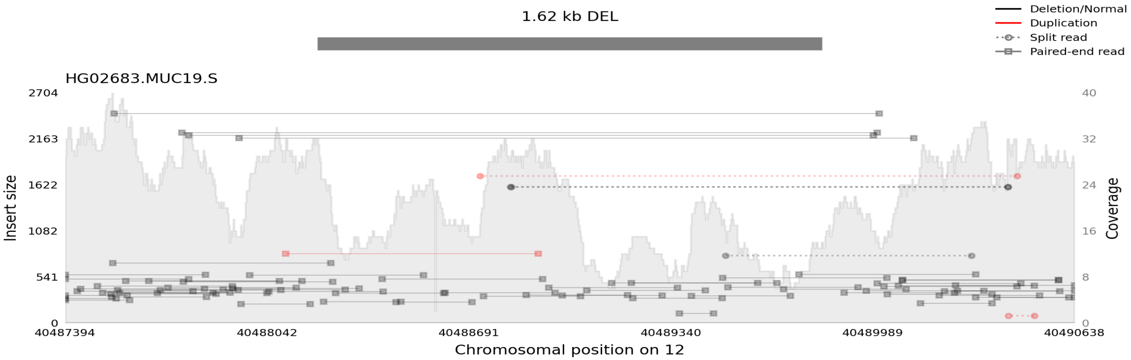

HG02683

Long−reads

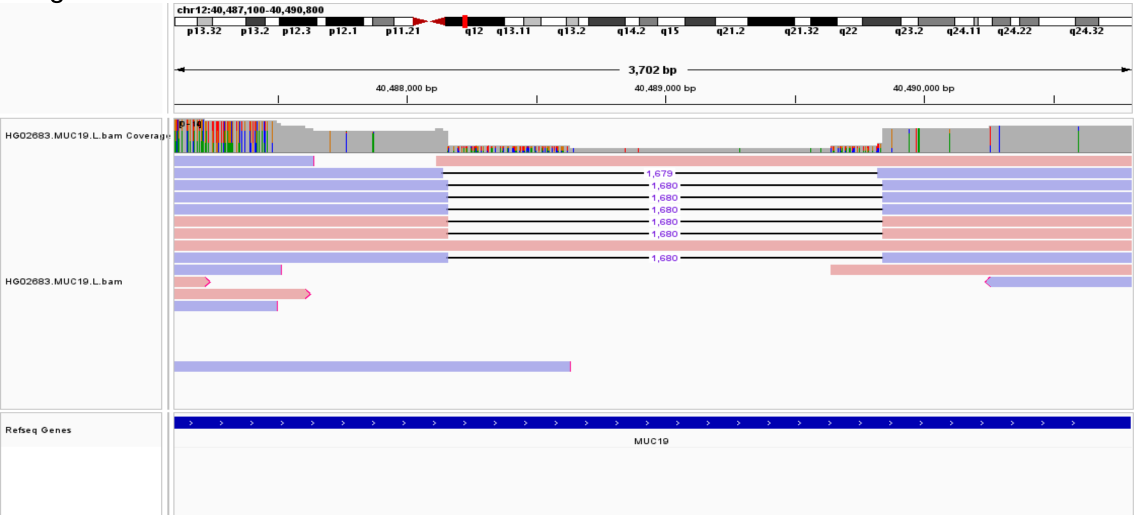

HG02698
Short−reads

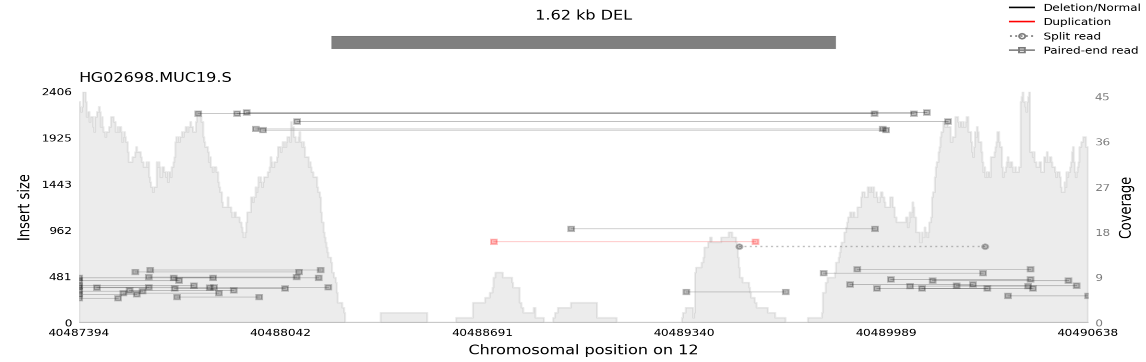

HG02698
Long−reads

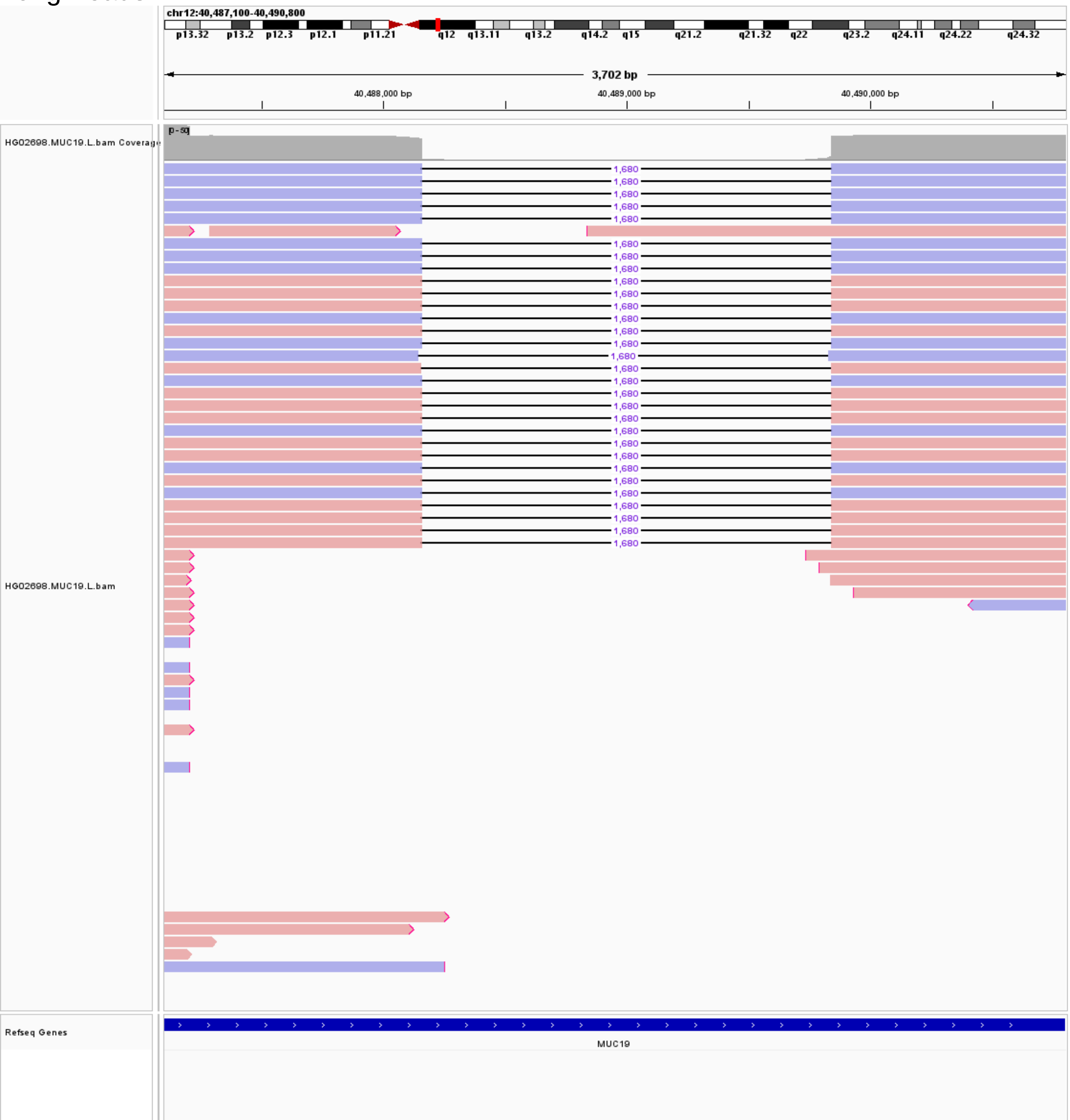

HG02735

Short−reads

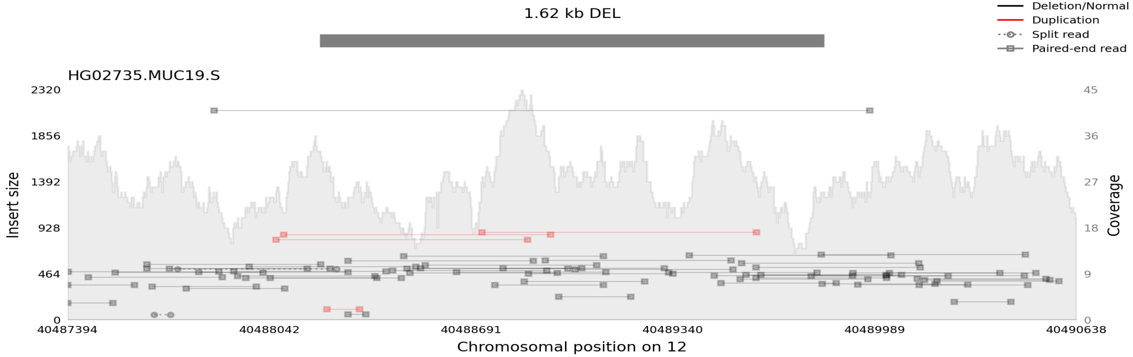

HG02735

Long−reads

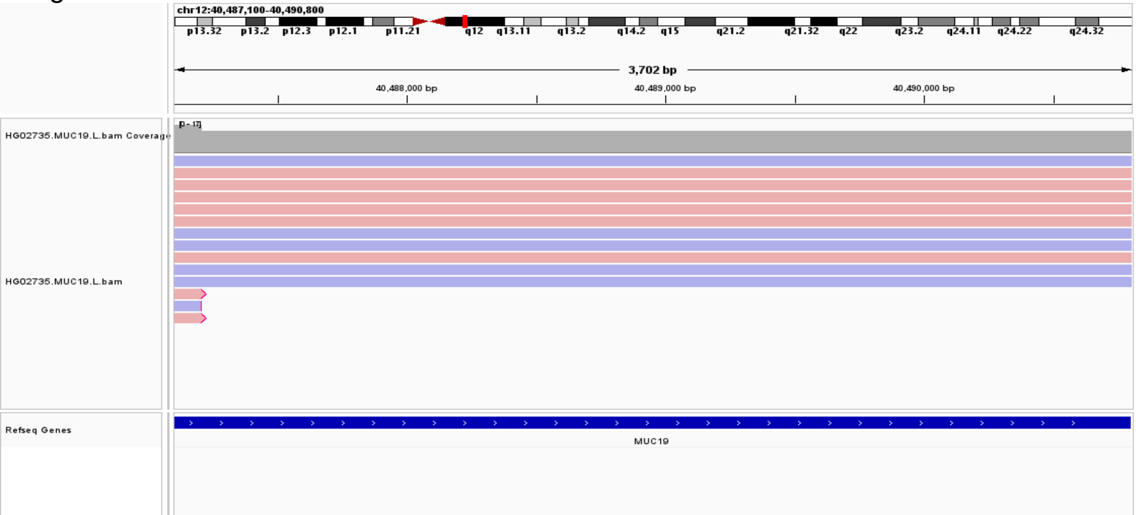

HG02738

Short−reads

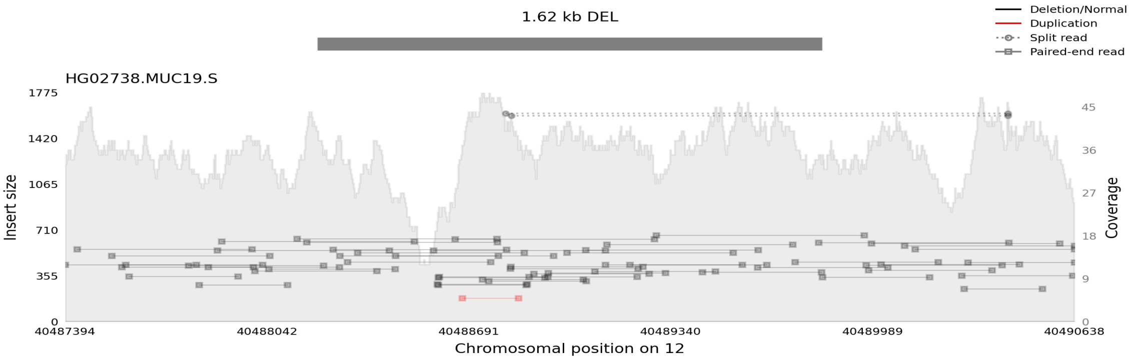

HG02738

Long−reads

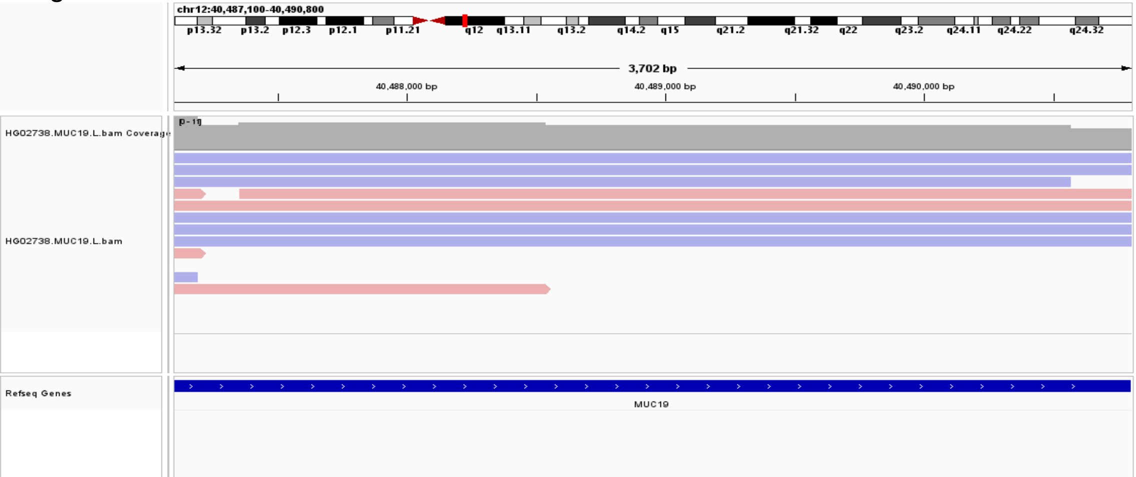

HG02809

Short−reads

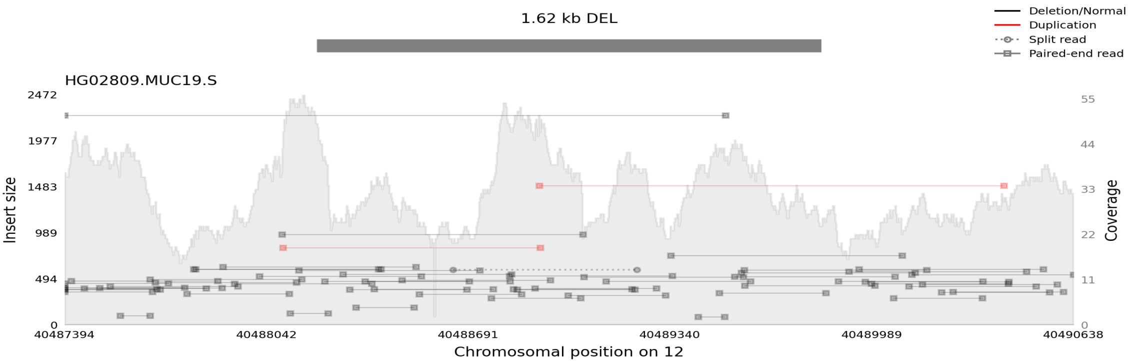

HG02809

Long−reads

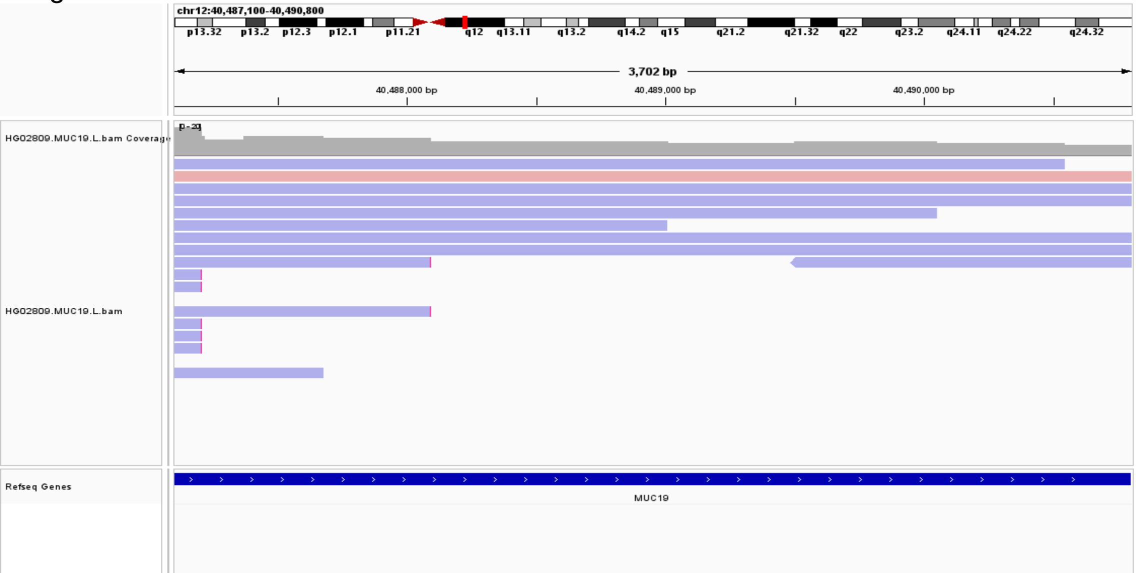

HG02818

Short−reads

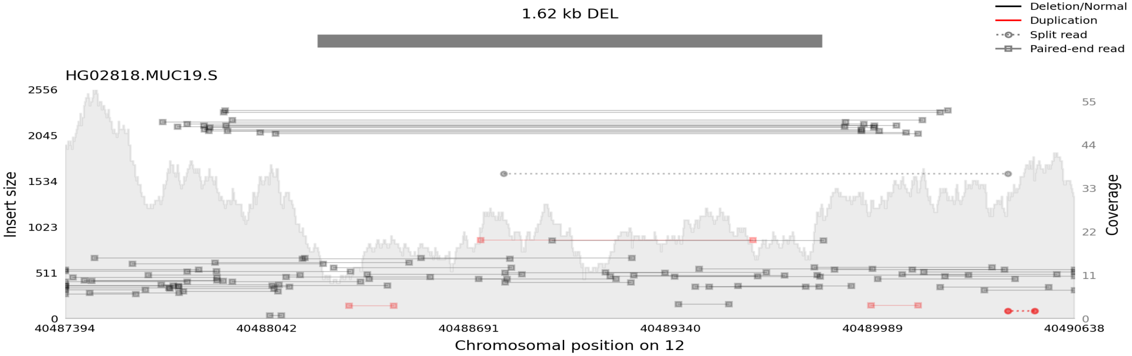

HG02818

Long−reads

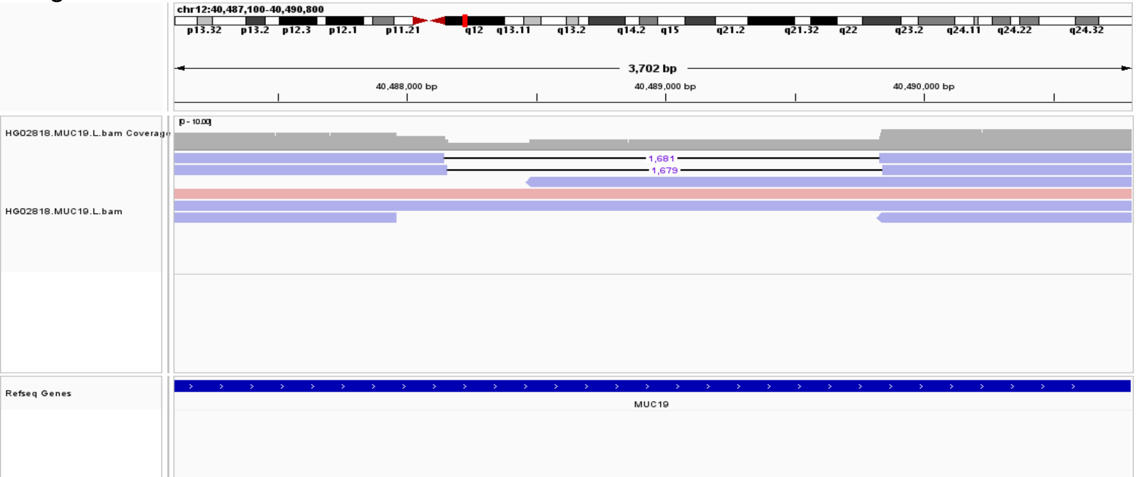

HG02922

Short−reads

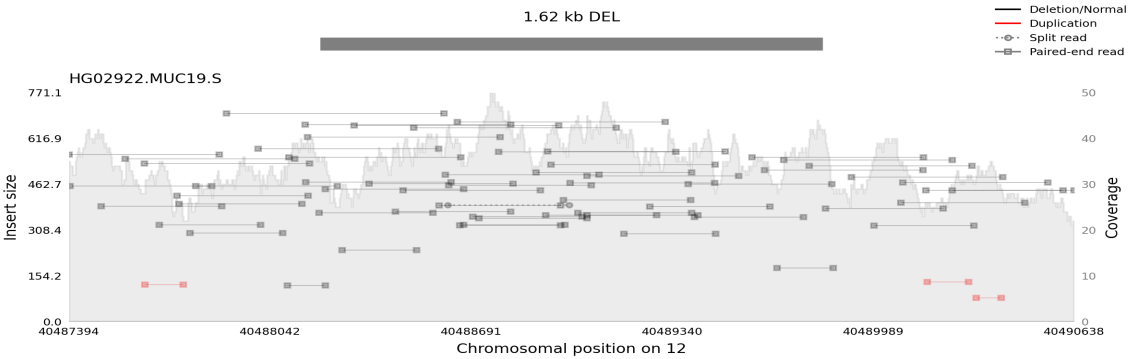

HG02922

Long−reads

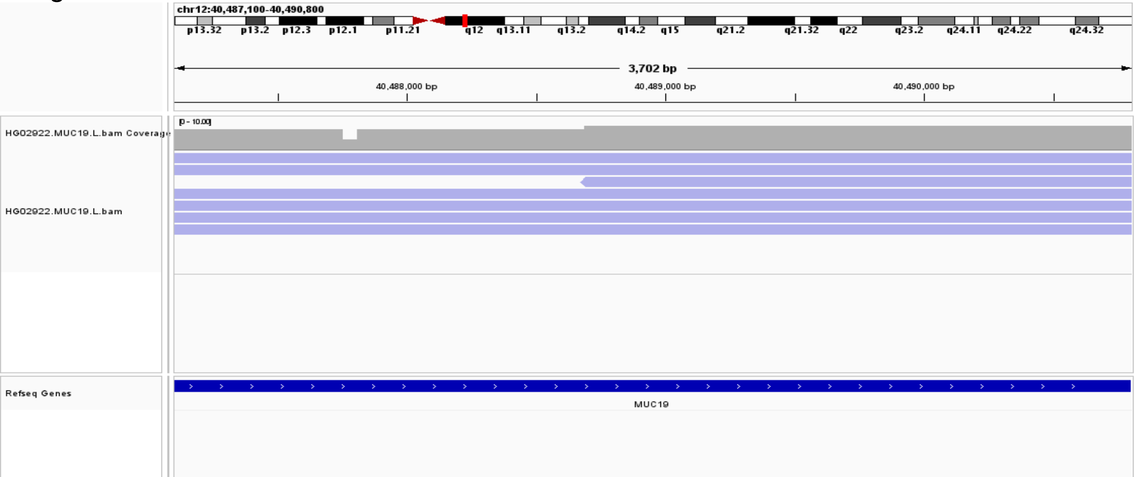

HG02965

Short−reads

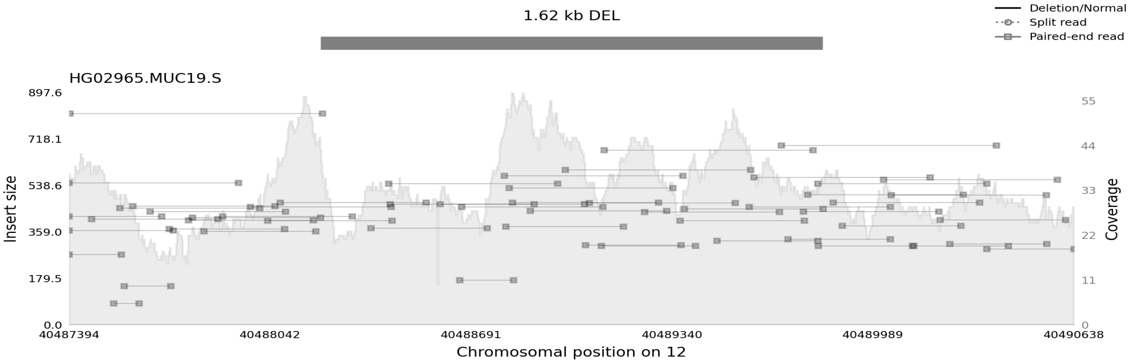

HG02965

Long−reads

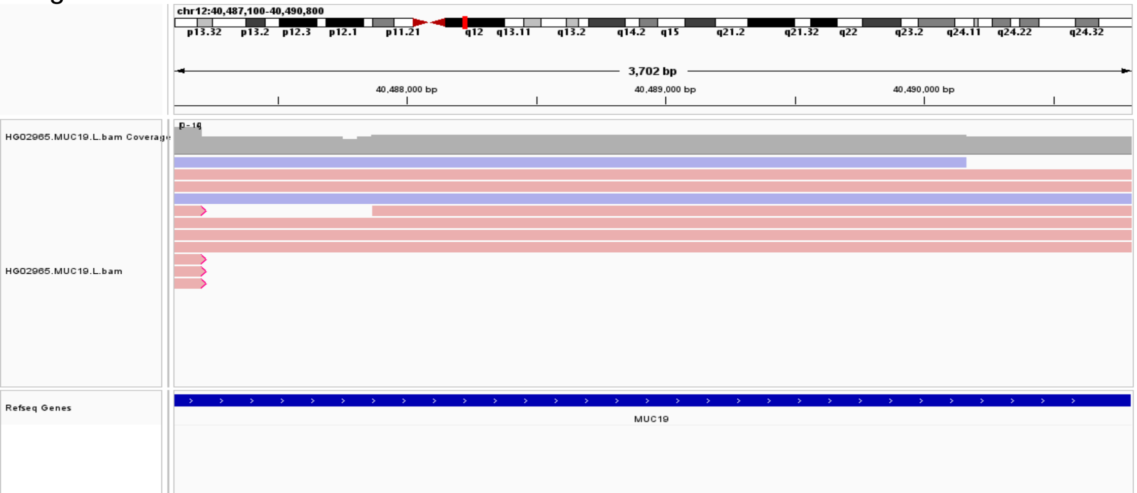

HG02976

Short−reads

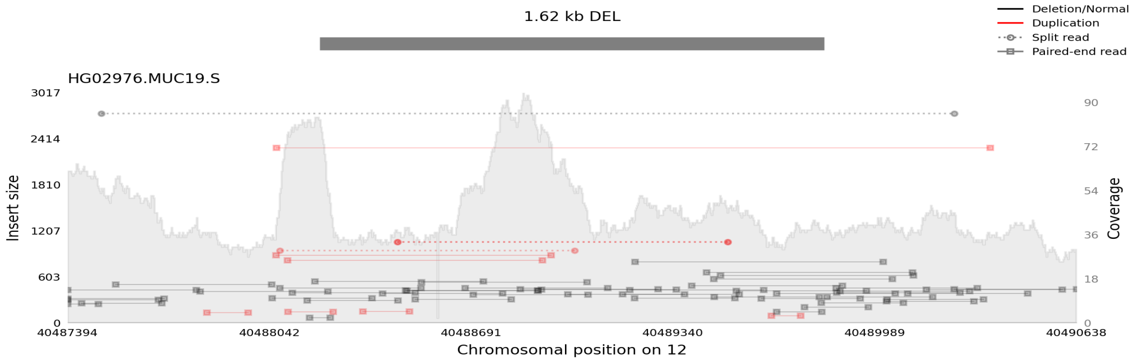

HG02976

Long−reads

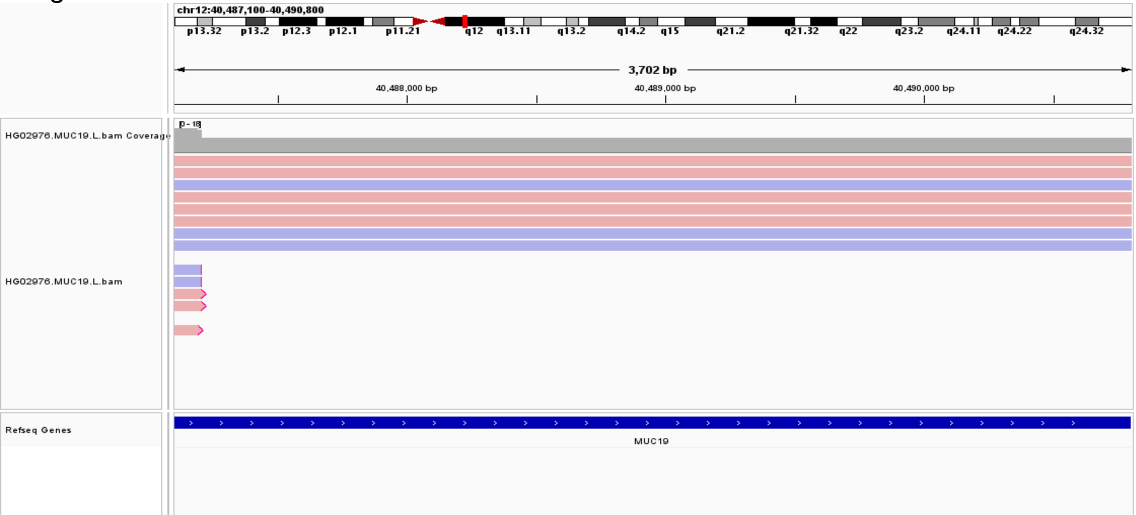

HG03017

Short−reads

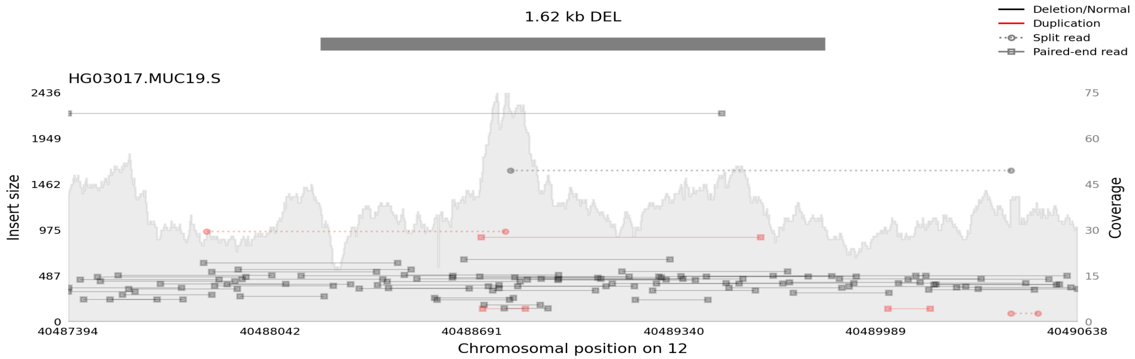

HG03017

Long−reads

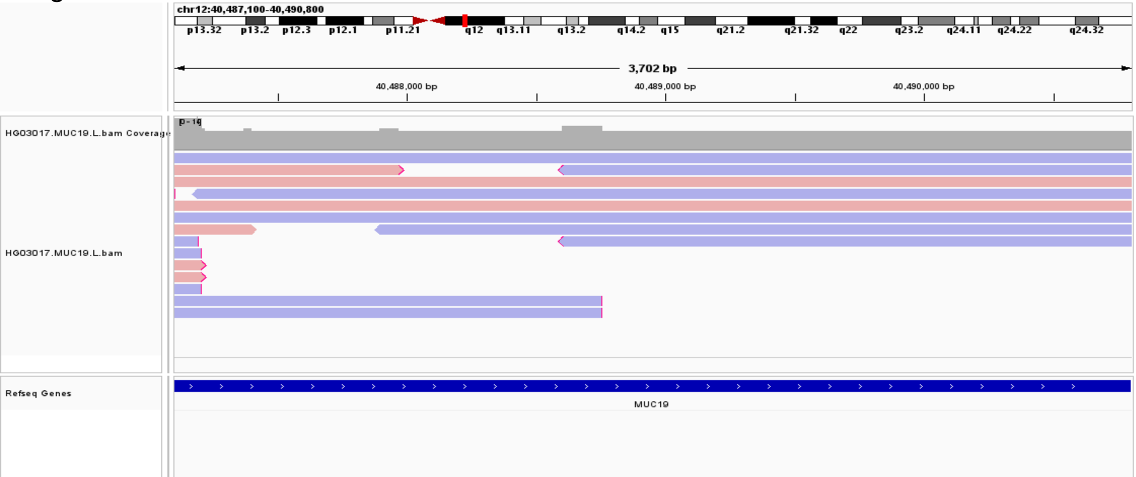

HG03041

Short−reads

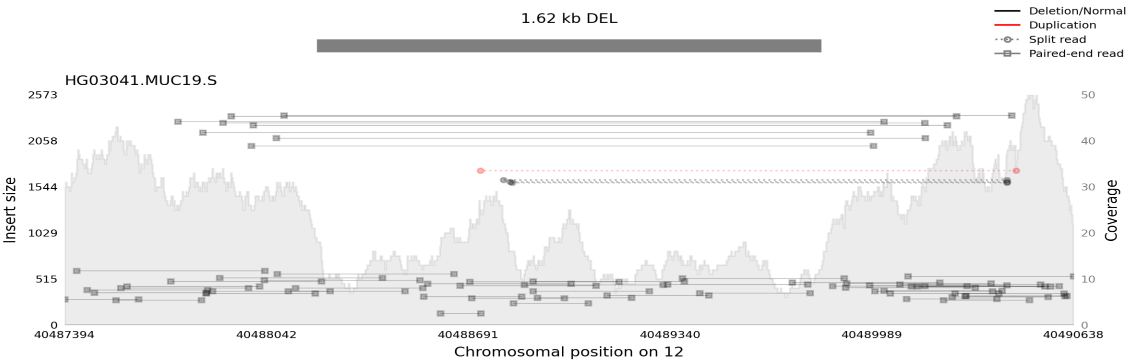

HG03041

Long−reads

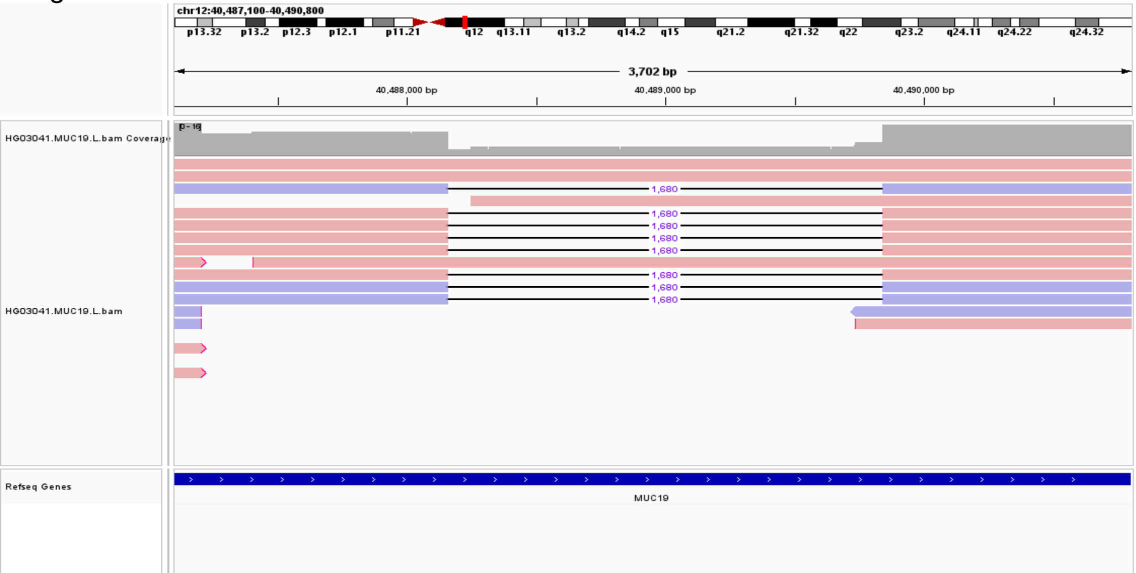

HG03130

Short−reads

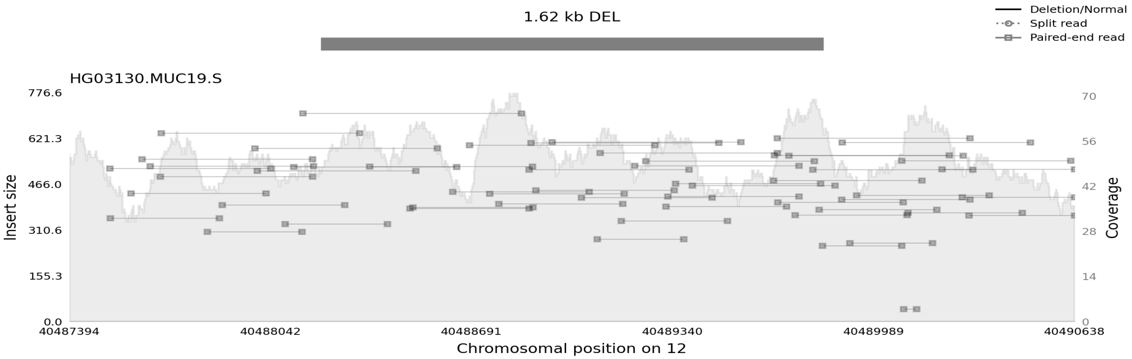

HG03130

Long−reads

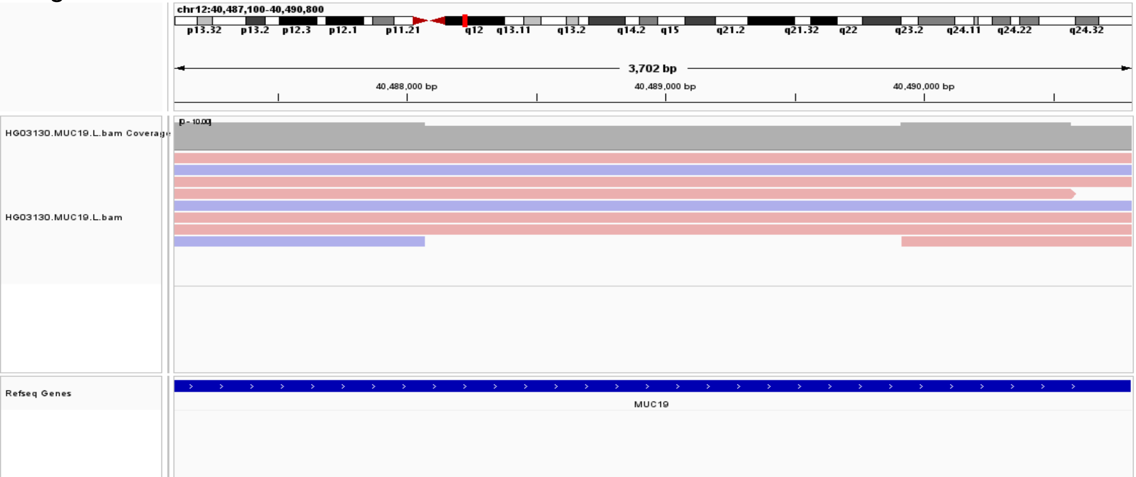

HG03139

Short−reads

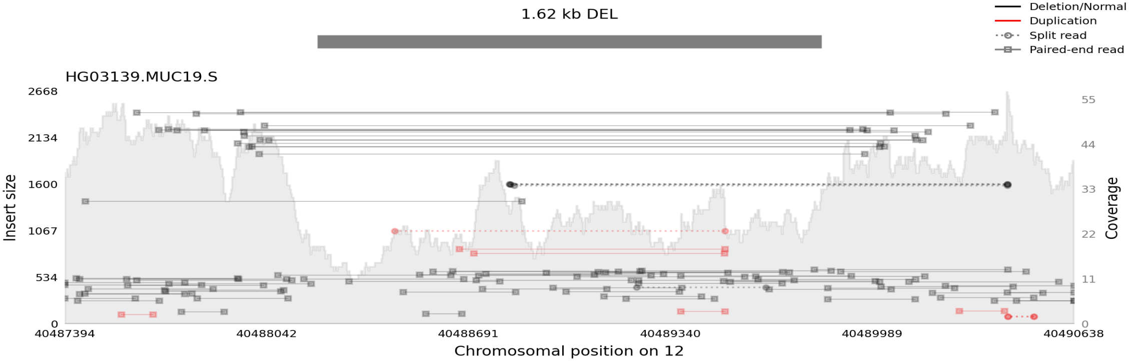

HG03139

Long−reads

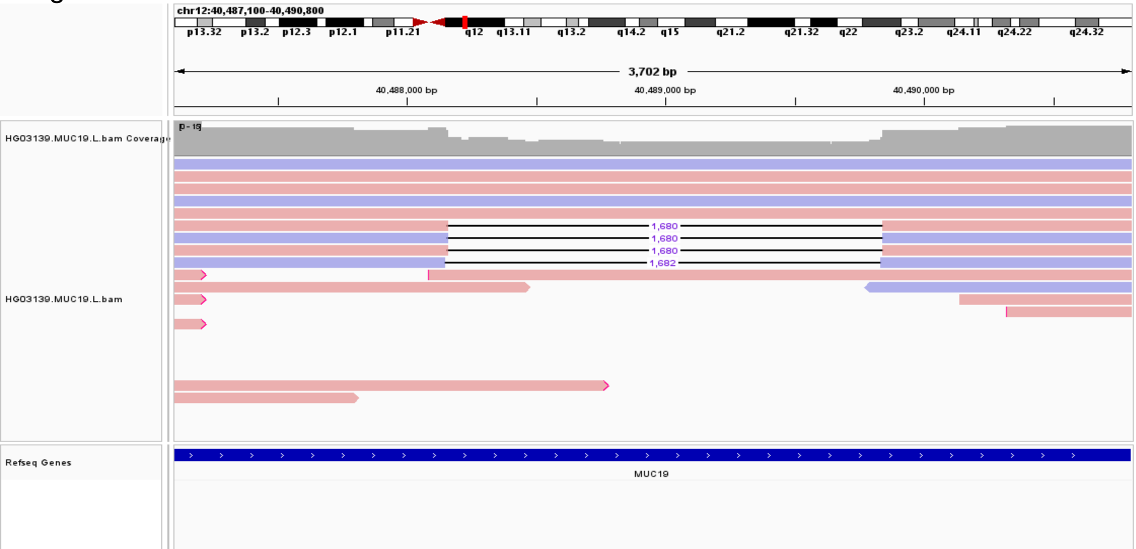

HG03195

Short−reads

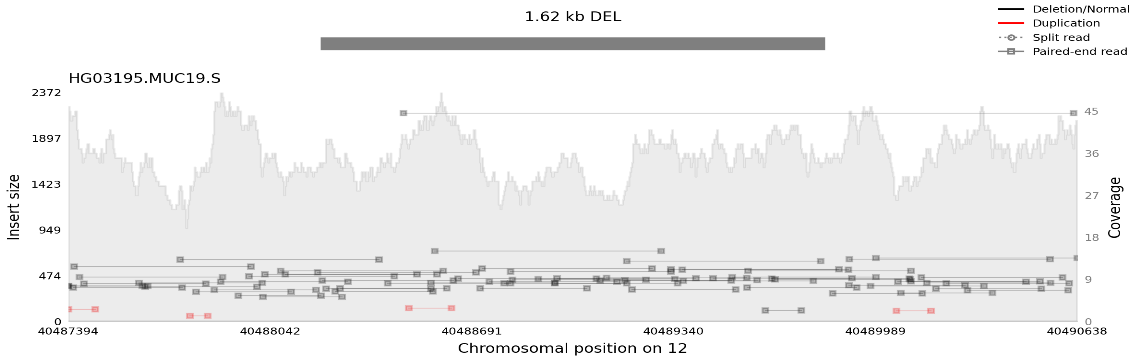

HG03195

Long−reads

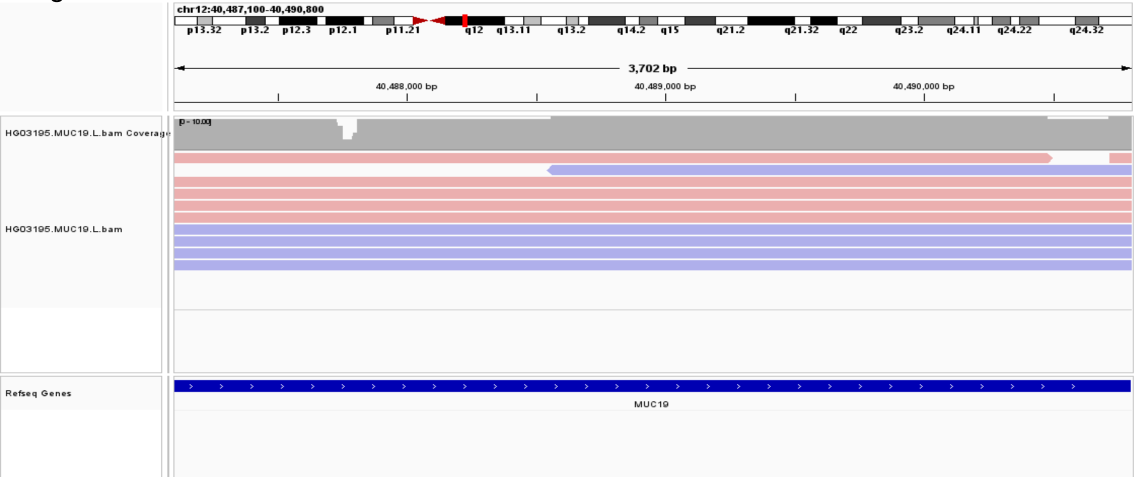

HG03209

Short−reads

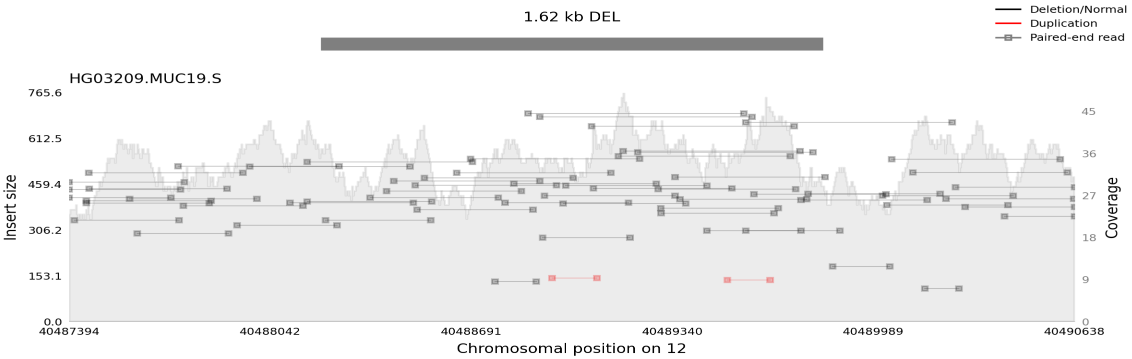

HG03209

Long−reads

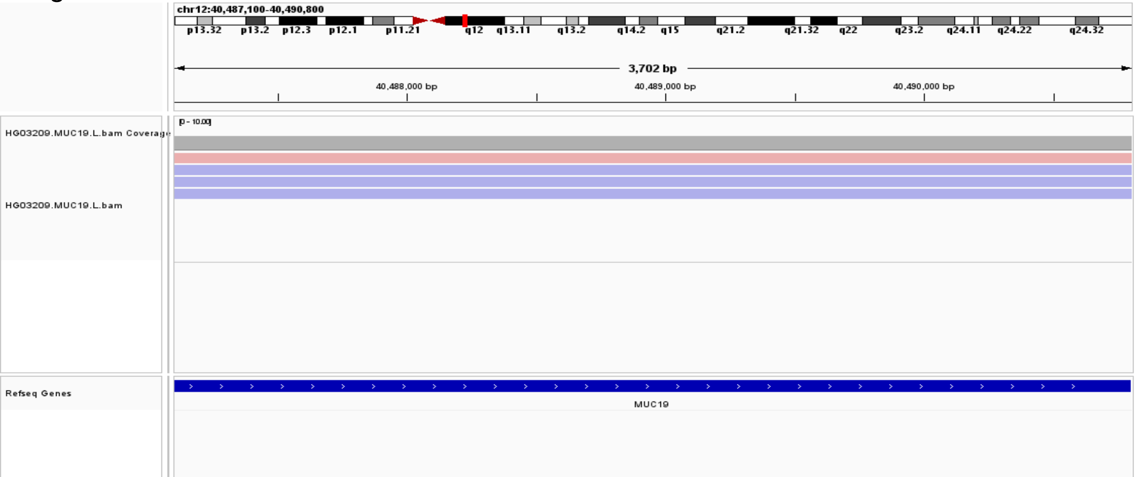

HG03225

Short−reads

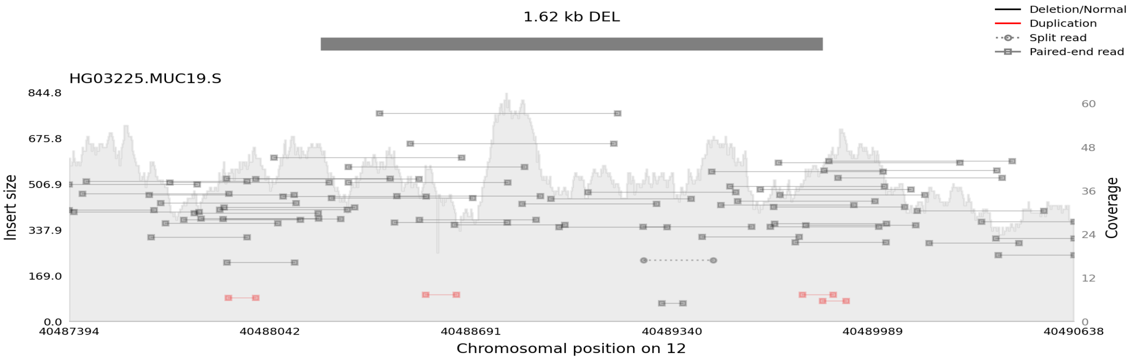

HG03225

Long−reads

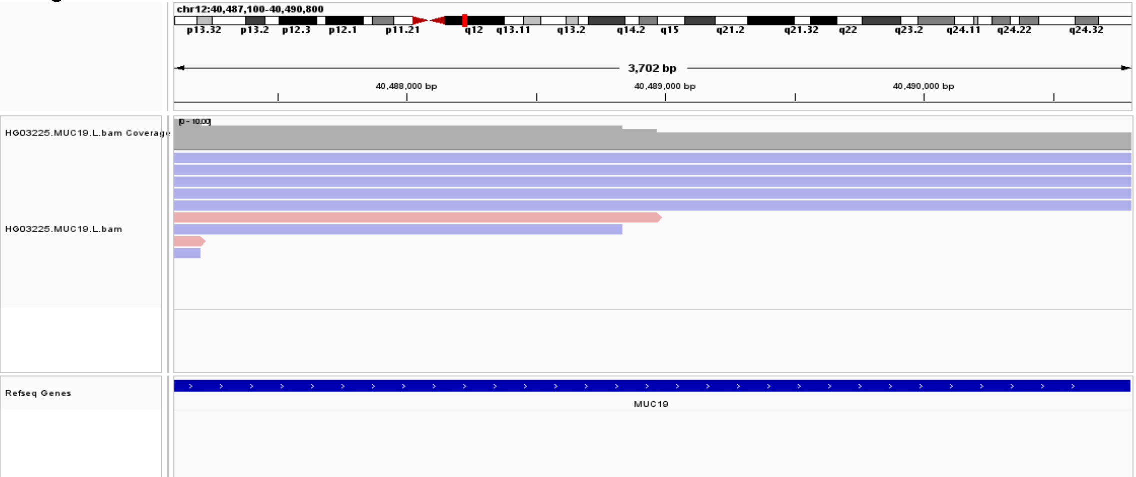

HG03239

Short−reads

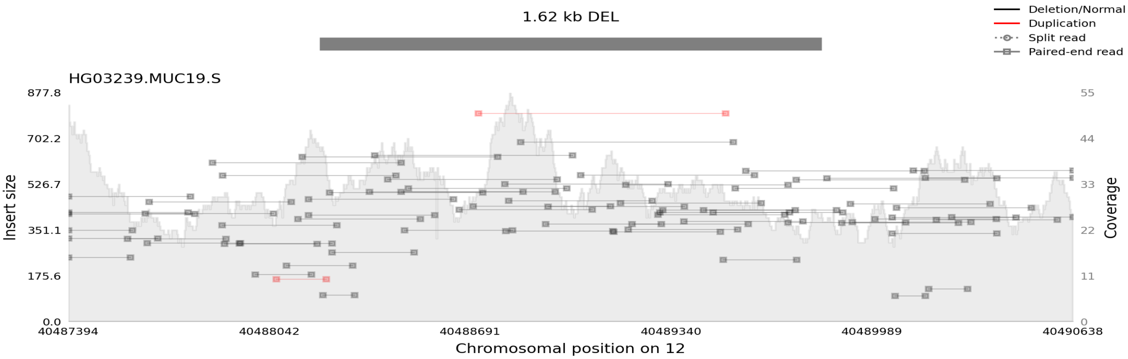

HG03239

Long−reads

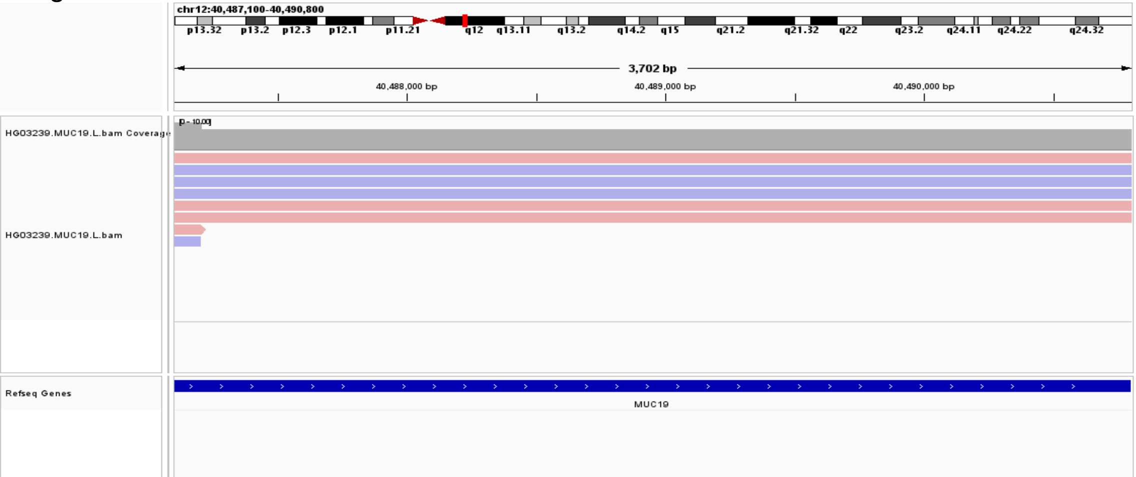

HG03453

Short−reads

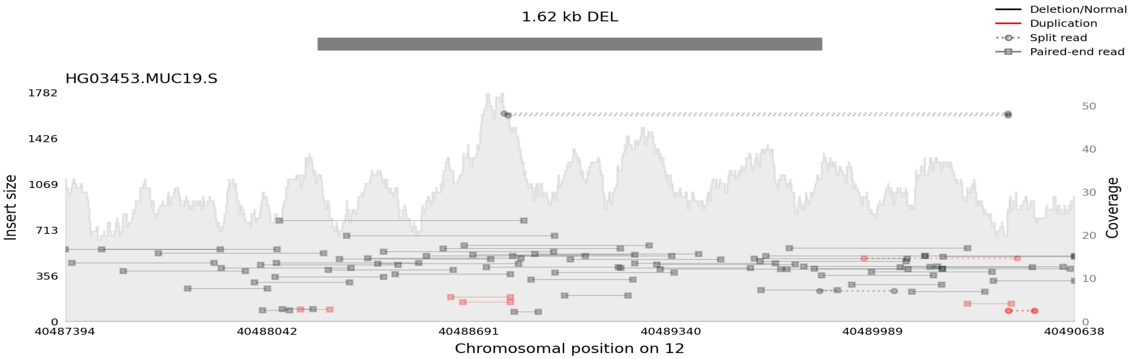

HG03453

Long−reads

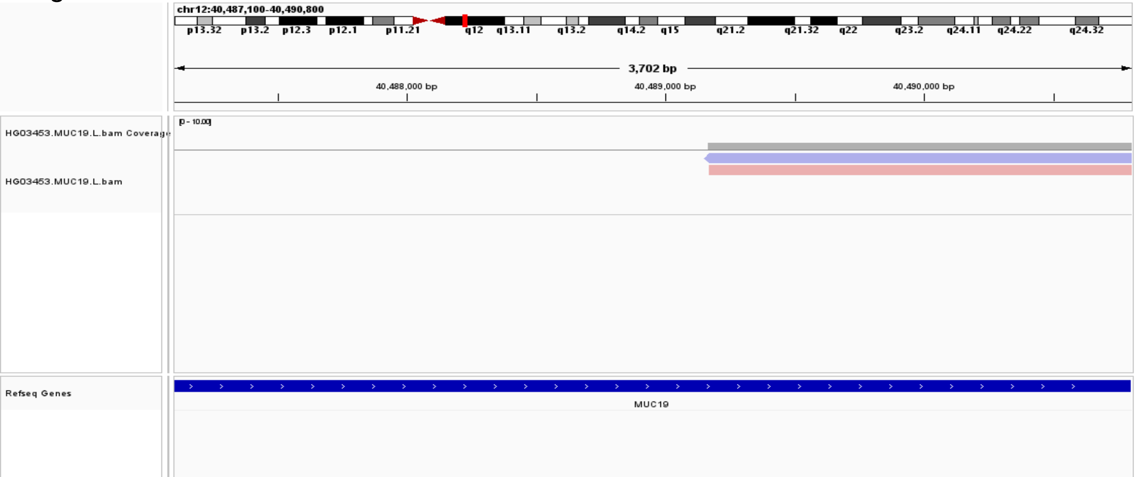

HG03516

Short−reads

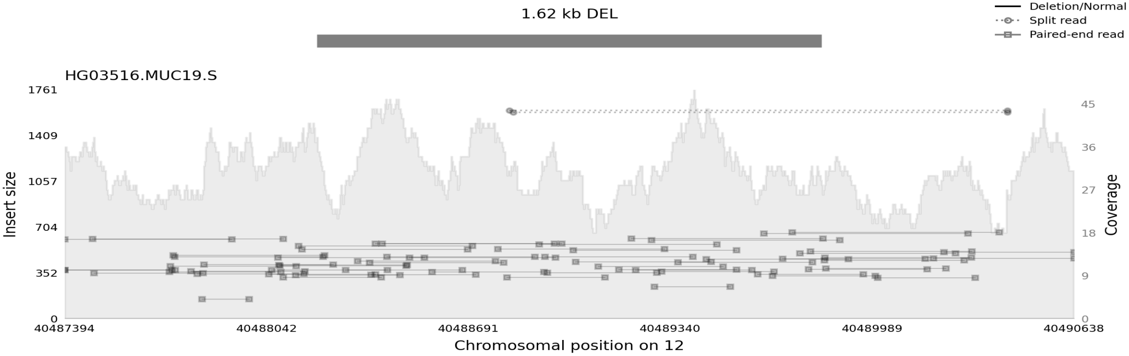

HG03516

Long−reads

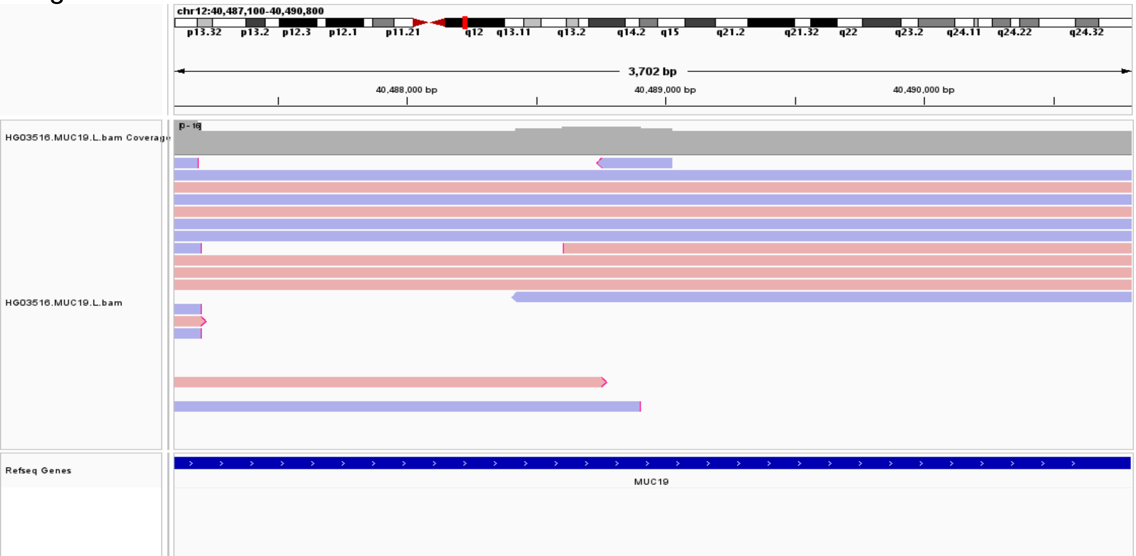

HG03654

Short−reads

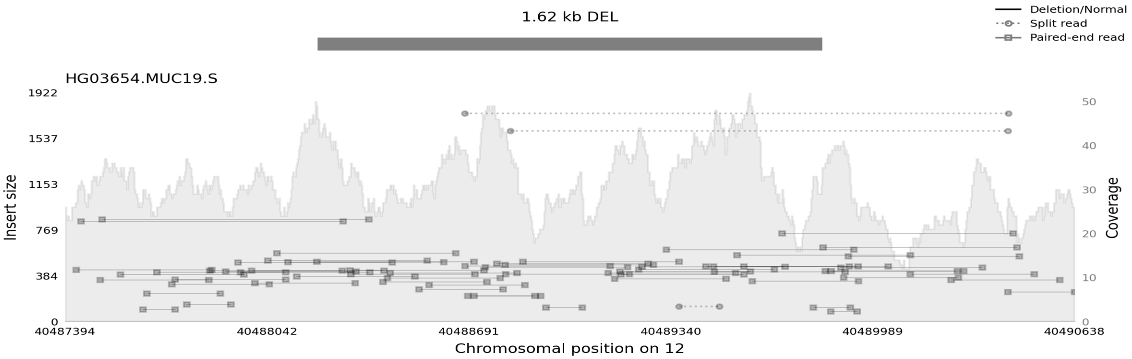

HG03654

Long−reads

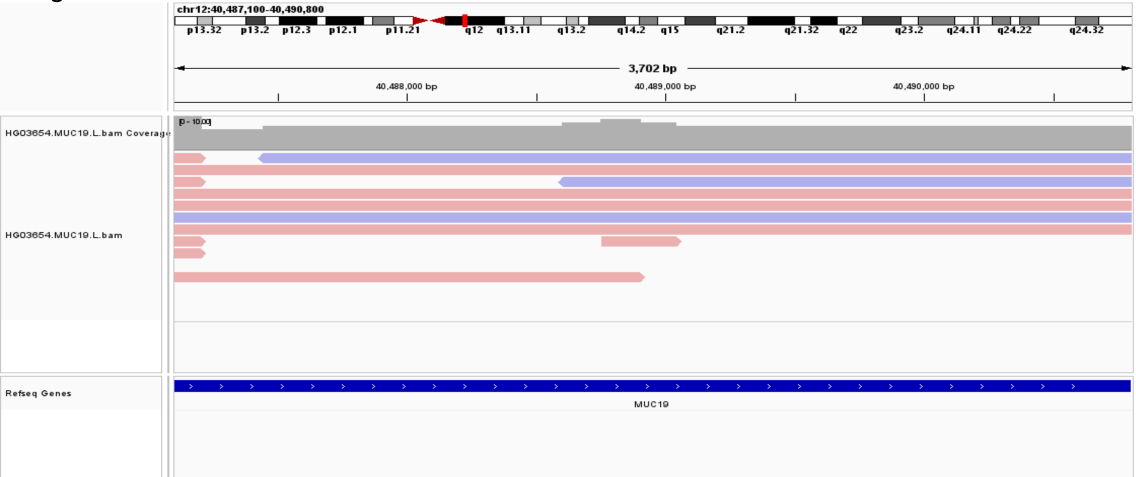

HG03669

Short−reads

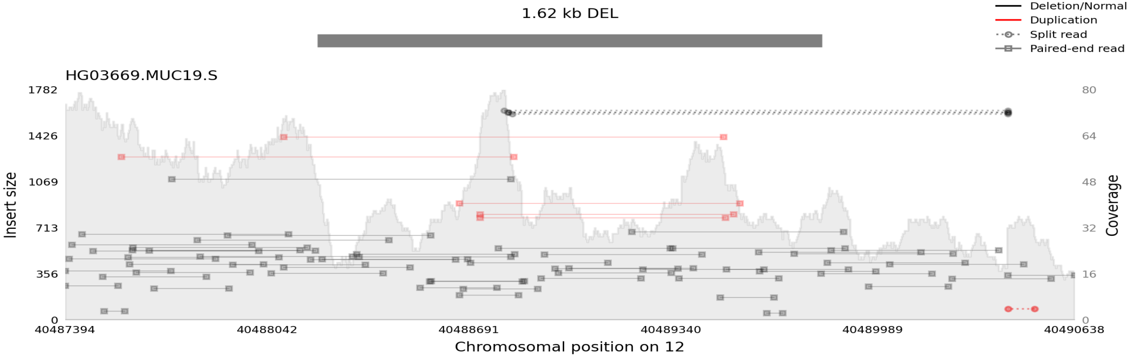

HG03669

Long−reads

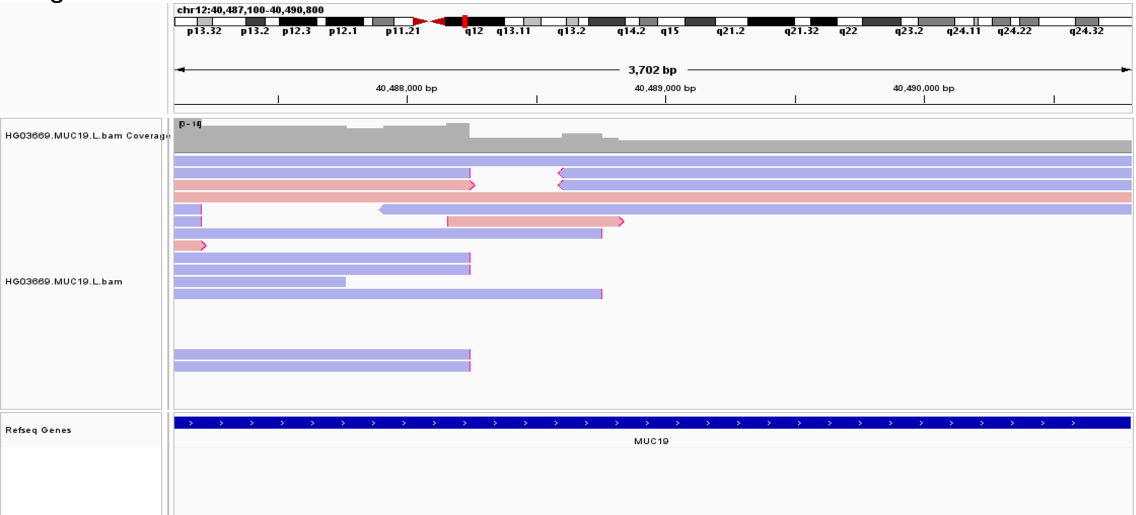

HG03683

Short−reads

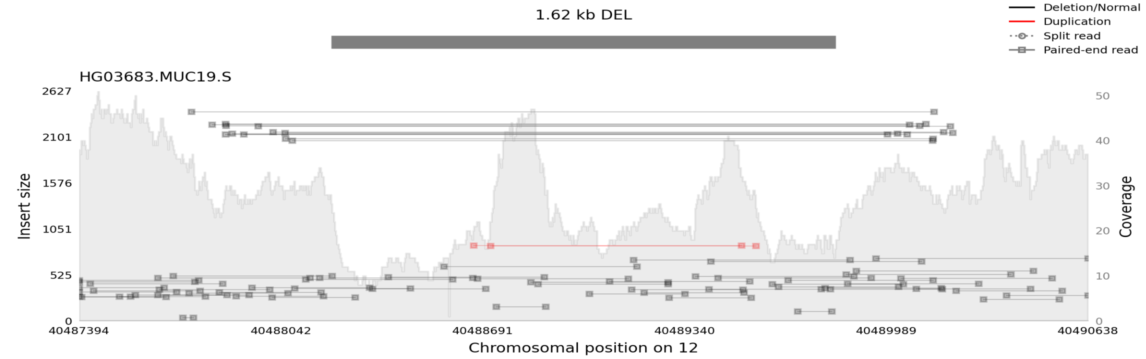

HG03683

Long−reads

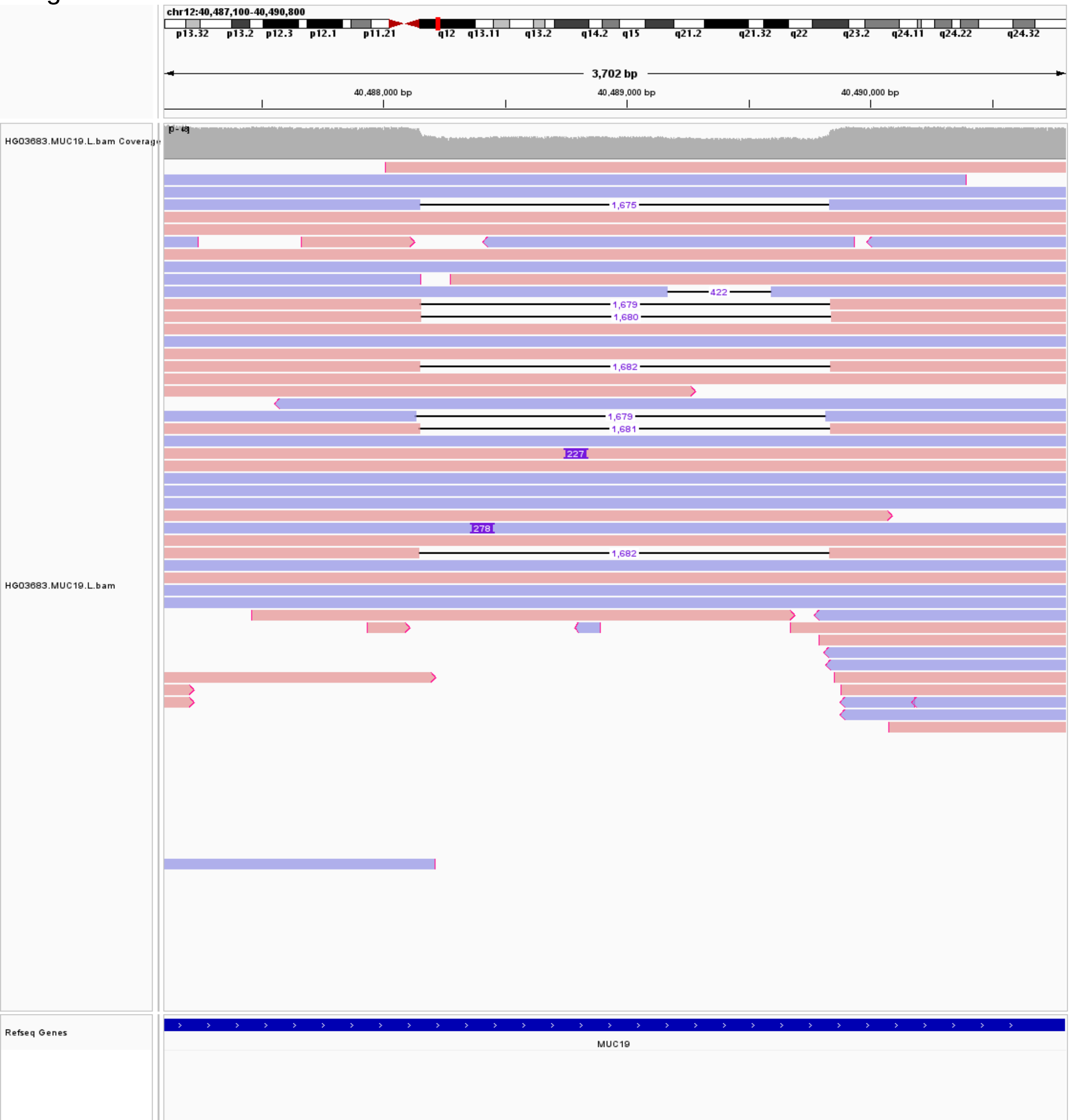

HG03688

Short−reads

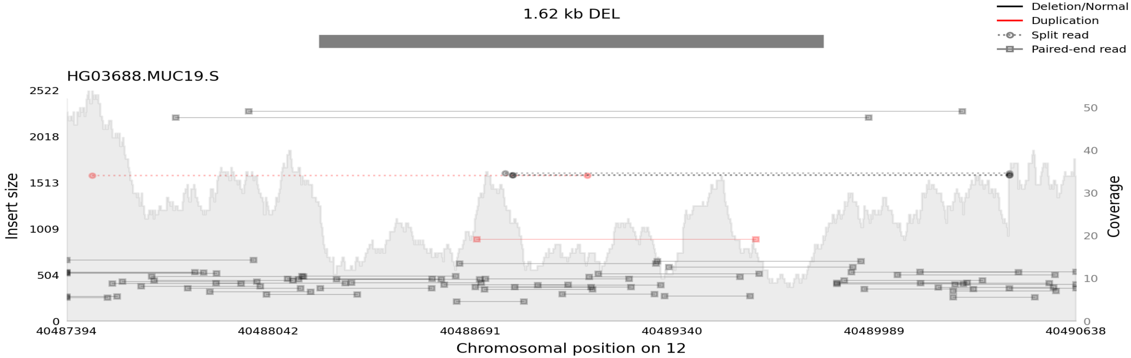

HG03688

Long−reads

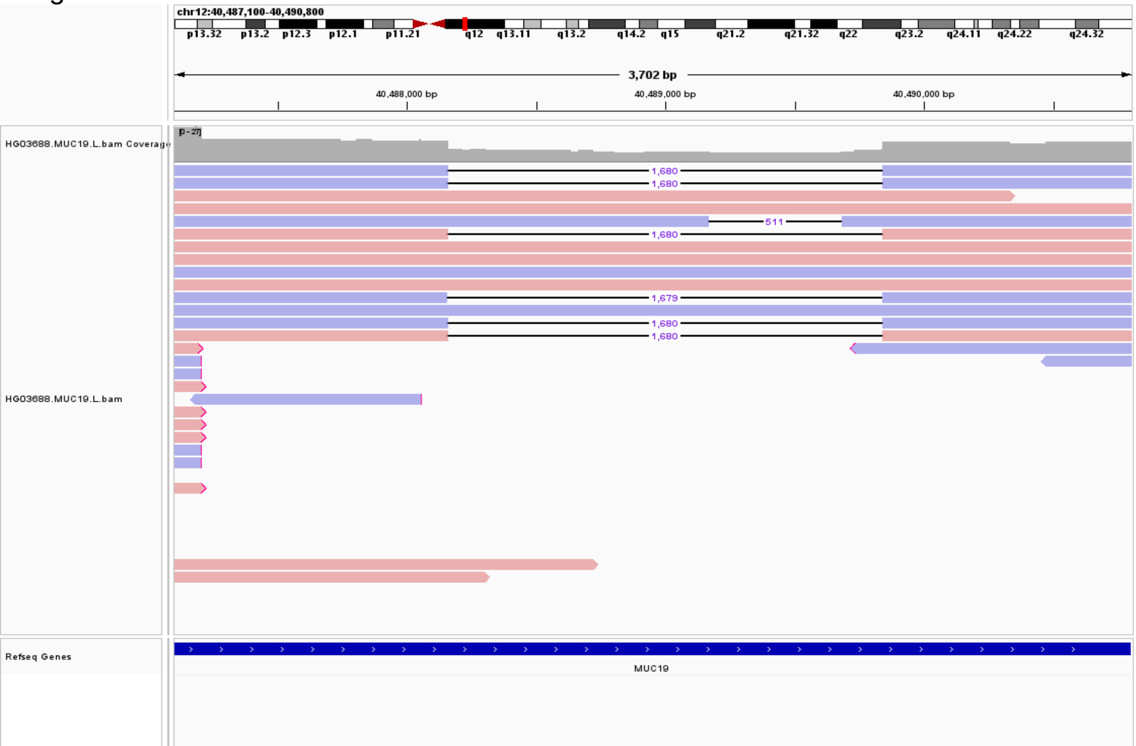

HG03704

Short−reads

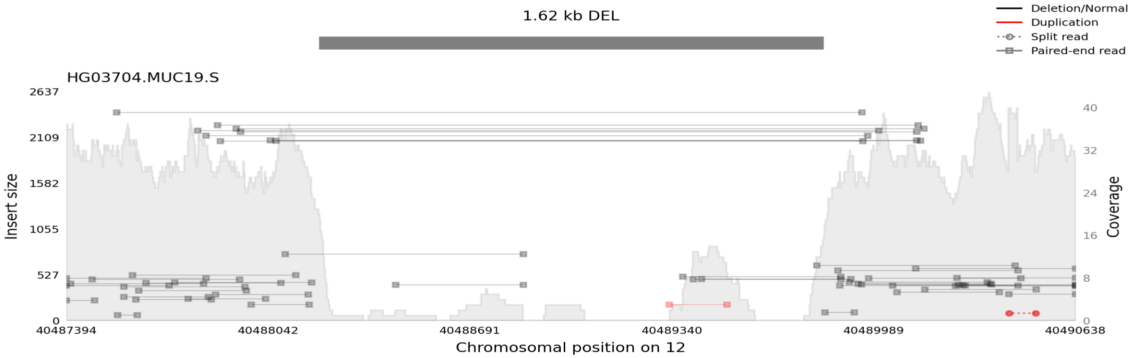

HG03704

Long−reads

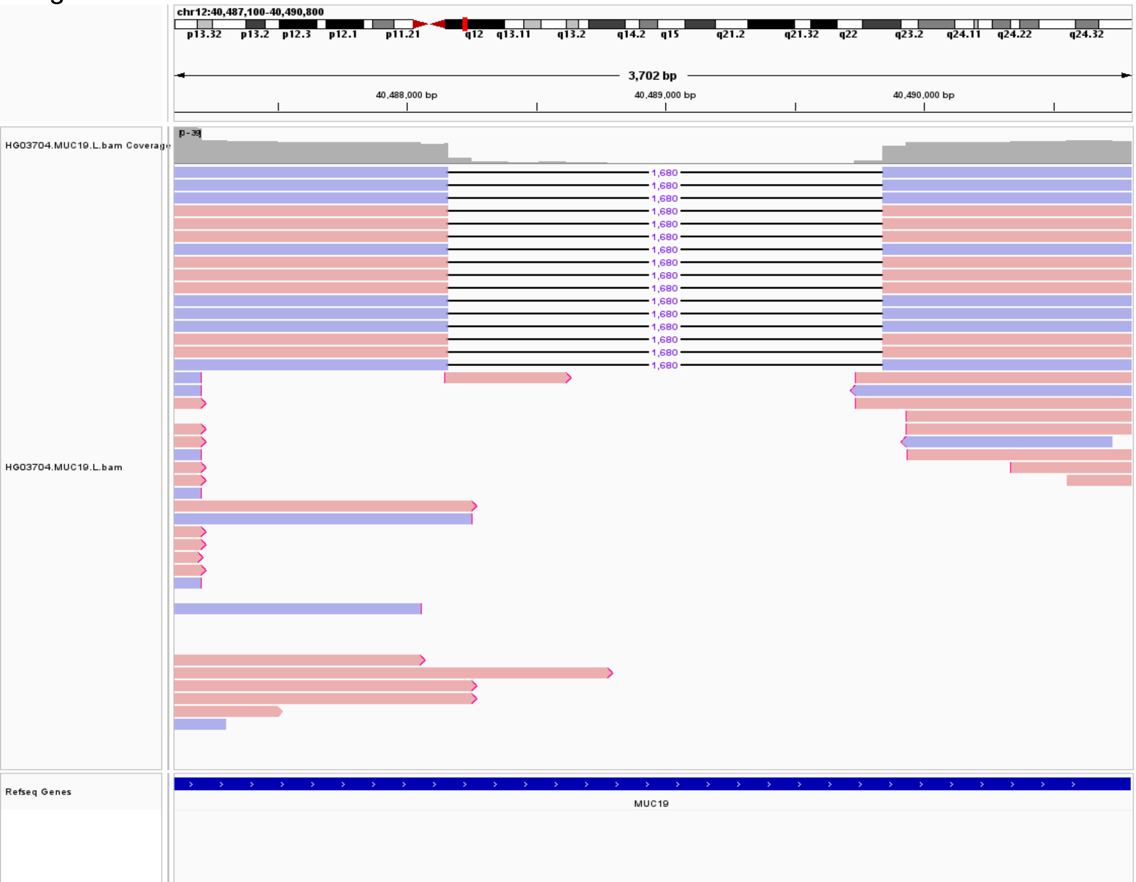

HG03710

Short−reads

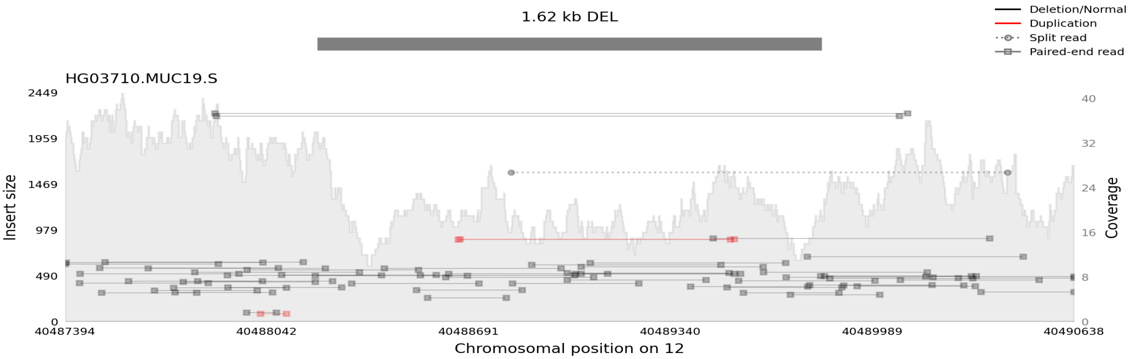

HG03710

Long−reads

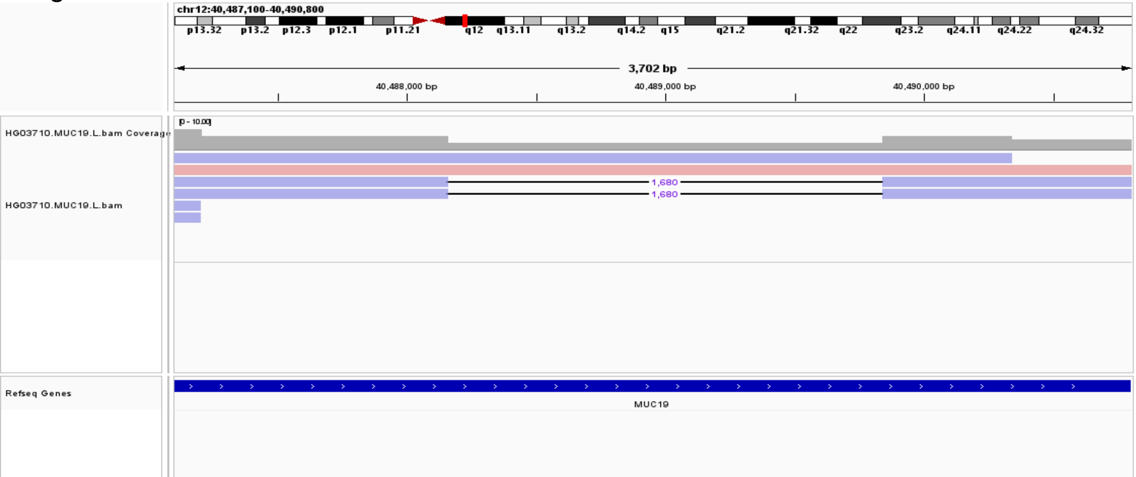

HG03732

Short−reads

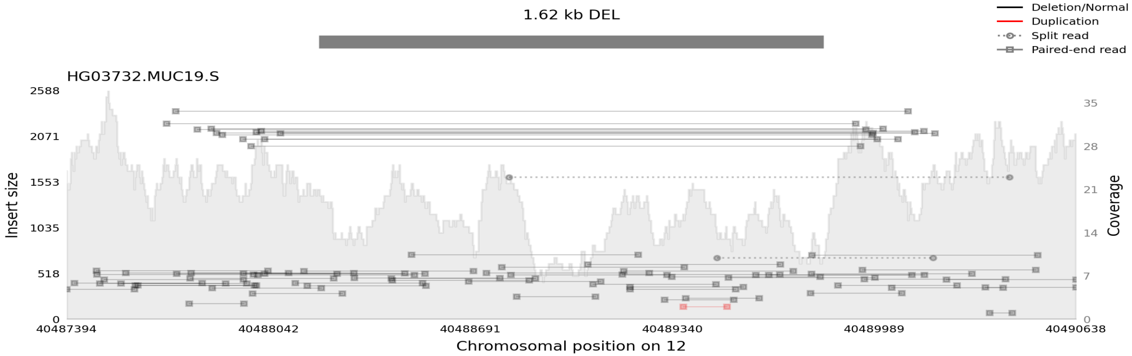

HG03732

Long−reads

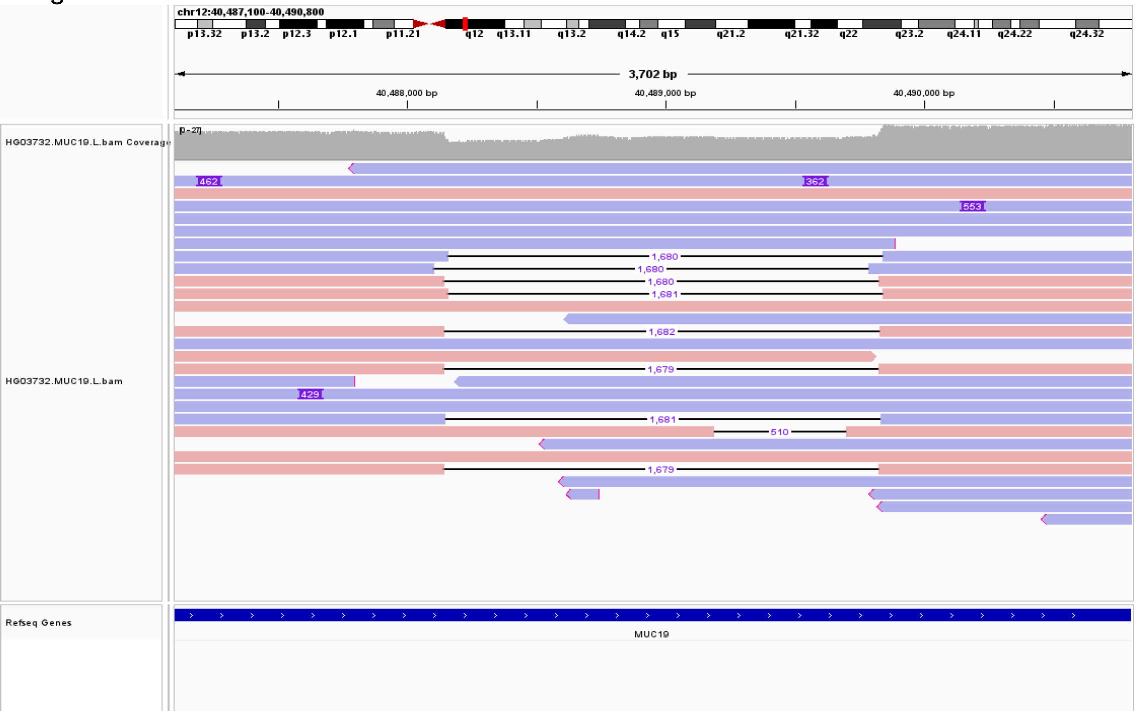

HG03831

Short−reads

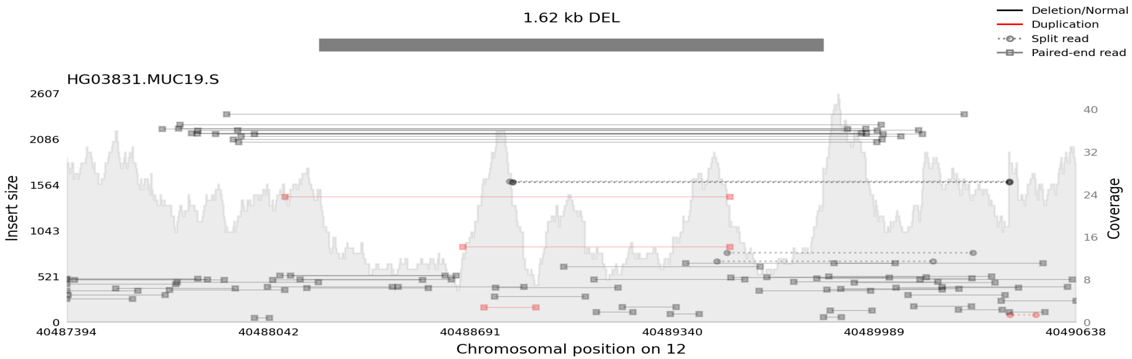

HG03831

Long−reads

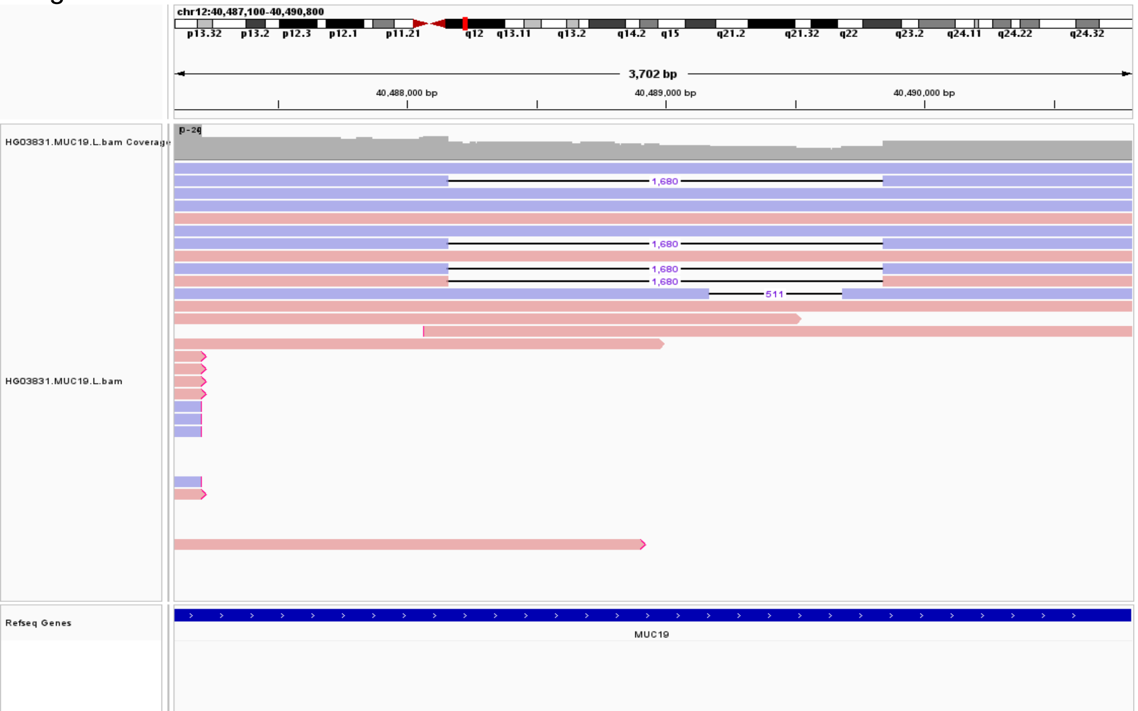

HG03942

Short−reads

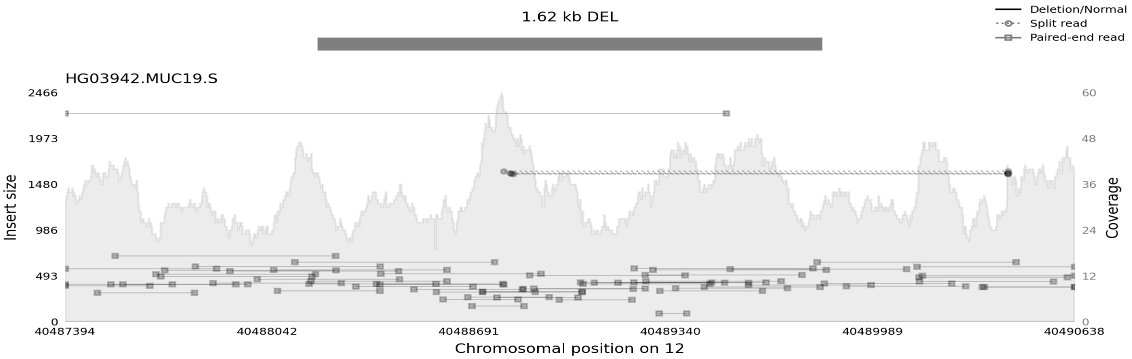

HG03942

Long−reads

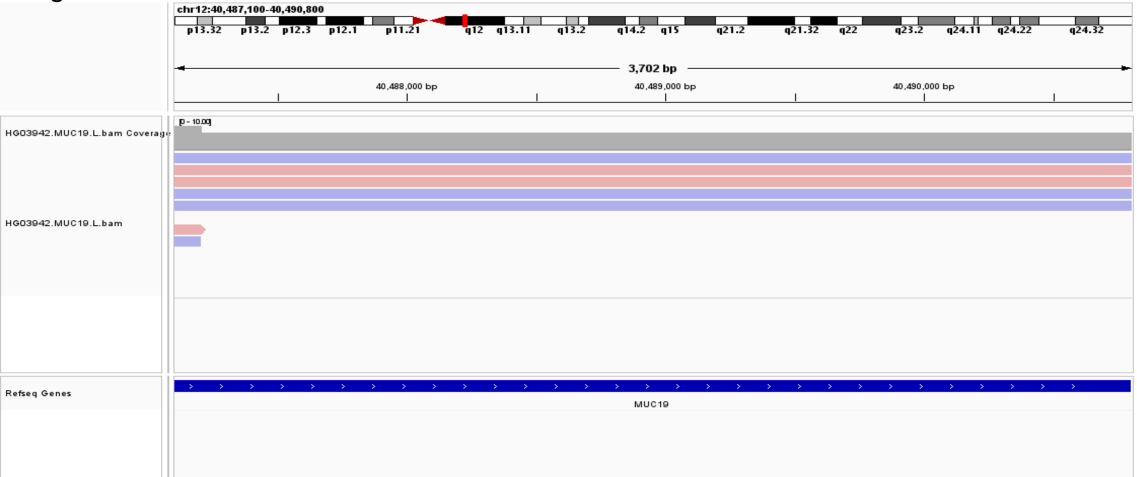

HG04115

Short−reads

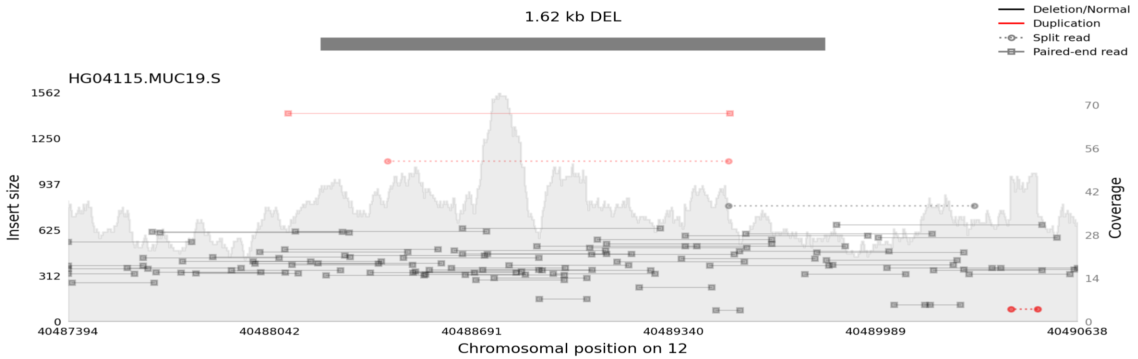

HG04115

Long−reads

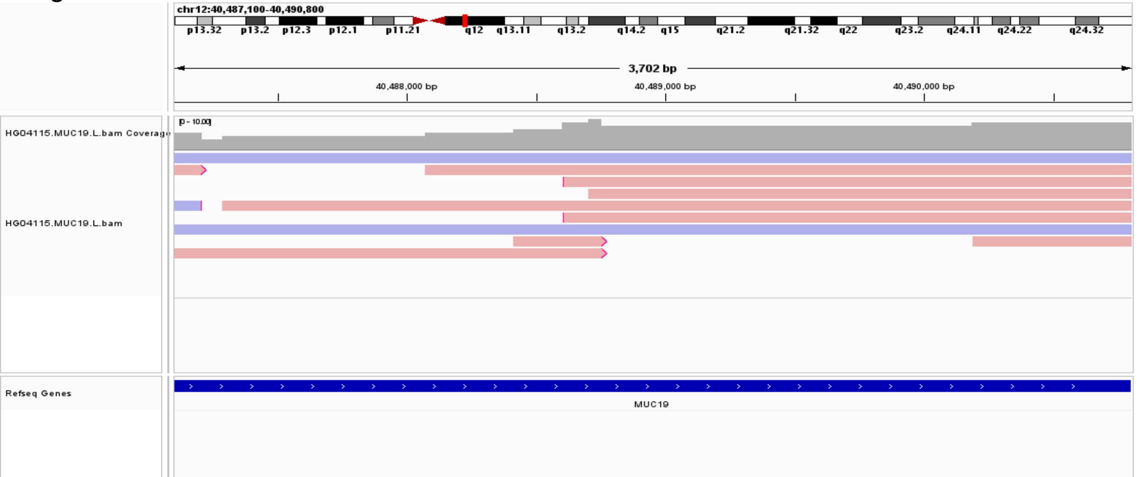

HG04157

Short−reads

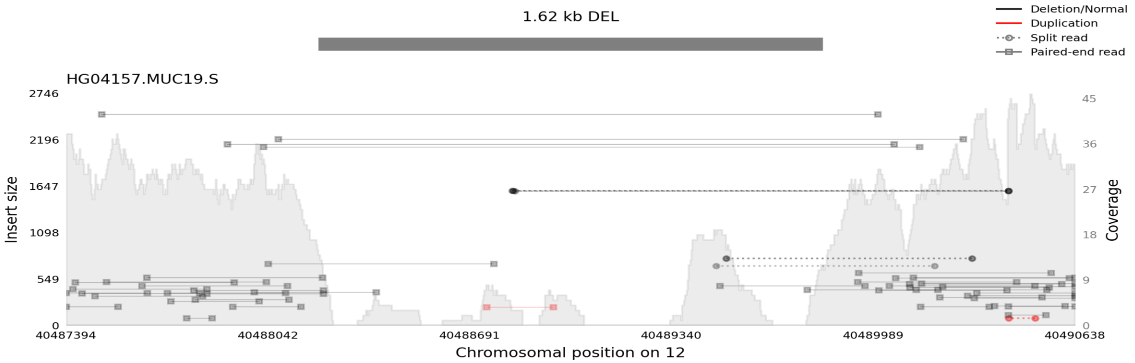

HG04157

Long−reads

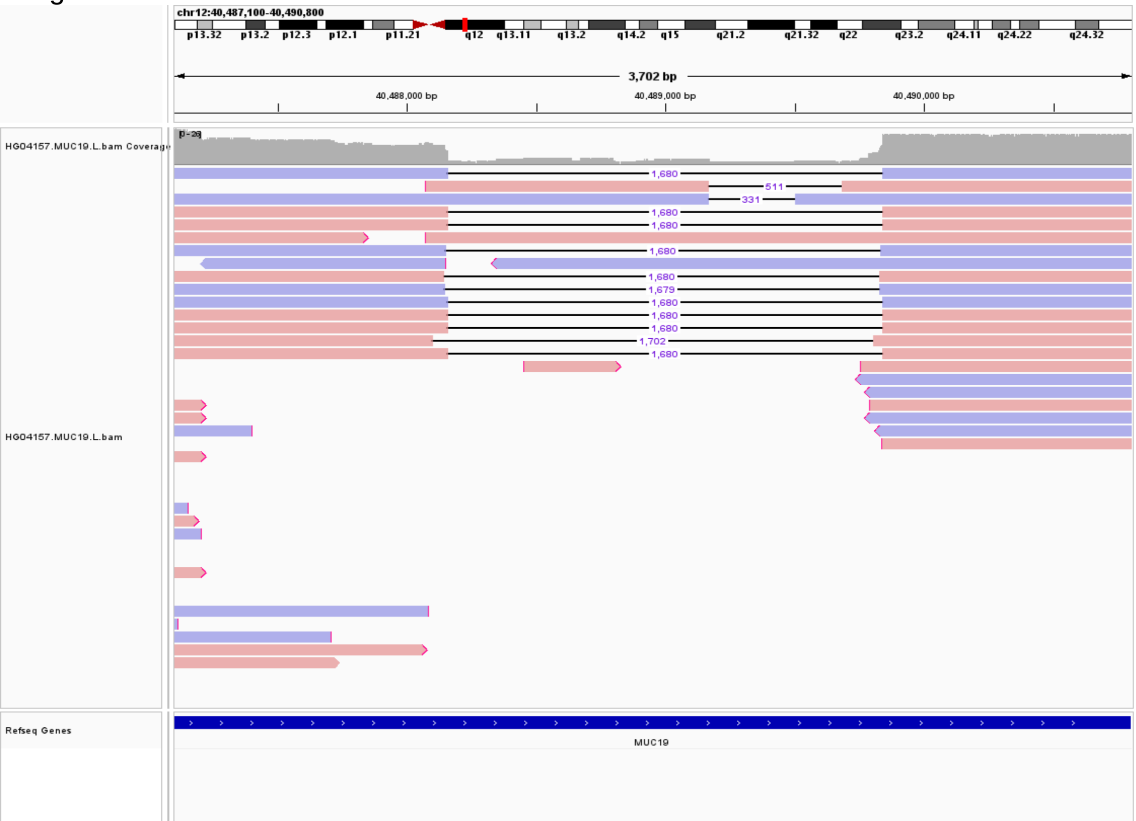

HG04184

Short−reads

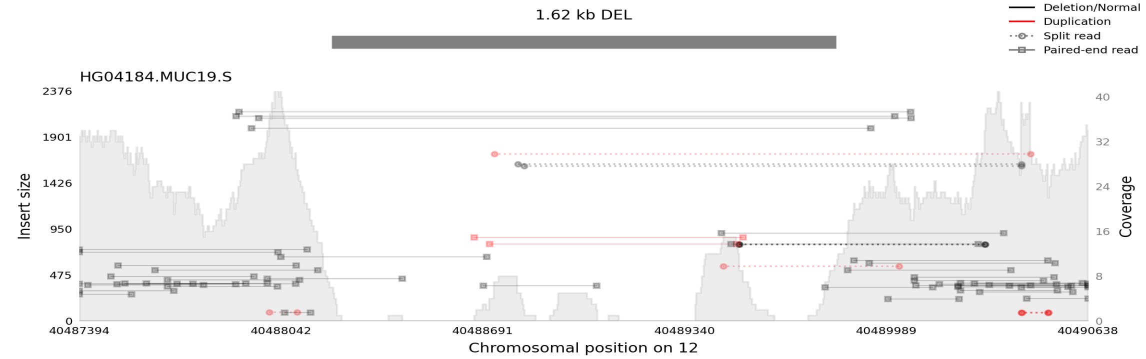

HG04184

Long−reads

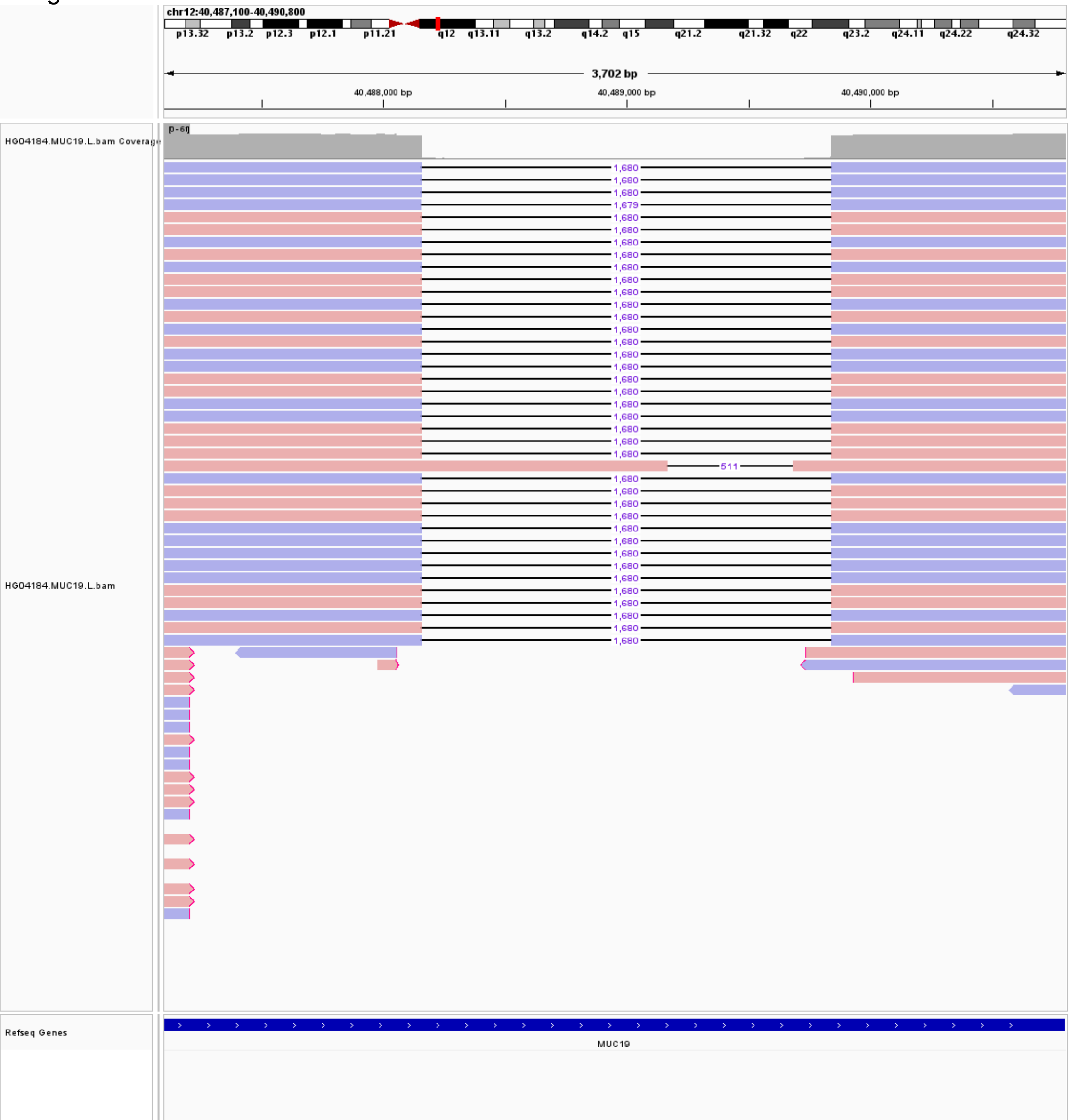

HG04187

Short−reads

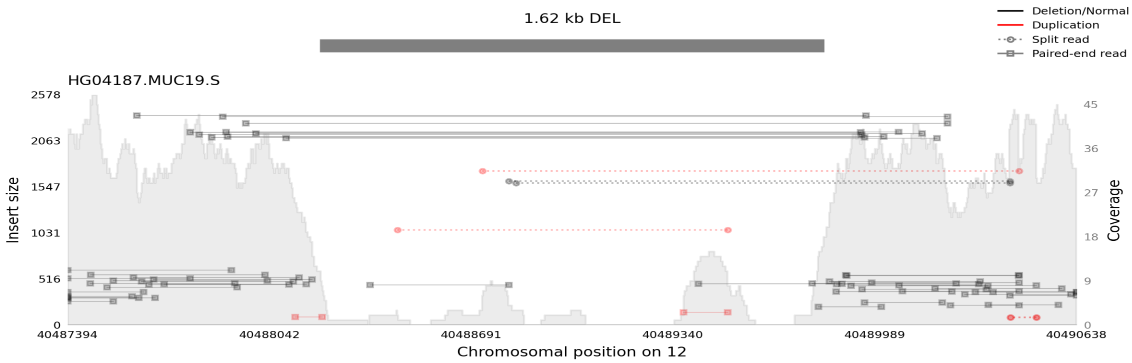

HG04187

Long−reads

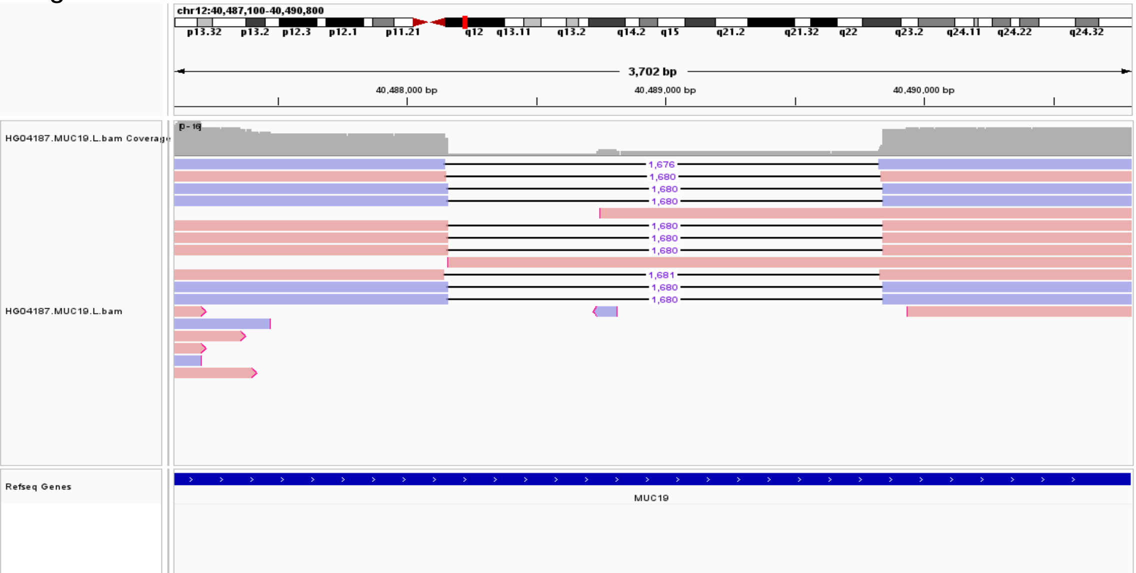

HG04199

Short−reads

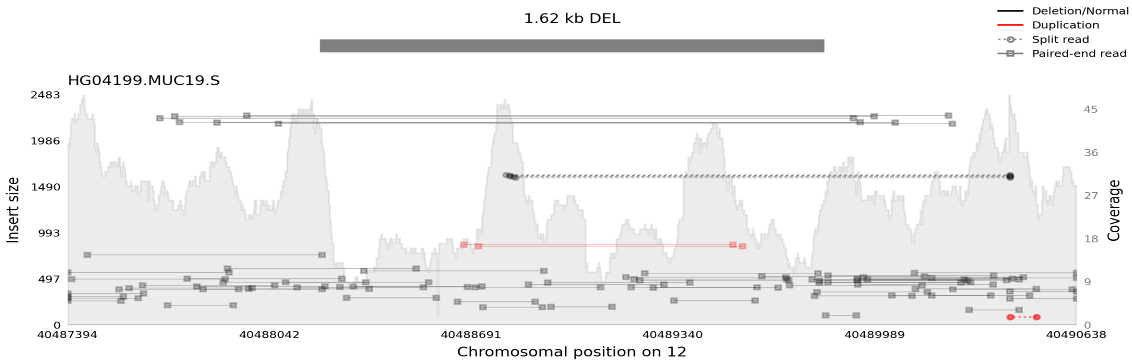

HG04199

Long−reads

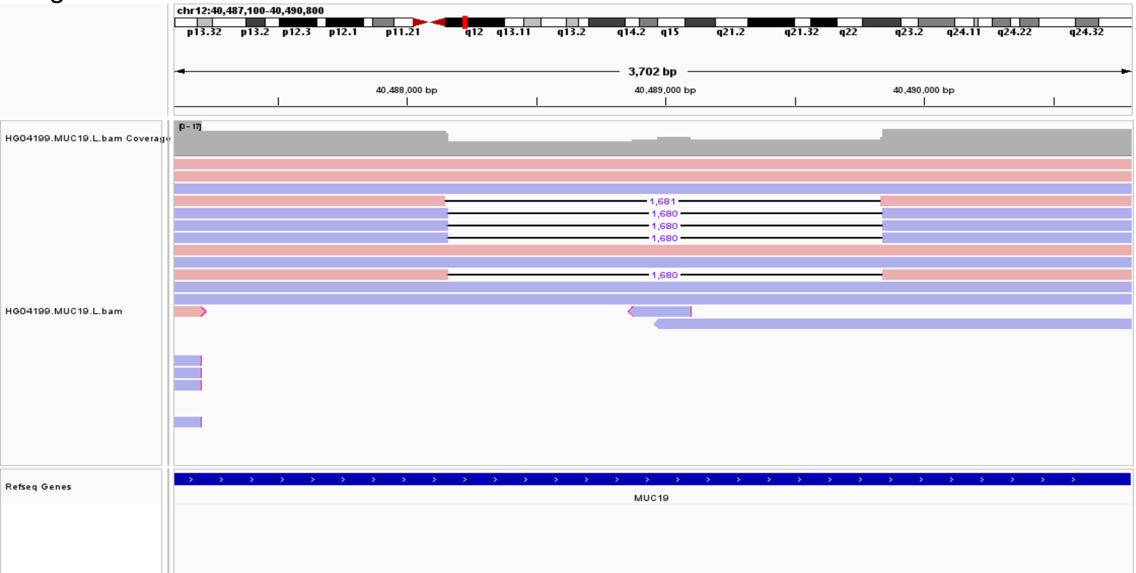

HG04204

Short−reads

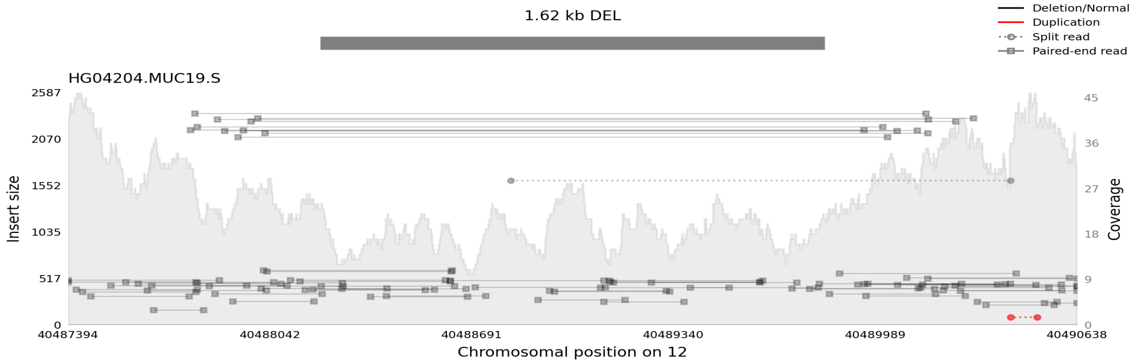

HG04204

Long−reads

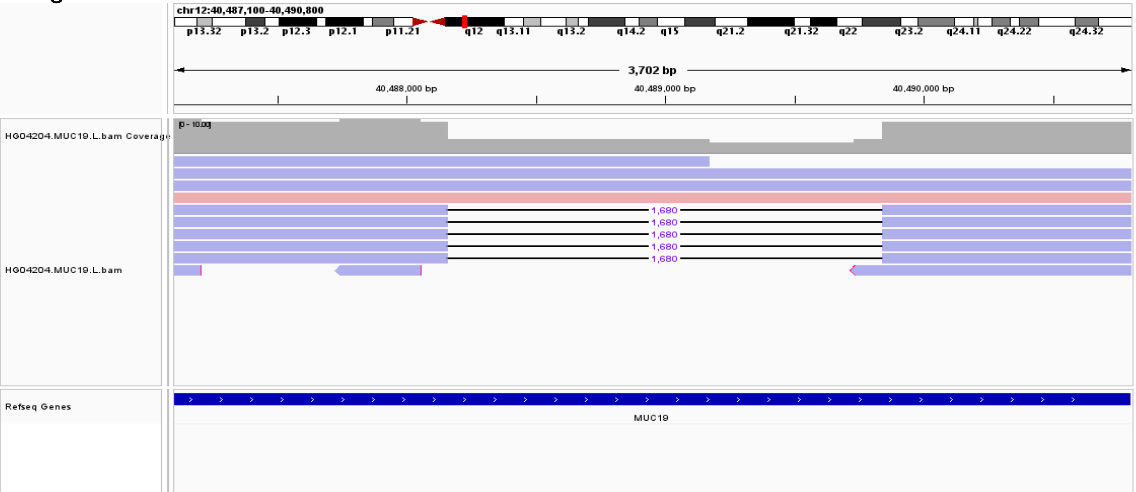

HG04217
Short−reads

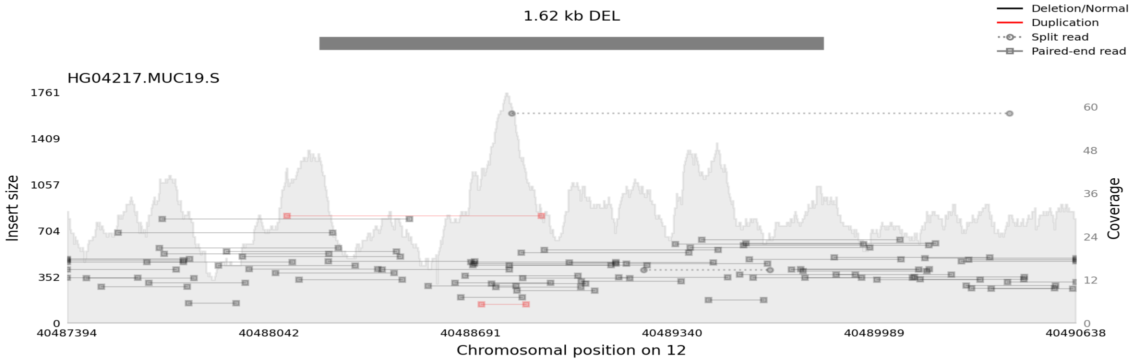

HG04217
Long−reads

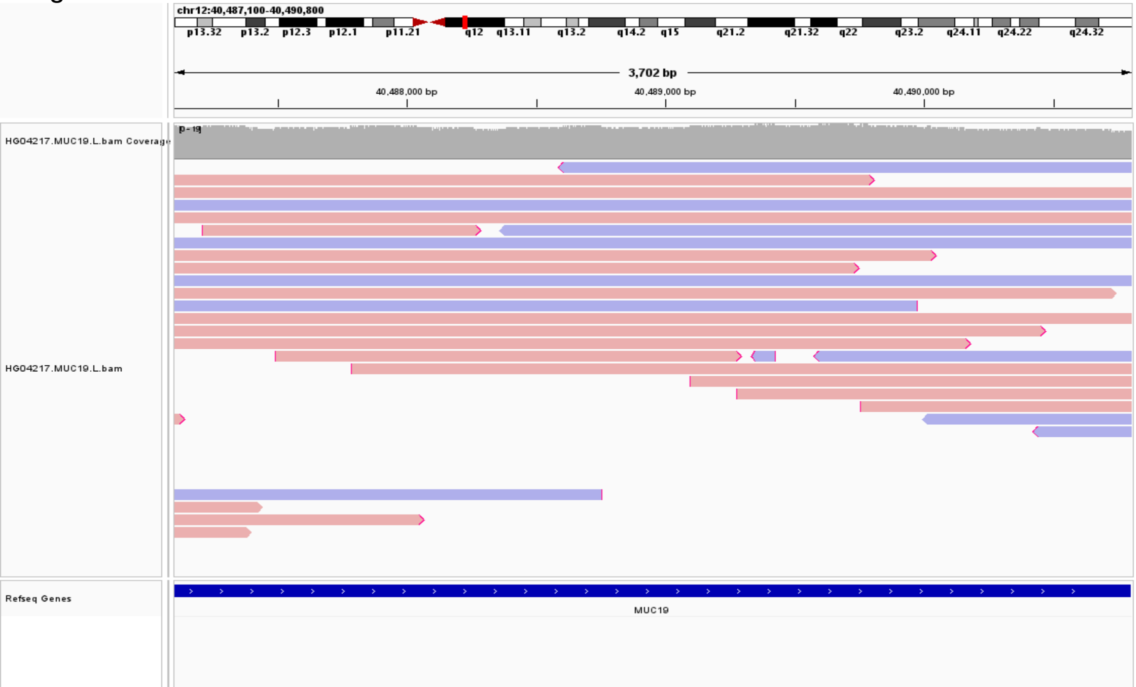

HG04228
Short−reads

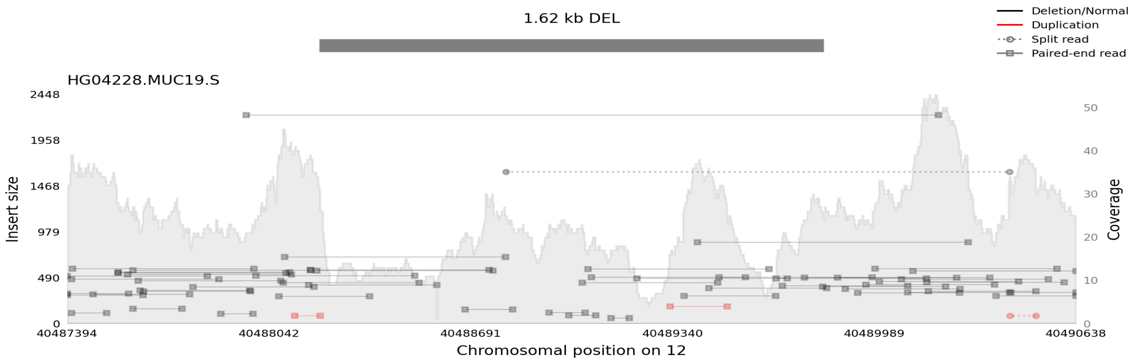

HG04228
Long−reads

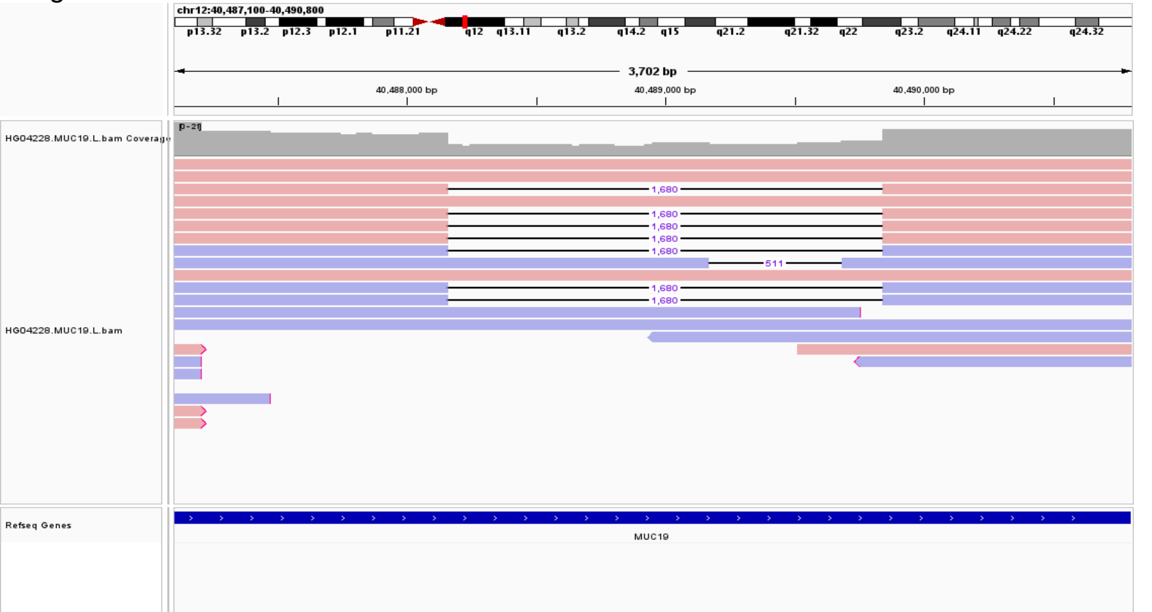

HX1

Short−reads

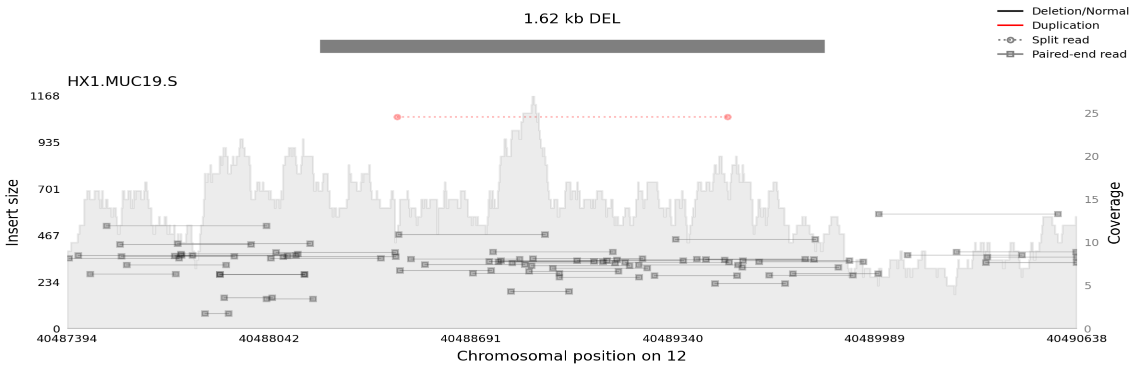

HX1

Long−reads

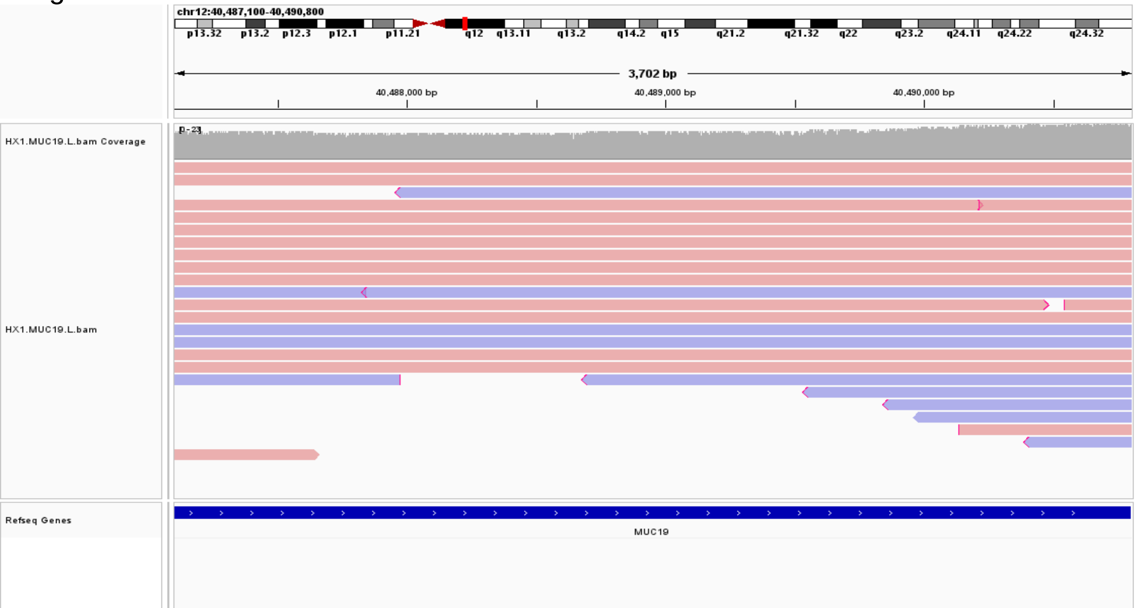

Korean

Short−reads

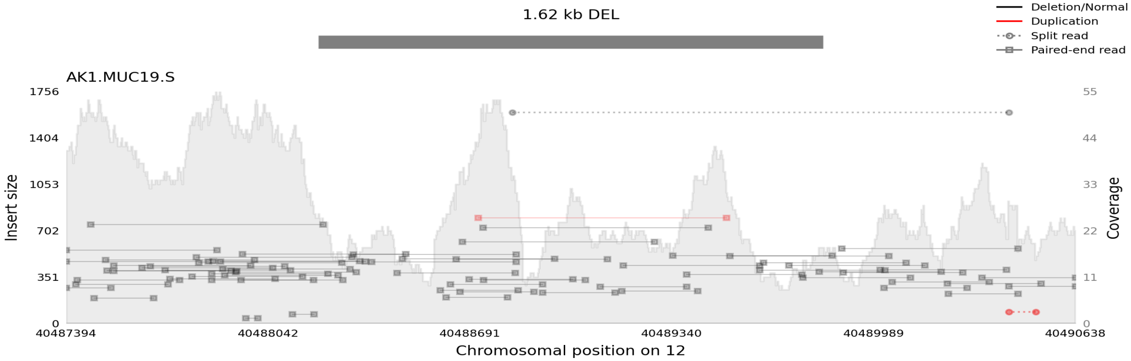

Korean

Long−reads

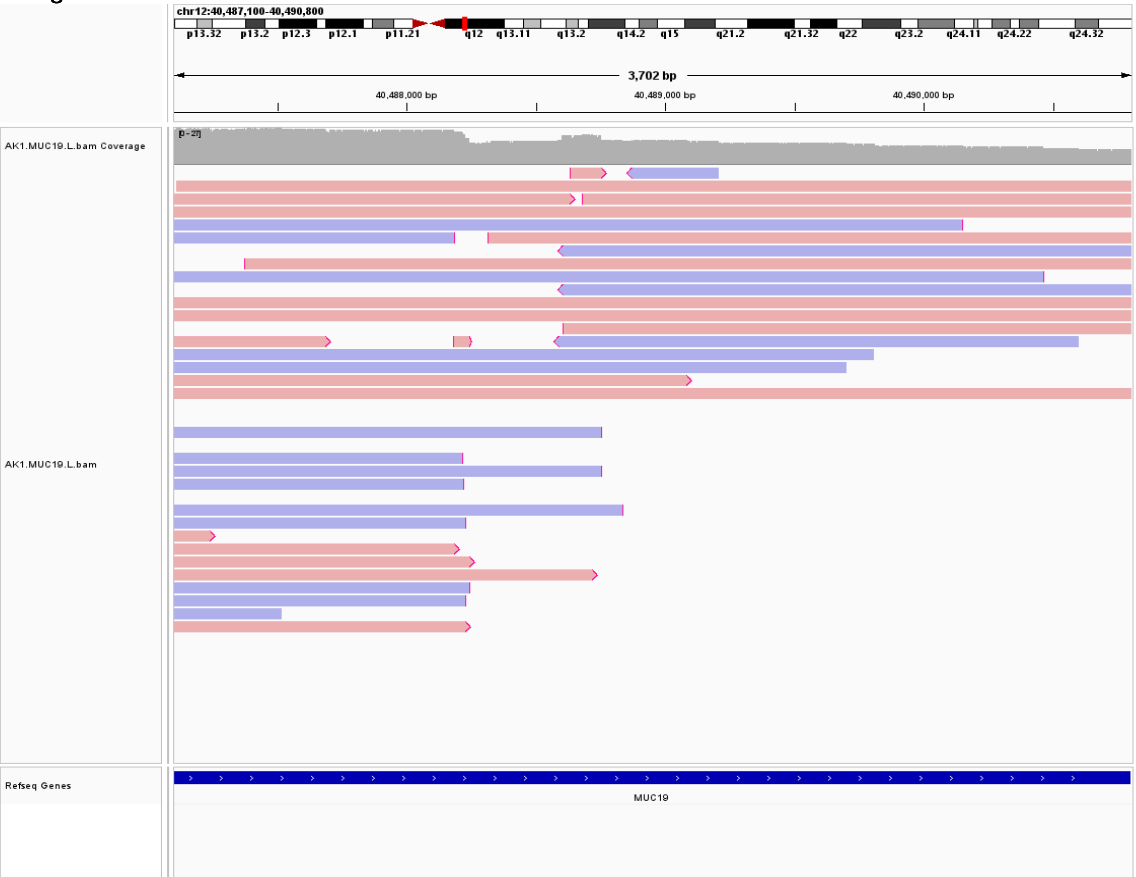

Supplement: Supplementary file 3 — Data set 1 [file 41531_2024_722_MOESM3_ESM.pdf]
